# Supplementary material for: High-throughput triazole-based combinatorial click chemistry for the synthesis and identification of functional metal complexes
Source: Nat Commun. 2025 Dec 23;16:11195. doi: 10.1038/s41467-025-67341-z (PMC12728216; doi:10.1038/s41467-025-67341-z)
Supplement: Supplementary file 1 — Supplementary Information [file 41467_2025_67341_MOESM1_ESM.pdf]

Supporting Information for

High-throughput Triazole-based Combinatorial Click Chemistry for the Synthesis and Identification of Functional Metal Complexes

*David R. Husbands[1], Çağrı Özsan[1,2], Athi Welsh[1,2], Richard J. Gammons[1], Angelo Frei[1,2]**

[1] Department of Chemistry, University of York, York YO10 5DD, U.K.

[2] Department of Chemistry, Biochemistry & Pharmaceutical Sciences, University of Bern, Freiestrasse 3, 3012 Bern, Switzerland

Table of Contents

1. General Information 2
   1.1 Compound Preparative Techniques 2
   1.2 Instrument Details and Methods for Compound Characterization 2
2. Experimental Details 3
   2.1 Preparation of Ligand libraries 3
   2.2 Preparation of Metal Libraries 4
   2.3 Synthesis of Organic Compounds 9
   2.4 Batch Synthesis of Target Ligands 10
   2.5 Synthesis of Target Metal Complexes 17
   2.6 Catalytic Testing 26
   2.7 Photophysical Properties 41
   2.8 Stability of Purified Compounds 44
   2.9 LCMS Processing 47
3. Biological Testing 51
   3.1 Single Dose Response (SDR) 52
   3.2 Minimum Inhibitory Concentration (MIC) 57
   3.3 Toxicity Testing 59
4. Structure-Activity Relationship Analysis 65
5. X-Ray Crystallography 74
6. NMR Spectral data for Organic and Inorganic Compounds 81
7. HRMS data for Organic and Inorganic Compounds 122
8. LCMS traces for Organic and Inorganic Compounds 140
9. FTIR data for Organic and Inorganic Compounds 156
10. References 174

1. General Information

1.1 Compound Preparative Techniques

Reagents were purchased from Merck, Fluorochem, Alfa Aesar, and Fisher and used without further purification. The purity of chemicals was confirmed by LCMS before use.

1.2 Instrument Details and Methods for Compound Characterization

NMR spectra were obtained in the solvent indicated in the text below, using a Bruker Avance Neo 700 instrument fitted with a liquid N_2 cooled triple resonance cryoprobe (700 MHz [^{1}H], 659 MHz [^{19}F], 176 MHz [^{13}C]) MHz Bruker AVIIIHD 600 Widebore instrument (600 MHz [^{1}H], 565 MHz [^{19}F], 151 MHz [^{13}C]) or JEOL ECX400 or JEOL ECS400 spectrometer (400 MHz [^{1}H], 101 MHz [^{13}C] and 377 MHz [^{19}F]). Chemical shifts (δ) are reported in parts per million (ppm) and were referenced to the residual non-deuterated solvent of the deuterated solvent used; $CDCl_3$: δ ^{1}H = 7.26 (C*H*Cl_3) and ^{13}C = 77.16 ($CDCl_3$); $(CD_3)_2SO$: ^{1}H = 2.50 ((CD_3)(CD_2*H*)SO) and ^{13}C = 39.52 ((*C*D_3)$_2$SO). Spectral data were typically collected at 298 K (25 °C), unless stated otherwise. All ^{1}H NMR signals are reported as they appear in the spectrum.

All ^{13}C NMR spectra were obtained with ^{1}H decoupling. All NMR spectra were processed using MestReNova (MNova) software (v. 15.1.0-37919).

High-resolution mass spectrometry (HRMS) ESI-MS spectra were measured using a Bruker Daltronics micrOTOF MS, Agilent series 1200LC with electrospray ionization (ESI) or on a Thermo LCQ using electrospray ionization, with <5 ppm error recorded for all HRMS samples. High resolution mass spectra (HRMS) are reported with <5 ppm error (ESI). For clarity, data are reported for the most abundant natural isotope of metals, which is part of 'exact mass' values. ESI ions are reported as the $[M+H]^+$ cation, unless a Na or K is present in the molecular formula, in which case the $[M+Na]^+$ or $[M+K]^+$ ion is being measured.

LCMS data was collected using a Dr. Maisch ReproSil-Pur 120 ODS-3, 2.4 µm, 50 mm x 3 mm column, on a ThermoScientific Vanquish instrument, coupled to a ThermoScientific HCT ultra ETD II ion trap. Mass spectrometry (ESI) was run in the positive mode. The solvent system consisted of H_2O / MeCN, both containing 0.1% formic acid. The gradient used for the "7 minute method" that was commonly used is described in Table S1. The UV channels correspond to UV1: 200 nm, UV2: 220 nm, UV3:254 nm. Characterization data and assessments of purity on the generated libraries were done using 254 nm absorbances.

Table S1: Flow gradient for LCMS "7 min method"

Time / min	Flow / mL min^{-1}	% MeCN
0.0	0.500	10.0
4.0	0.500	90.0
6.3	0.500	90.0
6.4	0.500	10.0
7.0	0.500	10.0

UV-Vis absorbance and fluorescence data was collected using a Tecan Infinite 200 Pro plate reader, using the "Greiner 96 Flat Bottom Transparent Polystyrene" option.

Infrared spectra were obtained using a PerkinElmer UATR Spectrum Two FTIR Spectrometer with a platinum-diamond ATR sampling module. All reaction temperatures were measured independently from the heating block using a TENMA 72-7715 electronic thermometer and thermocouple to ensure accurate reaction temperature regulation. 96-well reactions were shaken and heated using an Eppendorf ThermoMixer with "plates" attachment. Irradiation at 405 nm was achieved using a 96 LED array (250 mW/LED) (Atlas Photonics Lumos Bio).

For single crystal X-ray crystallographic analysis details, please see Section 8.

2. Experimental Details

2.1 Preparation of Ligand libraries

The following procedure was carried out using an Opentrons liquid handling robot OT2, utilizing both 300 µL and 20 µL pipettes (Gen2). This procedure generates ligands at nominal concentration of 20 mM in 400 µL of solvent. It should be noted that the solubility of some ligands is an issue, with a number precipitating or crystallizing out of solution over time. This can be mitigated to a certain extent by further dilution, or by sonicating the individual reaction vials to pulverize the crystals. For accurate additions, these ligand solutions were aspirated 3 times to ensure efficient mixing before dispensing.

Preparation of the FSO_2N_3 reagent

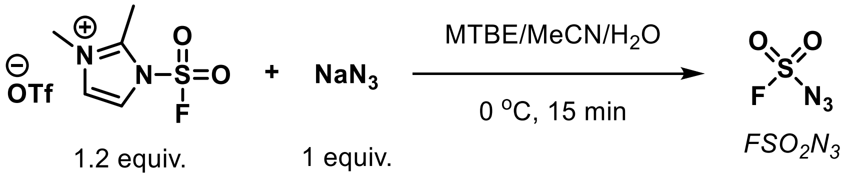

Method adapted from Meng *et al.*[1]

NaN_3 (99 mg, 1.524 mmol, 1 equiv.) was dissolved in deionized water (3.0 mL) in a 15 mL falcon tube. To this, 3.0 mL of MTBE was added and the mixture stirred vigorously at 0 °C. Separately, 1-(fluorosulfonyl)-2,3-dimethyl-1H-imidazol-3-ium trifluoromethanesulfonate (600 mg, 1.83 mmol, 1.2

equiv.) was dissolved in 150 µL of MeCN in a falcon tube, and was added to the reaction mixture. A further 150 µL of MeCN was used to wash the falcon tube and ensure efficient transfer of material, with this also being added to the reaction mixture. The mixture was stirred vigorously at 0 °C with the lid loosely screwed on top for 15 min, then allowed to settle at r.t. for 30 min The upper organic layer was decanted and diluted with 3 mL DMSO, giving a solution of FSO_2N_3 of approximately 173 mM (calculated according to the method described by Meng *et al.*).[1]

The following procedure assumes a concentration of 173 mM for FSO_2N_3 and describes the addition to 1 well of a Paradox® 96 well plate (700 µL glass vial) using a Opentrons OT2 liquid handling robot (P300 Gen2 300 µL pipette and P20 Gen2 20 µL pipette). It is also adapted from Meng *et al.*[1]

Preparation of the ligand library

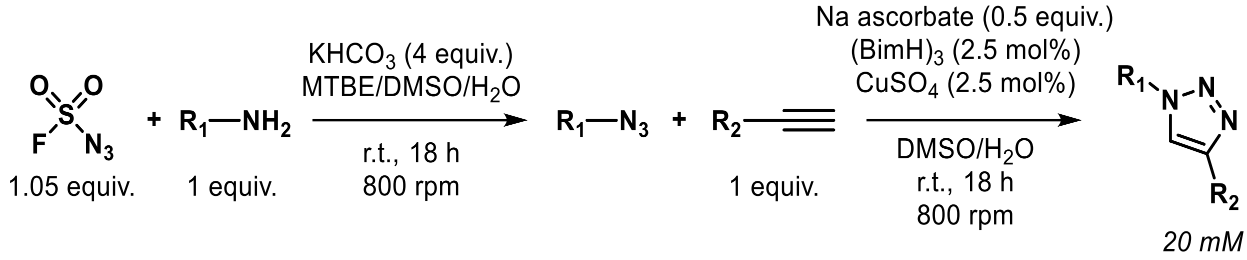

To the reaction well, sat. $KHCO_3$ solution (11 µL, 4 equiv., nominally 3 M), followed by DMSO (65 µL, Note: this is the volume required to make up the total volume to 200 µL, while additionally accounting for evaporation of MTBE, which is half the volume of the FSO_2N_3 stock solution) was added, then amine stock solution (100 µL, 80 mM, 1 equiv.). FSO_2N_3 stock solution was then added (48 µL, 173 mM 1.05 equiv., Note: the pipette was aspirated 3 times to equilibrate solvent vapor pressure. It is recommended to prepare a significant excess (25%) of the FSO_2N_3 stock solution to account for evaporation during addition). The reaction vials were then sealed, and shaken at 800 rpm for 18 h at r.t.

Next, to each reaction well, sodium ascorbate solution (80 µL, 50 mM, 0.5 equiv.) was added and the reaction shaken for a further 15 min at r.t.

To each reaction well, alkyne stock solution (100 µL, 80 mM, 1 equiv.) was added, followed by $(BimH)_3$ solution in DMSO (10 µL, 20 mM, 2.5 mol%), then $CuSO_4$ solution in water (10 µL, 20 mM, 2.5 mol%). The reaction was sealed, and shaken at 800 rpm for 18 h at r.t. The reaction mixture was analyzed by LCMS (5 µL in 200 µL MeCN / H_2O (60:40)) to confirm the presence and identity of the product ligand.

2.2 Preparation of Metal Libraries

Once made and characterized by LCMS, the libraries were stored at -30 °C until being used for further testing. Either 96 well polystyrene plates (TPP, Switzerland) or Paradox® metal plates containing 96x 700 µL glass vials were used as “plates”. Plates were shaken and/or heated in using an Eppendorf Thermomixer.

IrCN(Tz-4-P) Library

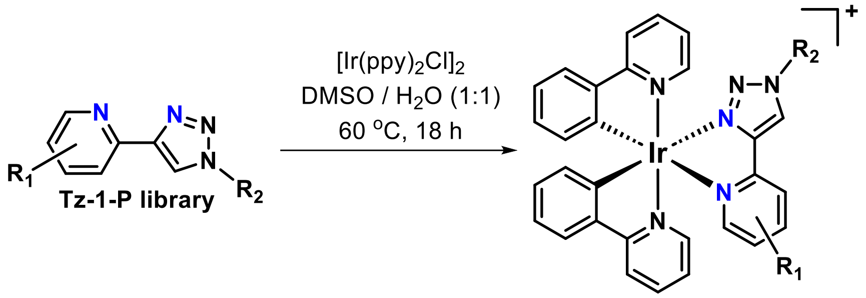

IrCN dimer ([Ir(ppy)$_2$Cl]$_2$, 34.3 mg, 0.032 mmol) was dissolved in DMSO (3.2 mL, 10 mM). Ligand stock solution (**Tz-4-P library**, 30 µL, 20 mM) was dispensed into 700 µL glass vials, with 30 µL of IrCN dimer stock solution added to each well, followed by 60 µL of water (giving 120 µL of 5 mM complexes). The plate was sealed and heated at 60 °C (600 rpm) for 18 h, before analysis by LCMS.

Re(CO)$_3$(Tz-4-P) Library

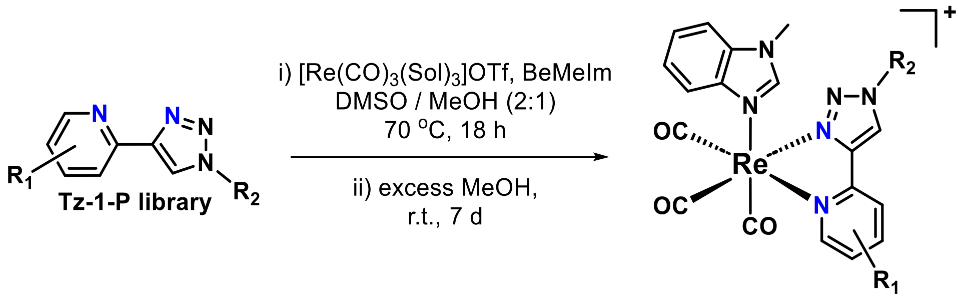

Re(CO)$_5$Cl (50.6 mg, 0.14 mmol) and AgOTf (36 mg, 0.14 mmol. Note: hydroscopic, light-sensitive) were placed in a 20 mL microwave vial. MeOH (7 mL) was added, and the mixture was heated in a Biotage Initiator microwave reactor at 120 °C for 30 mins. After cooling, the supernatant was used as a stock solution of [Re(CO)$_3$(MeOH)$_3$][OTf] (20 mM). Ligand stock solution (30 µL, 20 mM) and 1-methylbenzimidazole stock solution (30 µL, 20 mM, DMSO) were dispensed into 700 µL glass vials, with 30 µL of Re(CO)$_3$(MeOH)$_3$OTf stock solution added to each well. The plate was sealed, and heated at 70 °C (600 rpm) for 18 h. After this, 240 µL of MeOH was added to each well, and the plate was left unsealed for one week, allowing for the MeOH to evaporate off (giving 60 µL of 10 mM complexes). The plate was then analyzed by LCMS.

Re(CO)₃(Tz-4-P)Solvent Library

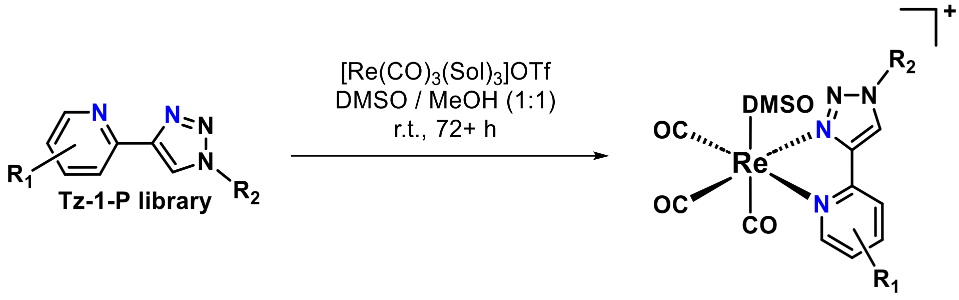

$Re(CO)_5Cl$ (50.6 mg, 0.14 mmol) and AgOTf (36 mg, 0.14 mmol. Note: hydroscopic, light-sensitive) were placed in a 20 mL microwave vial. MeOH (7 mL) was added, and the mixture was heated in a microwave reactor at 120 °C for 30 mins. After cooling, the supernatant was used as a stock solution of $Re(CO)_3(MeOH)_3OTf$ (20 mM). Ligand stock solution (30 µL, 20 mM) was dispensed into 700 µL glass vials, with 30 µL of $Re(CO)_3(MeOH)_3OTf$ stock solution added to each well. The plate was left standing unsealed for 1 week at r.t., allowing for the MeOH to evaporate off. 30 µL of DMSO was added to each well (giving 60 µL of 10 mM complexes), then the plate was analyzed by LCMS.

Mn(CO)₃(Tz-4-P) Library

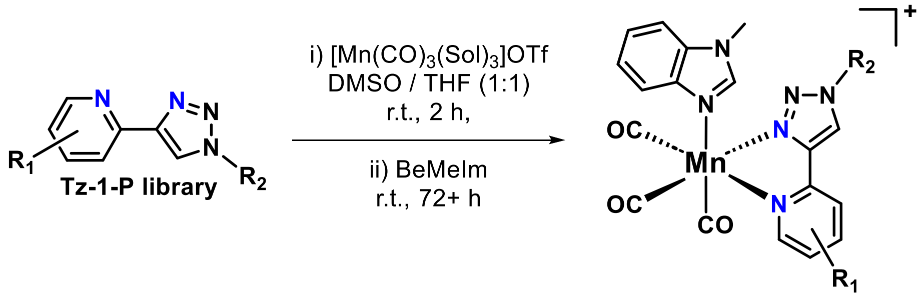

(Note: Mn complexes are light sensitive, so all steps were done in darkness. The pre-activation of Mn was done under N_2, but other steps were done under ambient conditions) $Mn(CO)_5Br$ (38.5 mg, 0.14 mmol) and AgOTf (36 mg, 0.14 mmol. Note: hydroscopic, light-sensitive) were placed in a 50 mL 2-necked round bottomed flask fitted with a condenser, and evacuated/backfilled with N_2 three times. Dry THF (7 mL) was added, and the mixture was refluxed under N_2 at 70 °C for 45 mins. After cooling, the supernatant was used as a stock solution of $Mn(CO)_3(THF)_3OTf$ (20 mM). Ligand stock solution (30 µL, 20 mM) was dispensed into 700 µL glass vials, with 30 µL of $Mn(CO)_3(THF)_3OTf$ stock solution added to each well. After 2 h at r.t., 1-methylbenzimidazole stock solution (30 µL, 20 mM, DMSO) was added, and the plate was left shaking at r.t. (600 rpm) for 72 h, allowing for the THF to evaporate off (giving 60 µL of 10 mM complexes). The plate was then analyzed by LCMS.

$Mn(CO)_3$(Tz-4-P)Solvent Library

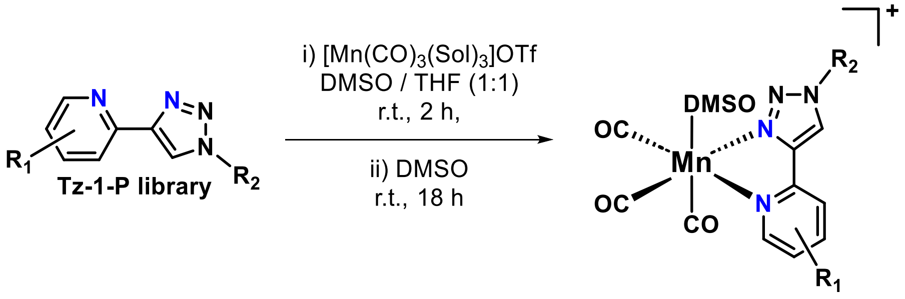

$Mn(CO)_5Br$ (38.5 mg, 0.14 mmol) and AgOTf (36 mg, 0.14 mmol. Note: hydroscopic, light-sensitive) were placed in a 50 mL 2-necked round bottomed flask fitted with a condenser, and evacuated/backfilled with N_2 three times. Dry THF (7 mL) was added, and the mixture was refluxed under N_2 at 70 °C for 45 mins. After cooling, the supernatant was used as a stock solution of $Mn(CO)_3(THF)_3OTf$ (20 mM). Ligand stock solution (30 µL, 20 mM) was dispensed into 700 µL glass vials, with 30 µL of $Mn(CO)_3(THF)_3OTf$ stock solution added to each well. After 2 h at r.t., DMSO (30 µL) was added, and the plate was left shaking at r.t. (600 rpm) for 18 h, allowing for the THF to evaporate off (giving 60 µL of 10 mM complexes). The plate was then analyzed by LCMS. (Note: Mn complexes are light sensitive, so all steps were done in darkness. The pre-activation of Mn was done under N_2, but other steps were done under ambient conditions)

IrCp*(Tz-4-P) and IrCp*(Tz-1-MP) Libraries

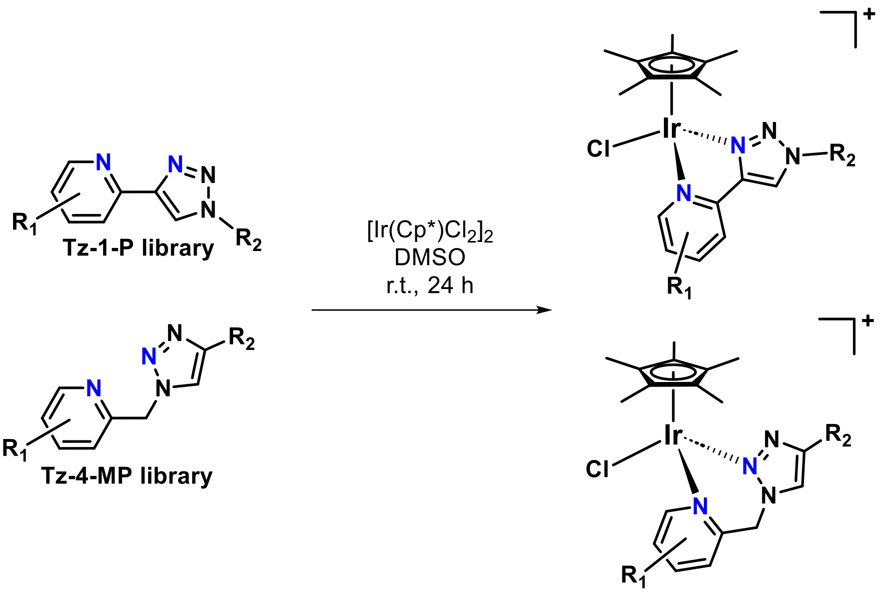

$[IrCp^*Cl_2]_2$ (IrCp*, 49.4 mg, 0.062 mmol) was dissolved in DMSO (6.2 mL, 10 mM). Ligand stock solution (30 µL, 20 mM) was dispensed into a polystyrene 96 well plate, with 30 µL of IrCp* stock solution added to each well (giving 60 µL of 10 mM complexes). The plate was left stationary for 24 h at r.t., before analysis by LCMS.

RuCy(Tz-4-P) Library

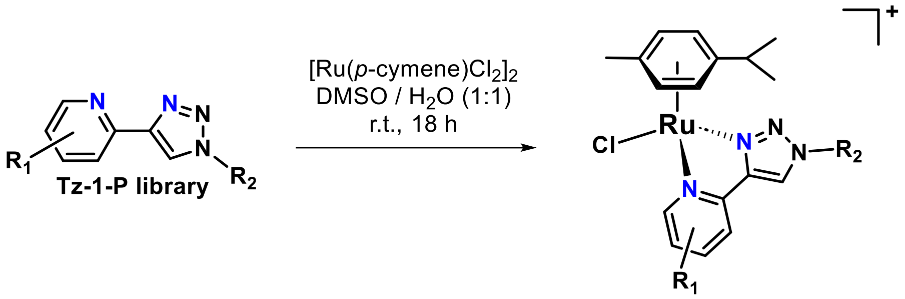

$[Ru(p\text{-cymene})Cl_2]_2$ (RuCy, 21.4 mg, 0.035 mmol) was dissolved in DMSO (3.5 mL, 10 mM). Ligand stock solution (30 µL, 20 mM) was dispensed into a polystyrene 96 well plate, with 30 µL of RuCy stock solution added to each well, followed by 60 µL water (giving 120 µL of 5 mM complexes). The plate was left stationary for 18 h at r.t., before analysis by LCMS.

RuCy(Tz-1-MP) Library

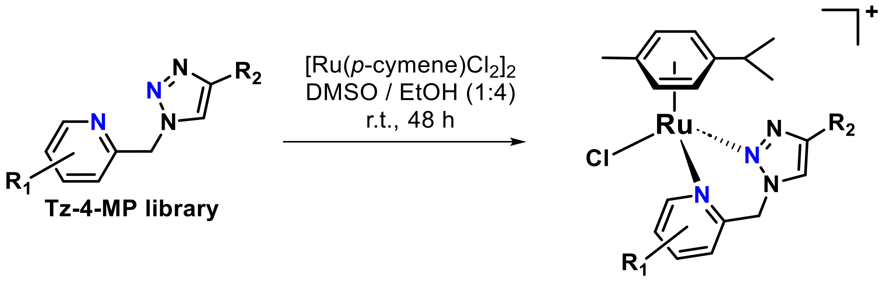

$[Ru(p\text{-cymene})Cl_2]_2$ (RuCy, 38.3 mg, 0.0625 mmol) was dissolved in EtOH (25 mL, 2.5 mM). Ligand stock solution (60 µL, 20 mM) was dispensed into 700 µL glass vials, with 240 µL of RuCy stock solution added to each well. The plate was sealed and shaken at r.t. (600 rpm) for 48 h, after which the EtOH was evaporated under a flow of nitrogen, and the compounds diluted with DMSO (120 µL of 10 mM compounds), before analysis by LCMS.

2.3 Synthesis of Organic Compounds

Synthesis of $(BimH)_3$ ligand (lab book ref. DRH-008)

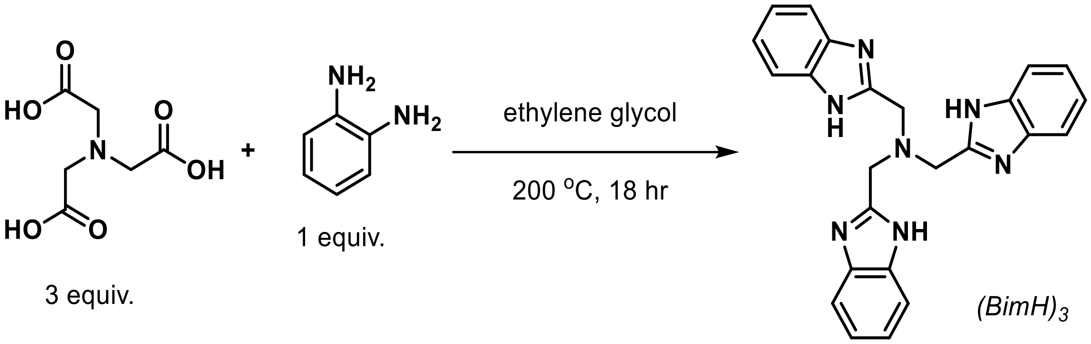

(adapted from patent N. WO2020/89630, 2020, A1, para. 0190-0191)

Nitrilotriacetic acid (2.49 g, 13 mmol, 1 equiv.), o-phenylenediamine (4.22 g, 39 mmol, 3 equiv.) and ethylene glycol (30 mL) were charged into a round bottom flask fitted with an air condenser. The reaction was heated to 200 °C using a metal heating block and stirred for 18 h, before being allowed to cool. The mixture was poured into a beaker containing ice-water, and stirred for 5 min, giving a thick beige precipitate. This precipitate was collected by filtration, transferred to a round bottom flask with 50 mL of MeOH and charcoal (2 heaped tsp) and refluxed for 30 min. The mixture was filtered hot, and the MeOH evaporated to approx. 15 mL in a conical flask. The flask was cooled on ice, and upon scratching, crystals formed. These were isolated by filtration, washed with a small quantity of cold MeOH to remove the red impurity, and dried *in vacuo*, giving the product.

Beige crystals (1.262 g, 24%);

^1H NMR (600 MHz, DMSO-d_6) δ 12.60 – 12.34 (m, 3H), 7.57 (d, J = 45.7 Hz, 6H), 7.29 – 7.09 (m, 6H), 4.15 (s, 6H);

^{13}C NMR (151 MHz, DMSO-d_6) δ 152.2, 143.2, 134.2, 122.0, 121.1, 118.6, 111.2, 51.4;

HRMS (ESI+) $(C_{24}H_{22}N_7)^+$ m/z (calculated) 408.1931, (found) 408.1937, mass difference 1.3 ppm;

(ATIR): ṽ (cm^{-1}) 3053 (br), 1622, 1536, 1434, 1272, 1215, 1116, 1022, 735 (s), 543.

Synthesis of Coumarin-N_3 (lab book ref. DRH-030)

The method was adapted from a literature procedure.[2]

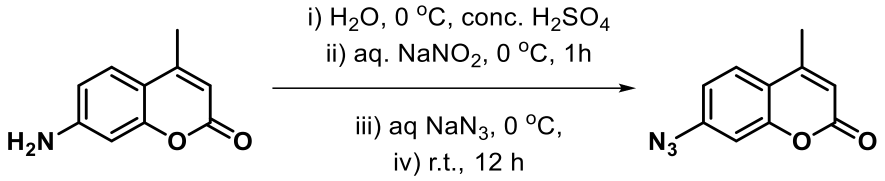

7-amino-4-methylcoumarin (250 mg, 1.43 mmol, 1 equiv.) was added to water (5.5 mL) and stirred at 0 °C. Conc. H_2SO_4 (1.5 mL) was added dropwise. Separately, $NaNO_2$ (125 mg, 1.81 mmol, 1.3 equiv.) was dissolved in water (1.7 mL), cooled to 0 °C and added to the reaction mixture dropwise by pipette over 30 mins. The resulting mixture was stirred at 0 °C for 1 h. NaN_3 was dissolved in water (1 mL), cooled to 0 °C and added to the reaction mixture dropwise, resulting in a white precipitate forming. After 20 mins at r.t., the precipitate was collected by filtration, washed with water (50 mL) and air dried. After this, the precipitate was dissolved in $CHCl_3$, dried ($MgSO_4$), filtered and the solvent removed *in vacuo* to give the product. NMR data match the literature.

Yellow solid, 176 mg (61% yield);

^{1}H NMR (400 MHz, DMSO-d_6) δ 7.75 (d, *J* = 8.3 Hz, 1H), 7.15 – 7.07 (m, 2H), 6.32 (q, *J* = 1.3 Hz, 1H), 2.40 (d, *J* = 1.3 Hz, 3H);

^{13}C NMR (101 MHz, DMSO-d_6) δ 159.5, 154.0, 152.9, 143.3, 127.0, 116.7, 115.5, 113.2, 106.8, 18.1;

HRMS (ESI+) $(C_{37}H_{30}IrN_6O_2)^+$ m/z (calculated) 783.2054, (found) 783.2057, mass difference 0.3 ppm;

(ATIR): ṽ (cm^{-1}) 3044 (C-H aromatic), 2117 (N=N=N), 2094 (N=N=N), 1720, 1606, 1389, 1293, 1273, 1163, 1067, 979, 872, 855, 817, 748, 731, 704, 621, 523.

2.4 Batch Synthesis of Target Ligands

General procedure

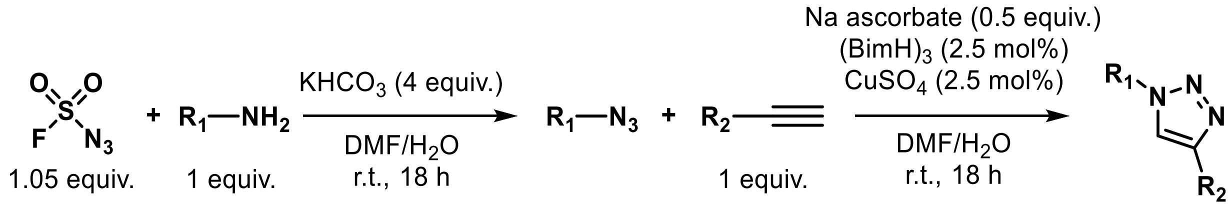

FSO_2N_3 was synthesized as before and diluted with DMF to approx. 200 mM in 1:1 MTBE:DMF (by volume). The desired amine (0.56 mmol, 1.0 equiv.) was charged into a 50 mL polypropylene Falcon tube, followed by DMF (7 mL). Sat. $KHCO_3$ solution (0.8 mL, approx. 2.4 mmol, 4 equiv. in water) was added, followed by FSO_2N_3 solution (1.05 equiv.). The resulting mixture was stirred at r.t. for 18 h.

Sodium ascorbate solution (5.6 mL, 50 mM, 0.5 equiv. in water) was added, and the mixture was stirred for a further 10 mins. 7 mL of DMF was added, followed by alkyne (0.56 mmol, 1.0 equiv.), then $(BimH)_3$ solution (0.7 mL, 20 mM, 2.5 mol% in DMF) and $CuSO_4$ solution (0.7 mL, 20 mM, 2.5 mol% in water). The resulting mixture was stirred for a further 18 h at r.t.

The crude reaction mixture was analyzed by LCMS, then 100 mL of deionized water was added. The mixture was extracted with 3:1 $CHCl_3$: isopropyl alcohol (3 x 30 mL), followed by EtOAc (2 x 30 mL). The combined organic layers were washed with 5 wt% LiCl solution (5 x 25 mL), dried ($MgSO_4$), filtered, and the solvent evaporated. The residue was redissolved in EtOAc (40 mL) and washed with more LiCl solution (3 x 20 mL). The organic layers were dried ($MgSO_4$), filtered, and the solvent evaporated to give the crude product. Purification was achieved by flash column chromatography (Combiflash, 12g SiO_2 cartridges, 0% to 100% gradient of EtOAc in hexane. All compounds were loaded 'wet' by dissolution in the minimum volume of EtOAc).

M1Y1 (lab book ref. DRH-051-1)

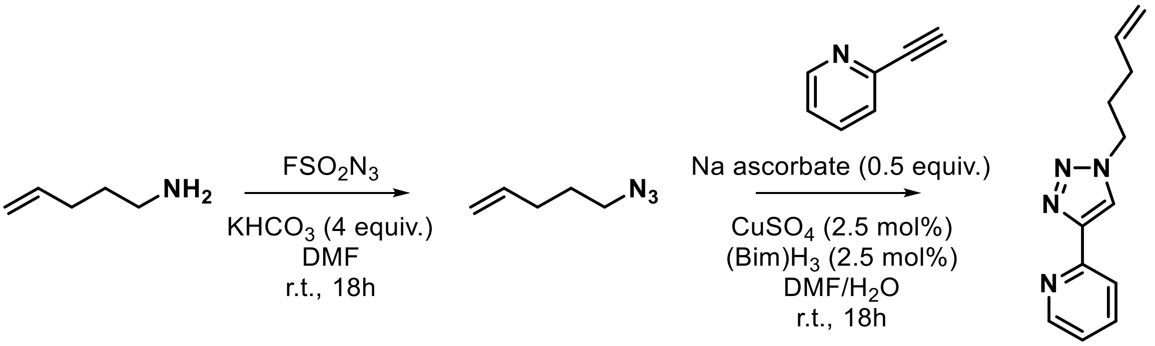

M1Y1

Colorless oil, 115 mg (96% yield), R_f = 0.17 (40% EtOAc in hexane);

^{1}H NMR (600 MHz, C*D*Cl$_3$) δ 8.55 (ddd, *J* = 4.9, 1.8, 0.9 Hz, 1H), 8.16 (dt, *J* = 7.9, 1.1 Hz, 1H), 8.12 (s, 1H,), 7.75 (td, *J* = 7.7, 1.8 Hz, 1H,), 7.20 (ddd, *J* = 7.5, 4.9, 1.2 Hz, 1H), 5.77 (ddt, *J* = 16.9, 10.2, 6.5 Hz, 1H), 5.08 – 5.00 (m, 2H), 4.41 (t, *J* = 7.0 Hz, 2H), 2.14 – 2.01 (m, 4H);

^{13}C NMR (151 MHz, C*D*Cl$_3$) δ 150.5, 149.5, 148.5, 137.0, 136.5, 122.9, 122.0, 120.3, 116.4, 49.8, 30.4, 29.3;

HRMS (ESI+) $(C_{12}H_{14}N_4Na)^+$ m/z (calculated) 237.1111, (found) 237.1114, mass difference 0.8 ppm;

LCMS retention time (ESI+, 7 min method, H_2O:MeCN 0.1% formic acid): 2.76 min, m/z 215.15;

(ATIR): ṽ (cm^{-1}) 3078 (C-H aromatic), 2937 (C-H aliphatic), 1603, 1473, 1420, 1360, 1247, 1198, 1042, 995, 914, 782 (s), 745, 620, 518.

M8Y4 (lab book ref. DRH-051-2)

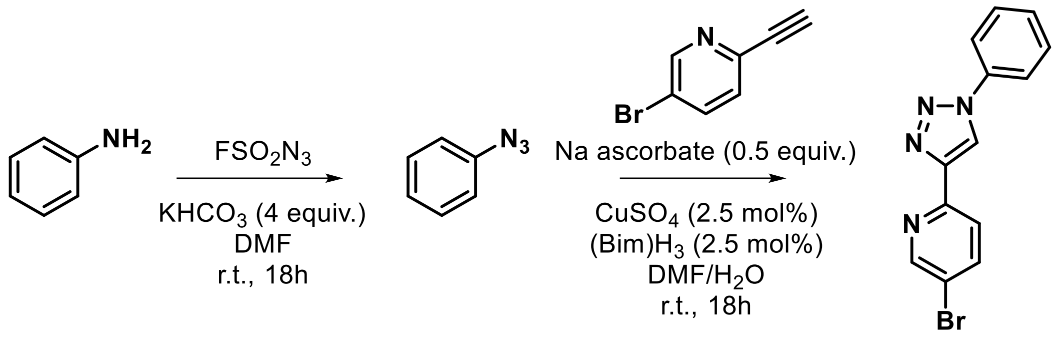

M8Y4

Crystaline white solid, 107 mg (64% yield), R_f = 0.75 (40% EtOAc in hexane);

^{1}H NMR (600 MHz, C*D*Cl$_3$) δ 8.65 (dd, *J* = 2.4, 0.7 Hz, 1H), 8.57 (s, 1H), 8.14 (dd, *J* = 8.4, 0.7 Hz, 1H), 7.93 (dd, *J* = 8.4, 2.4 Hz, 1H), 7.82 – 7.78 (m, 2H), 7.57 – 7.52 (m, 2H), 7.48 – 7.44 (m, 1H);

^{13}C NMR (151 MHz, C*D*Cl$_3$) δ 150.7, 148.6, 148.2, 139.7, 137.0, 130.0, 129.1, 121.7, 120.6, 120.3, 119.9;

HRMS (ESI+) ($C_{13}H_9BrN_4Na$)$^+$ m/z (calculated) 322.9903, (found) 322.9900, mass difference 0.4 ppm;

LCMS retention time (ESI+, 7 min method, H_2O:MeCN 0.1% formic acid): 4.02 min, m/z 301.20;

(ATIR): ṽ (cm^{-1}) 3134 (C-H aromatic), 1594, 1503, 1459, 1361, 1235, 1030, 1001, 823, 687, 539.

M12Y1 (lab book ref. DRH-051-3)

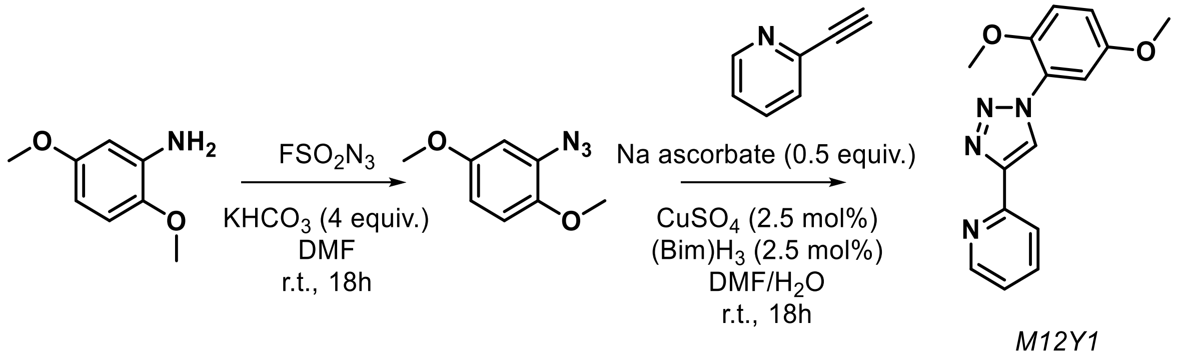

M12Y1

Pink solid, 125 mg (79% yield), R_f = 0.22 (40% EtOAc in hexane);

^{1}H NMR (600 MHz, C*D*Cl$_3$) δ 8.75 (s, 1H), 8.62 (ddd, *J* = 4.9, 1.8, 1.0 Hz, 1H), 8.25 (dt, *J* = 7.9, 1.1 Hz, 1H), 7.80 (td, *J* = 7.7, 1.8 Hz, 1H), 7.48 (d, *J* = 3.0 Hz, 1H), 7.24 (ddd, *J* = 7.6, 4.8, 1.2 Hz, 1H), 7.03 (d, *J* = 9.1 Hz, 1H), 6.97 (dd, *J* = 9.1, 3.1 Hz, 1H), 3.86 (s, 3H), 3.83 (s, 3H);

^{13}C NMR (151 MHz, $CDCl_3$) δ 154.0, 150.6, 149.6, 148.0, 145.2, 137.0, 126.6, 124.2, 122.9, 120.6, 115.9, 113.7, 110.5, 56.7, 56.1;

HRMS (ESI+) $(C_{15}H_{14}N_4NaO_2)^+$ m/z (calculated) 305.1009, (found) 305.1015, mass difference 1.8 ppm;

LCMS retention time (ESI+, 7 min method, H_2O:MeCN 0.1% formic acid): 3.20 min, m/z 283.16;

(ATIR): ṽ (cm^{-1}) 3140 (C-H aromatic), 2949, 2837, 1603, 1510, 1471, 1427, 1274, 1231, 1220, 1149, 1041, 1016, 865, 786, 743.

M20Y3 (lab book ref. DRH-051-5)

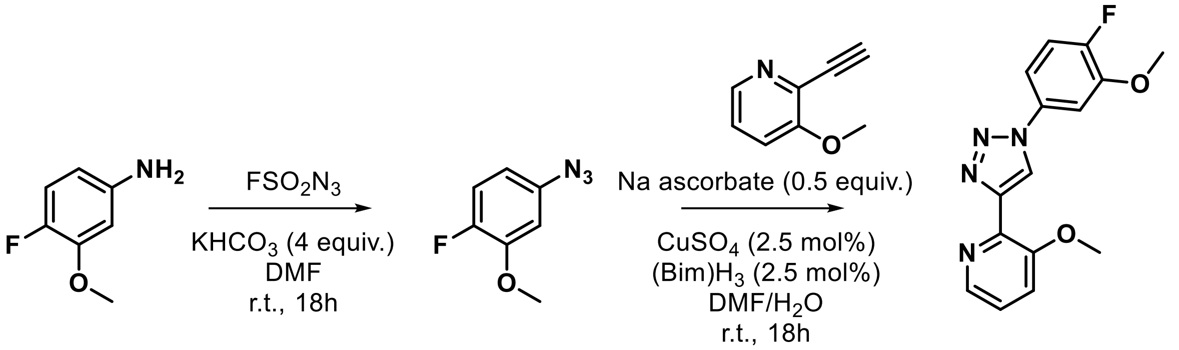

Cream solid, 34 mg (20% yield), R_f = 0.32 (100% EtOAc);

^{1}H NMR (600 MHz, $CDCl_3$) δ 8.49 (s, 1H), 8.40 (dd, *J* = 4.6, 1.3 Hz, 1H), 7.56 – 7.51 (m, 1H), 7.32 (dd, *J* = 8.4, 1.3 Hz, 1H), 7.26 (dd, *J* = 8.4, 4.5 Hz, 1H), 7.21 – 7.16 (m, 2H), 3.98 (s, 3H), 3.97 (s, 3H);

^{19}F NMR (565 MHz, $CDCl_3$) δ -133.59 – -134.71 (m);

^{13}C NMR (151 MHz, $CDCl_3$) δ 153.14, 152.83, 151.49, 148.72 (d, *J* = 11.8 Hz), 142.00, 141.9 (d, *J* = 903 Hz), 133.66 (d, *J* = 3.4 Hz), 123.58, 122.79, 118.35, 116.63 (d, *J* = 20.1 Hz), 112.60 (d, *J* = 7.3 Hz), 107.21 (d, *J* = 2.3 Hz), 56.73, 55.74;

HRMS (ESI+) $(C_{15}H_{13}FN_4NaO_2)^+$ m/z (calculated) 323.0915, (found) 323.0924, mass difference 3.2 ppm;

LCMS retention time (ESI+, 7 min method, H_2O:MeCN 0.1% formic acid): 2.81 min, m/z 301.21;

(ATIR): ṽ (cm^{-1}) 3174 (C-H aromatic), 3035, 2978, 1845, 1621, 1521, 1481, 1469, 1438, 1352, 1276, 1228, 1129, 1119, 846, 792, 781, 765, 676, 618, 586.

M19Y1 (lab book ref. DRH-066-1)

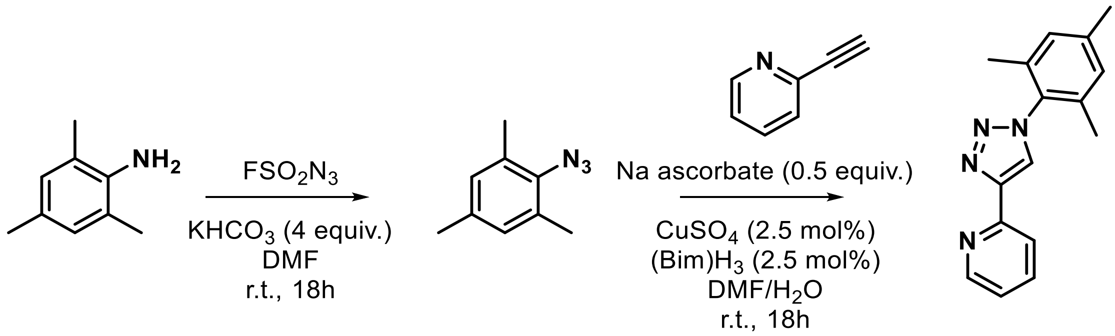

M19Y1

Viscous yellow oil, 111 mg (75% yield), R_f = 0.17 (20% EtOAc in hexane);

^{1}H NMR (700 MHz, C*D*Cl3) δ 8.61 (ddd, *J* = 4.9, 1.8, 0.9 Hz, 1H), 8.29 (dt, *J* = 7.9, 1.1 Hz, 1H), 8.21 (s, 1H), 7.83 (td, *J* = 7.7, 1.8 Hz, 1H), 7.26 (m, 1H), 7.01 (s, 2H), 2.37 (s, 3H), 2.02 (s, 6H);

^{13}C NMR (176 MHz, C*D*Cl3) δ 150.4, 149.6, 148.3, 140.3, 137.2, 135.2, 133.6, 129.3, 124.2, 123.1, 120.5, 21.3, 17.5;

HRMS (ESI+) $(C_{16}H_{16}N_4Na)^+$ m/z (calculated) 287.1275, (found) 287.1267, mass difference 2.6 ppm;

LCMS retention time (ESI+, 7 min method, H_2O:MeCN 0.1% formic acid): 3.64 min, m/z 265.206;

(ATIR): ṽ (cm^{-1}) 3144 (C-H aromatic), 2923, 2852, 1596, 1571, 1495, 1471, 1426, 1404, 1230, 1103, 1030, 995, 852, 784, 712, 580, 515, 403.

M22Y1 (lab book ref. DRH-066-2)

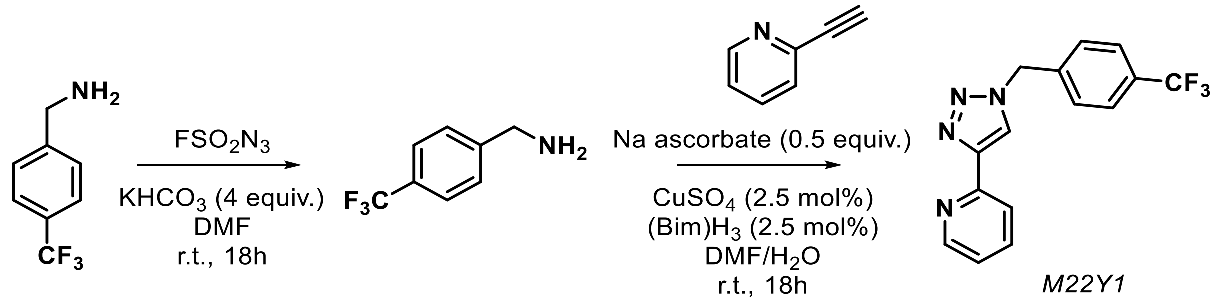

M22Y1

White solid, 117 mg (69% yield), R_f = 0.21 (50% EtOAc in hexane);

^{1}H NMR (700 MHz, C*D*Cl3) δ 8.59 – 8.51 (m, 1H), 8.18 (dd, *J* = 8.1, 1.4 Hz, 1H), 8.09 (s, 1H), 7.78 (td, *J* = 7.7, 1.8 Hz, 1H), 7.64 (d, *J* = 7.9 Hz, 2H), 7.43 (d, *J* = 8.0 Hz, 2H), 7.25 – 7.20 (m, 1H), 5.65 (s, 2H);

^{19}F NMR (659 MHz, C*D*Cl3) δ -62.80 (s);

^{13}C NMR (176 MHz, $CDCl_3$) δ 149.98, 149.39, 149.07, 138.35, 137.04, 131.17 (q, ^2J = 32.8 Hz), 128.44, 126.20 (q, ^3J = 3.8 Hz), 123.8 (q, ^1J = 272.6 Hz), 123.07, 122.05, 120.31, 53.71;

HRMS (ESI+) $(C_{15}H_{12}F_3N_4)^+$ m/z (calculated) 305.1017, (found) 305.1009, mass difference 2.9 ppm;

LCMS retention time (ESI+, 7 min method, H_2O:MeCN 0.1% formic acid): 3.36 min, m/z 305.169;

(ATIR): ṽ (cm^{-1}) 3119, 2923, 2853, 1606, 1471, 1431, 1325, 1226, 1164, 1123, 1067, 1018, 995, 778, 753, 592, 512, 409.

P1A7 (lab book ref. DRH-085-2)

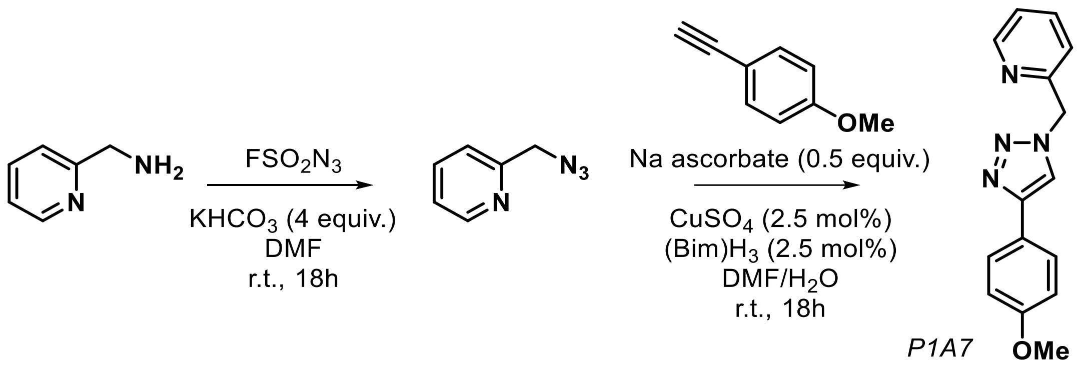

Cream solid, 112 mg (75% yield, Rf = 0.35 (70% EtOAc in hexane)

^1H NMR (700 MHz, $CDCl_3$) δ 8.61 (ddd, J = 4.9, 1.8, 0.9 Hz, 1H), 7.84 (s, 1H), 7.77 – 7.73 (m, 2H), 7.69 (td, J = 7.7, 1.8 Hz, 1H), 7.28 – 7.25 (m, 1H), 7.22 (dt, J = 7.8, 1.0 Hz, 1H), 6.96 – 6.92 (m, 2H), 5.68 (s, 2H), 3.83 (s, 3H).

^{13}C NMR (176 MHz, $CDCl_3$) δ 159.75, 154.77, 149.89, 148.27, 137.52, 127.17, 123.56, 123.39, 122.58, 119.49, 114.36, 55.86, 55.45.

HRMS (ESI+) $(C_{15}H_{15}N_4O)^+$ m/z (calculated) 267.1240, (found) 267.1241, mass difference 0.1 ppm;

LCMS retention time (ESI+, 7 min method, H_2O:MeCN 0.1% formic acid): 2.74 min, m/z 267.11;

(ATIR): ṽ (cm^{-1}) 3098, 2965, 2839, 1616, 1588, 1562, 1498, 1475, 1454, 1443, 1436, 1358, 1304, 1264, 1246, 1176, 1105, 1029, 976, 824, 748, 716, 610, 596, 530, 402.

P4A11 (lab book ref. DRH-085-3)

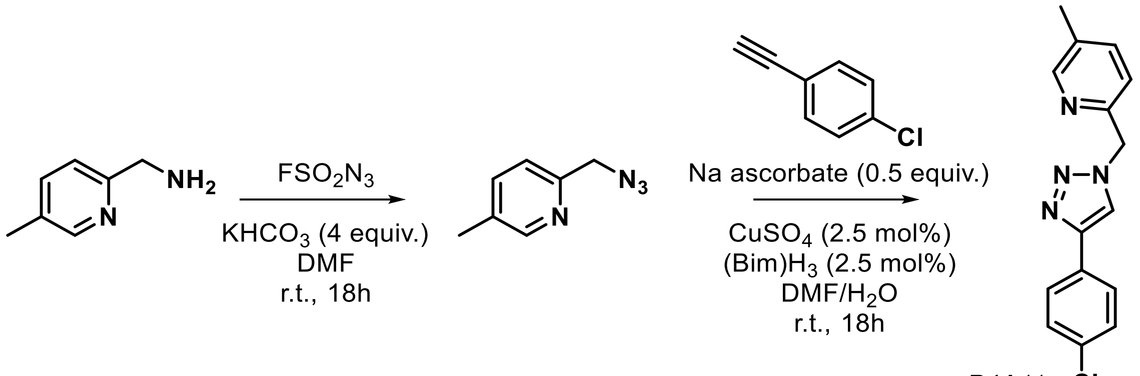

P4A11

White solid, 91 mg (57% yield), R_f = 0.28 (50% EtOAc in hexane);

^{1}H NMR (700 MHz, C*D*Cl$_3$) δ 8.46 – 8.41 (m, 1H), 7.91 (s, 1H), 7.78 – 7.72 (m, 2H), 7.50 (ddd, *J* = 7.9, 2.1, 0.9 Hz, 1H), 7.40 – 7.35 (m, 2H), 7.18 (d, *J* = 7.9 Hz, 1H), 5.65 (s, 2H), 2.34 (s, 3H);

^{13}C NMR (176 MHz, C*D*Cl$_3$) δ 151.5, 150.4, 147.3, 138.0, 134.0, 133.5, 129.3, 129.2, 127.1, 122.4, 120.2, 55.7, 18.3;

HRMS (ESI+) $(C_{15}H_{14}ClN_4)^+$ m/z (calculated) 285.0902, (found) 285.0899, mass difference 1.0 ppm;

LCMS retention time (ESI+, 7 min method, H_2O:MeCN 0.1% formic acid): 3.37 min, m/z 285.05;

(ATIR): ṽ (cm^{-1}) 3125, 3010, 2960, 2923, 1575, 1482, 1451, 1428, 1349, 1223, 1094, 1048, 1015, 973, 832, 804, 790, 736, 705, 645, 551, 503, 482, 411.

2.5 Synthesis of Target Metal Complexes

$Re(CO)_3$(M1Y1) (lab book ref. DRH-052)

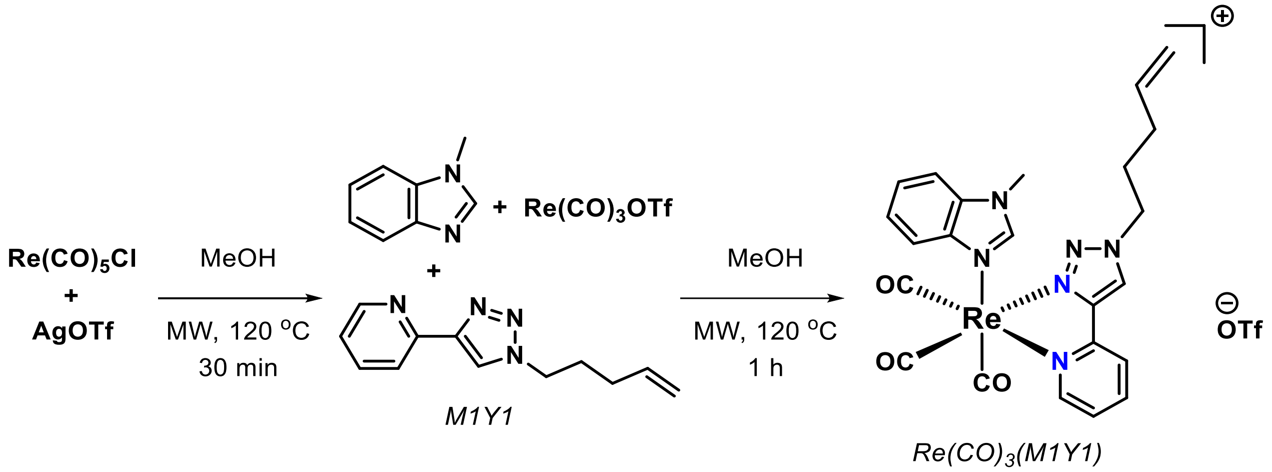

$Re(CO)_5Cl$ (50 mg, 0.138 mmol, 1 equiv.) and AgOTf (35.5 mg, 0.138 mmol, 1 equiv., Note: hydroscopic, light-sensitive) were charged to a 5 mL microwave vial, followed by MeOH (2 mL). The vial was sealed, and heated in a microwave reactor at 120 °C for 30 min. The colorless solution was filtered through celite (with an additional 1 mL of MeOH) into a second 5 mL microwave vial containing M1Y1 (33 mg, 0.152 mmol, 1.1 equiv.) and 1-methylbenzimidazole (BeMeIm, 20 mg, 0.152 mmol, 1.1 equiv.). The vial was sealed and heated in a microwave reactor at 120 °C for 1 h. The solvent was removed to give the crude compound as a pale yellow solid, which was purified by crystallization (approx. 2 mL DCM, layered with approx. 15 mL n-hexane, and stored at 5°C overnight). The mother liquor was decanted, and the resulting crystals were washed with hexane (3 x 2 mL), to give the product. Single crystals suitable for X-ray diffraction were also obtained as part of the purification.

Cream solid, 64 mg, (60% yield);

^{1}H NMR (600 MHz, C*D*Cl_3) δ 9.03 (s, 1H), 8.94 (ddd, *J* = 5.6, 1.5, 0.9 Hz, 1H), 8.40 (dt, *J* = 8.0, 1.1 Hz, 1H), 8.13 (td, *J* = 7.9, 1.5 Hz, 1H), 8.06 (dt, *J* = 8.3, 1.0 Hz, 1H), 7.51 – 7.37 (m, 4H), 7.30 (s, 1H), 5.78 (ddt, *J* = 16.4, 10.2, 6.2 Hz, 1H), 5.11 – 5.02 (m, 2H), 4.62 – 4.52 (m, 2H), 3.72 (s, 3H), 2.22 – 2.10 (m, 4H);

^{13}C NMR (151 MHz, C*D*Cl_3) δ 196.6, 194.3, 191.3, 152.9, 150.0, 149.1, 144.4, 142.2, 141.0, 136.0, 134.0, 127.6, 126.6, 125.3, 124.9, 124.8, 119.5, 116.9, 111.1, 52.3, 32.0, 30.4, 28.7;

HRMS (ESI+) $(C_{23}H_{22}N_6O_3Re)^+$ m/z (calculated) 617.1305, (found) 617.1286, mass difference 3.9 ppm;

LCMS retention time (ESI+, 7 min method, H_2O:MeCN 0.1% formic acid): 2.93 min, m/z 617.24;

(ATIR): ṽ (cm^{-1}) 3106 (C-H aromatic), 2029 (s, C≡O), 1908 (vs, C≡O), 1530, 1456, 1259, 1153, 1030, 925, 789, 747, 636, 515.

Re(CO)$_3$(M20Y3) (lab book ref. DRH-053)

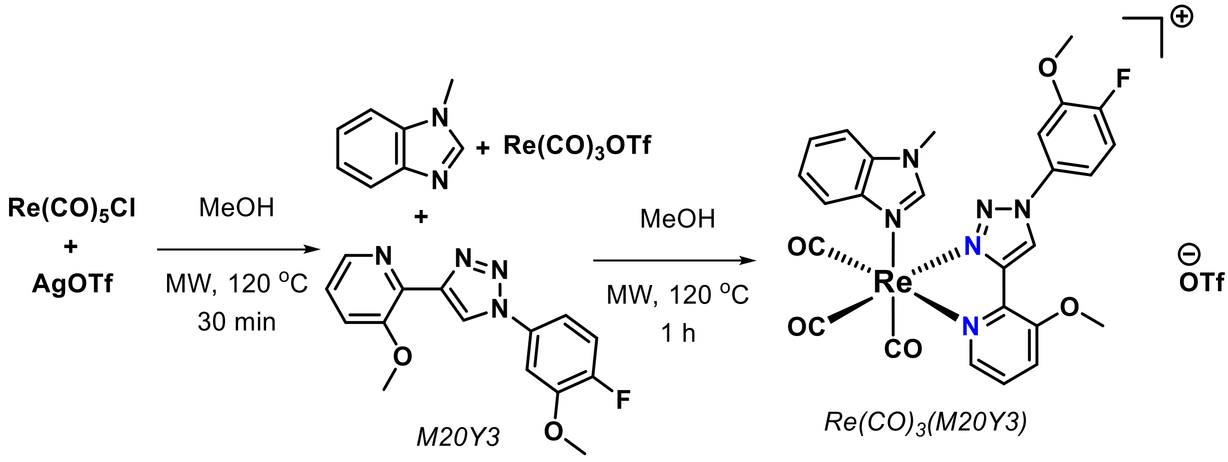

Re(CO)$_5$Cl (25 mg, 0.069 mmol, 1 equiv.) and AgOTf (18 mg, 0.069 mmol, 1 equiv., Note: hydroscopic, light-sensitive) were charged to a 5 mL microwave vial, followed by MeOH (2 mL). The vial was sealed, and heated in a microwave reactor at 120 °C for 30 min. The colorless solution was filtered through celite (with an additional 1 mL of MeOH) into a second 5 mL microwave vial containing M20Y3 (22 mg, 0.073 mmol, 1.1 equiv.) and 1-methylbenzimidazole (BeMeIm, 10 mg, 0.073 mmol, 1.1 equiv.). The vial was sealed and heated in a microwave reactor at 120 °C for 1 h. The solvent was removed to give the crude compound as a pale yellow solid, which was purified by flash column chromatography (Combiflash, 4 g SiO$_2$ cartridge, 0 – 5% MeOH in DCM. Sample loaded 'wet' in DCM (approx. 2 mL)) to obtain the pure compound.

Cream solid, 34 mg, (58% yield), R_f = 0.11 – 0.21 (concentration dependent, streaking);

^{1}H NMR (700 MHz, C*D*Cl$_3$) δ 9.29 (d, *J* = 1.4 Hz, 1H), 8.52 (dt, *J* = 5.4, 1.2 Hz, 1H), 8.12 (dd, *J* = 8.3, 1.3 Hz, 1H), 7.85 (ddd, *J* = 7.2, 2.7, 1.3 Hz, 1H), 7.71 (dt, *J* = 8.8, 1.3 Hz, 1H), 7.55 – 7.51 (m, 1H), 7.49 (dddt, *J* = 8.6, 4.0, 2.8, 1.3 Hz, 2H), 7.42 (ddd, *J* = 8.6, 7.1, 1.3 Hz, 1H), 7.37 (dt, *J* = 8.1, 1.1 Hz, 1H), 7.34 (d, *J* = 1.2 Hz, 1H), 7.28 – 7.24 (m, 3H, Note: residual CHCl$_3$ peak overlaps, leading to higher than expected integration. Expected 1H), 4.28 (d, *J* = 1.3 Hz, 3H), 4.04 (d, *J* = 1.3 Hz, 3H), 3.71 (d, *J* = 1.3 Hz, 3H);

^{13}C NMR (176 MHz, C*D*Cl$_3$) δ 196.7, 194.6, 191.5, 155.2, 154.1, 152.7, 149.2 (d, *J* = 11.5 Hz), 147.3, 144.6, 144.5, 141.2 (d, *J* = 399.4 Hz), 134.0, 132.4 (d, *J* = 3.4 Hz), 128.1, 127.7, 125.1, 124.7, 122.4, 119.7, 117.0 (d, *J* = 20.3 Hz), 114.3 (d, *J* = 7.4 Hz), 111.0, 108.3 (d, *J* = 2.5 Hz), 57.4, 57.1, 31.9;

^{19}F NMR (659 MHz, C*D*Cl$_3$) δ -78.56 (triflate), -131.04 (ddd, *J* = 10.8, 7.2, 3.7 Hz);

HRMS (ESI+) $(C_{26}H_{21}FN_6O_5Re)^+$ m/z (calculated) 703.1109, (found) 703.1101, mass difference 2.2 ppm;

LCMS retention time (ESI+, 7 min method, H_2O:MeCN 0.1% formic acid): 3.08 min, m/z 703.25;

(ATIR): ṽ (cm^{-1}) 3109 (C-H aromatic), 2029 (s, C≡O), 1901 (vs, C≡O), 1571, 1517, 1461, 1478, 1445, 1249, 1139, 1029, 746, 636, 517.

IrCN dimer (lab book ref. DRH-055)

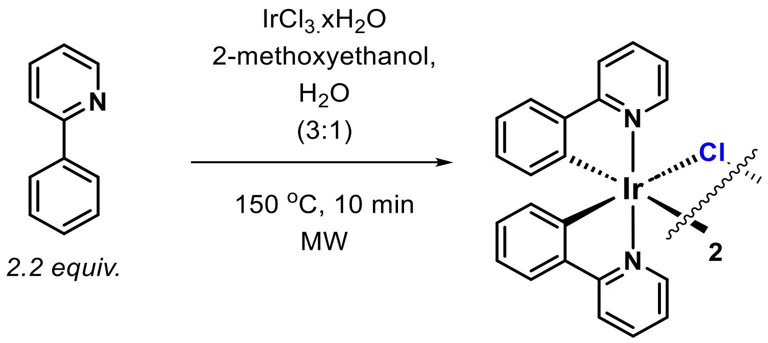

IrCN dimer

[iridium(III)(μ_2-chloro)(2-phenylpyridine)$_2$]$_2$ (IrCN dimer) was synthesized by a literature method developed by Kench *et al.*[3] The ^{1}H NMR data was consistent with the literature. ^{1}H NMR (700 MHz, DMSO-d_6) δ 9.81 (dt, *J* = 5.8, 1.1 Hz, 2H), 9.60 – 9.52 (m, 2H), 8.26 (dd, *J* = 8.0, 1.4 Hz, 2H), 8.18 (dt, *J* = 8.2, 1.2 Hz, 2H), 8.09 (td, *J* = 7.8, 1.6 Hz, 2H), 8.01 (td, *J* = 7.6, 1.5 Hz, 2H), 7.79 (dd, *J* = 7.9, 1.4 Hz, 2H), 7.73 (dd, *J* = 7.8, 1.4 Hz, 2H), 7.57 (ddd, *J* = 7.4, 5.8, 1.4 Hz, 2H), 7.45 (ddd, *J* = 7.4, 5.8, 1.5 Hz, 2H), 6.89 (td, *J* = 7.4, 1.2 Hz, 2H), 6.84 (td, *J* = 7.4, 1.2 Hz, 2H), 6.76 (td, *J* = 7.4, 1.4 Hz, 2H), 6.69 (td, *J* = 7.4, 1.3 Hz, 2H), 6.25 (dd, *J* = 7.7, 1.3 Hz, 2H), 5.66 (dd, *J* = 7.7, 1.2 Hz, 2H).

IrCN(M12Y1) (lab book ref. DRH-056)

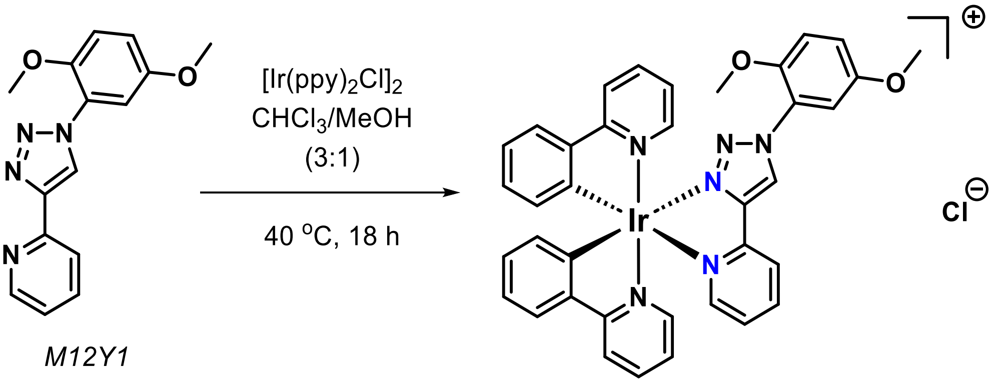

IrCN(M12Y1)

To a 100 mL round bottom flask fitted with an air condenser, IrCN dimer (31 mg, 0.029 mmol, 1 equiv.) and M12Y1 (16 mg, 0.058 mmol, 2 equiv.) were added. $CHCl_3$ (30 mL) and MeOH (10 mL) were added, and the resulting solution was stirred rapidly at 40 °C for 18 h. The solvent was removed *in vacuo*, and the residue was purified by flash column chromatography (Combiflash, 4 g SiO_2 cartridge, 0 – 10%

MeOH in DCM, with compound eluting at 10% MeOH. Sample loaded 'wet' in DCM (approx. 2 mL)) to obtain the pure compound.

Bright yellow solid, 37 mg, (78% yield), R_f = 0.13 (6% MeOH in DCM);

^{1}H NMR (700 MHz, C*D*Cl$_3$) δ 10.95 (s, 1H), 9.80 (dt, *J* = 8.1, 1.1 Hz, 1H), 8.10 (td, *J* = 7.8, 1.6 Hz, 1H), 7.93 – 7.88 (m, 2H), 7.79 – 7.72 (m, 4H), 7.67 (dd, *J* = 7.9, 1.3 Hz, 1H), 7.62 (dd, *J* = 7.8, 1.3 Hz, 1H), 7.52 (ddd, *J* = 5.8, 1.6, 0.7 Hz, 1H), 7.22 (ddd, *J* = 7.6, 5.5, 1.3 Hz, 1H), 7.15 (d, *J* = 3.0 Hz, 1H), 7.06 – 7.01 (m, 2H), 7.01 – 6.89 (m, 5H), 6.84 (td, *J* = 7.5, 1.4 Hz, 1H), 6.32 (ddd, *J* = 7.4, 5.9, 1.2 Hz, 2H), 3.99 (s, 3H), 3.74 (s, 3H);

^{13}C NMR (176 MHz, C*D*Cl$_3$) δ 168.6, 167.8, 153.4, 150.4, 150.3, 149.5, 149.4, 148.9, 148.6, 146.7, 146.1, 143.8, 143.7, 140.2, 138.0, 137.9, 132.0, 131.8, 130.8, 130.8, 130.2, 126.3, 126.0, 125.6, 124.8, 124.4, 123.3, 122.9, 122.7, 122.3, 119.5, 119.5, 116.1, 114.0, 111.5, 57.3, 56.2;

HRMS (ESI+) $(C_{37}H_{30}IrN_6O_2)^+$ m/z (calculated) 783.2054, (found) 783.2057, mass difference 0.3 ppm;

LCMS retention time (ESI+, 7 min method, H_2O:MeCN 0.1% formic acid): 3.02 min, m/z 783.39;

(ATIR): ṽ (cm^{-1}) 3038 (C-H aromatic), 1606, 1582, 1512, 1476, 1437, 1418, 1289, 1209, 1031, 871, 756, 729, 630.

IrCN(M8Y4) (lab book ref. DRH-057)

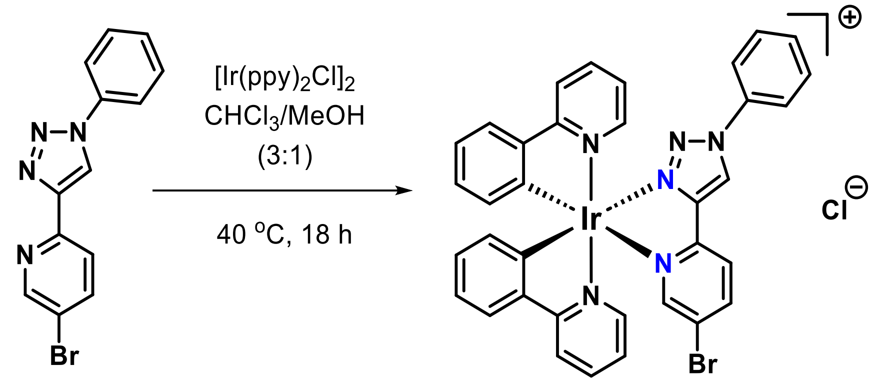

M8Y4 *IrCN(M8Y4)*

To a 100 mL round bottom flask fitted with an air condenser, IrCN dimer (40 mg, 0.037 mmol, 1 equiv.) and M8Y4 (22 mg, 0.074 mmol, 2 equiv.) were added. $CHCl_3$ (30 mL) and MeOH (10 mL) were added, and the resulting solution was stirred rapidly at 40 °C for 18 h. The solvent was removed *in vacuo*, and the residue was purified by flash column chromatography (Combiflash, 4 g SiO_2 cartridge, 0 – 10% MeOH in DCM. Sample loaded 'wet' in DCM (approx. 2 mL)) to obtain the pure compound.

Bright yellow solid, 49 mg, (79% yield), R_f = 0.18 (6% MeOH in DCM);

^{1}H NMR (700 MHz, $CDCl_3$) δ 11.95 – 11.89 (m, 1H), 9.52 (d, *J* = 8.5 Hz, 1H), 8.14 (dt, *J* = 8.5, 1.8 Hz, 1H), 8.08 – 8.03 (m, 2H), 7.96 – 7.90 (m, 2H), 7.79 (dtt, *J* = 18.8, 8.0, 1.6 Hz, 2H), 7.75 – 7.73 (m, 2H), 7.69 (dt, *J* = 7.9, 1.5 Hz, 1H), 7.65 (dt, *J* = 7.9, 1.5 Hz, 1H), 7.52 (dd, *J* = 5.8, 1.8 Hz, 1H), 7.51 – 7.46 (m, 2H), 7.41 (td, *J* = 7.3, 1.3 Hz, 1H), 7.05 (qd, *J* = 6.8, 2.9 Hz, 2H), 7.03 – 6.98 (m, 2H), 6.94 (tt, *J* = 7.5, 1.4 Hz, 1H), 6.88 (tt, *J* = 7.4, 1.4 Hz, 1H), 6.30 (ddt, *J* = 17.6, 7.7, 1.4 Hz, 2H);

^{13}C NMR (176 MHz, $CDCl_3$) δ 168.4, 167.8, 150.5, 149.5, 149.3, 149.0, 148.8, 148.5, 145.5, 143.7, 143.6, 142.8, 138.3, 138.2, 136.2, 131.9, 131.8, 131.1, 130.2, 130.1, 130.0, 127.7, 126.7, 125.0, 124.5, 123.5, 123.2, 123.1, 122.5, 122.3, 120.4, 119.8, 119.7;

HRMS (ESI+) $(C_{35}H_{30}BrIrN_6)^+$ m/z (calculated) 801.0948, (found) 801.0944, mass difference 1.7 ppm;

LCMS retention time (ESI+, 7 min method, H_2O:MeCN 0.1% formic acid): 3.08 min, m/z 801.39;

(ATIR): ṽ (cm^{-1}) 3043 (C-H aromatic), 1606, 1582, 1478, 1268, 1127, 1096, 1063, 1030, 1008, 755, 729, 675.

$Mn(CO)_3$(M19Y1) (lab book ref. DRH-068-1)

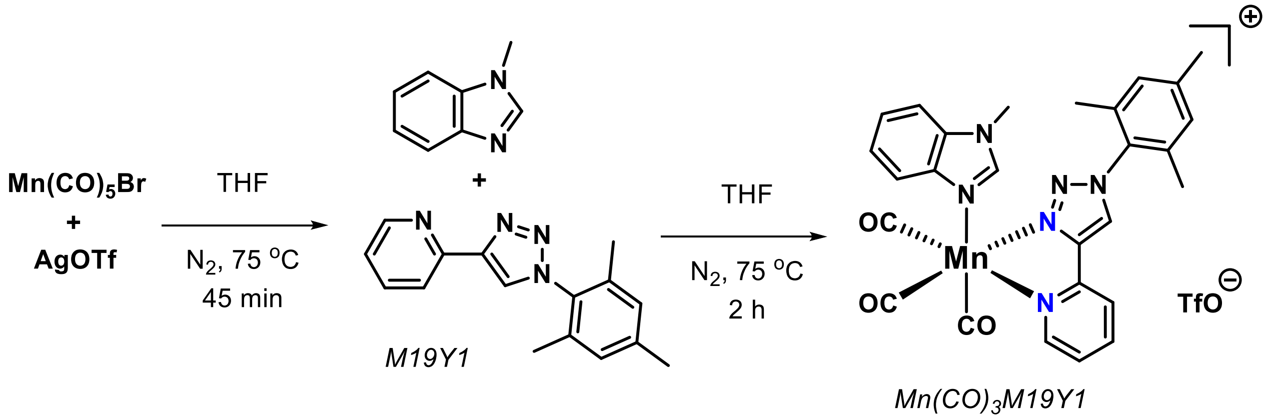

For this procedure, all reaction vessels and containers were covered in aluminium foil and handled in the dark to prevent degradation by light. $Mn(CO)_5Br$ (91 mg, 0.33 mmol, 1 equiv.) and AgOTf (85 mg, 0.33 mmol, 1 equiv., Note: hydroscopic) were charged to a 10 mL microwave vial, which was evacuated and backfilled with N_2. Dry THF (4.5 mL) was added, and the vial was sealed under N_2 and heated at 75 °C for 45 mins, after which it was allowed to cool. The pressure was released by piercing the seal with a N_2 balloon when cool, and 2 mL of the yellow solution (0.15 mmol Mn complex) was decanted into a second 10 mL microwave vial containing M19Y1 (41 mg, 0.155 mmol, 1.05 equiv.) and 1-methylbenzimidazole (BeMeIm, 21 mg, 0.155 mmol, 1.05 equiv.) under N_2. The vial was sealed and heated at 75 °C for 2 h. The solvent was removed *in vacuo* to give the crude compound as a yellow solid, which was purified by flash column chromatography (Combiflash, 4 g SiO_2 cartridge, 0 – 5% MeOH in DCM. Sample loaded 'wet' in DCM (approx. 2 mL)) to obtain the pure compound.

Note – this compound degraded on the TLC plate, so only the major compound R_f is given.

Yellow solid, 86 mg, (84% yield), R_f = 0.37 (5% MeOH in DCM);

^{1}H NMR (700 MHz, CD_2Cl_2) δ 9.33 (dt, *J* = 5.5, 1.2 Hz, 1H), 8.83 (s, 1H), 8.30 (dt, *J* = 7.8, 1.1 Hz, 1H), 8.14 – 8.08 (m, 2H), 7.72 – 7.67 (m, 1H), 7.60 (s, 1H), 7.45 – 7.42 (m, 1H), 7.42 – 7.34 (m, 2H), 7.09 (s, 2H), 3.74 (s, 3H), 2.40 (s, 3H), 1.92 (s, 6H);

^{13}C NMR (176 MHz, CD_2Cl_2) δ 220.7, 220.1, 217.7, 154.2, 149.8, 148.6, 145.5, 142.5, 142.2, 140.7, 135.2, 135.0, 132.7, 129.8, 127.8, 127.0, 124.9, 124.3, 124.3, 118.7, 111.5, 32.1, 21.3, 17.3;

HRMS (ESI+) $(C_{27}H_{24}MnN_6O_3)^+$ m/z (calculated) 535.1291, (found) 535.1285, mass difference 1.2 ppm;

LCMS retention time (ESI+, 7 min method, H_2O:MeCN 0.1% formic acid): 3.34 min, m/z 535.273;

(ATIR): ṽ (cm^{-1}) 3109, 2038 (s, C≡O), 1930 (vs, C≡O), 1616, 1529, 1464, 1264 (s), 1162, 1030, 747, 637, 516, 427.

$Mn(CO)_3$(M22Y4) (lab book ref. DRH-068-2)

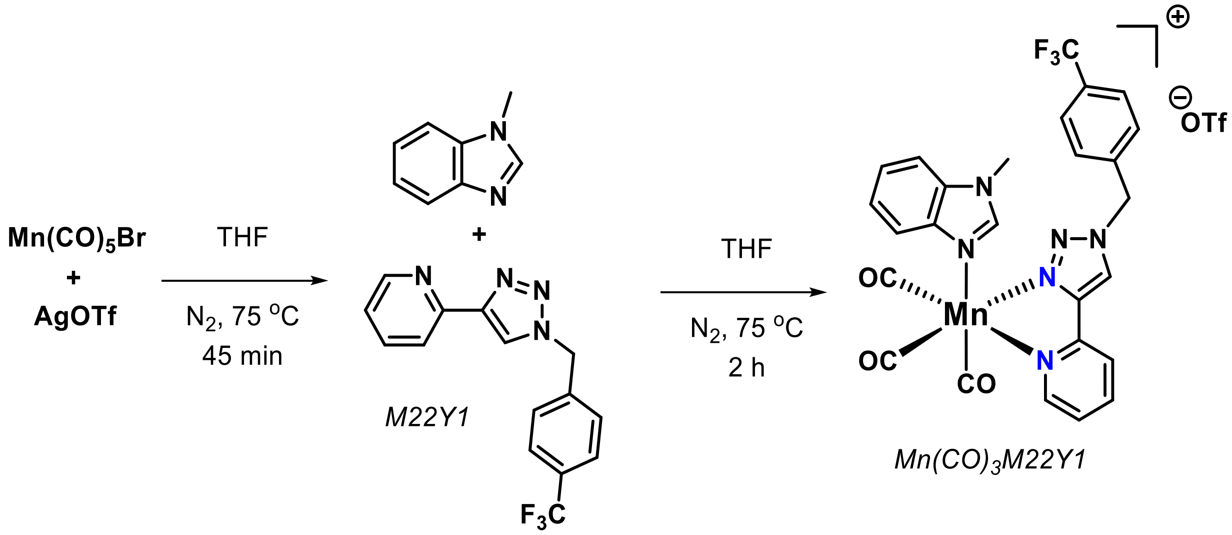

For this procedure, all reaction vessels and containers were covered in aluminium foil and handled in the dark to prevent degradation by light. $Mn(CO)_5Br$ (91 mg, 0.33 mmol, 1 equiv.) and AgOTf (85 mg, 0.33 mmol, 1 equiv., Note: hydroscopic) were charged to a 10 mL microwave vial, which was evacuated and backfilled with N_2. Dry THF (4.5 mL) was added, and the vial was sealed under N_2 and heated at 75 °C for 45 mins, after which it was allowed to cool. The pressure was released by piercing the seal with a N_2 balloon when cool, and 2 mL of the yellow solution (0.15 mmol Mn complex) was decanted into a second 10 mL microwave vial containing M22Y1 (47 mg, 0.155 mmol, 1.05 equiv.) and 1-methylbenzimidazole (BeMeIm, 21 mg, 0.155 mmol, 1.05 equiv.) under N_2. The vial was sealed and heated at 75 °C for 2 h. The solvent was removed *in vacuo* to give the crude compound as a yellow oil, which was purified by flash column chromatography (Combiflash, 4 g SiO_2 cartridge, 0 – 5% MeOH in DCM. Sample loaded 'wet' in DCM (approx. 2 mL)) to obtain the pure compound.

Note – this compound degraded on the TLC plate, so only the major compound R_f is given.

Yellow solid, 83 mg, (76% yield), R_f = 0.33 (5% MeOH in DCM);

^{1}H NMR (700 MHz, CD_2Cl_2) 9.26 (d, *J* = 5.5 Hz, 1H), 9.13 (s, 1H), 8.09 (d, *J* = 7.9 Hz, 1H), 8.03 (td, *J* = 7.8, 1.5 Hz, 1H), 7.96 (d, *J* = 8.4 Hz, 1H), 7.67 – 7.58 (m, 5H), 7.38 (d, *J* = 8.1 Hz, 1H), 7.37 – 7.32 (m, 2H), 7.23 (ddd, *J* = 8.4, 7.1, 1.3 Hz, 1H), 5.90 – 5.82 (m, 2H), 3.66 (s, 3H);

^{13}C NMR (176 MHz, CD_2Cl_2) δ 220.7, 220.2, 217.5, 154.0, 149.9, 148.5, 145.2, 142.4, 140.7, 137.9, 134.9, 131.4 (q, *J* = 32.4 Hz), 129.7, 126.6, 126.5 (q, *J* = 3.6 Hz), 126.4, 124.8, 124.3 (q, *J* = 272.6 Hz) 124.2, 123.7, 118.4, 111.4, 55.7, 32.2;

^{19}F NMR (659 MHz, CD_2Cl_2) δ -63.09 (s, 3F), -78.95 (s, 3F);

HRMS (ESI+) $(C_{26}H_{19}F_3MnN_6O_3)^+$ m/z (calculated) 575.0858, (found) 575.0846, mass difference 2.1 ppm;

LCMS retention time (ESI+, 7 min method, H_2O:MeCN 0.1% formic acid): 3.19 min, m/z 575.158;

(ATIR): ṽ (cm^{-1}) 3109, 2038 (s, C≡O), 1927 (vs, C≡O), 1621, 1528, 1458, 1324, 1252, 1157, 1123, 1029, 781, 746, 635, 517, 427.

IrCp*(P1A7) (lab book ref. DRH-088-2)

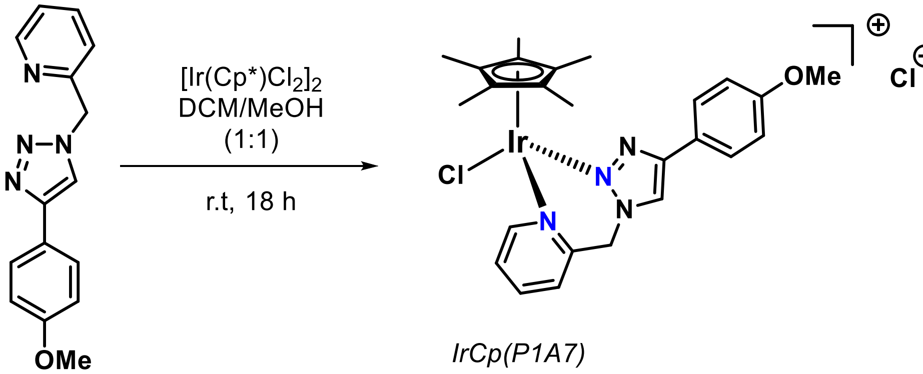

P1A7

IrCp(P1A7)

To a 7 mL glass vial, di-µ-chloro-bis[chloro(pentamethylcyclopentadienyl)iridium(III)] (40 mg, 0.05 mmol, 1 equiv.) and P1A7 (26.7 mg, 0.10 mmol, 2 equiv.) were added. DCM (2 mL) and MeOH (2 mL0 were added, and the reaction mixture stirred overnight at room temperature. The mixture was transferred to a 20 mL vial and the solvent was evaporated *in vacuo*. The residue was dissolved in DCM (2 mL), to which Et_2O (10 mL) was slowly added to precipitate the complex. The fine precipitate was isolated on a glass sinter, and washed with Et_2O (3 x 20 mL). The precipitate was dissolved in DCM, collected and the solvent removed in vacuo. Et_2O (2 mL) was added to the residue and dried *in vacuo*, yielding the target complex as a yellow solid.

Yellow solid, 62 mg, (94% yield);

^1H NMR (700 MHz, CD_2Cl_2) (Note: in several different NMR solvents (MeCN-d_3, DMSO-d_6, CD_2Cl_2, a quantity of free ligand was observed (17% by ^1H NMR), which was not present in LCMS of the same NMR samples. This is likely due to the instability of 6-membered chelates, and proved impossible to remove by further recrystallisation. Only peaks relating to the target complex are reported here) δ 9.43 (s, 1H), 8.80 (dd, *J* = 5.8, 1.6 Hz, 1H), 8.48 – 8.44 (m, 1H), 7.98 (td, *J* = 7.7, 1.6 Hz, 1H), 7.84 – 7.80 (m, 2H), 7.76 – 7.69 (m, 1H), 7.49 (ddd, *J* = 7.4, 5.7, 1.5 Hz, 1H), 7.00 – 6.93 (m, 2H), 4.99 (d, *J* = 15.7 Hz, 1H), 3.83 (s, 3H), 1.65 (s, 15H).

^{13}C NMR (176 MHz, CD_2Cl_2) δ 160.90, 155.71, 152.83, 150.68, 141.17, 128.39, 127.84, 127.14, 126.85, 121.79, 114.65, 114.55, 89.61, 55.73, 9.29.

HRMS (ESI+) $(C_{25}H_{29}ClIrN_4O)^+$ m/z (calculated) 629.1654, (found) 629.1660, mass difference 2.2 ppm;

LCMS retention time (ESI+, 7 min method, H_2O:MeCN 0.1% formic acid): $[M–Cl, +MeCN]^{2+}$ 1.55 min, m/z 317.63, $[M]^+$ 2.36 min, m/z 629.23, unknown Ir containing impurity 2.94 min, m/z 705.40;

(ATIR): ṽ (cm^{-1}) 3369 (br), 1515, 1495, 1464, 1423, 1382, 1304, 1249, 1178, 1027, 973, 838, 768, 600, 534, 457.

IrCp*(P4Y11) (lab book ref. DRH-088-3)

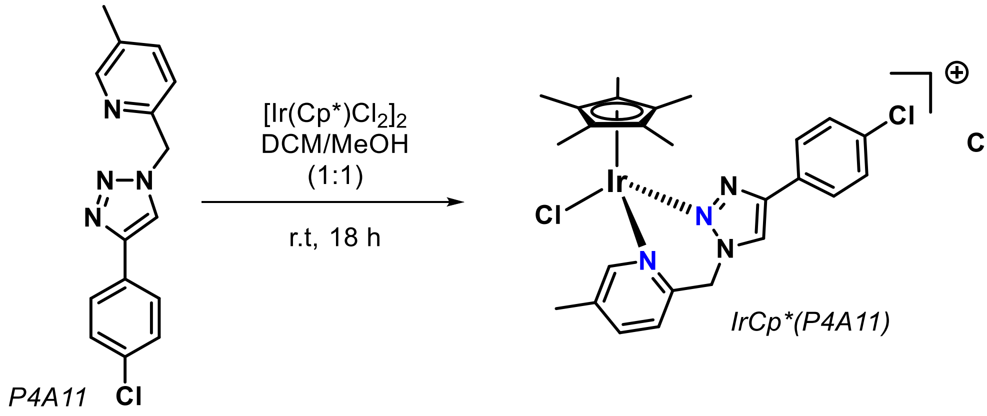

To a 7 mL glass vial, di-μ-chloro-bis[chloro(pentamethylcyclopentadienyl)iridium(III)] (40 mg, 0.05 mmol, 1 equiv.) and P4A11 (28.6 mg, 0.10 mmol, 2 equiv.) were added. DCM (2 mL) and MeOH (2 mL0 were added, and the reaction mixture stirred overnight at room temperature. The mixture was transferred to a 20 mL vial and the solvent was evaporated *in vacuo*. The residue was dissolved in DCM (2 mL), to which Et_2O (10 mL) was slowly added to precipitate the complex. The fine precipitate was isolated on a glass sinter, and washed with Et_2O (3 x 20 mL). The precipitate was dissolved in DCM, collected and the solvent removed in vacuo. Et_2O (2 mL) was added to the residue and dried *in vacuo*, yielding the target complex as a yellow solid.

Yellow solid, 60 mg, (88% yield);

^{1}H NMR (700 MHz, CD_2Cl_2) (Note: in several different NMR solvents (MeCN-d_3, DMSO-d_6, CD_2Cl_2, a quantity of free ligand was observed (19% by ^{1}H NMR), which was not present in LCMS of the same NMR samples. This is likely due to the instability of 6-membered chelates, and proved impossible to remove by further recrystallisation. Only peaks relating to the target complex are reported here) δ 9.68 (s, 1H), 8.58 (d, *J* = 2.0 Hz, 1H), 8.30 (d, *J* = 7.9 Hz, 1H), 7.89 – 7.84 (m, 2H), 7.81 – 7.74 (m, 2H), 7.46 – 7.41 (m, 2H), 4.94 (d, *J* = 15.7 Hz, 1H), 2.41 (s, 3H), 1.65 (s, 15H).

^{13}C NMR (176 MHz, CD_2Cl_2) δ 155.59, 149.85, 149.63, 141.71, 137.84, 135.26, 129.48, 127.88, 127.83, 127.69, 127.27, 89.58, 54.92, 18.43, 9.30.

HRMS (ESI+) $(C_{25}H_{28}Cl_2IrN_4)^+$ m/z (calculated) 647.1315, (found) 547.1312, mass difference 1.7 ppm;

LCMS retention time (ESI+, 7 min method, H_2O:MeCN 0.1% formic acid): [M–Cl, +MeCN]$^{2+}$ 1.77 min, m/z 326.55, $[M]^+$ 2.63 min, m/z 647.23;

(ATIR): ṽ (cm^{-1}) 3381 (br), 2982, 2922, 1609, 1484, 1461, 1383, 1307, 1093, 1030, 1014, 973, 838, 798, 706, 563, 502, 446.

2.6 Catalytic Testing

Method for transfer hydrogenation of Coumarin-N_3 by crude RuCy libraries

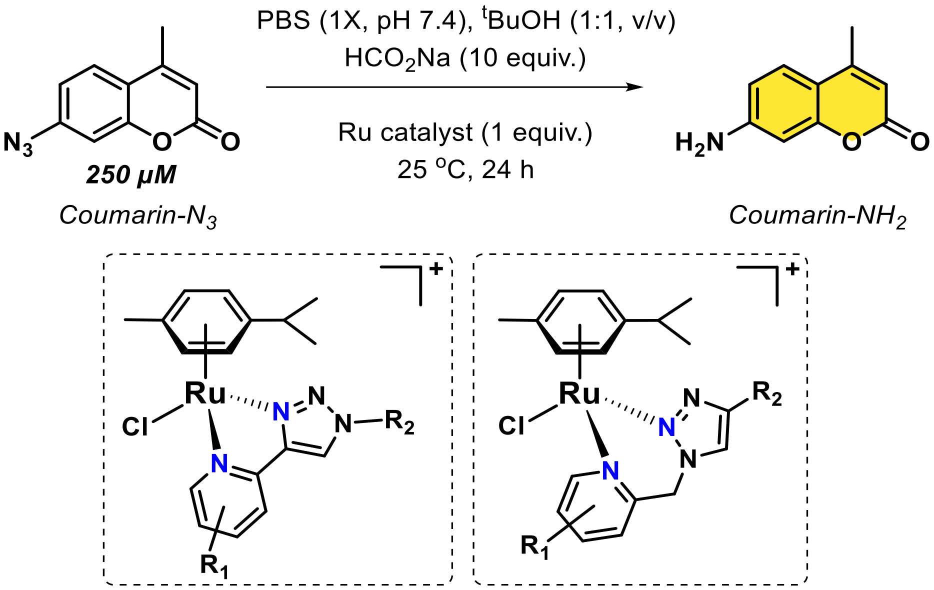

Method adapted from Weng *et al.*[2]

Coumarin-N_3 (10 mg, 0.05 mmol) and sodium formate (34 mg, 0.5 mmol) were dissolved in a mixture of 1:1 tBuOH / 1X PBS solution (25 mL) to give a solution of 2 mM coumarin-N_3. In a polystyrene 96 well plate, 165 uL of 1:1 tBuOH /1X PBS solution was added, followed by coumarin-N_3 / sodium formate mix (25 uL). 10 uL of RuCy catalyst stock solution at 5 mM was added to initiate the reaction. The reaction was monitored by fluorescence (generation of Coumarin-NH_2) using a plate reader (λ_{ex} = 350 nm, λ_{em} = 445 nm) over 21 h at 25 °C, with 2 mins of shaking (400 rpm) and 13 min delay between data points.

Method for transfer hydrogenation of Harmaline by crude IrCp* libraries

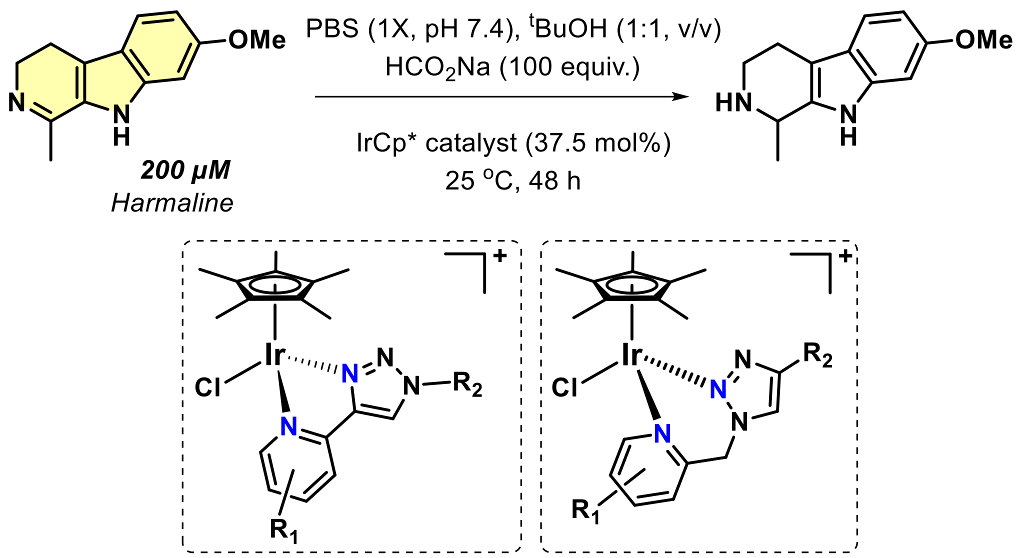

Method adapted from Miller *et al.*[4]

IrCp* catalyst libraries were diluted to a stock solution of 750 µM (50:50 DMSO/H_2O). Separately, stock solutions of harmaline (4.3 mg, 0.02 mmol, 2 mM) and sodium formate (136 mg, 2 mmol, 200 mM) were dissolved in mixtures of 1:1 tBuOH / 1X PBS solution (10 mL). In a polystyrene 96 well plate, 135 uL of 1:1 tBuOH /1X PBS solution was added, followed by the harmaline stock solution (20 µL, 1 equiv.), then sodium formate stock (25 µL, 125 equiv.), and finally 20 uL of IrCp* catalyst stock solution at 750 µM was added to initiate the reaction. The reaction was monitored by UV-Vis absorbance (consumption of harmaline) using a plate reader (λ = 374 nm) over 48 h at 25 °C, with 2 mins of shaking (400 rpm) and 3 min delay between data points.

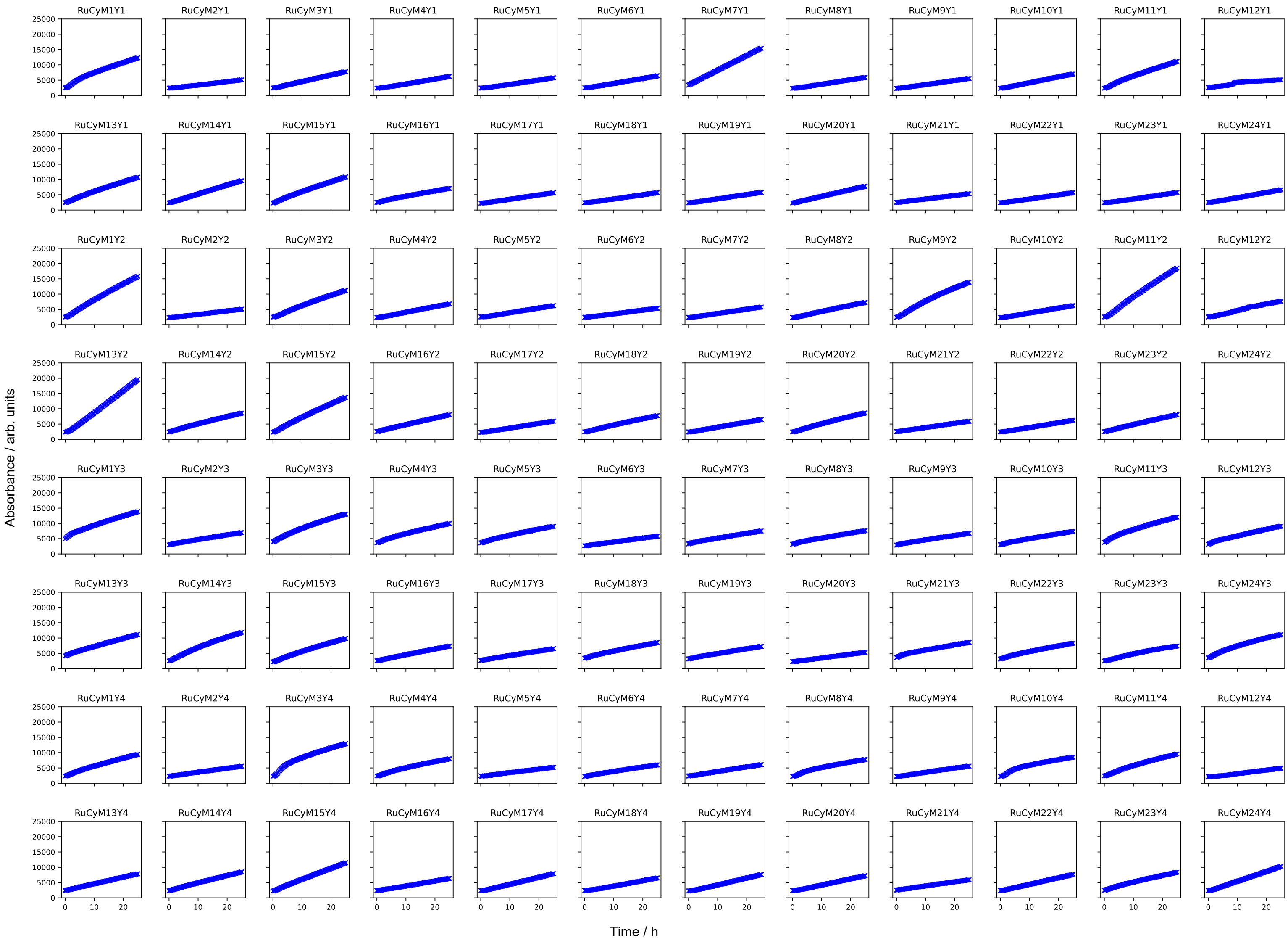

Figure S1: Kinetic traces for the RuCy(Tz-4-P) library for the transfer hydrogenation of Coumarin-N_3. Fluorescence indicates the presence of Coumarin-NH_2.

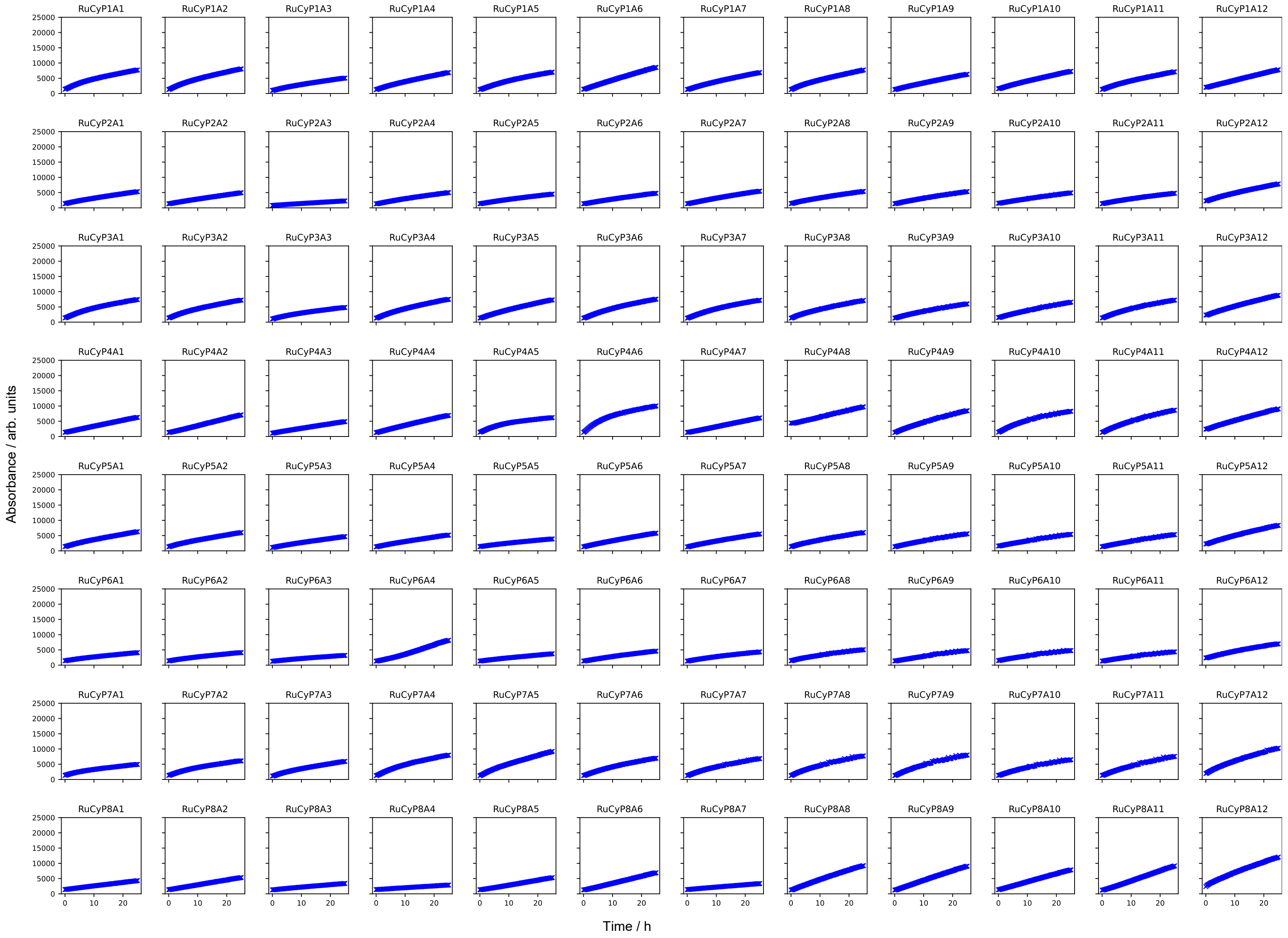

Figure S2: Kinetic traces for the RuCy(Tz-1-MP) library for the transfer hydrogenation of Coumarin-N_3. Fluorescence indicates the presence of Coumarin-NH_2.

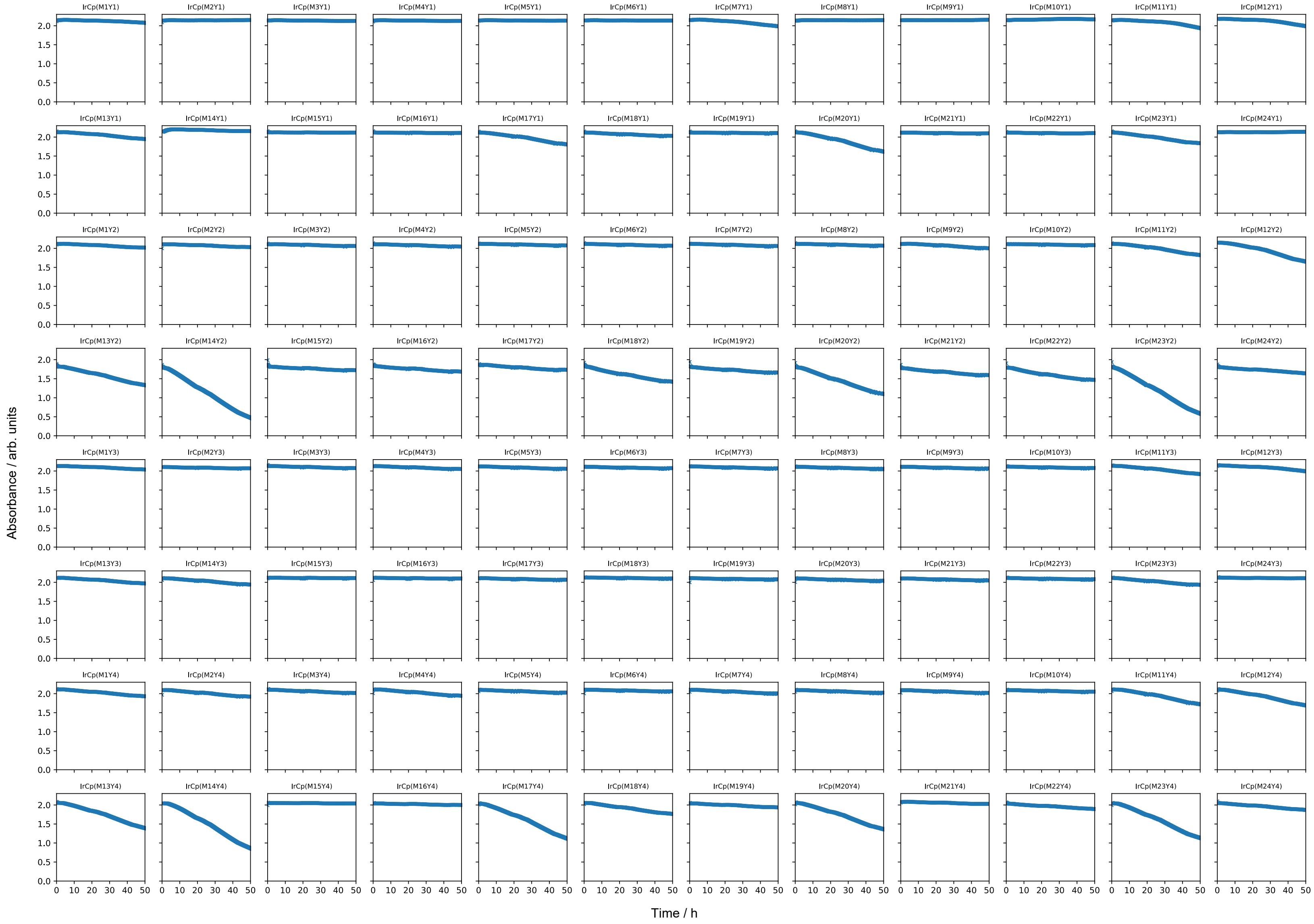

Figure S3: Kinetic traces for the IrCp*(Tz-4-P) library for the transfer hydrogenation of harmaline. Absorbance (374 nm) decrease indicates consumption of starting material.

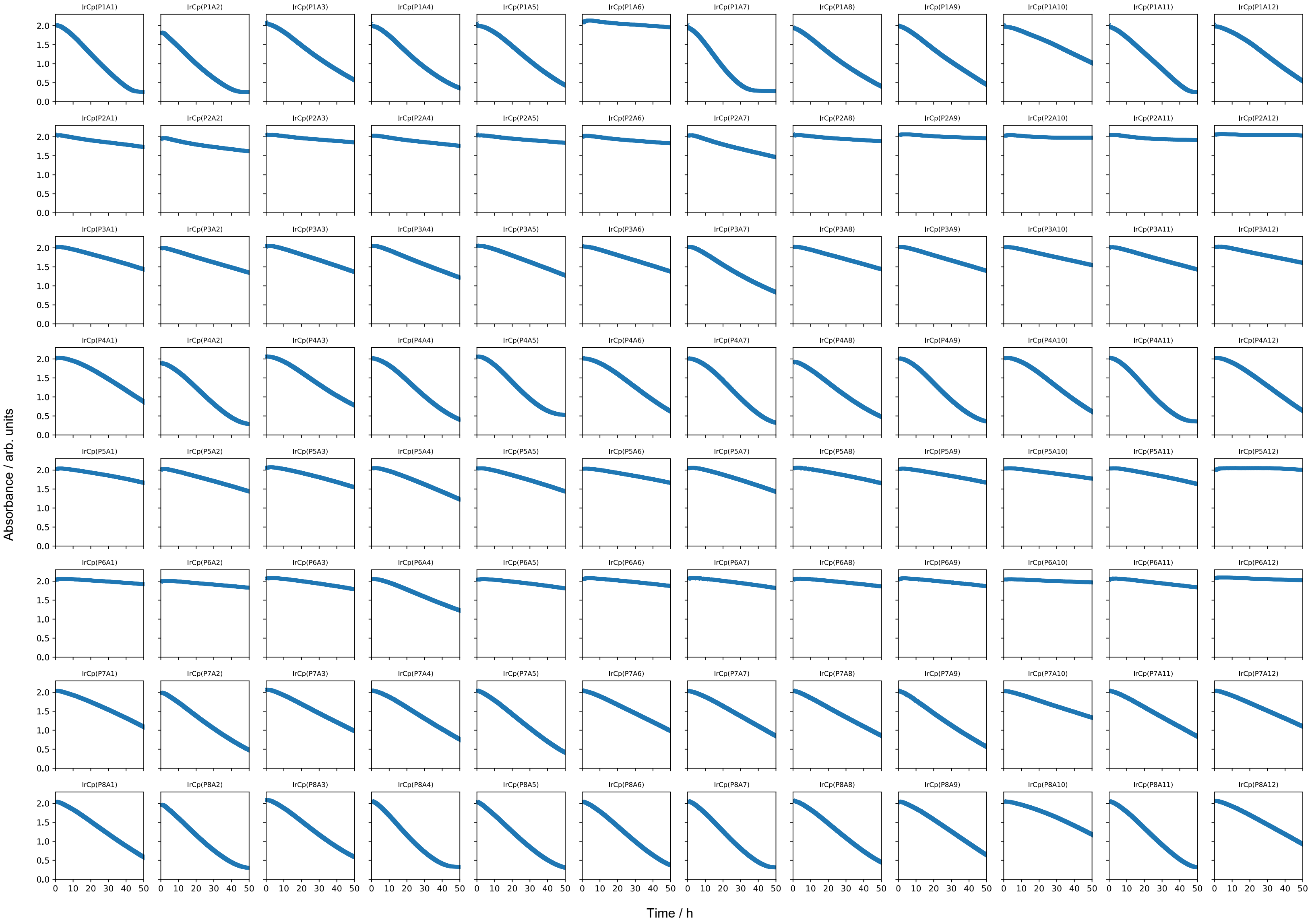

Figure S4: Kinetic traces for the IrCp*(Tz-1-MP) library for the transfer hydrogenation of harmaline. Absorbance (374 nm) decrease indicates consumption of starting material.

Transfer hydrogenation of Coumarin-N_3 by pure IrCp* compounds

Coumarin-N_3 (4 mg, 0.02 mmol) and sodium formate (136 mg, 2 mmol) were dissolved in a mixture of 1:1 tBuOH / 1X PBS solution (10 mL) or tBuOH / H_2O solution to give a solution of 2 mM coumarin-N_3.Stock solutions of IrCp*(P1A7) and IrCp*(P4A11) were prepared in 1:1 DMSO / H_2O solution at 5 mM. Preactivation was achieved by mixing a stock of the catalyst in DMSO (10 mM) with an equal quantity of AgOTf in H_2O (20 mM) and heating the resulting 5 mM mixture for 1 h at 60 °C in a 2 mL eppindorf tube. Formation of the Ir^{2+} adduct was confirmed by LCMS. In a polystyrene 96 well plate, 165 uL of 1:1 tBuOH /1X PBS or tBuOH / H_2O solution was added, followed by coumarin-N_3 / sodium formate mix (25 uL). 10 uL of catalyst stock solution at 5 mM was added to initiate the reaction. The reaction was monitored by fluorescence (generation of Coumarin-NH_2) using a plate reader (λ_{ex} = 350 nm, λ_{em} = 445 nm) over 21 h at 25 °C, with 2 mins of shaking (400 rpm) and 13 min delay between data points. Reactions were done in triplicate, and the values averaged.

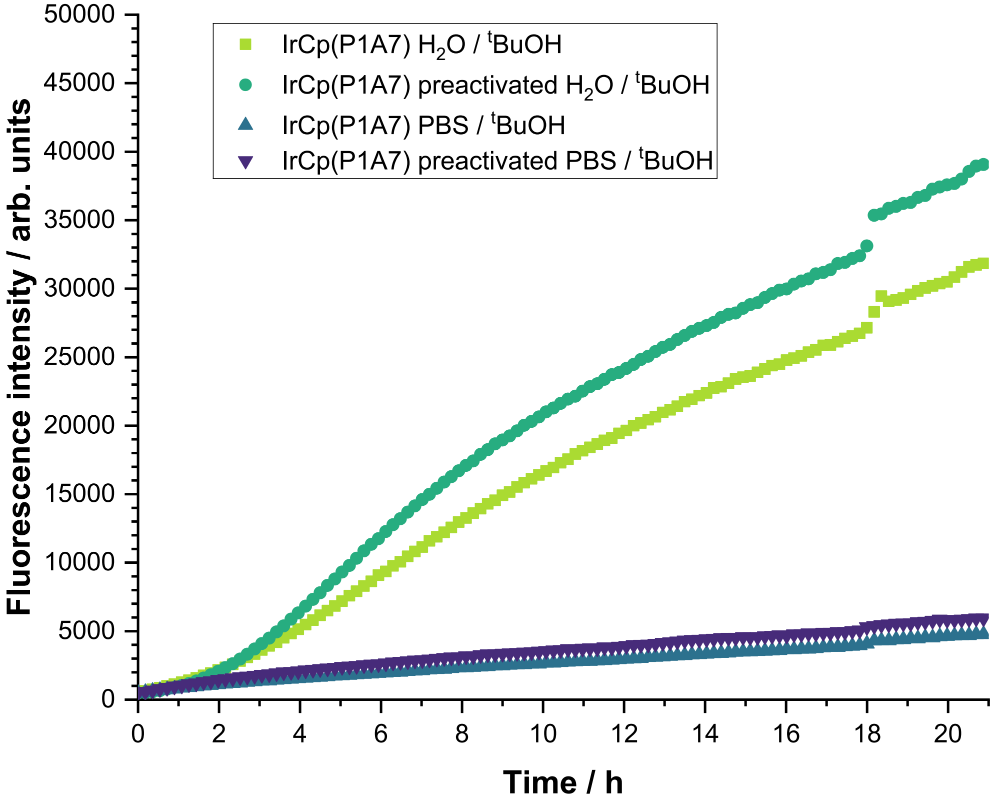

Figure S5: Kinetic trace of Coumarin-N_3 generation by transfer hydrogenation using pure IrCp*(P1A7) under various conditions.

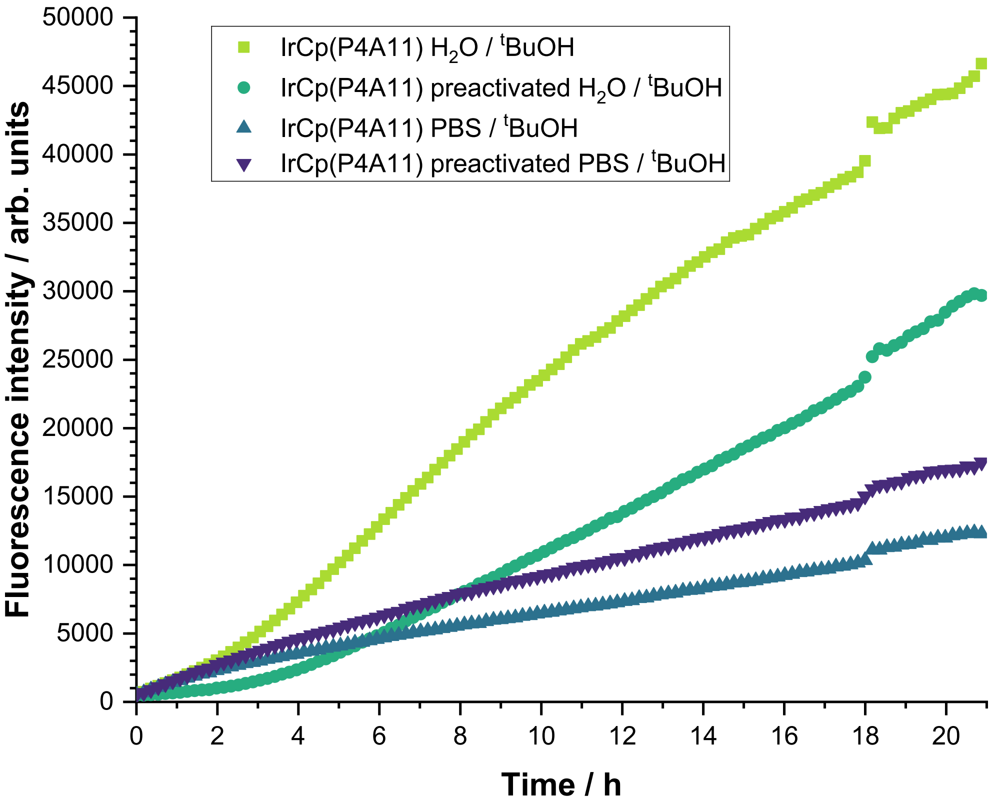

Figure S6: Kinetic trace of Coumarin-N_3 generation by transfer hydrogenation using pure IrCp*(P4A11) under various conditions.

Transfer hydrogenation of Harmaline by pure IrCp* compounds

Harmaline (4.3 mg, 0.02 mmol) and sodium formate (136 mg, 2 mmol) were dissolved in a mixture of 1:1 tBuOH / 1X PBS solution (10 mL) or tBuOH / H_2O solution to give a solution of 2 mM harmaline. Stock solutions of IrCp*(P1A7) and IrCp*(P4A11) were prepared in 1:1 DMSO / H_2O solution at 750 μM. Preactivation was achieved by mixing a stock of the catalyst in DMSO (1.5 mM) with an equal quantity of AgOTf in H_2O (5 mM) and heating the resulting 750 μM mixture for 1 h at 60 °C in a 2 mL eppindorf tube. Formation of the Ir^{2+} adduct was confirmed by LCMS. In a polystyrene 96 well plate, 160 uL of 1:1 tBuOH /1X PBS or tBuOH / H_2O solution was added, followed by harmaline / sodium formate mix (20 uL). 20 uL of catalyst stock solution at 750 μM was added to initiate the reaction. The reaction was monitored by UV-Vis absorbance (consumption of harmaline) using a plate reader (λ = 374 nm) over 48 h at 25 °C, with 2 mins of shaking (400 rpm) and 3 min delay between data points. LCMS of relevant reactions was taken to verify consumption of harmaline and generation of the hydrogenated product. LCMS peak area% conversions were used to calibrate the graphs for %consumption of starting material by 2 point normalisation (maximum absorbance value = 100%, minimum absorbance = conversion by LCMS). When no starting material was

detected by LCMS, conversion was assumed to be quantitative. Reactions were done in triplicate, and the values averaged. No significant conversion to product by IrCp*(P1A7) was detected under any conditions.

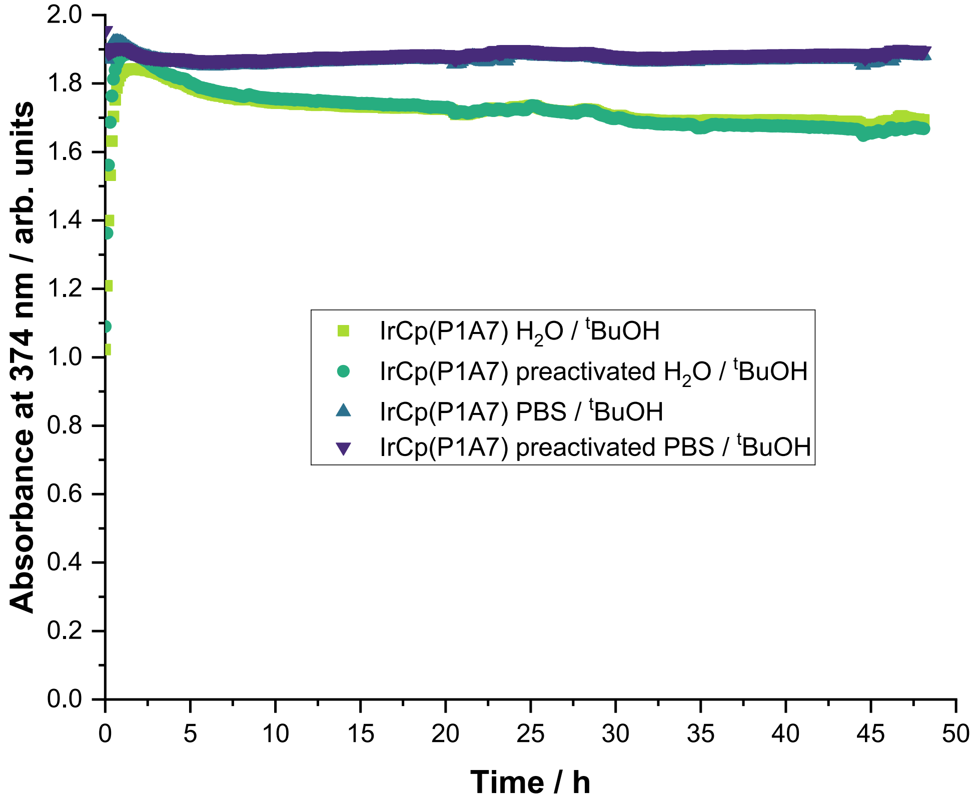

Figure S7: Kinetic trace of Harmaline consumption by pure IrCp*(P1A7) under various conditions.

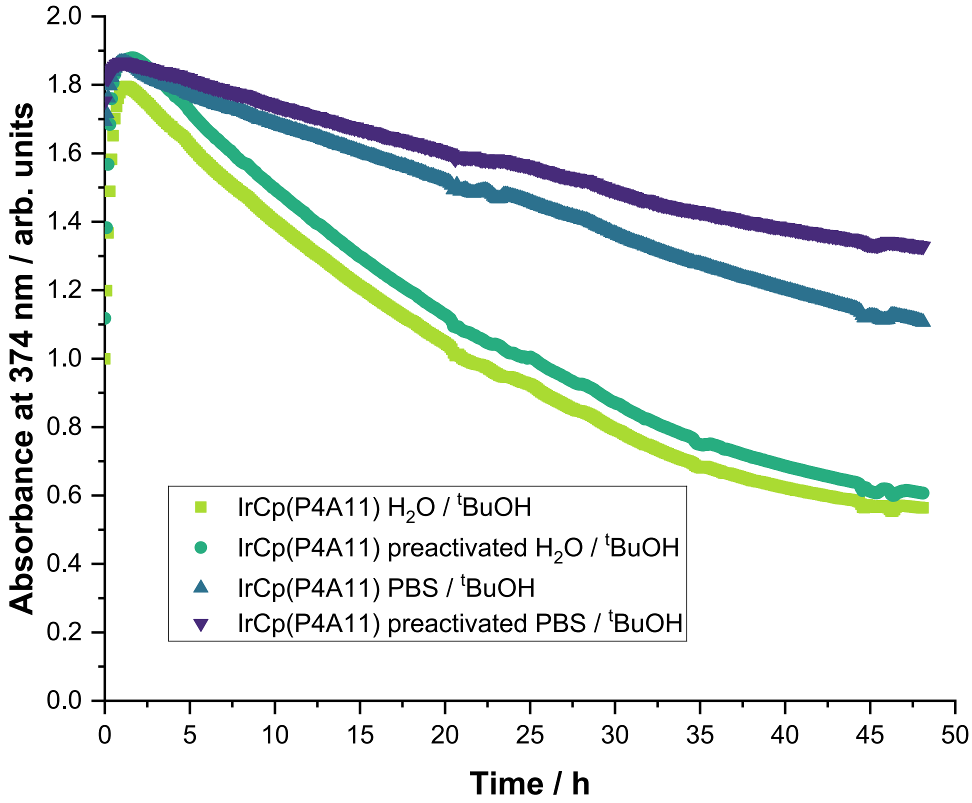

Figure S8: Kinetic trace of Harmaline consumption by transfer hydrogenation using pure IrCp*(P1A7) under various conditions.

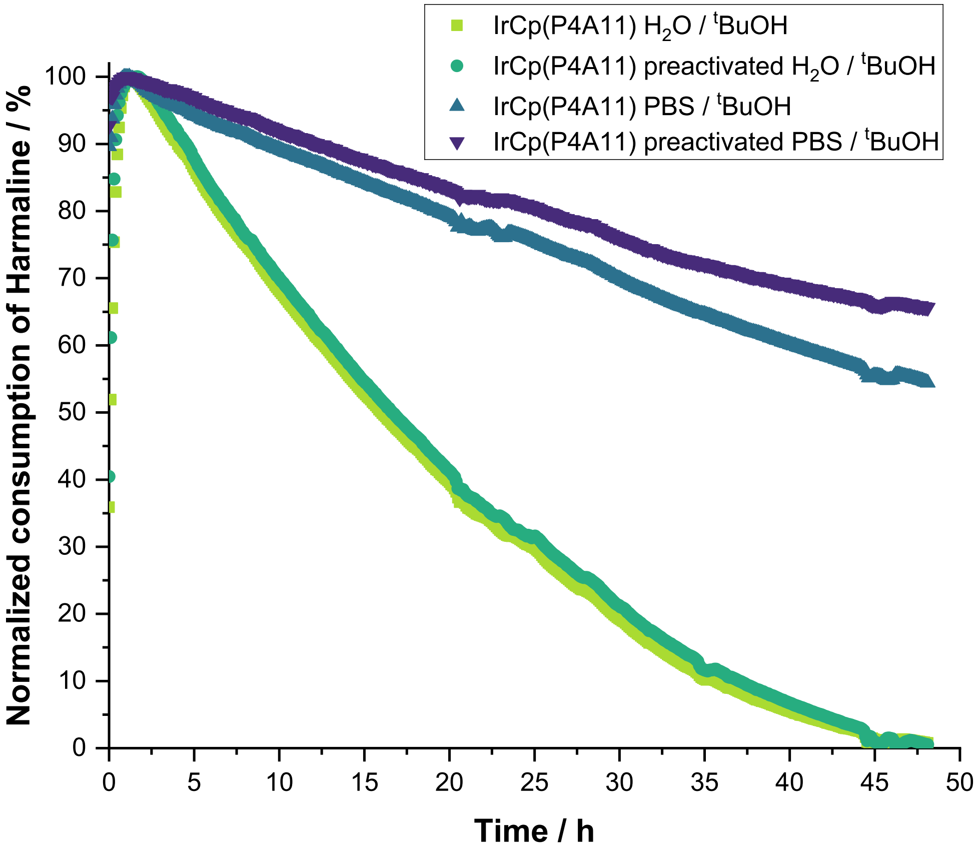

Figure S9: Normalized kinetic trace of Harmaline consumption by transfer hydrogenation using pure IrCp*(P4A11) under various conditions.

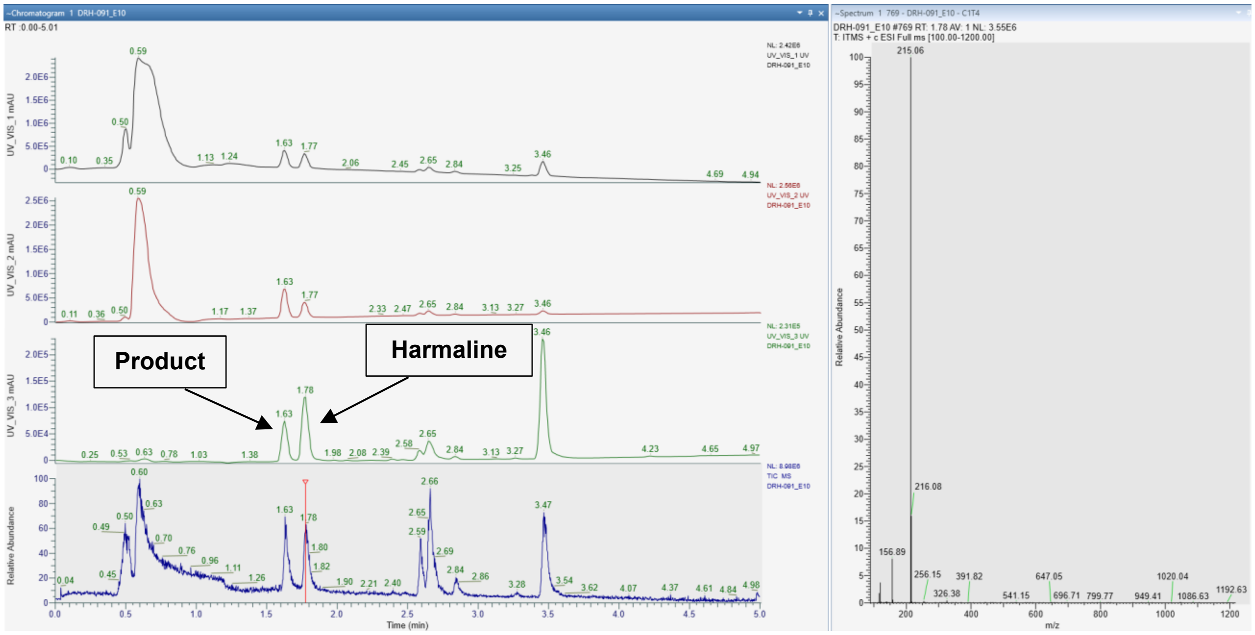

Figure S10: LCMS trace (5 min method) of pure IrCp*(P4A11) catalyzed reaction (preactivated, PBS/tBuOH) after 48 h at 25 °C. [Harmaline + H]$^+$ 1.76 min, m/z 215.06; [Product + H]$^+$ 1.63 min, m/z 217.07 (lab book ref. DRH-091)

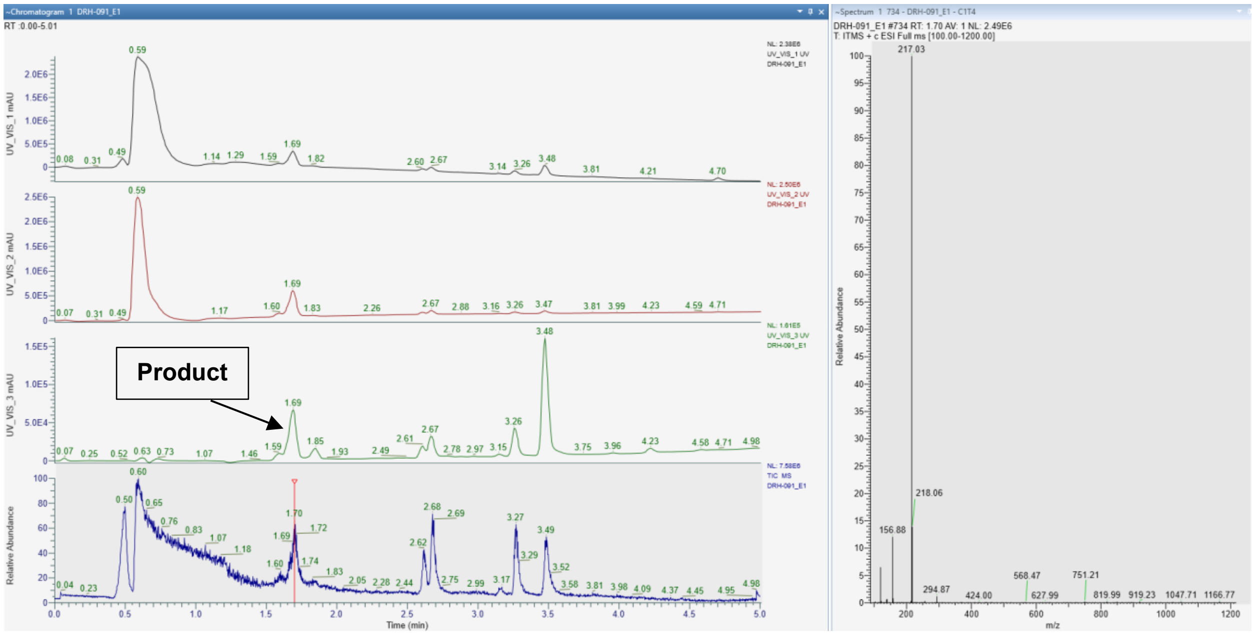

Figure S11: LCMS trace (5 min method) of pure IrCp*(P4A11) catalyzed reaction (H_2O/tBuOH) after 48 h at 25 °C. [Product + H]$^+$ 1.69 min, m/z 217.07. No harmaline mass detected (lab book ref. DRH-091)

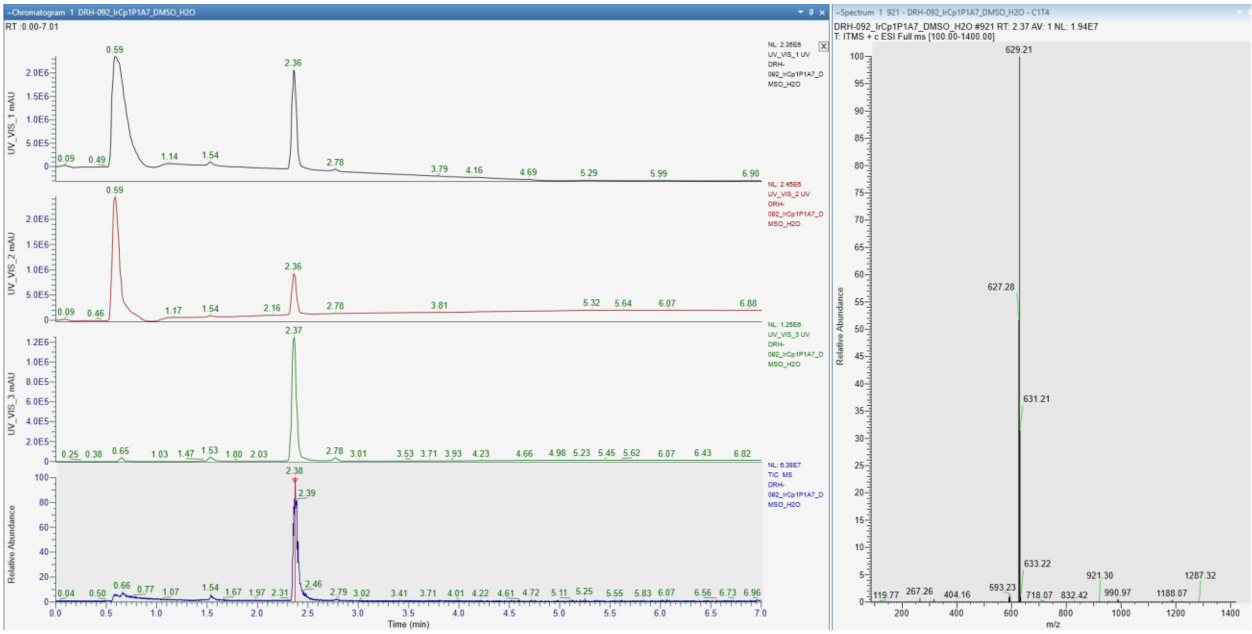

Figure S12: LCMS trace (7 min method) of pure IrCp*(P1A7) 5 mM in 1:1 DMSO:H_2O solution. [M]$^+$ 2.36 min, m/z 629.21 (lab book ref. DRH-092)

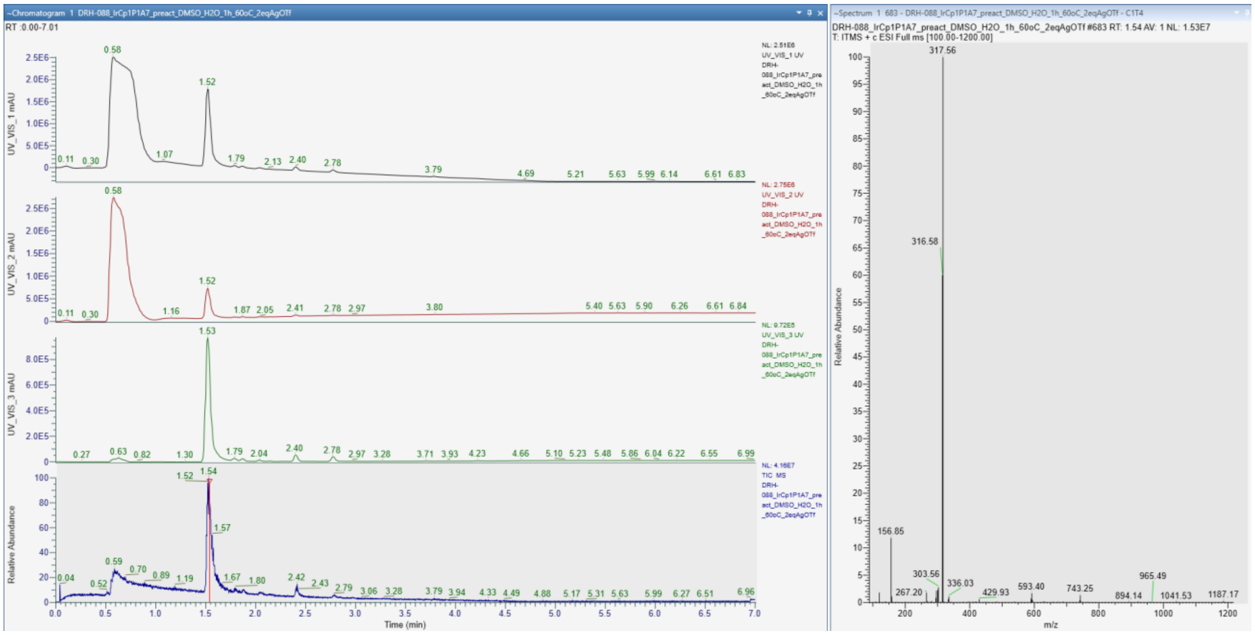

Figure S13: LCMS trace (7 min method) of pure IrCp*(P1A7) 5 mM in 1:1 DMSO:H_2O solution after preactivation with AgOTf (2 equiv., 1 h at 60 °C). $[M–Cl, +MeCN]^{2+}$ 1.53 min, m/z 317.56 (lab book ref. DRH-088)

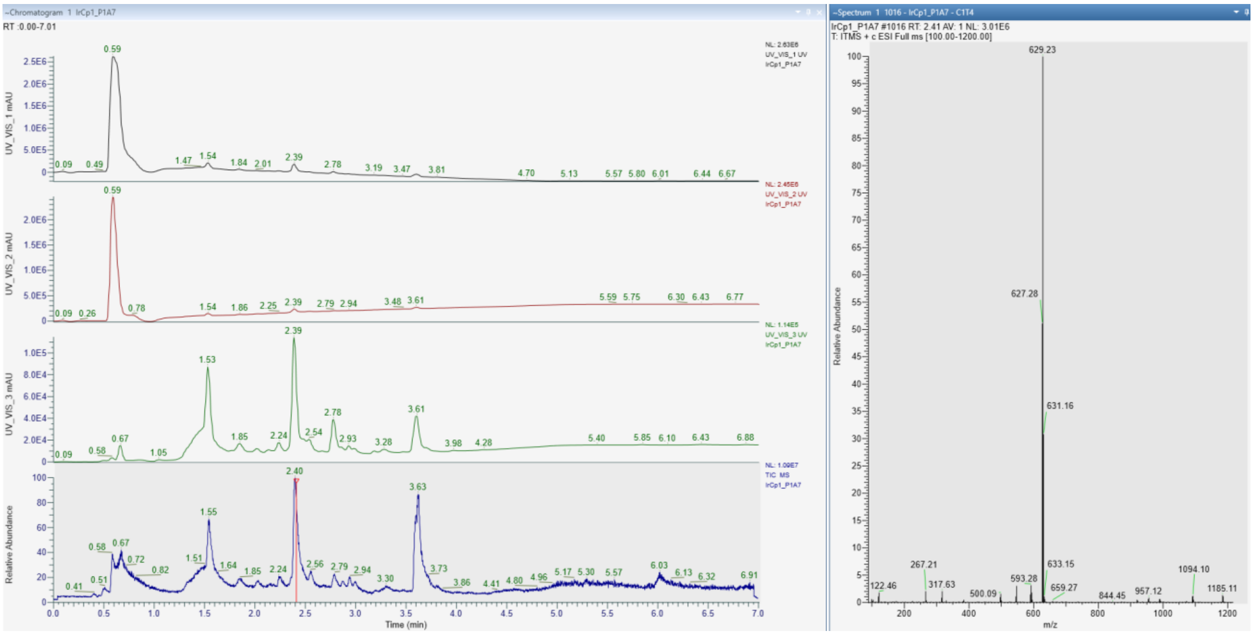

Figure S14: LCMS trace (7 min method) of crude IrCp*(P1A7) solution from combinatorial synthesis. $[M–Cl, +MeCN]^{2+}$ 1.53 min, m/z 317.58; $[M]^{+}$ 2.39 min, m/z 629.23; Unknown Ir dimer species at 3.61 min, m/z 1019.26.

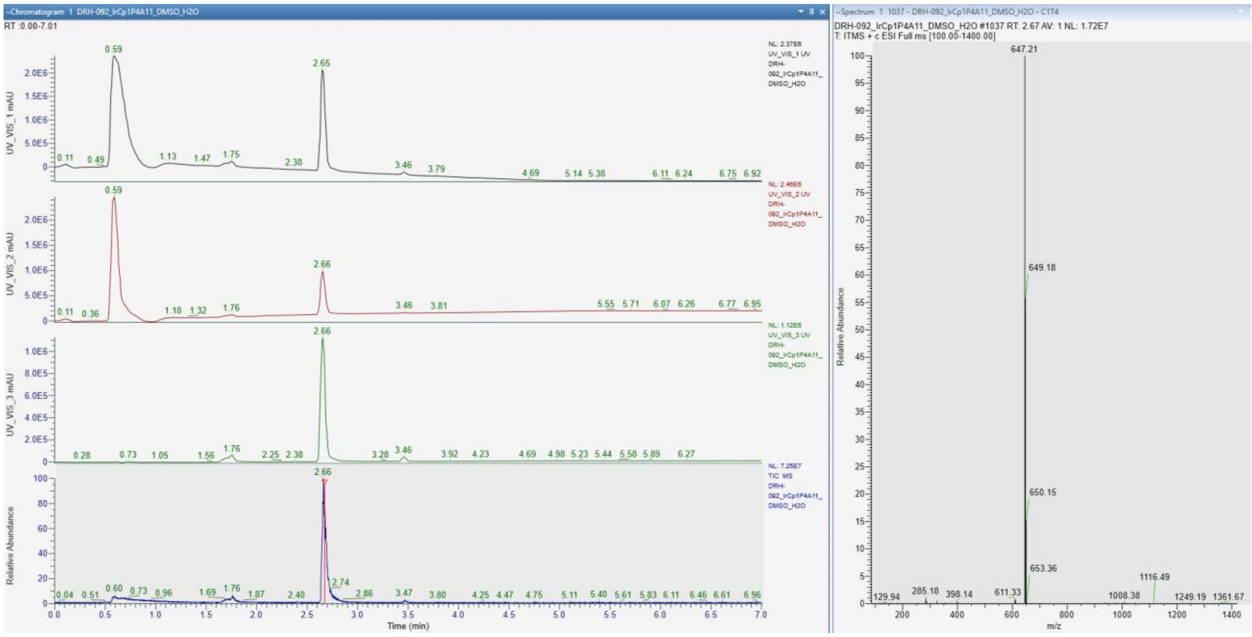

Figure S15: LCMS trace (7 min method) of pure IrCp*(P4A11) 5 mM in 1:1 DMSO:H_2O solution. $[M]^+$ 2.66 min, m/z 647.21; (lab book ref. DRH-092)

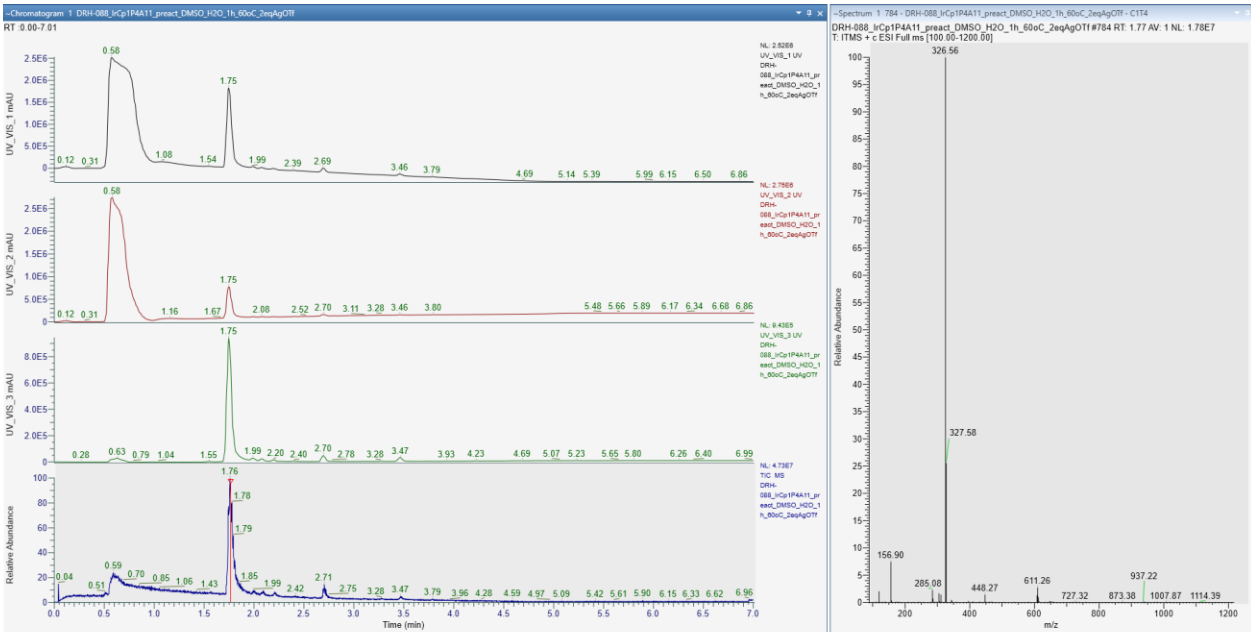

Figure S16: LCMS trace (7 min method) of IrCp*(P4A11) 5 mM in 1:1 DMSO:H_2O solution after preactivation with AgOTf (2 equiv., 1 h at 60 °C). $[M–Cl, +MeCN]^{2+}$ 1.75 min, m/z 326.56 (lab book ref. DRH-088)

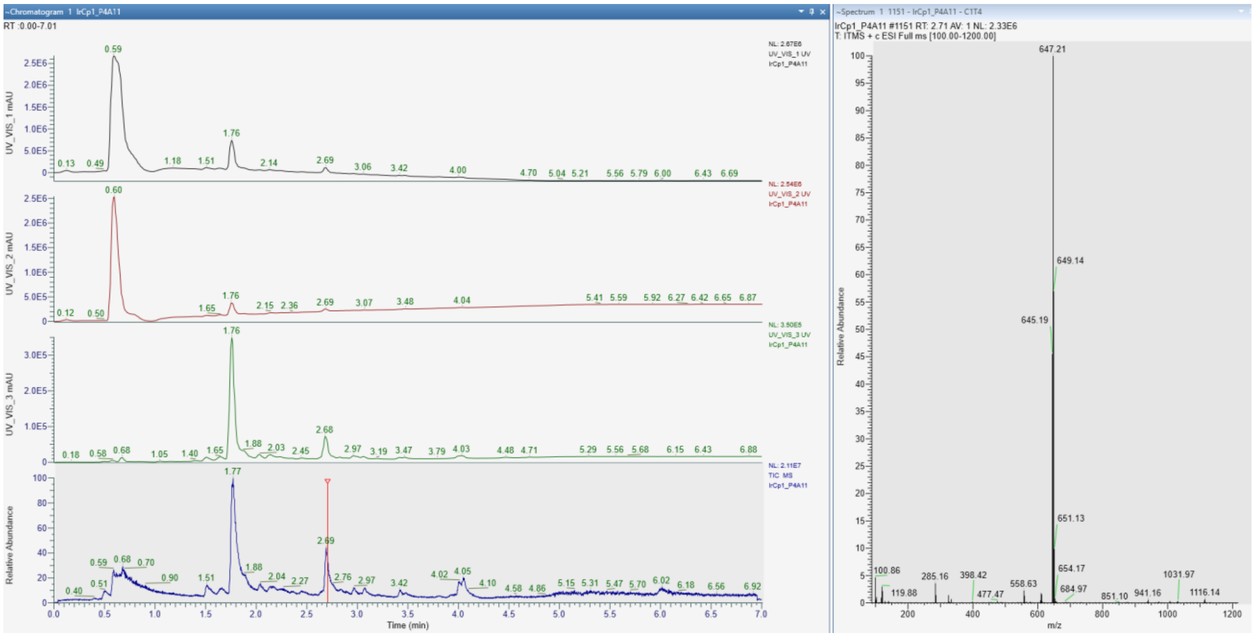

Figure S17: LCMS trace (7 min method) of crude IrCp*(P4A11) solution from combinatorial synthesis. [M–Cl, +MeCN]$^{2+}$ 1.76 min, m/z 326.55; [M]$^{+}$ 2.69 min, m/z 647.20.

2.7 Photophysical Properties

5 µL of each compound in the IrCN library (5 mM) was diluted with 200 µL DMSO. Of this, 50 µL was taken and diluted with a further 150 µL DMSO in a polystyrene 96 well plate (31.25 µM). The UV-Vis absorbance (270 – 600 nm, 25 flashes) and fluorescence (λ_{ex} = 406 nm, λ_{em} = 450 – 700 nm, and λ_{ex} = 380 nm, λ_{em} = 450 – 700 nm, 25 flashes) of these compounds were then measured using a plate reader. There were negligible differences between the emission spectra at the different wavelengths.

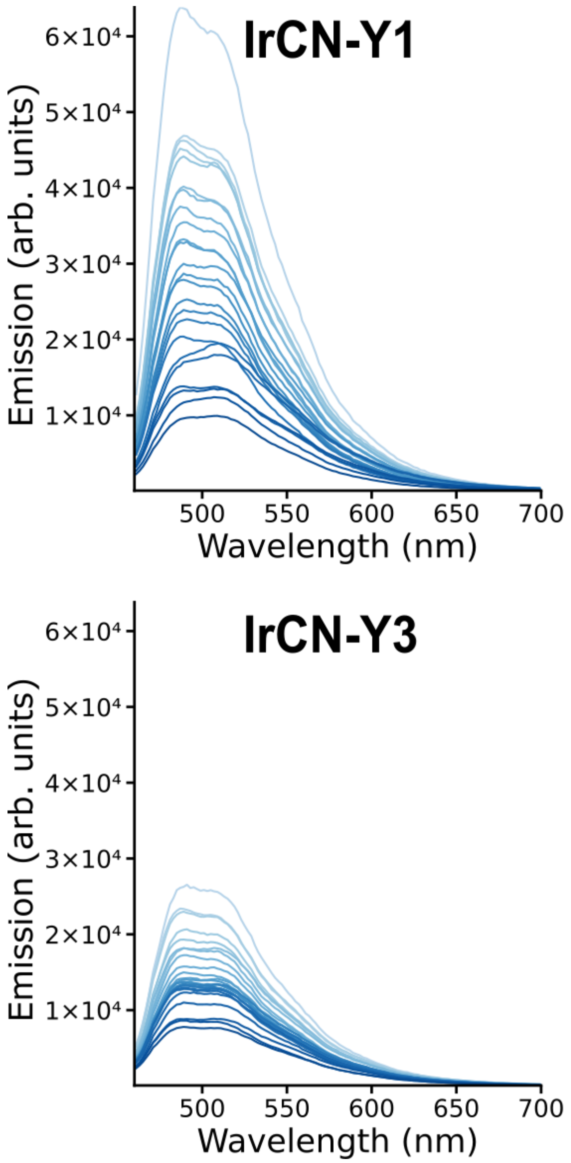

Figure S18: Photophysical emission spectra of the IrCN library (31.25 µM solutions in DMSO, λ_{ex} = 380 nm)

Stock solutions of purified **IrCN(M12Y1)** and **IrCN(M8Y4)** were measured in the same way, and the fluorescence was compared to the crude reaction mixtures.

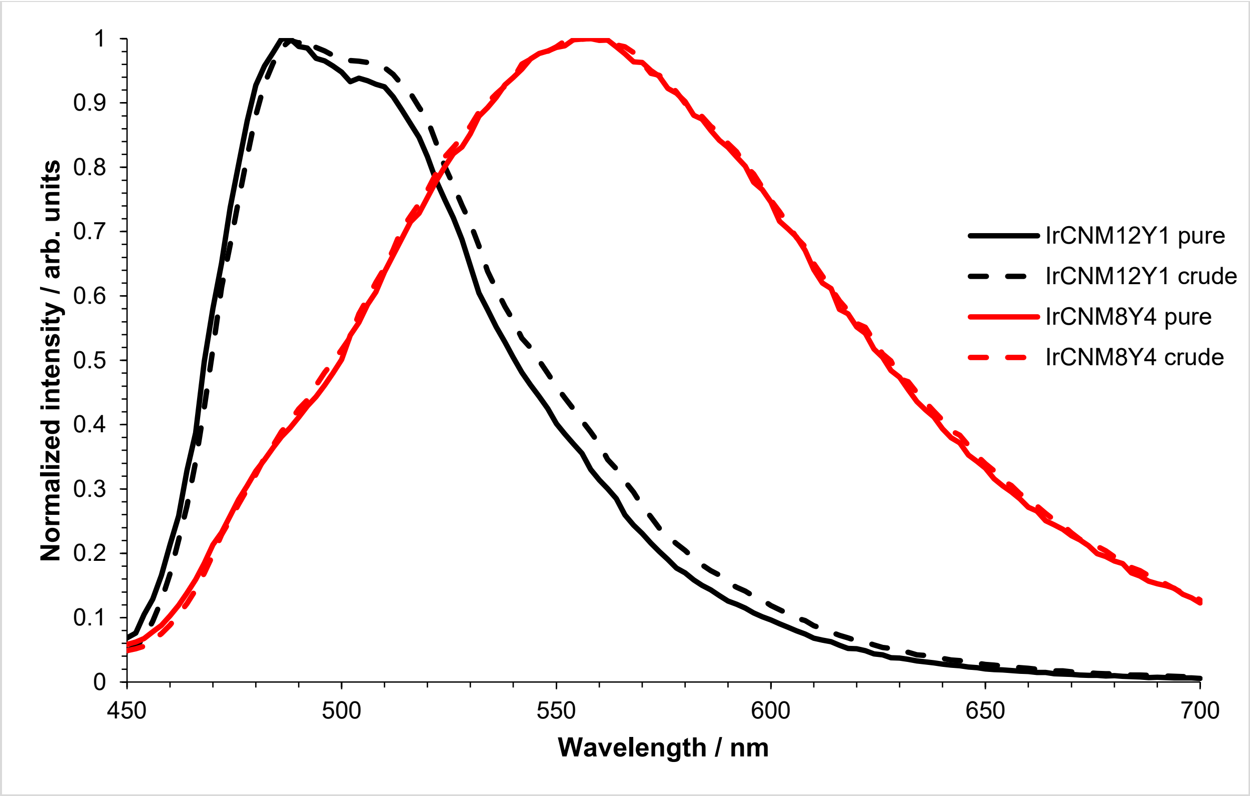

Figure S19: Normalized fluorescence in DMSO (31.3 µM, λ_{ex} = 406 nm) of IrCN(M12Y1) and IrCN(M8Y4) (pure and crude compounds)

Reactive Oxygen Species (ROS) Assays

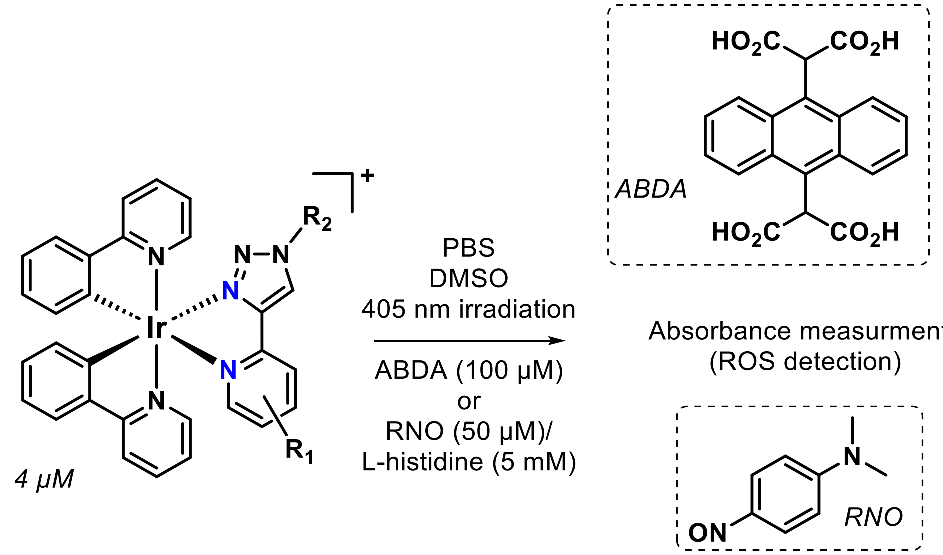

5 μL of IrCN compounds (5 mM in DMSO:H_2O) were diluted with 200 μL of DMSO. 13 μL of this stock was diluted with 187 μL PBS (1X) to generate the IrCN stocks at 4 μM concentration.

Tris(2,2′-bipyridyl)ruthenium(II) chloride hexahydrate (5 mM in DMSO) was used as the control for these reactions, and diluted down to 4 μM in the same manner as the IrCN library.

For ABDA assay:[5]

Anthracenediyl-bis(methylene) dimalonic acid (ABDA, 3.3 mg, 0.008 mmol) was dissolved in 40 mL PBS (1X, 200 μM). 50 μL of the ABDA stock was combined with 50 μL of IrCN stock in a polypropylene 96 well plate. The plate was irradiated at 405 nm (96 LED array (250 mW/LED)) in 30 second intervals (with 30 s shaking) before having the absorbance measured at 384 nm.

A control with ABDA (no photocatalyst) showed that under the irradiation, ABDA degraded, invalidating this approach.

For RNO assay:[6]

N,N-dimethyl-4-nitrosoaniline (RNO, 1.2 mg, 0.008 mmol) and *L*-histidine (62 mg, 0.4 mmol) were dissolved in 40 mL PBS (1X, 200 μM RNO). 50 μL of the RNO stock was combined with 50 μL of IrCN stock in a polypropylene 96 well plate. The plate was irradiated at 405 nm (96 LED array (250 mW/LED)) in 10 second intervals (with 30 s shaking) before having the absorbance measured at 420 and 440 nm.

$Ru(bpy)_3$ control showed a significant decrease in absorbance at 420 and 440 nm, indicating that the ROS detection method works. However, no decrease was observed with any of the IrCN library.

2.8 Stability of Purified Compounds

Purified compounds were dissolved in DMSO to make solutions at 10 mM concentration. These were diluted down to 50 μM for stability tests with the following media: DMSO, DMSO:H_2O (1:1), DMSO:PBS 1X (1:1), H_2O, PBS (1X). UV-Vis spectra (280 – 600 nm, 10 flashes) were collected over 21 h to monitor the stability of these complexes at 37 °C.

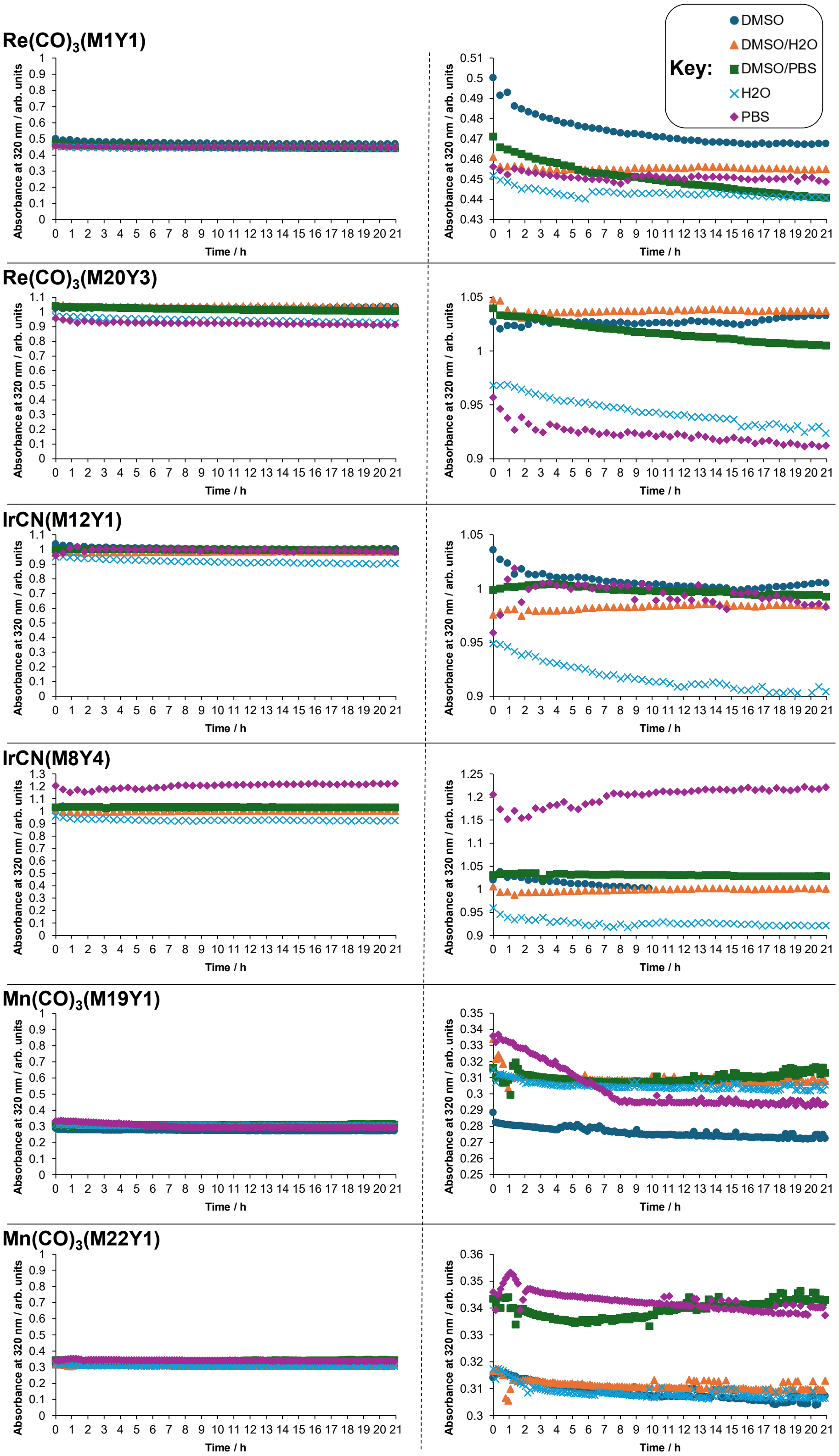

Figure S20: Stability of pure compounds, absorbance measured at 320 nm at 37 °C. Left = stability graphs; Right = the same graphs with the scales expanded.

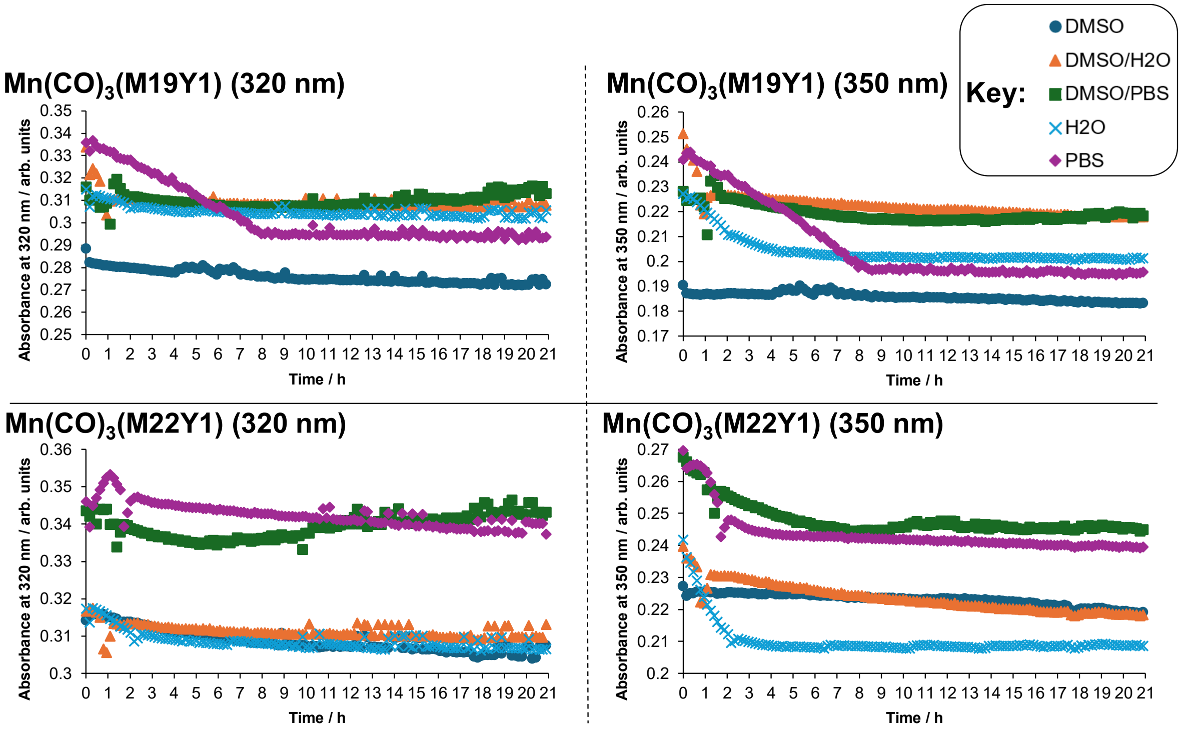

Figure S21: Stability of $Mn(CO)_3$ compounds, absorbance measured at 320 nm and 350 nm at 37 °C

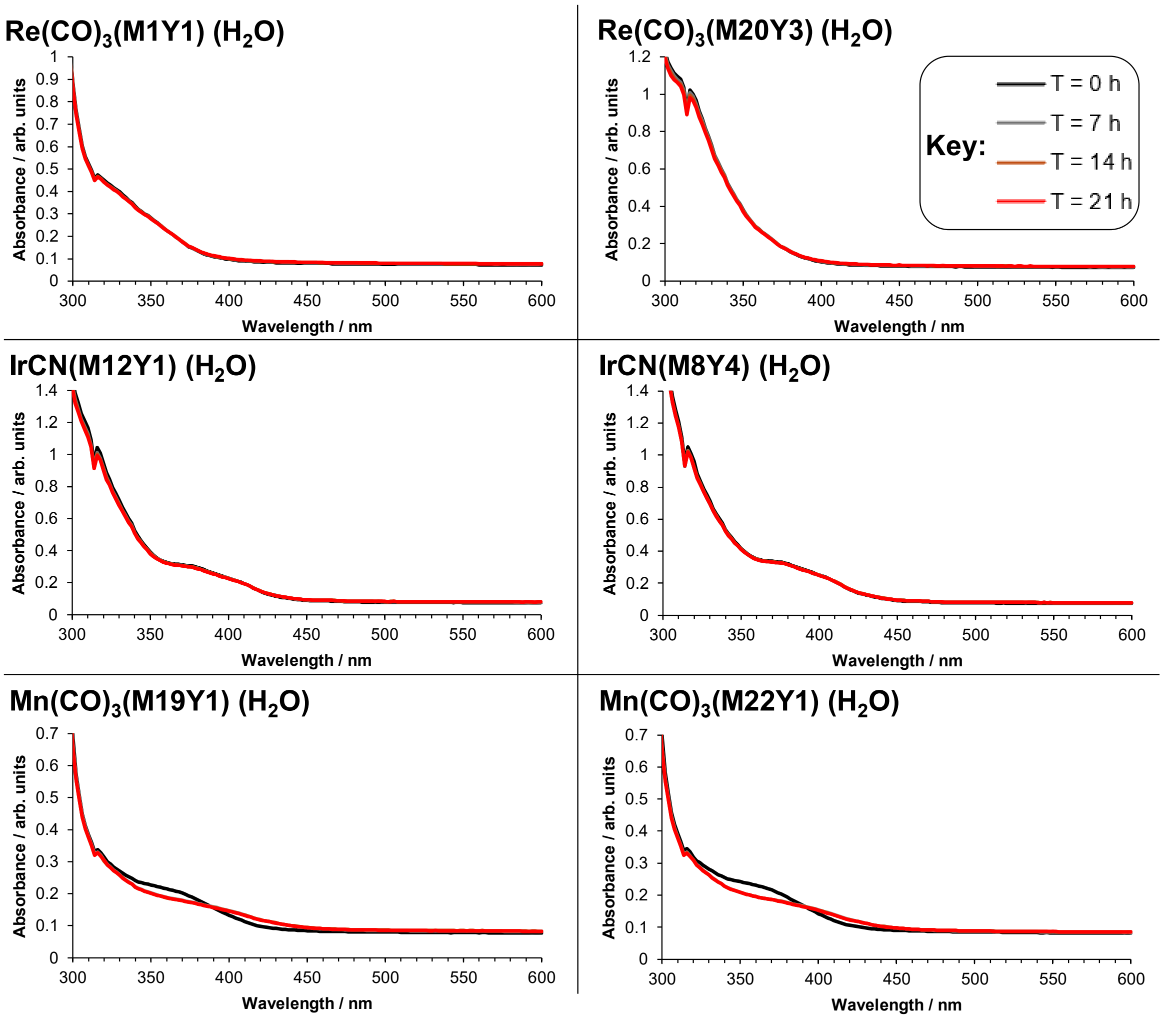

Figure S22: Example UV-Vis spectra in H_2O for the pure compounds.

Table S2: Change in absorbance (A.U., 320 nm) over 21 h in different solvents

	Solvent				
**Compound**	**DMSO**	**DMSO/H_2O (1:1)**	**DMSO/PBS 1X (1:1)**	**H_2O**	**PBS 1X**
**$Re(CO)_3$(M1Y1)**	0.0313	0.0053	0.0302	0.0107	0.0065
**$Re(CO)_3$(M20Y3)**	-0.0056	0.0112	0.0355	0.039	0.0463
**IrCN(M12Y1)**	0.03	-0.0091	0.0059	0.0452	-0.0251
**IrCN(M8Y4)**	-	0.0045	0.0042	0.0408	-0.0187
**$Mn(CO)_3$(M19Y1)**	0.0141	0.0264	0.0037	0.0099	0.0403
**$Mn(CO)_3$(M22Y1)**	0.0106	0.0041	-0.0016	0.0083	0.0062

2.9 LCMS Processing

LCMS spectra were automatically processed using bespoke Python and R code. This processing was used to obtain purity data (as peak area% of the total spectrum), to characterize the compounds (using mass spec associated with the UV peak), to obtain retention times of the compounds as a pseudo logP measurement, and to generate the spectra in the supplementary data associated with this paper.

As a workflow, the LCMS data was obtained from the ThermoScientific LCMS as .raw files. These files were converted to .mzml files using the ThermoRawFileParser (version 1.4.4) integrated with Python.[7] .mzml files were then converted into .csv files using R code (using the RaMS library). A list of target masses for the complexes in the files being processed was prepared. Then, using custom Python code, the mass list was searched and filtered for the target mass, and the associated UV peak (using UV3, 254 nm) was found. The UV peak was integrated to determine the %peak area as a total area, and the UV trace and extracted ion chromatogram were plotted in a single graph (using normalized data). The peak was labeled, and its retention time extracted.

To allow for accurate integration and account for baseline drift, a baseline correction was used, where the UV3 trace of a blank sample (60% MeCN in H_2O) was subtracted from the UV trace. An example of the spectra generated is shown below. For the IrCp* libraries, both the parent ion and the doubly charged [M – Cl, + MeCN]$^{2+}$ species were observed at different retention times. In this case, the sum of the two species was used for conversion, and only the parent ion retention time is used.

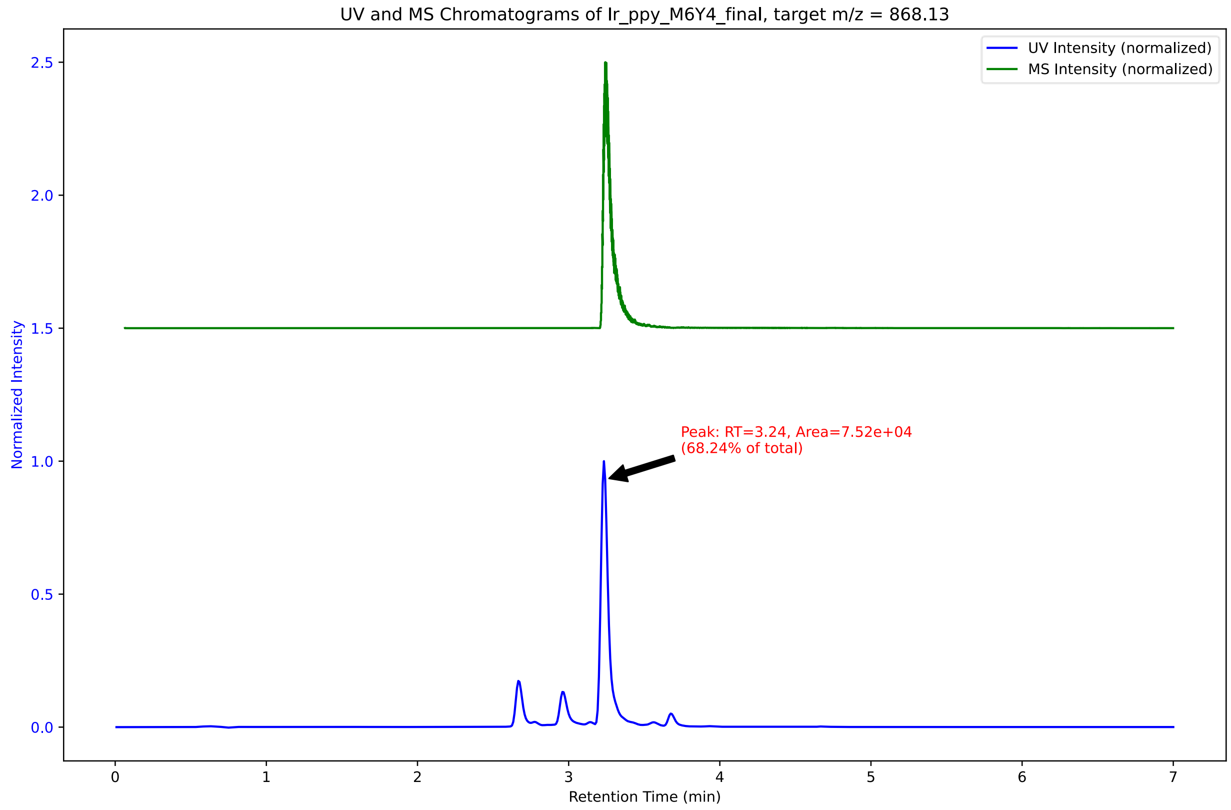

Figure S23: An example automatically processed LCMS spectrum for IrCN(M6Y4) (crude). The lower graph is the UV trace at 254 nm, and the upper trace is the extracted ion chromatograph for the target mass of the complex.

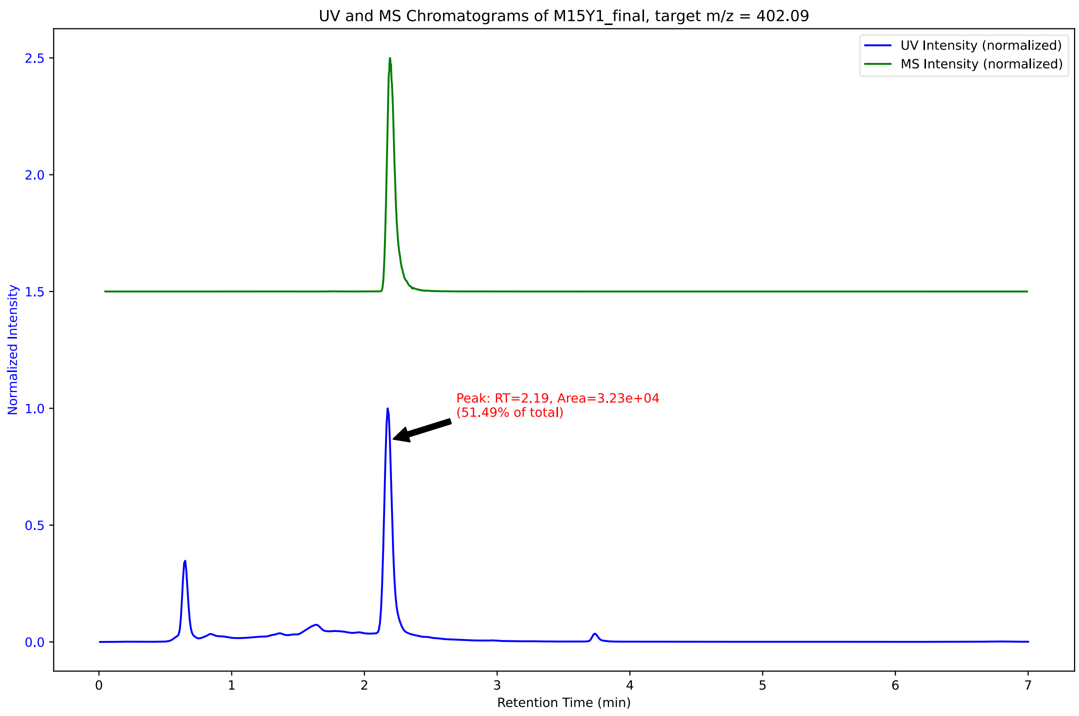

Figure S24: An example automatically processed LCMS spectrum for M15Y1 (crude). The lower graph is the UV trace at 254 nm, and the upper trace is the extracted ion chromatograph for the target mass of the complex.

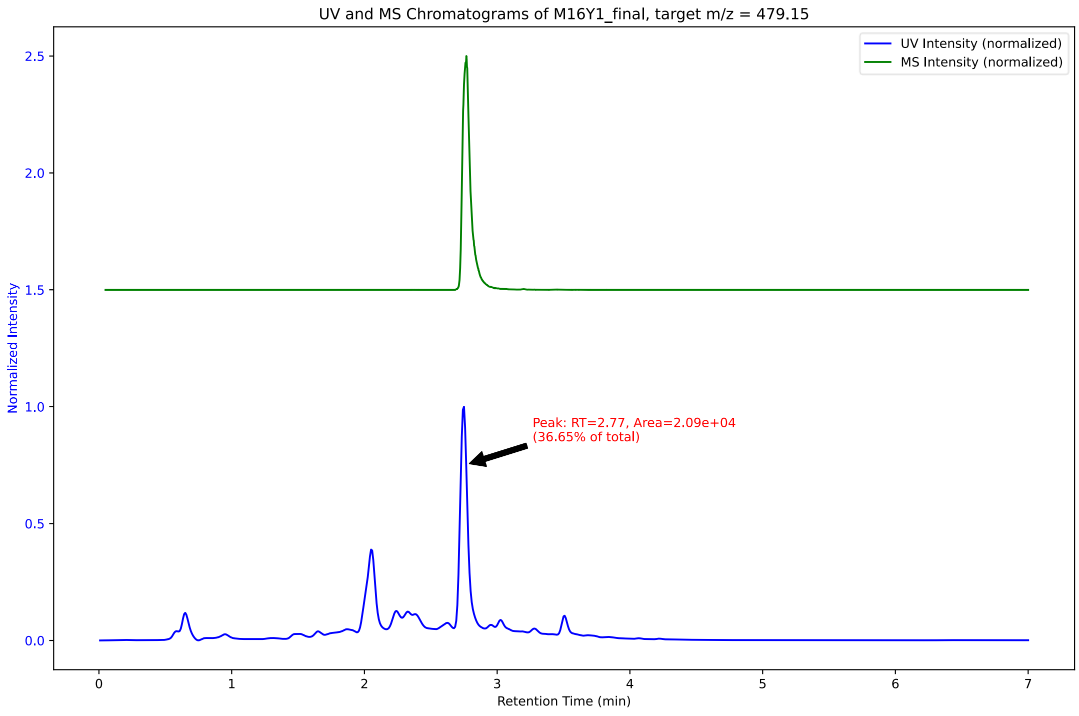

Figure S25: An example automatically processed LCMS spectrum for M16Y1 (crude). The lower graph is the UV trace at 254 nm, and the upper trace is the extracted ion chromatograph for the target mass of the complex.

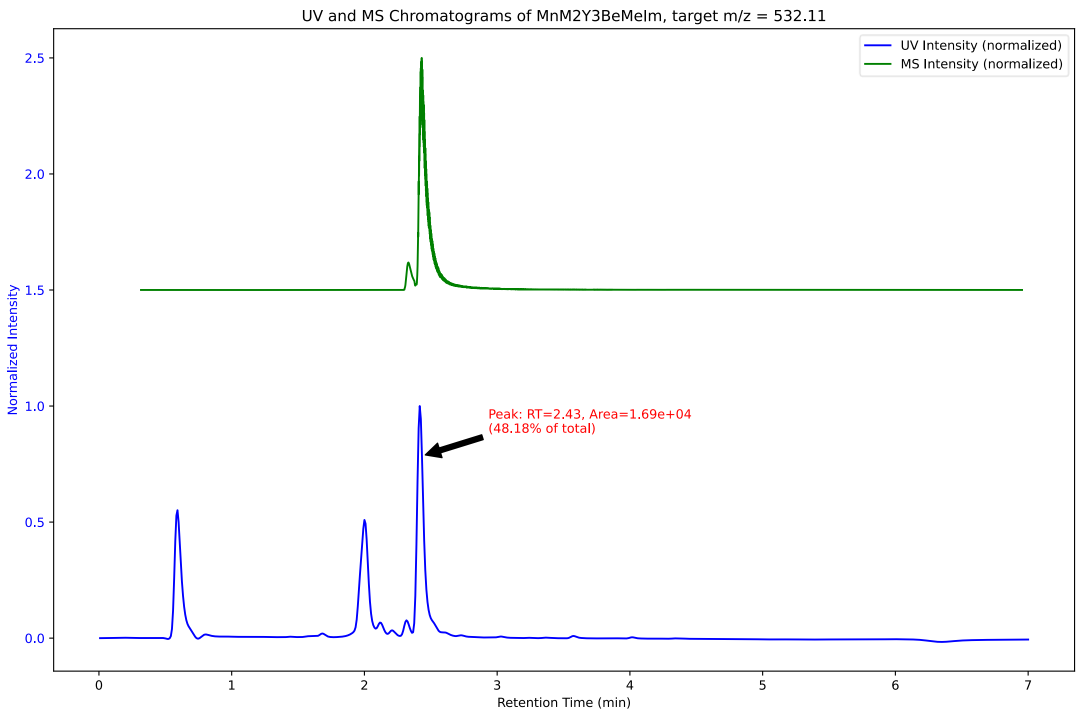

Figure S26: An example automatically processed LCMS spectrum for Mn(CO)$_3$(M2Y3) (crude). The lower graph is the UV trace at 254 nm, and the upper trace is the extracted ion chromatograph for the target mass of the complex.

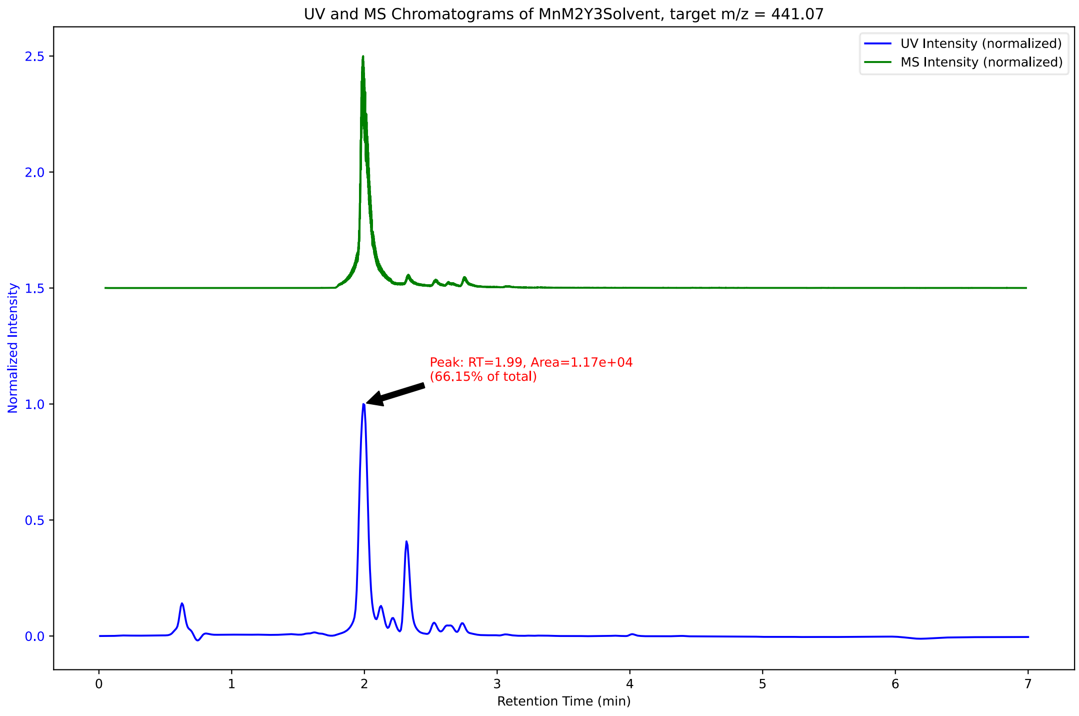

Figure S27: An example automatically processed LCMS spectrum for Mn(CO)$_3$(M2Y3) solvent adduct (crude). The lower graph is the UV trace at 254 nm, and the upper trace is the extracted ion chromatograph for the target mass of the complex.

Table S3: Calculated and observed masses for a selection of compounds across the combinatorial libraries for characterization. The LC peak area% is calculated at 254 nm, and is not normalized for the RuCy compounds. The masses displayed are for the M+ parent ion. Values in brackets relate to the $[M - Cl + MeCN)^{2+}$ adduct for the IrCp* libraries.

Compound Identity	Calculated Mass	LC-MS mass	LC peak area%	LC Retention time
M10Y3	281.14	281.15	93.5	3.01
M13Y4	378.92	379.00	56.6	4.48
IrCN(M4Y1)	737.19	737.30	71.9	3.09
IrCN(M18Y2)	755.21	755.26	65.8	3.15
$Re(CO)_3$M5Y1	653.13	653.17	65.6	3.09
$Re(CO)_3$M3Y4	709.05	709.14	37.5	3.30
$Mn(CO)_3$M6Y2	574.14	574.04	18.9	3.10
$Mn(CO)_3$M19Y3	565.13	565.02	59.8	3.38
RuCyM22Y1	575.07	575.22	51.3	2.62
RuCyM5Y3	551.11	551.23	42.8	2.55
IrCp*M8Y1	585.14<br>(295.60)	585.30<br>(295.59)	60.2<br>(11.5)	2.38<br>(1.50)
P2A1	271.08	271.07	91.5	3.44
P7A4	329.04	329.09	88.0	3.68
RuCyP1A5	563.15	563.25	62.6	2.88
IrCp*P1A11	633.12<br>(319.59)	633.21<br>(319.59)	21.0<br>(31.6)	2.59<br>(1.73)
IrCp*P4A6	681.15<br>(343.605)	681.24<br>(343.59)	8.3<br>(2.8)	2.79<br>(1.90)

3. Biological Testing

The following bacterial strains were used for testing:

Enterococcus faecalis CCUG 19916T (Gram positive)

Enterococcus faecium CUG 19434 (Gram positive)

Staphylococcus aureus CCUG 19434 (Gram positive)

Escherichia coli NCTC 13476 (Gram negative)

Bacterial stock solutions were prepared as follows. Single bacterial colonies were grown on Tryptic Soy Broth (TSB, Gram positive) or Miller's LB Broth (LB, *E. coli*) agar. A single colony was removed and placed in the relevant growth medium (5 mL, TSB or LB), and incubated overnight at 37 °C (200 rpm). 100 µL of this overnight culture was added to 5 mL of the same growth medium, and the day culture was incubated at 37 °C (200 rpm) until the OD600 was 0.4 – 0.9 (approx. 4 h). A sample of this culture was diluted with Mueller Hinton (MH) broth to OD600 = 0.022, at which point the bacterial cultures were added to plates for testing.

For all testing, samples were incubated with bacteria for 16 – 20 h at 37 °C, after which the absorbance at 600 nm was measured using a plate reader.

Antibiotic controls were Vancomycin (stock solution at 1 mg / mL in water, MIC beginning at 20 µg / mL) for Gram positive bacteria, and Polymyxin B (stock solution at 1 mg / mL in water, MIC beginning at 20 µg / mL) for *E. coli.*

Unless stated otherwise, 2 biological repeats per sample were recorded, with each having 2 technical repeats. Polystyrene 96 well plates from TPP and CytoOne were used in all cases (sterile, DNA, DNase, RNase, pyrogen free). DMSO and other solvents were kept below 5% volume in the final solutions for biological testing.

3.1 Single Dose Response (SDR)

Samples were diluted to 750 µM using DMSO/H_2O (to give a 50:50 stock solution). Of these stock solutions, 20 µL (for 50 µM) or 5 µL (for 12.5 µM) per sample was added to 96 well plates. MH growth media was added to each well (making up the volume to 290 µL), followed by the relevant bacteria in MH growth media (10 µL). The plates were incubated for 16 – 20 h at 37 °C, after which the absorbance at 600 nm was measured using a plate reader.

For the following plots, a pale blue dot indicates total bacterial growth inhibition at 50 µM of compound, and a dark blue dot inside a pale blue dot indicates total bacterial growth inhibition at both 12.5 µM and 50 µM of compound. A compound is classed as active if ≥3 of 4 repeats showed inhibition (below 0.1 absorbance at 600 nm).

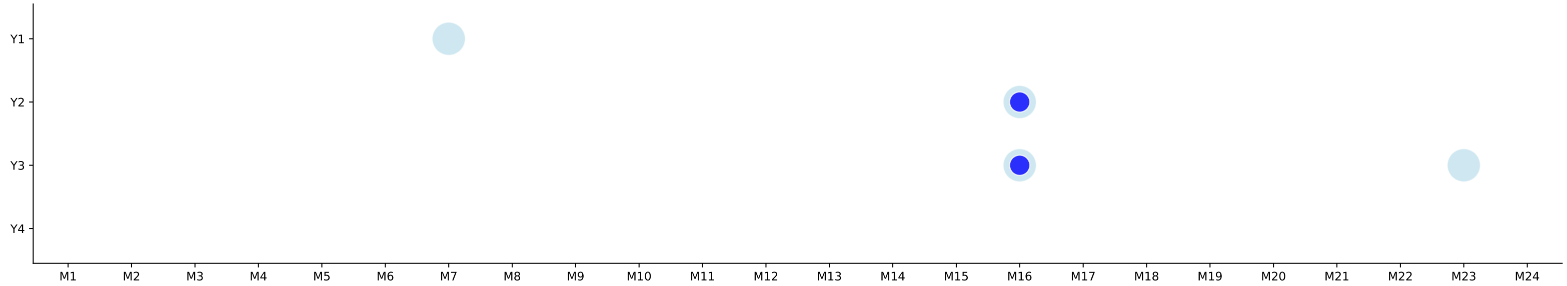

Figure S28: Single dose response at 50 and 12.5 µM of the Tz-4-P library against *S. aureus*.

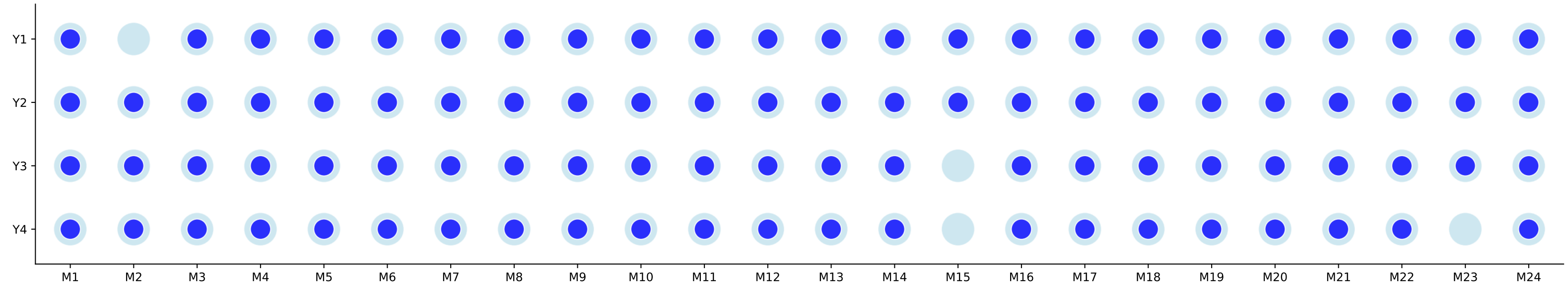

Figure S29: Single dose response at 50 and 12.5 µM of the IrCN library against *S. aureus*.

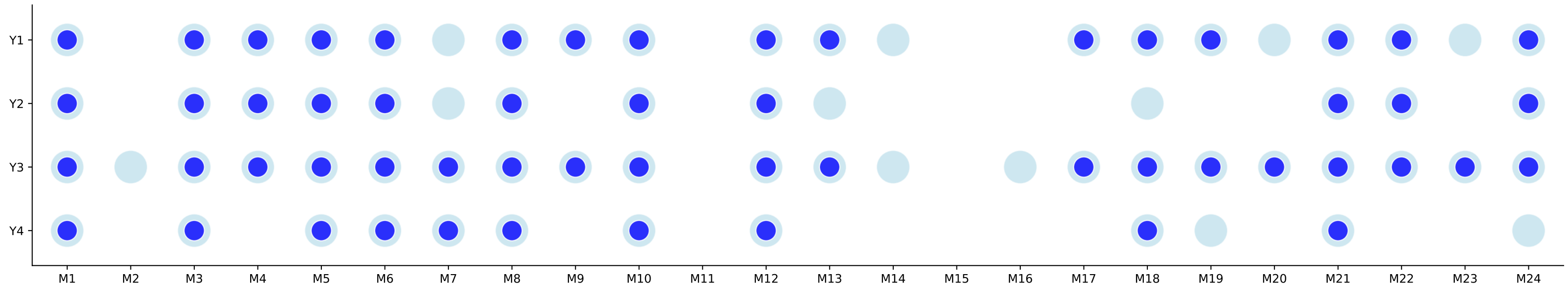

Figure S30: Single dose response at 50 and 12.5 µM of the $Re(CO)_3$ library against *S. aureus*.

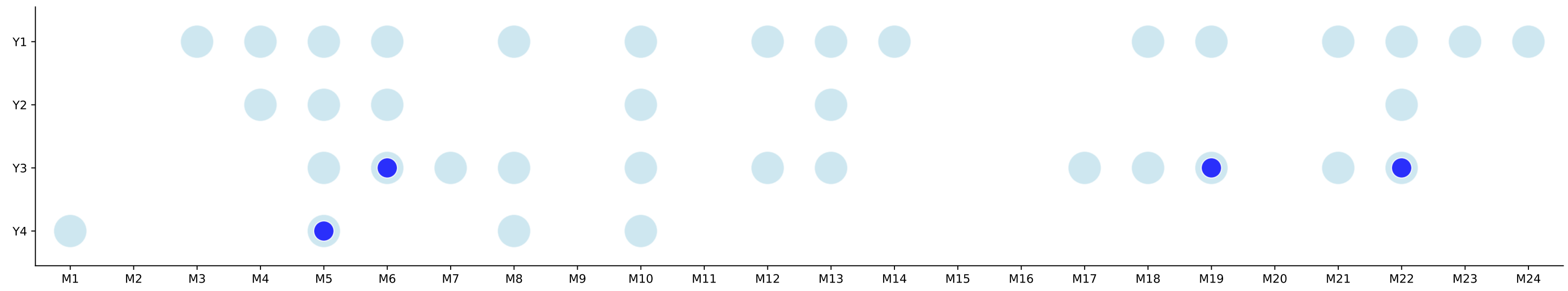

Figure S31: Single dose response at 50 and 12.5 μM of the $Re(CO)_3$(Solvent) library against *S. aureus*.

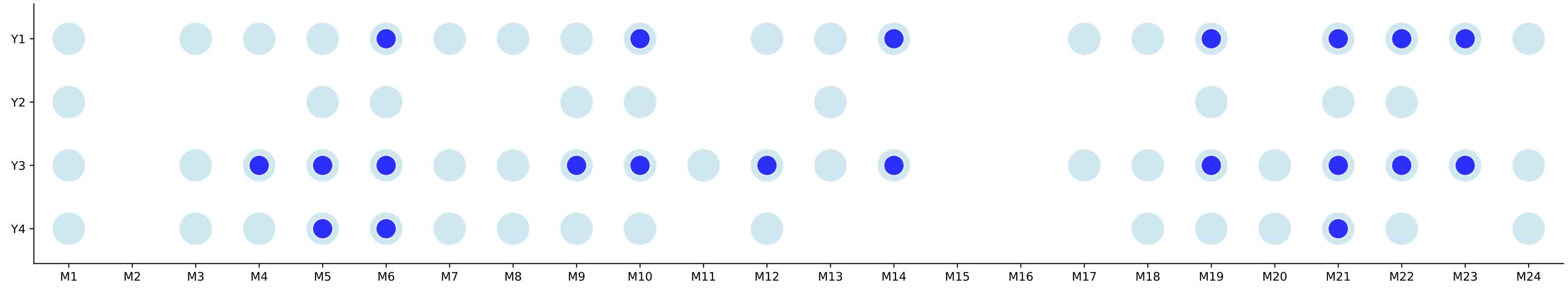

Figure S32: Single dose response at 50 and 12.5 μM of the $Mn(CO)_3$() library against *S. aureus*.

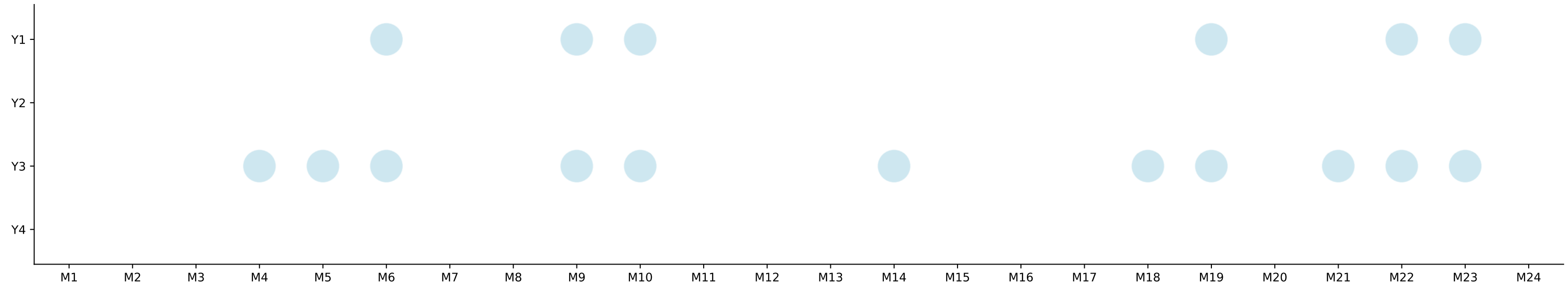

Figure S33: Single dose response at 50 and 12.5 μM of the $Mn(CO)_3$(Solvent) library against *S. aureus*.

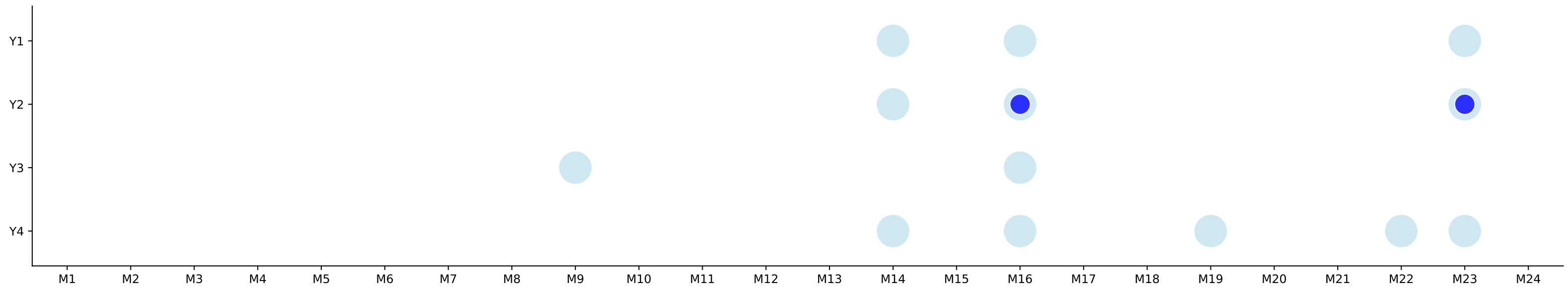

Figure S34: Single dose response at 50 and 12.5 μM of the RuCy(Tz-4-P) library against *S. aureus*.

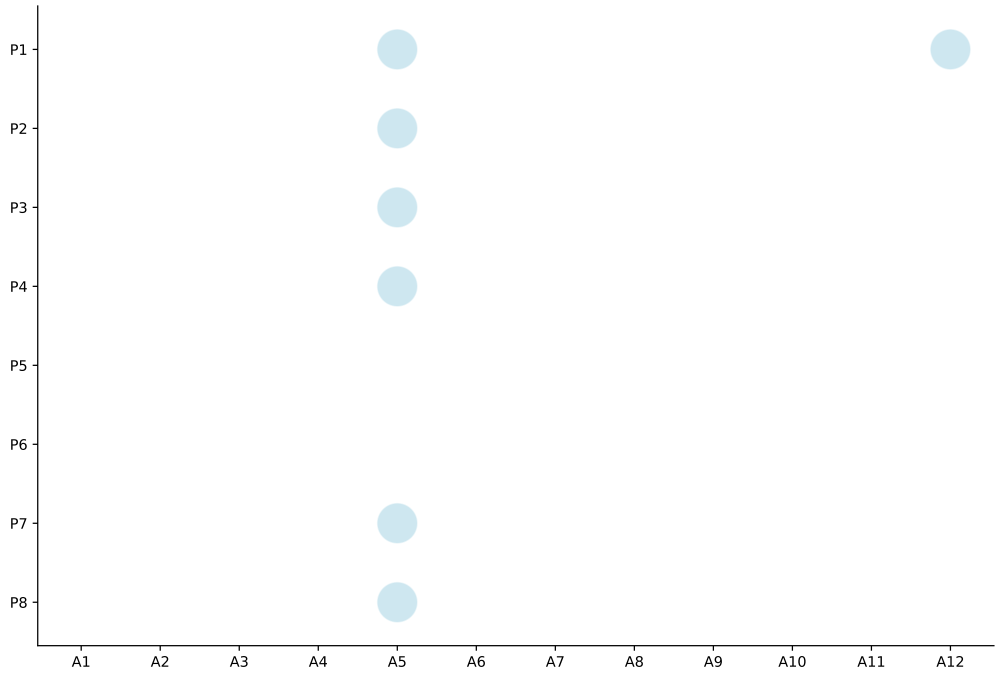

Figure S35: Single dose response at 50 and 12.5 µM of the RuCy(Tz-1-MP) library against *S. aureus.*

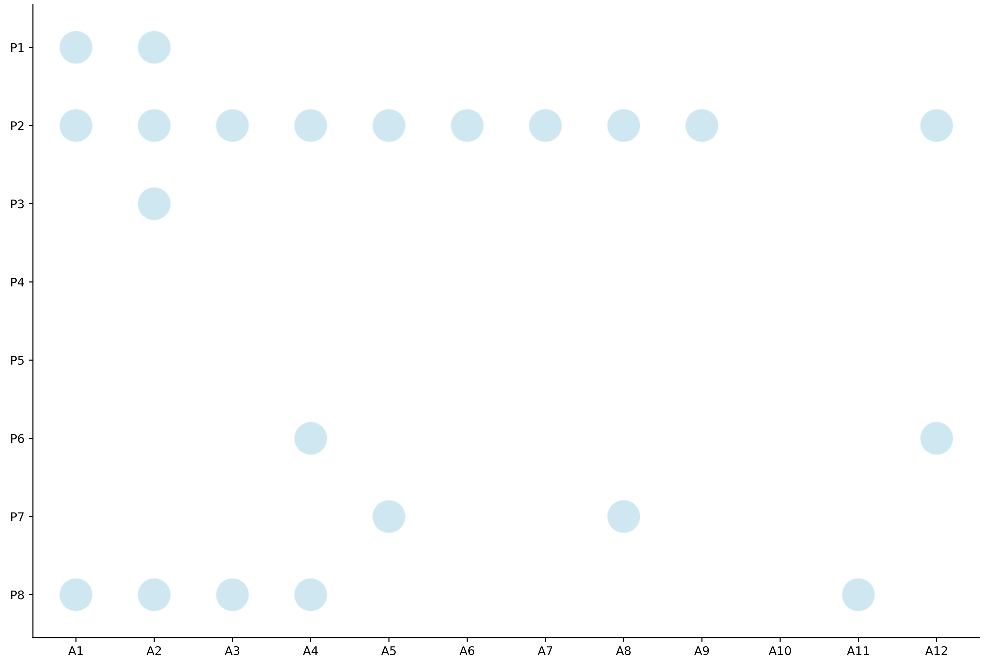

Figure S36: Single dose response at 50 and 12.5 µM of the Tz-1-MP library against *S. aureus.*

3.2 Minimum Inhibitory Concentration (MIC)

Stock solutions of samples were diluted with DMSO/H_2O (to give a 50:50 stock solution) so that 6 uL of stock solution can be added to 300 μL of MH growth media to give an initial concentration of either 50 μM (crude compounds) or 100 μM (pure compounds). These solutions were serially diluted (150 μL into 150 μL) 7 times (crude compounds) or 11 times (pure compounds) to give solutions of 150 μL. To these, 5 μL of the relevant bacterial strain was added in MH growth media. The plates were incubated for 16 – 20 h at 37 °C, after which the absorbance at 600 nm was measured using a plate reader.

For testing with Polymyxin B nonapeptide, a solution of 300 μg / mL was prepared, and 5 μL of this was added to each well immediately before the addition of *E. coli.*

Data for the MICs of purified compounds are shown below (in the tables, a "-" means not active at any of the tested concentrations):

Table S4: MIC data for metal scaffold controls (starting concentration 50 μM) against *Staphylococcus aureus* CCUG 19434 (MH growth media)

	S. aureus **(biological repeat 1) MIC (μM)**		***S. aureus*** **(biological repeat 2) MIC (μM)**	
	**Statistical repeat**		**Statistical repeat**	
**Compound**	**1**	**2**	**1**	**2**
**$Mn(CO)_5Br$**	-	-	-	-
**$Re(CO)_5Cl$**	-	-	-	-
**[Ru(*p*-cymene)Cl_2]$_2$**	-	-	-	-
**$[Ir(ppy)_2Cl]_2$**	50	50	50	50
**Vancomycin (μg / mL)**	0.625	0.625	0.625	0.625

Table S5: MIC data for purified compounds against *Escherichia coli* NCTC 13476 (MH growth media)

	E. coli **(biological repeat 1) MIC (μM)**			***E. coli*** **(biological repeat 2) MIC (μM)**		
	**Statistical repeat**			**Statistical repeat**		
**Compound**	**1**	**2**	**3**	**1**	**2**	**3**
**IrCN(M8Y4)**	-	-	-	-	-	-
**IrCN(M12Y1)**	-	-	-	-	-	-
**$Re(CO)_3$(M20Y3)**	-	-	-	-	-	-
**$Re(CO)_3$(M1Y1)**	100	100	100	100	100	100
**$Mn(CO)_3$(M19Y1)**	-	-	-	50	-	-
**$Mn(CO)_3$(M22Y1)**	100	100	100	100	100	100
**Polymyxin B (μg / mL)**	0.313	0.313	0.313	0.625	0.313	0.313

Table S6: MIC data for purified compounds against *Escherichia coli* NCTC 13476 treated with Polymyxin B nonapeptide (PMBN) (MH growth media)

	E. coli + PMBN (biological repeat 1) MIC (μM)			*E. coli* + PMBN (biological repeat 2) MIC (μM)		
	Statistical repeat			Statistical repeat		
**Compound**	**1**	**2**	**3**	**1**	**2**	**3**
**IrCN(M8Y4)**	6.25	3.13	6.25	6.25	6.25	6.25
**IrCN(M12Y1)**	1.56	0.781	1.56	0.781	0.781	1.56
**$Re(CO)_3$(M20Y3)**	6.25	6.25	6.25	3.125	6.25	3.13
**$Re(CO)_3$(M1Y1)**	25	6.25	12.5	12.5	25	12.5
**$Mn(CO)_3$(M19Y1)**	3.13	3.13	6.25	3.13	6.25	3.13
**$Mn(CO)_3$(M22Y1)**	12.5	12.5	12.5	25	25	12.5
**Polymyxin B (μg / mL)**	0.313	0.313	0.313	0.313	0.156	0.313

Table S7: MIC data for purified compounds against *Staphylococcus aureus* CCUG 19434 (MH growth media)

	S. Aureus (biological repeat 1) MIC (μM)			*S. Aureus* (biological repeat 2) MIC (μM)		
	Statistical repeat			Statistical repeat		
**Compound**	**1**	**2**	**3**	**1**	**2**	**3**
**IrCN(M8Y4)**	0.781	0.391	0.391	0.391	0.781	0.391
**IrCN(M12Y1)**	0.391	0.195	0.195	0.195	0.195	0.391
**$Re(CO)_3$(M20Y3)**	0.391	0.391	0.391	0.391	0.391	0.391
**$Re(CO)_3$(M1Y1)**	0.781	0.391	0.781	0.391	0.781	0.781
**$Mn(CO)_3$(M19Y1)**	0.781	0.781	0.781	0.391	0.391	0.781
**$Mn(CO)_3$(M22Y1)**	6.25	6.25	6.25	3.13	3.13	3.13
**Vancomycin (μg / mL)**	1.25	1.25	1.25	0.625	0.625	0.625

Table S8: MIC data for purified compounds against *Enterococcus faecalis* CCUG 19916T (MH growth media)

	E. faecalis (biological repeat 1) MIC (μM)			*E. faecalis* (biological repeat 2) MIC (μM)		
	Statistical repeat			Statistical repeat		
**Compound**	**1**	**2**	**3**	**1**	**2**	**3**
**IrCN(M8Y4)**	1.56	1.56	1.56	1.56	1.56	3.13
**IrCN(M12Y1)**	1.56	1.56	1.56	1.56	1.56	1.56
**$Re(CO)_3$(M20Y3)**	0.781	0.781	0.781	0.781	1.56	0.781
**$Re(CO)_3$(M1Y1)**	3.13	3.13	3.13	3.13	3.13	1.56
**$Mn(CO)_3$(M19Y1)**	3.13	6.25	3.13	6.25	6.25	6.25
**$Mn(CO)_3$(M22Y1)**	12.5	25	25	25	12.5	25
**Vancomycin (μg / mL)**	0.313	0.313	0.313	0.625	0.313	0.313

Table S9: MIC data for purified compounds against *Enterococcus faecalis* CCUG 19916T (TSB growth media)

TSB growth media	***E. faecalis* (biological repeat 1) MIC (μM)**			***E. faecalis* (biological repeat 2) MIC (μM)**		
	**Statistical repeat**			**Statistical repeat**		
**Compound**	**1**	**2**	**3**	**1**	**2**	**3**
**IrCN(M8Y4)**	6.25	6.25	6.25	6.25	6.25	6.25
**IrCN(M12Y1)**	3.13	3.13	3.13	6.25	6.25	6.25
**$Re(CO)_3$(M20Y3)**	3.13	3.13	3.13	3.13	3.13	3.13
**$Re(CO)_3$(M1Y1)**	6.25	6.25	6.25	6.25	6.25	6.25
**Vancomycin (μg / mL)**	0.625	0.625	0.625	1.25	0.625	1.25

Table S10: MIC data for purified compounds against *Enterococcus faecium* CUG 19434 (TSB growth media)

TSB growth media	***E. faecium* (biological repeat 1) MIC (μM)**			***E. faecium* (biological repeat 2) MIC (μM)**		
	**Statistical repeat**			**Statistical repeat**		
**Compound**	**1**	**2**	**3**	**1**	**2**	**3**
**IrCN(M8Y4)**	6.25	6.25	6.25	6.25	6.25	6.25
**IrCN(M12Y1)**	3.13	3.13	3.13	3.13	3.13	3.13
**$Re(CO)_3$(M20Y3)**	3.13	3.13	3.13	3.13	3.13	3.13
**$Re(CO)_3$(M1Y1)**	12.5	12.5	12.5	12.5	12.5	12.5
**$Mn(CO)_3$(M19Y1)**	12.5	12.5	12.5	12.5	12.5	12.5
**$Mn(CO)_3$(M22Y1)**	12.5	25	25	25	25	25
**Vancomycin (μg / mL)**	0.625	0.625	0.625	0.625	0.625	0.625

3.3 Toxicity Testing

Cell culture

The human embryonic kidney (HEK) 293T cell line was cultured in Dulbecco's Modified Eagle Medium high glucose (DMEM) (Sigma Aldrich, USA) supplemented with 10% heat-inactivated foetal bovine serum (FBS), 100 μg/mL streptomycin and 100 Units/mL penicillin, and 2 mM L-glutamine. The cells were maintained in a 5% CO_2 environment, at 37 °C and the culture media were replaced with fresh media every 72 hours.

Cytotoxicity studies on crude libraries

HEK-293T cells were seeded at 10000 cells per well and incubated overnight to enable adhesion. Thereafter, the cells were treated with either the negative control (0.1%-0.28% DMSO depending on the concentration of the crude complexes) or the crude complexes at 50 μM for 24 hours. To quantify cell viability, the cells were treated with the 3-(4,5-dimethylthiazol-2-yl)-2,5 diphenyltetrazolium bromide (MTT) salt (Apollo Scientific, UK) according to the procedure described by Mosmann.[8] The absorbance at 590 nm was measured using a TECAN well plate reader instrument M1000. Two biological repeats in duplicate

were performed for this study, and the recorded absorbances were adjusted for the respective crudes and growth media.

Determination of the cytotoxic concentration 50% (CC_{50})

HEK-293T cells were seeded at 10000 cells per well and incubated overnight to allow adhesion. 5 mM stock solutions of pure compounds were prepared in 1:1 DMSO : PBS 1X. In a 1.5 mL 96 well dilution plate, starting concentrations of 150 µM and 200 µM of each compound were made in growth media (1 mL). 500 uL of these were serial diluted into 500 uL of media 11 times. 500 uL of media was then added to each well added to make 1 mL of each concentration (starting at 75 µM and 100 µM). 100 uL of these solutions were added into wells containing the pre-counted cells and treated for 24 h. Thereafter, the MTT assay was used to quantify viable cells and at least three biological repeats in triplicate were conducted from which the CC_{50} was determined using GraphPad Prism v8.0.2 (GraphPad Software, California, USA). The absorbance at 590 nm was measured using a TECAN well plate reader instrument M1000. 4 biological repeats in triplicate were done for this study (12 data points). For the Mn complexes, 2 biological repeats in triplicate (6 data points) were used.

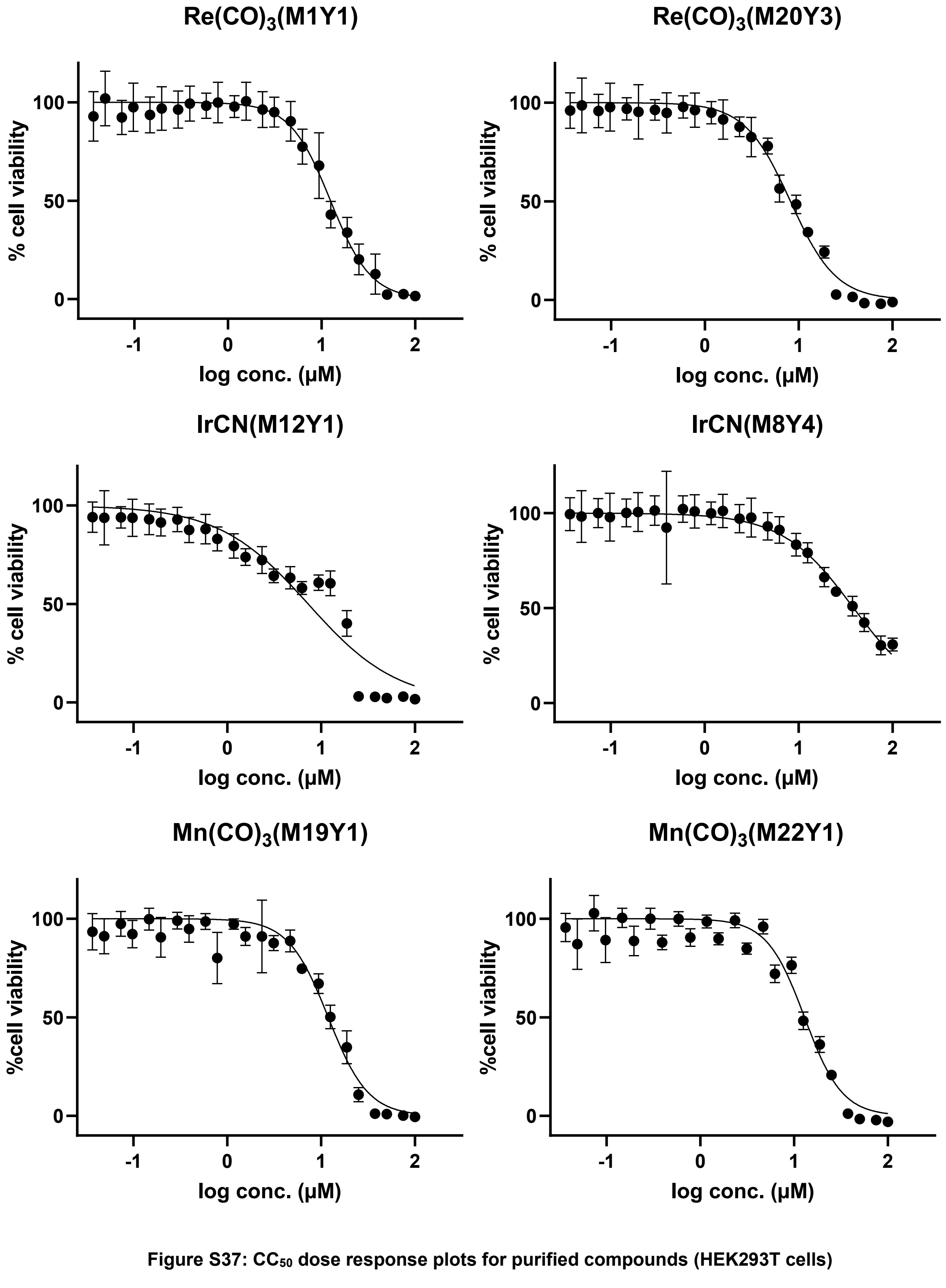

Figure S37: CC_{50} dose response plots for purified compounds (HEK293T cells)

Table S11: Calculated CC_{50} values with the associated standard error of the mean (SEM) and standard deviation (SD)

Compound	Toxicity [µM]		
	CC_{50}	SEM	SD
**$Re(CO)_3$(M1Y1)**	12.4	0.3	1.2
**$Re(CO)_3$(M20Y3)**	8.16	0.2	0.7
**IrCN(M12Y1)**	7.25	0.4	1.2
**IrCN(M8Y4)**	38.6	1.6	5.5
**$Mn(CO)_3$(M19Y1)**	11.9	0.5	1.1
**$Mn(CO)_3$(M22Y1)**	12.9	0.5	1.2

Haemolysis

Blood samples were obtained from healthy volunteers through Hull York Medical School. The haemolysis assay was adapted from a previously reported procedure.[9] Whole blood (5 mL) was centrifuged (Thermo Scientific SL 8R) at 4500 rpm for 1 h at 4 °C, and the plasma was discarded. The remaining human red blood cell (hRBC) pellet was washed three times with PBS 1X (pH 7.4): for each wash, PBS was added up to 15 mL, followed by centrifugation at 4500 rpm for 1 h at 4 °C. After the final wash, the cells were resuspended in PBS to a final volume of 35 mL. The stock solutions for the samples to be tested were 2.5 mM in PBS:DMSO (60:40). For the determination of HC_{50} and HC_{10} values, test compounds were diluted in PBS to give a concentration range from 200 µM down to 5 µM, giving a total volume of 125 µL. Dilutions were prepared in 20 µM decrements with the final points at 10 and 5 µM. Each plate included a blank medium control (PBS) and a haemolytic activity control (1% SDS in PBS). The hRBC suspension (125 µL) was incubated with the samples in PBS in a V-shaped 96-well plate for 4 hours at 25 °C. After incubation, 60 µL of supernatant was carefully pipetted to a flat bottom, clear 96-wells plate. Haemolysis was measured by absorbance at 540 nm using a plate reader (TECAN M1000). The percentage of haemolysis at each concentration was determined and the HC_{10} and HC_{50} were calculated by inhibitor vs. normalized response fit (Prism). The minimum haemolytic concentration (MHC) was determined by visual assessment of the wells after incubation. The MHC was defined as the lowest compound concentration at which visible haemolysis (red coloration of the supernatant and/or loss of intact red pellet) could be observed compared to the PBS control. Each experiment was repeated in triplicate (3 biological and 3 statistical replicates).

Table S12: Observed and calculated hemolytic concentrations. MHC = minimum haemolytic concentration, observed by eye; HC_{10} = the concentration at which 10% of the blood cells have been lysed; HC_{50} = the concentration at which 50% of the blood cells have been lysed. Calculated values are supplied with the standard error of the mean (SEM)

Compound	Concentration [µM]		
	MHC	HC_{10}	HC_{50}
$Re(CO)_3$(M1Y1)	40 – 100	73 ± 29	108 ± 36
$Re(CO)_3$(M20Y3)	20	36 ± 6	51 ± 7
IrCN(M12Y1)	10 – 20	81 ± 4	115 ± 7
IrCN(M8Y4)	40	> 200	> 200
$Mn(CO)_3$(M19Y1)	40	> 200	> 200
$Mn(CO)_3$(M22Y1)	40 – 80	87 ± 7	101 ± 4

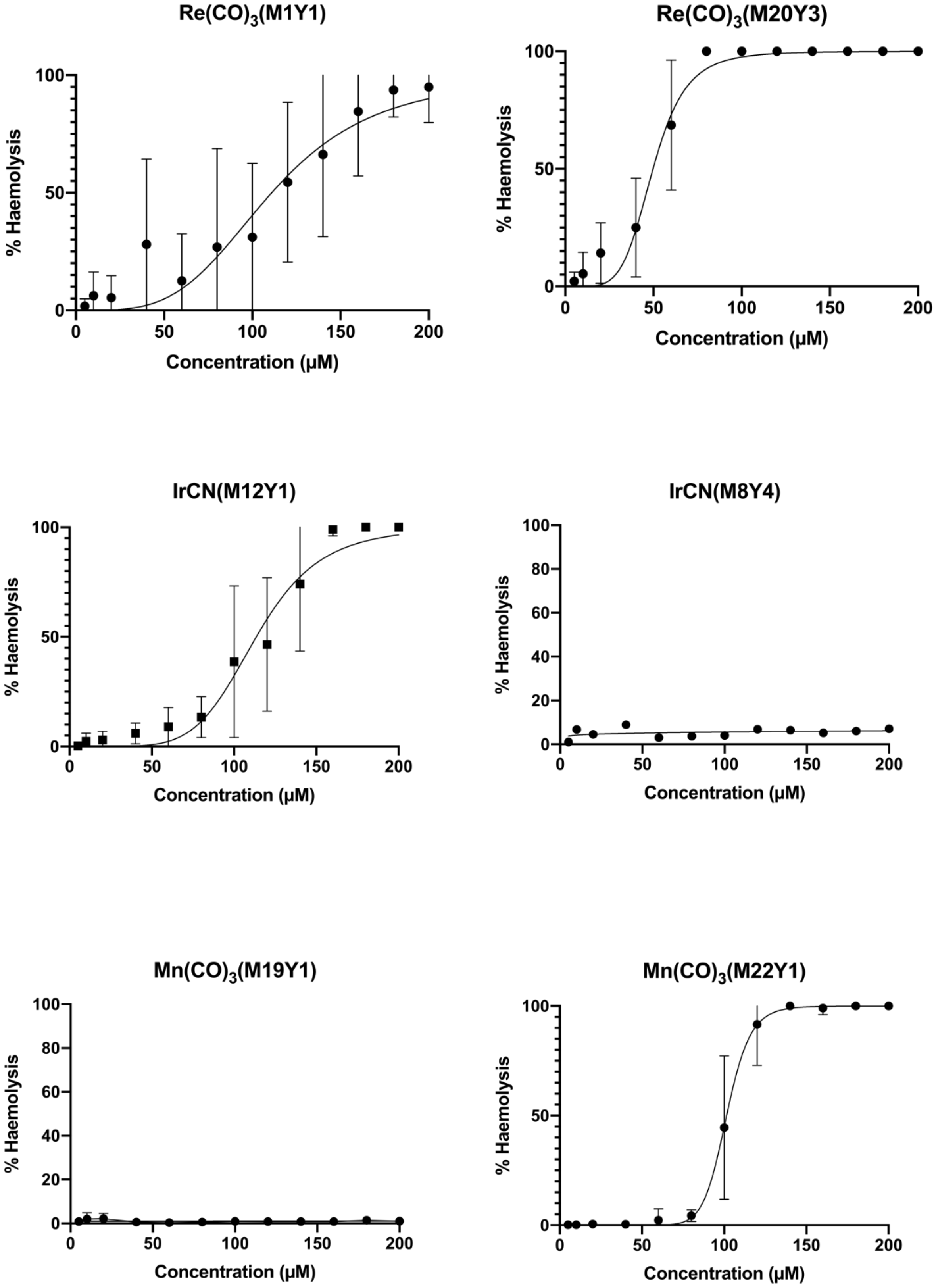

Figure S38: HC_{50} dose response plots for purified compounds (hRBC)

4. Structure-Activity Relationship Analysis

Properties were calculated utilizing Python and the corresponding RDKit and sci-kit learn libraries. Properties such as logP were calculated for the respective ligands as they constitute the changing factor between compounds. ELECTRUM fingerprints were calculated as 598-bit sized vectors using the electrum-fp library.[10]

Support Vector Machine (SVM) model was implemented using Python scikit-learn (1.7.1). All models were evaluated in a 5-fold cross validation with a train/test split of 80/20.

Table 13: Metrics for training and testing the SVM model

	AUC	**Accuracy**	**Precision**	**Recall**	**F1**
Mean	0.83	0.84	0.80	0.87	0.83
Std	0.05	0.05	0.09	0.06	0.07

Calculated logP values of the Tz-4-P library show a positive correlation with LC-MS retention time, indicating that retention time is a good descriptor of lipophilicity in these ligands.

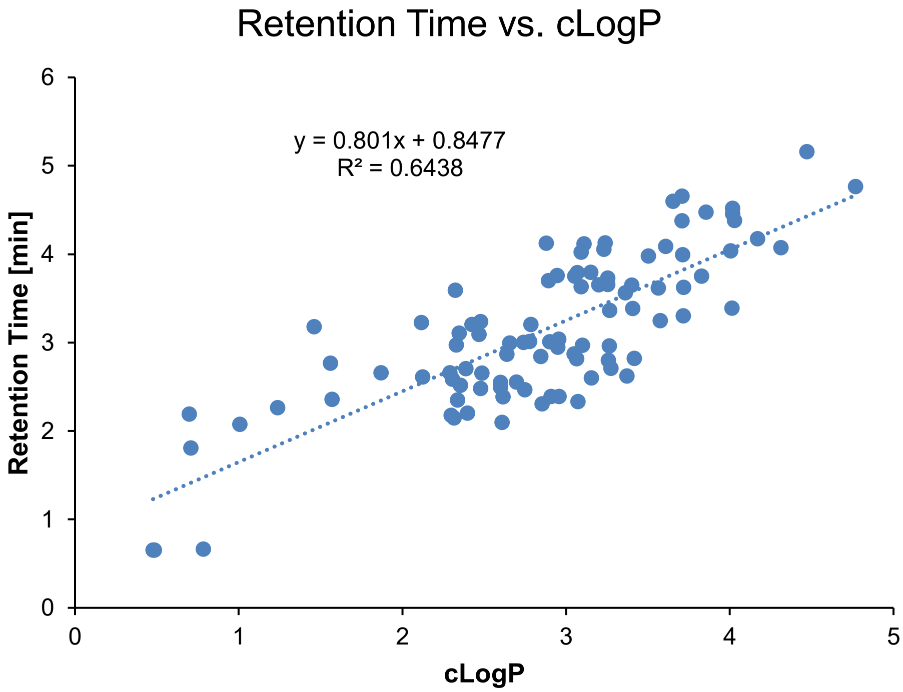

Figure S39: Plot of retention time *vs* calculated cLogP for the Tz-4-P library

$Re(CO)_3$ library correlation to ligand properties

Plots of measured and calculated properties of the $Re(CO)_3$(Tz-4-P) library have been generated. For clarity, the following terms are defined; TPSA – topological polarsurface area (the surface sum over all polar atoms in a molecule. Used to predict how well a molecule can permeate through cells), CSP3 – the fraction of sp^3 character across all carbon atoms in a molecule, MW – molecular weight.

- There is no visual correlation between TPSA and the measured MICs for the $Re(CO)_3$(Tz-4-P) library
- Highly active compounds tend to have a much narrower range of FractionSP3 values lower
- Active compounds tend to have a tighter range of Retention times than non-active compounds
- There is a visual trend between molecular weight and MIC activity, with lower MW compounds tending to be more active
- A clear correlation exists between the antimicrobial activity and toxicity of this library, with more active compounds being more toxic. However, at MIC 6.25 μM, most compounds are in the 50% viability range, so there is a good potential therapeutic window.

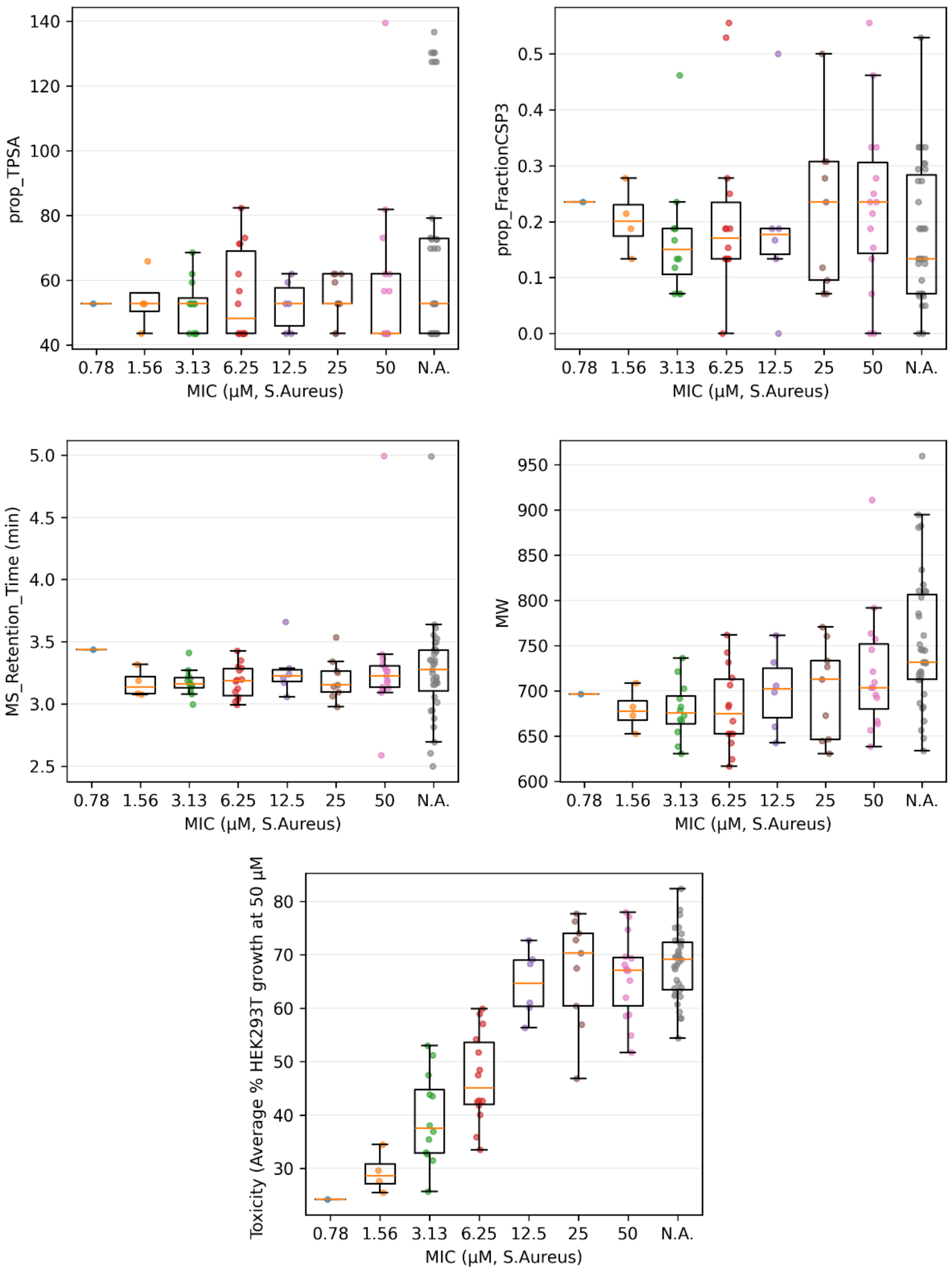

Figure S40: Correlations for the Re(CO)$_3$(Tz-4-P) library of measured and calculated properties *vs* the measured MIC of the crude compounds

Analysis with the same library for toxicity was also undertaken;

- There is no correlation between TPSA and toxicity of these compounds
- A weak correlation between MW and toxicity exists, with heavier compounds tending to be less toxic

- There is no correlation between retention time and toxicity for this library

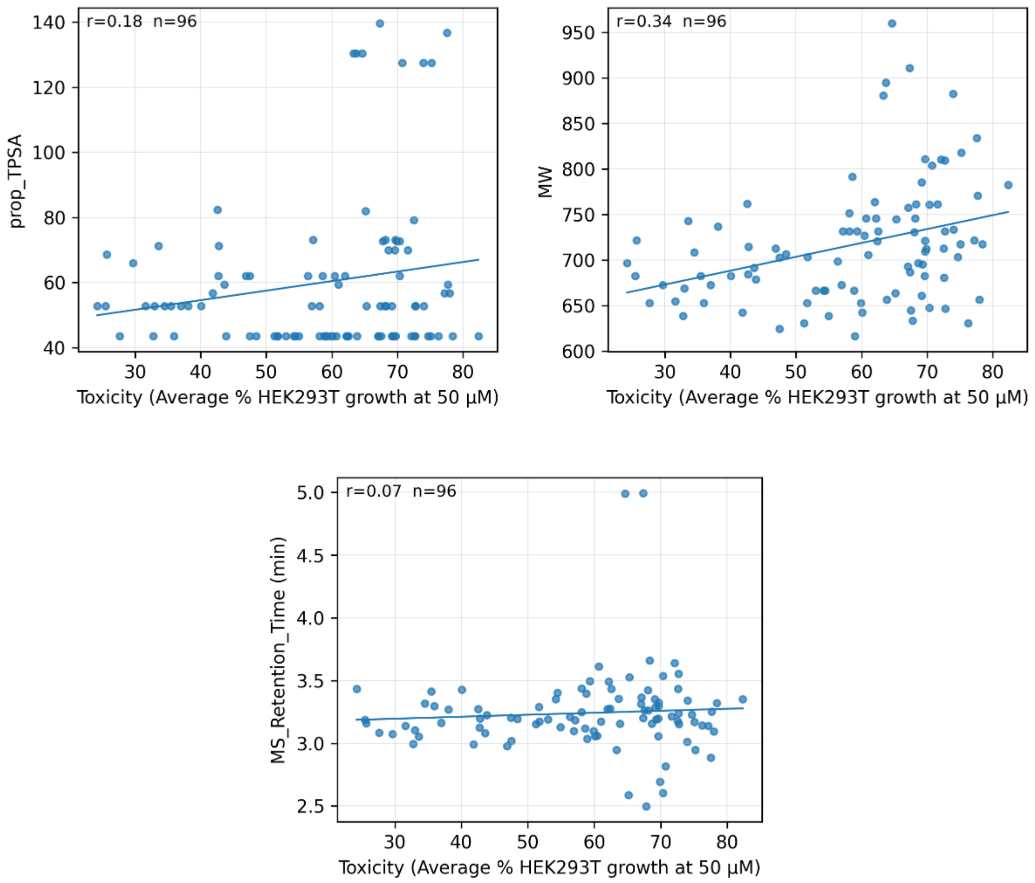

Figure S41: Correlations for the Re(CO)$_3$(Tz-4-P) library of measured and calculated properties *vs* the measured toxicity of the crude compounds

IrCN library correlation to ligand properties

A similar analysis was undertaken with regard to the IrCN(Tz-4-P) library;

- There is a weak trend between molecular weight and antimicrobial activity
- There appears to be an optimal window for TPSA in highly active compounds
- There is a weak correlation toxicity and MIC
- There is a positive correlation between toxicity and TPSA for the IrCN library

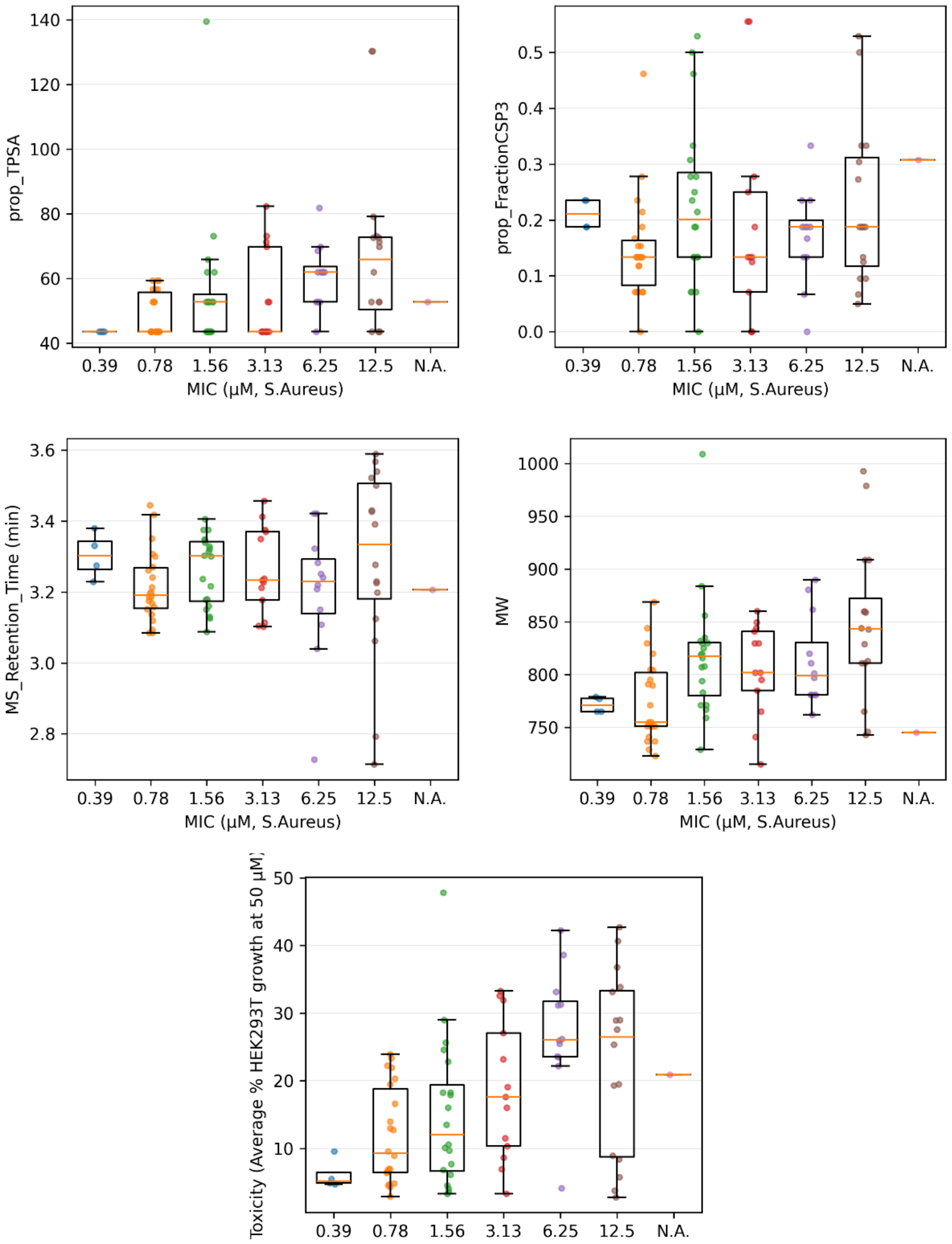

Figure S42: Correlations for the IrCN(Tz-4-P) library of measured and calculated properties *vs* the measured MIC of the crude compounds

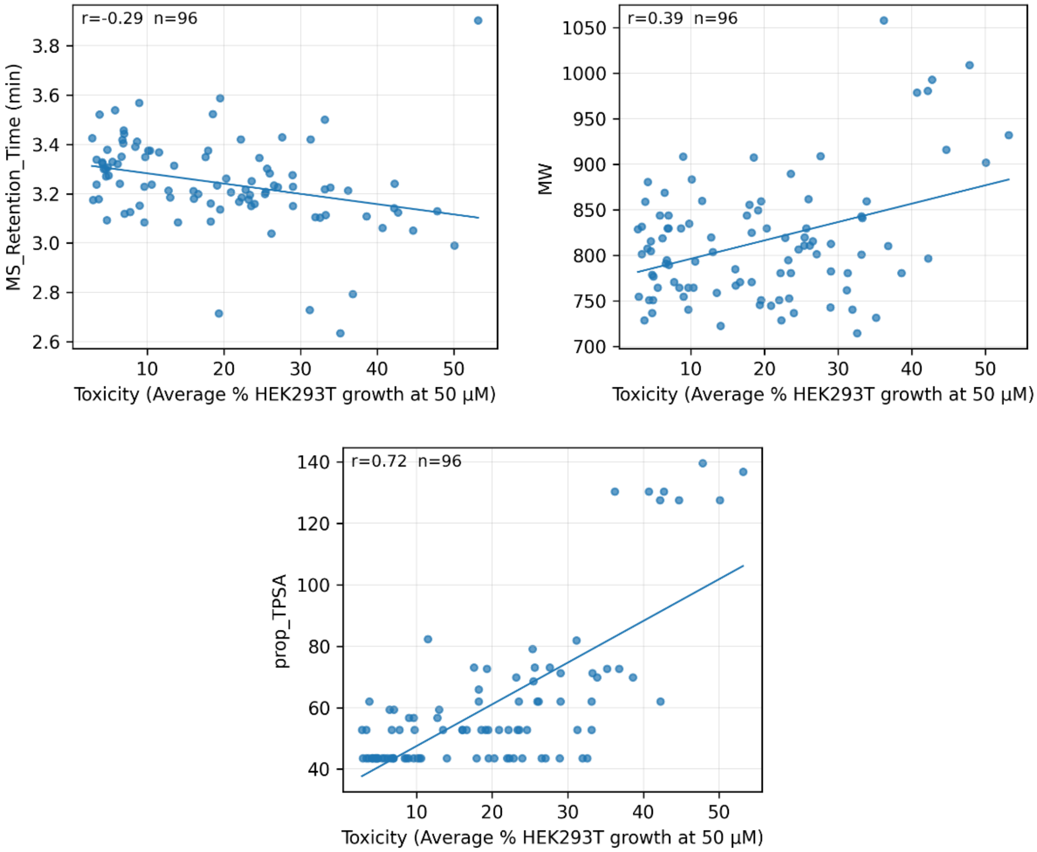

Figure S43: Correlations for the IrCN(Tz-4-P) library of measured and calculated properties *vs* the measured toxicity of the crude compounds

The effects of changing the metal scaffold on activity for the same ligands

To ascertain what the effect of changing the metal center and scaffold was on the activity of the complex, comparison graphs between the metals were plotted. For the same ligand, the change in activity is represented by a delta value. This value is derived from the MIC assay and refers to the difference in wells (and hence serial dilutions) for the same ligand with different metals. For example, a delta of -1 means that a Re complex with the same ligand is half as active as the corresponding Ir complex.

Generally, Re complexes are less active than their corresponding Ir complexes, although there are some cases in which they are more active. Similarly, Re complexes are more active than their corresponding Mn complexes, indicating that the identity of the metal scaffold has more of an impact on the bioactivity of the complex than the varying ligand.

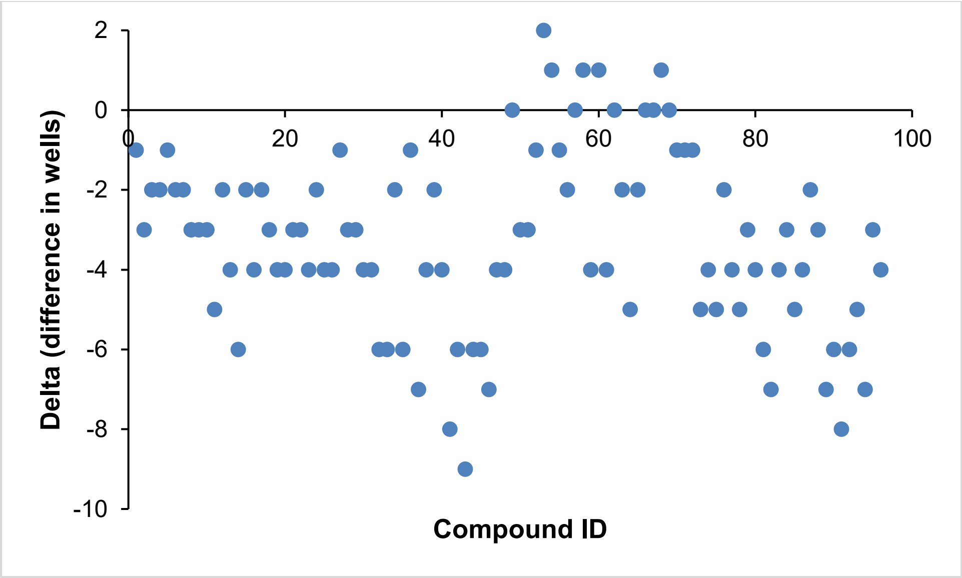

Figure S44: Difference in activity between Ir and Re complexes. Negative values mean the Re complex is less active compared to the Ir complex

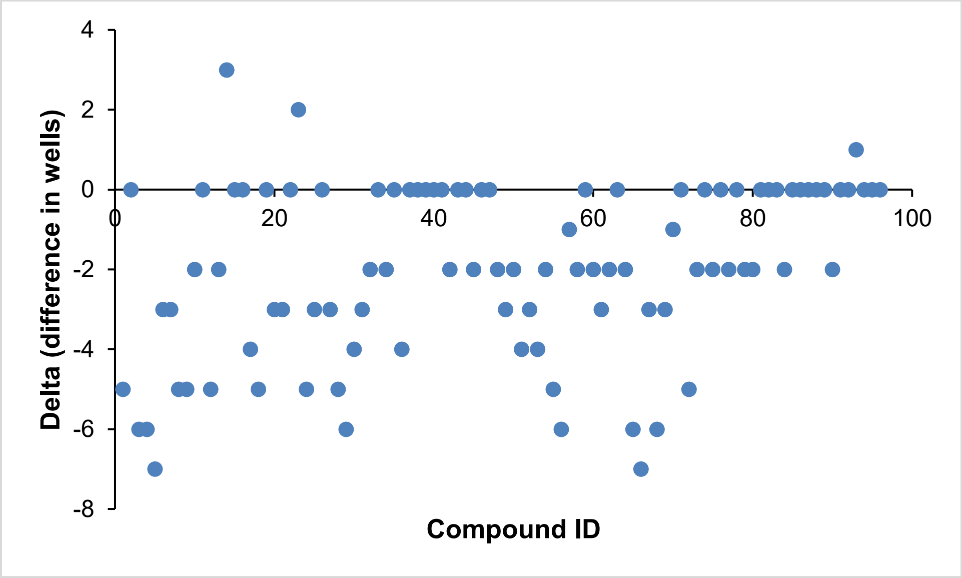

Figure S45: Difference in activity between Re and Mn complexes. Negative values mean the Mn complex is less active compared to the Re complex

Upon complexation, the retention times of the resulting metal complexes have a much tighter distribution than the free ligand, which varies significantly. This can be rationalized as a mass distribution effect, and upon complexation to a heavy metal fragment where the mass of the ligand is a lower percentage, there tends to be a tighter distribution than for lighter metal fragments.

Table S14: Summarized changes in retention time upon complexation with different metal fragments

Metal fragment	IrCN	Re	Mn	IrCp*	RuCy
**Average RT**	3.244179	3.240975	3.106592	2.575018	2.510253
**Standard deviation**	0.180071	0.328375	0.431834	0.296731	0.24624
**MW Metal fragment**	500.6	402.4	271.13	362.9	270.74
**Avg. MW Ligand**	314.5	314.5	314.5	314.5	314.5
**%Ligand**	0.386	0.439	0.537	0.464	0.537
**%Stdev**	5.55%	10.13%	13.90%	11.52%	9.81%

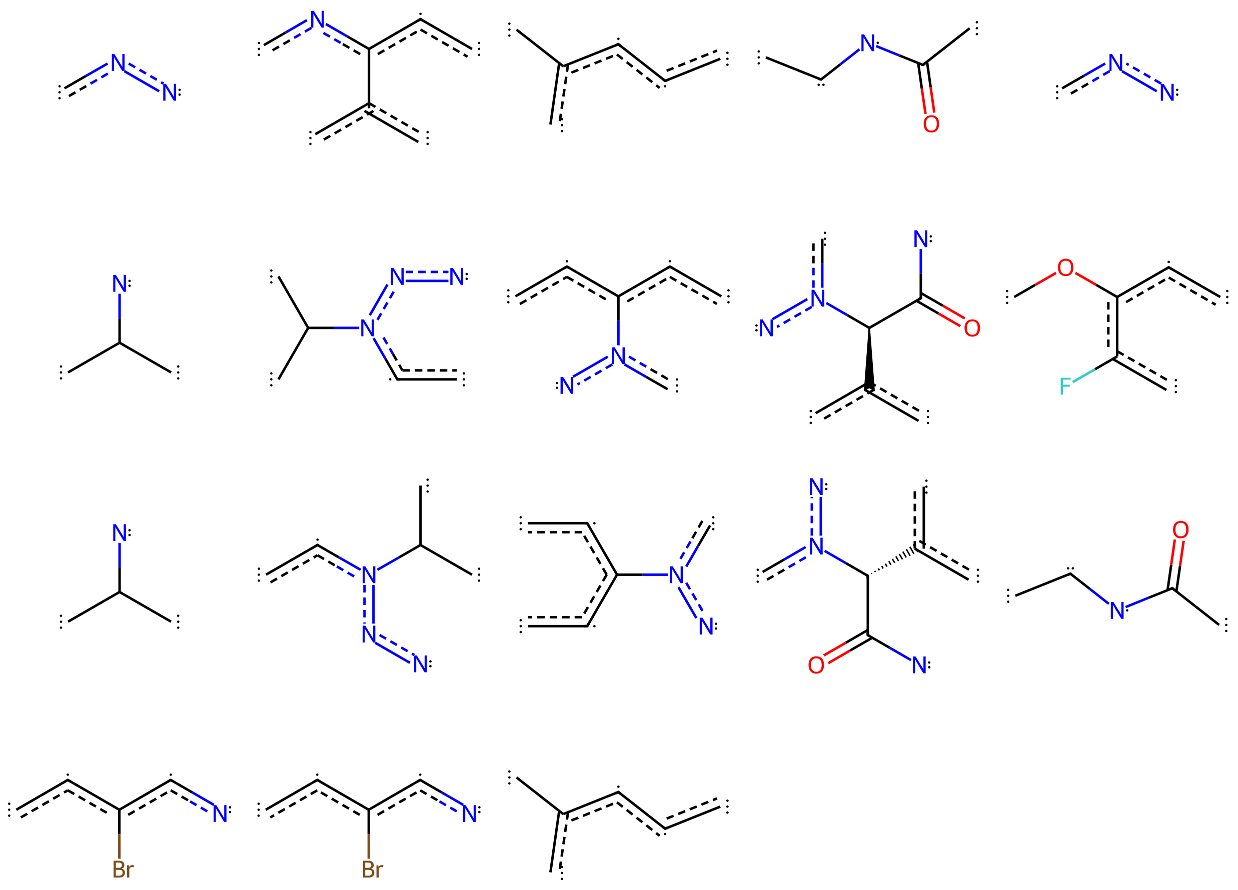

Figure S46: Molecular features that correlate with active compounds from the trained SVM model

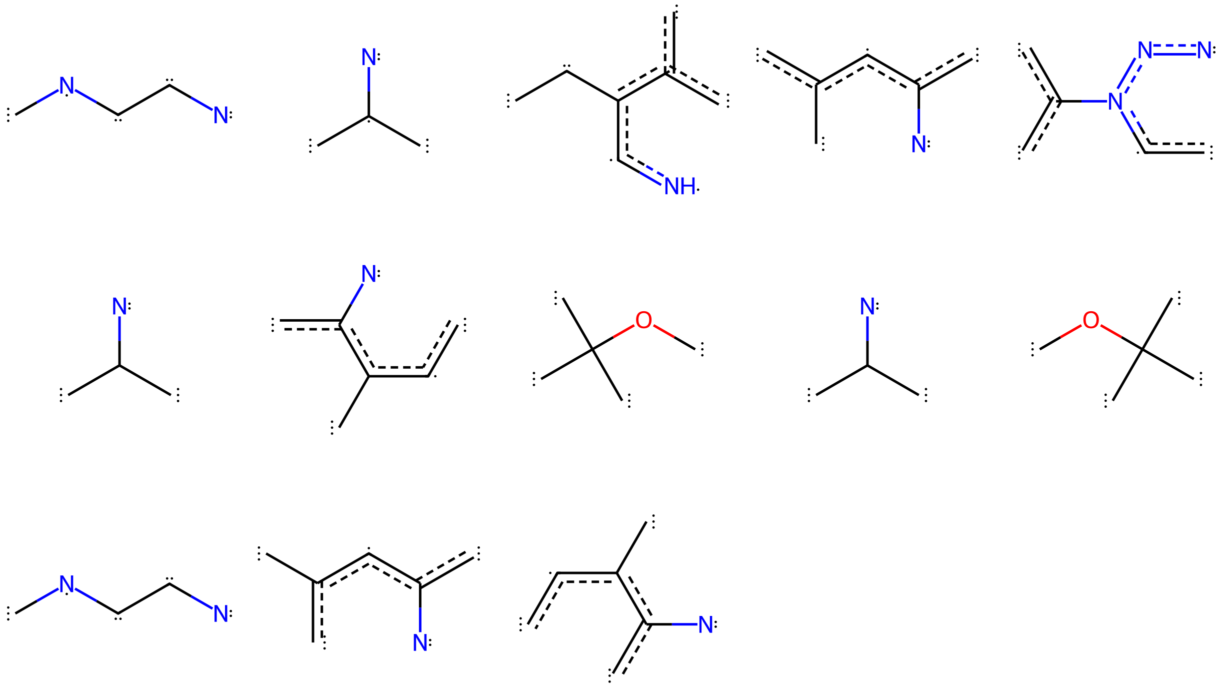

Figure S47: Molecular features that correlate with non-active compounds from the trained SVM model

5. X-Ray Crystallography

Diffraction data were collected at 110 K on an Oxford Diffraction XtaLAB Synergy HyPix-Arc 100 diffractometer with Cu-K_{α} radiation (λ = 1.54184 Å). The crystal was cooled with an Oxford Instruments Cryostream 1000. Diffractometer control, data collection, initial unit cell determination, frame integration and unit-cell refinement was carried out with "CrysAlisPro".[11] Face-indexed absorption corrections were applied using spherical harmonics, implemented in SCALE3 ABSPACK scaling algorithm.[12] OLEX2[13] was used for overall structure solution and refinement . Within OLEX2, the algorithm used for structure solution was "ShelXT dual-space".[14] Refinement was carried out by full-matrix least-squares used the SHELXL-97[14] algorithm within OLEX2. All non-hydrogen atoms were refined anisotropically. Crystalmaker® software was used to visualize the structures as well as generating the figures presented herein.

af25001: Re(CO)$_3$(M1Y1) (lab book ref. DRH-052), CCDC Deposition 2455425

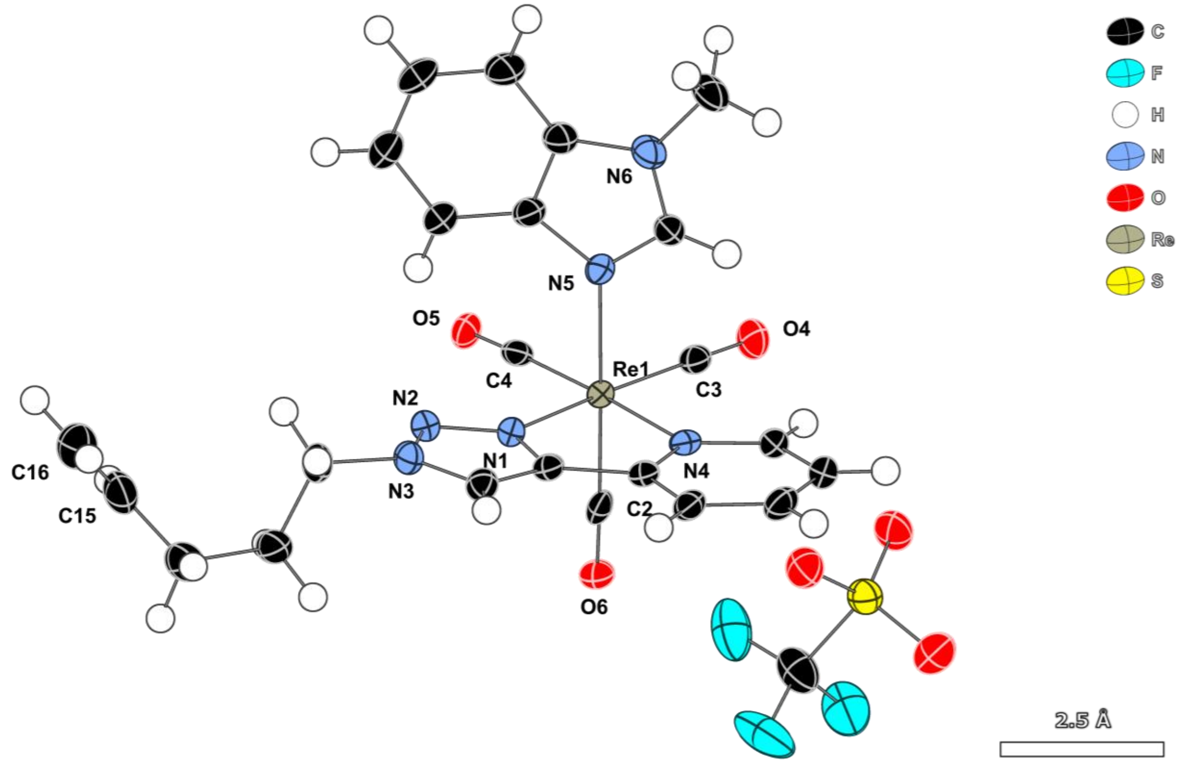

Figure S48: Structure, obtained by X-ray diffraction, of a single crystal of Re(CO)$_3$(M1Y1) (thermal ellipsoids are set at 50% probability). Selected interatomic lengths /Å: Re1-N5 = 2.190(2); Re1-N4 = 2.201(2); Re1-N1 = 2.156(2); Re1-C2 = 1.930(3); Re1-C3 = 1.922(3); Re1-C4 – 1.916(3); C15-C16 = 1.310(5). Selected interatomic angles /°: N1-Re1-N4 = 74.94(8); N1-Re1-N5 = 82.32(8); N4-Re1-N5 = 85.70(8); C3-Re1-C4 = 89.95(11); N1-Re1-C4 = 99.22(9).

Table S15: X-Ray Diffraction Data for Re(CO)$_3$(M1Y1)

Identification code	af25001
Empirical formula	$C_{24}H_{22}F_3N_6O_6ReS$
Formula weight	765.73
Temperature/K	110.00(10)
Crystal system	triclinic
Space group	P-1
a/Å	10.8034(4)
b/Å	10.8987(3)
c/Å	13.4061(5)
α/°	66.355(3)
β/°	71.959(3)
γ/°	79.442(3)
Volume/Å^3	1371.71(9)
Z	2
ρ_{calc}g/cm^3	1.854

**μ/mm^{-1}**	10.016
**F(000)**	748.0
**Crystal size/mm^3**	0.105 × 0.086 × 0.038
**Radiation**	Cu Kα (λ = 1.54184)
**2Θ range for data collection/°**	7.46 to 136.5
**Index ranges**	-13 ≤ h ≤ 13, -13 ≤ k ≤ 13, -14 ≤ l ≤ 16
**Reflections collected**	25320
**Independent reflections**	5007 [R_{int} = 0.0382, R_{sigma} = 0.0261]
**Data/restraints/parameters**	5007/0/379
**Goodness-of-fit on F^2**	1.088
**Final R indexes [I>=2σ (I)]**	R_1 = 0.0195, wR_2 = 0.0502
**Final R indexes [all data]**	R_1 = 0.0205, wR_2 = 0.0507
**Largest diff. peak/hole / e Å^{-3}**	0.89/-0.64

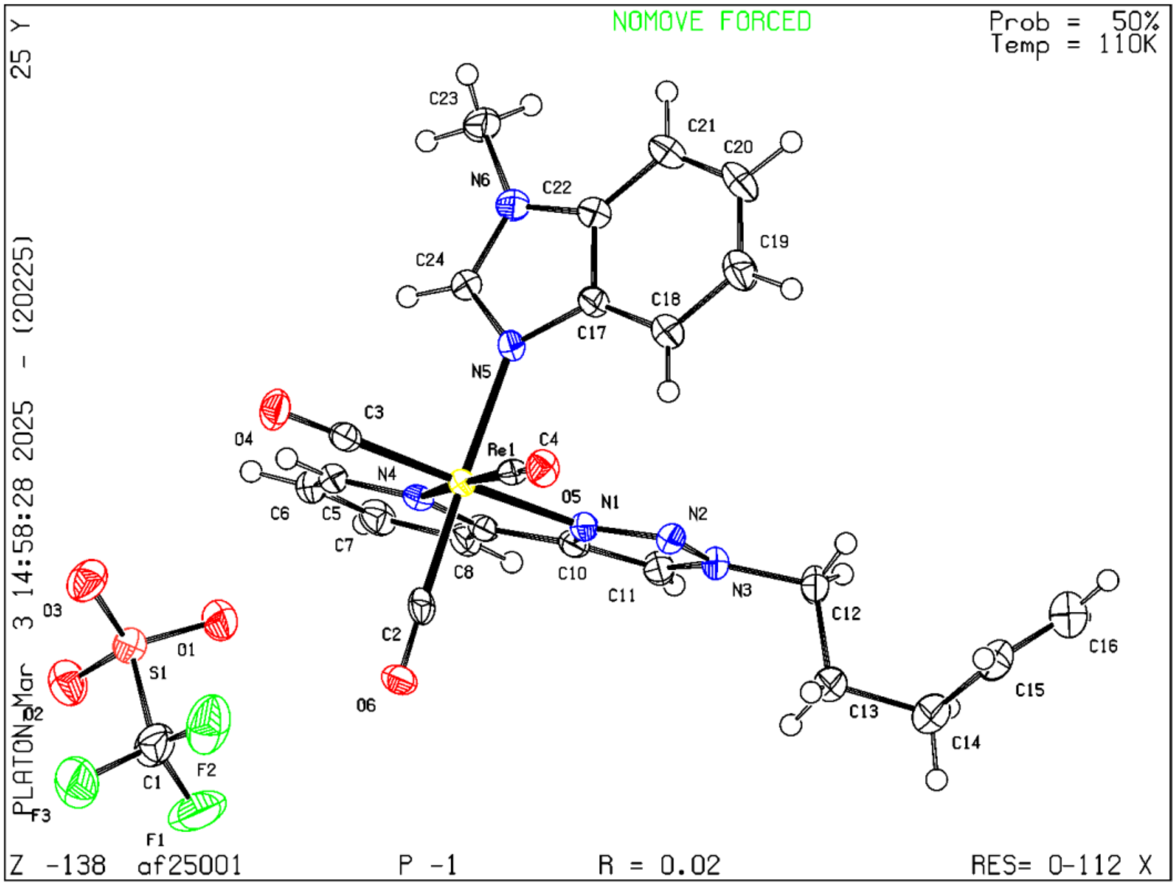

Figure S49: ORTEP view of Re(CO)$_3$(M1Y1) (thermal ellipsoids are set at 50% probability). CCDC deposition number 2455425

af25018: $Re(CO)_3$(M20Y3) (lab book ref. DRH-053), CCDC Deposition 2504788

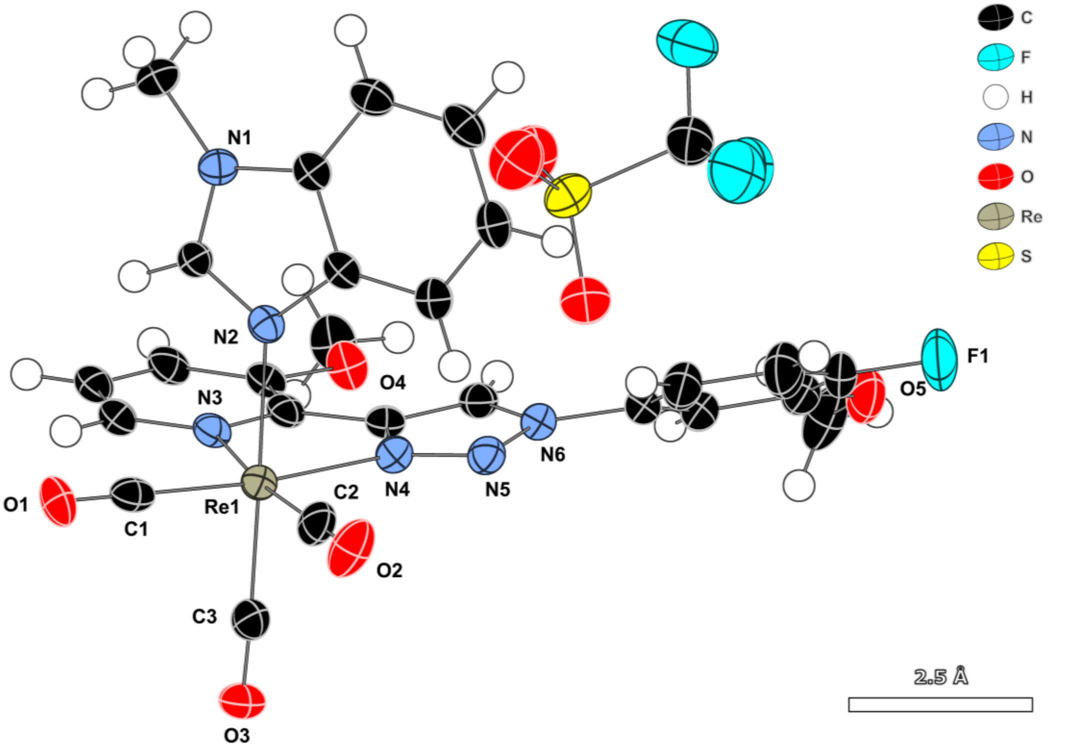

Figure S50: Structure, obtained by X-ray diffraction, of a single crystal of $Re(CO)_3$(M20Y3) (thermal ellipsoids are set at 50% probability). Selected interatomic lengths /Å: Re1-N2 = 2.193(2); Re1-N3 = 2.212(3); Re1-N4 = 2.151(3); Re1-C1 = 1.917(4); Re1-C2 = 1.916(4); Re1-C3 – 1.929(4). Selected interatomic angles /°: N3-Re1-N4 = 74.82(9); C1-Re1-C2 = 87.52(14).

Refinement special details

A solvent mask was calculated and 104 electrons were found in a volume of 414\%A^3^ in 1 void per unit cell. This is consistent with the presence of 0.6[C4H10O] per Asymmetric Unit which account for 101 electrons per unit cell.

Table S16: X-Ray Diffraction Data for $Re(CO)_3$(M20Y3)

Identification code	af25018
Empirical formula	$C_{29.4}H_{27}F_4N_6O_{8.6}ReS$
Formula weight	896.23
Temperature/K	109.97(19)
Crystal system	monoclinic
Space group	$P2_1/c$
a/Å	14.61900(10)
b/Å	15.07960(10)
c/Å	14.93150(10)
α/°	90

β/°	93.5490(10)
γ/°	90
Volume/Å^3	3285.32(4)
Z	4
ρ_{calc}g/cm^3	1.812
μ/mm^{-1}	8.581
F(000)	1765.0
Crystal size/mm^3	0.093 × 0.078 × 0.027
Radiation	Cu Kα (λ = 1.54184)
2Θ range for data collection/°	6.058 to 136.494
Index ranges	-17 ≤ h ≤ 17, -17 ≤ k ≤ 18, -17 ≤ l ≤ 16
Reflections collected	31051
Independent reflections	6023 [R_{int} = 0.0321, R_{sigma} = 0.0236]
Data/restraints/parameters	6023/0/427
Goodness-of-fit on F^2	1.024
Final R indexes [I>=2σ (I)]	R_1 = 0.0248, wR_2 = 0.0637
Final R indexes [all data]	R_1 = 0.0282, wR_2 = 0.0663
Largest diff. peak/hole / e Å^{-3}	1.10/-0.66

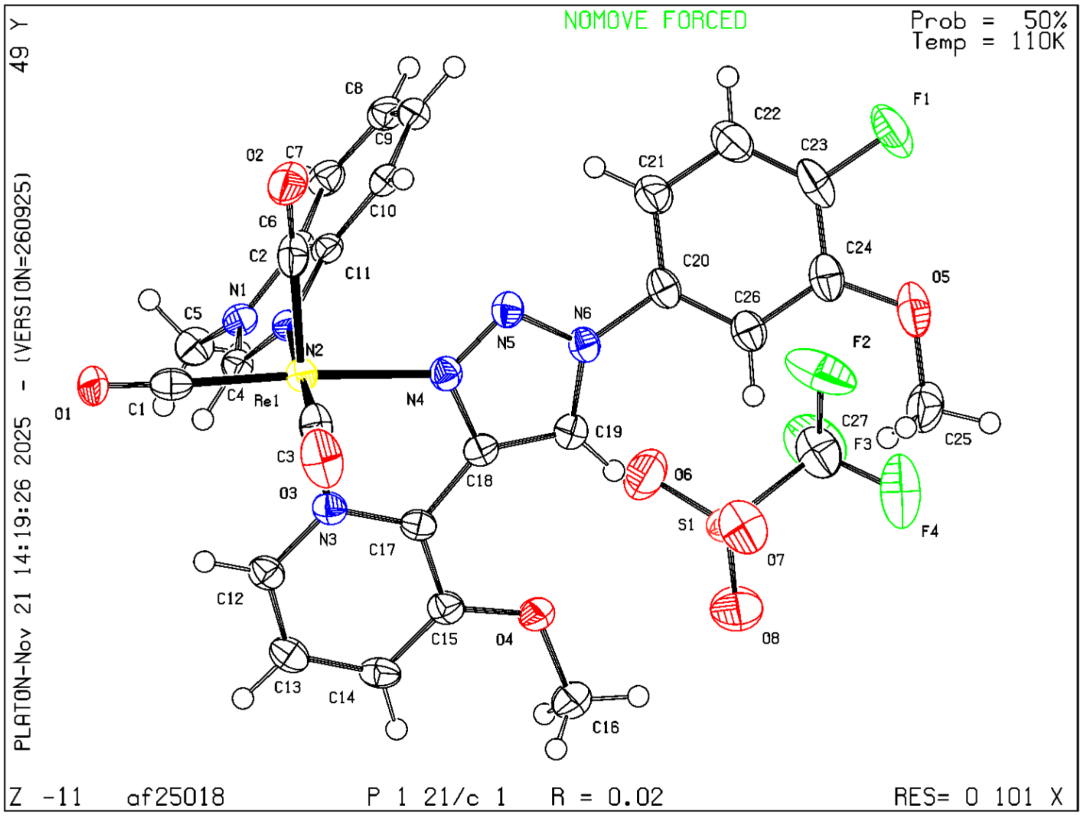

Figure S51: ORTEP view of Re(CO)$_3$(M20Y3) (thermal ellipsoids are set at 50% probability). CCDC deposition number 2504788

af25017: $Mn(CO)_3$(M19Y1) (lab book ref. DRH-068-1), CCDC Deposition 2504789

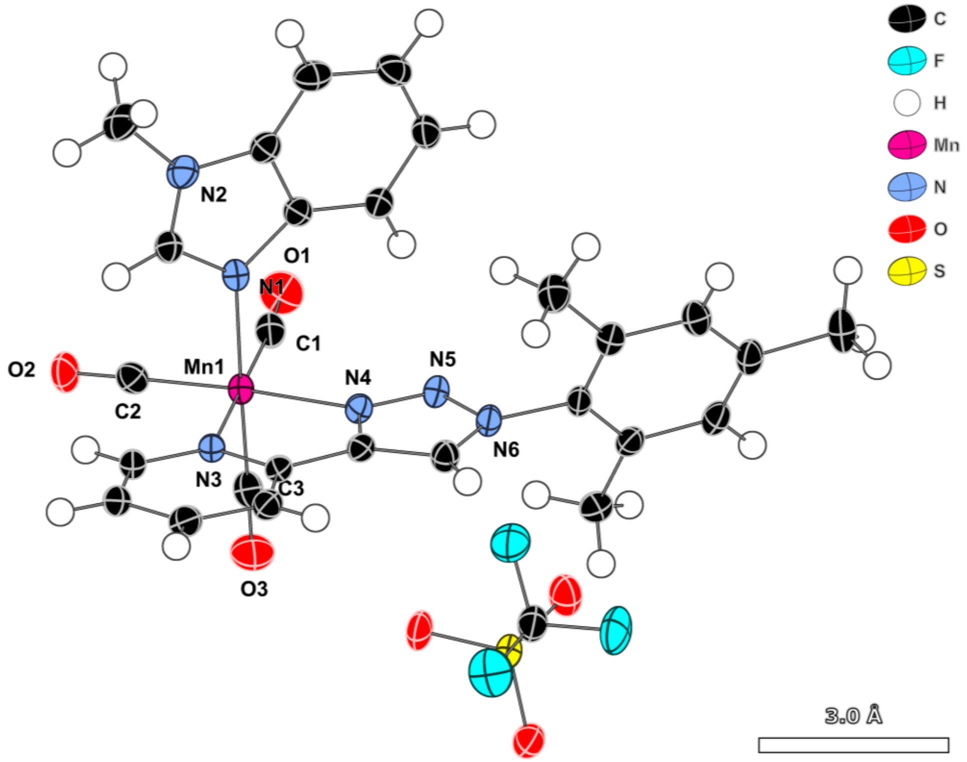

Figure S52: Structure, obtained by X-ray diffraction, of a single crystal of $Mn(CO)_3$(M19Y1) (thermal ellipsoids are set at 50% probability). Selected interatomic lengths /Å: Mn1-N1 = 2.0746(14); Mn1-N3 = 2.0704(13); Mn1-N4 = 2.0333(13); Mn1-C1 = 1.8156(18); Mn1-C2 = 1.8064(17); Mn1-C3 – 1.8142(18). Selected interatomic angles /°: N3-Mn1-N4 = 78.30(5); C1-Mn1-C2 = 85.79(7)

Table S17: X-Ray Diffraction Data for $Mn(CO)_3$(M19Y1)

Identification code	af25017
Empirical formula	$C_{28}H_{24}F_3MnN_6O_6S$
Formula weight	684.53
Temperature/K	109.97(16)
Crystal system	monoclinic
Space group	$P2_1/n$
a/Å	10.71900(10)
b/Å	14.4239(2)
c/Å	19.9546(2)
α/°	90
β/°	103.6210(10)
γ/°	90
Volume/Å^3	2998.40(6)
Z	4

ρ_{calc}g/cm^3	1.516
μ/mm^{-1}	4.864
F(000)	1400.0
Crystal size/mm^3	0.133 × 0.051 × 0.042
Radiation	Cu Kα (λ = 1.54184)
2Θ range for data collection/°	7.638 to 136.49
Index ranges	-12 ≤ h ≤ 12, -16 ≤ k ≤ 17, -24 ≤ l ≤ 24
Reflections collected	28045
Independent reflections	5488 [R_{int} = 0.0304, R_{sigma} = 0.0223]
Data/restraints/parameters	5488/0/410
Goodness-of-fit on F^2	1.045
Final R indexes [I>=2σ (I)]	R_1 = 0.0284, wR_2 = 0.0748
Final R indexes [all data]	R_1 = 0.0309, wR_2 = 0.0761
Largest diff. peak/hole / e Å^{-3}	0.45/-0.30

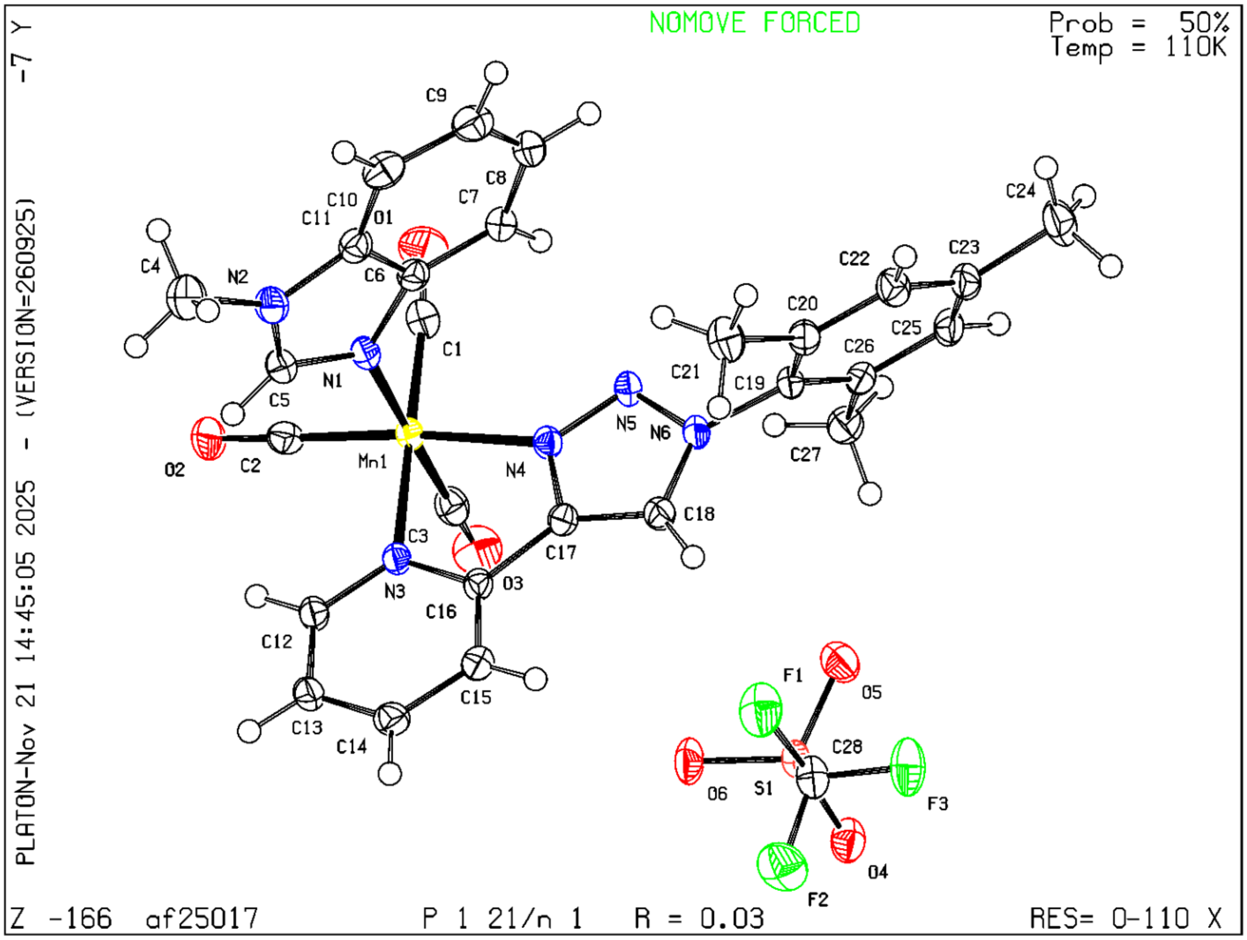

Figure S53: ORTEP view of Mn(CO)$_3$(M19Y1) (thermal ellipsoids are set at 50% probability). CCDC deposition number 2504789

6. NMR Spectral data for Organic and Inorganic Compounds

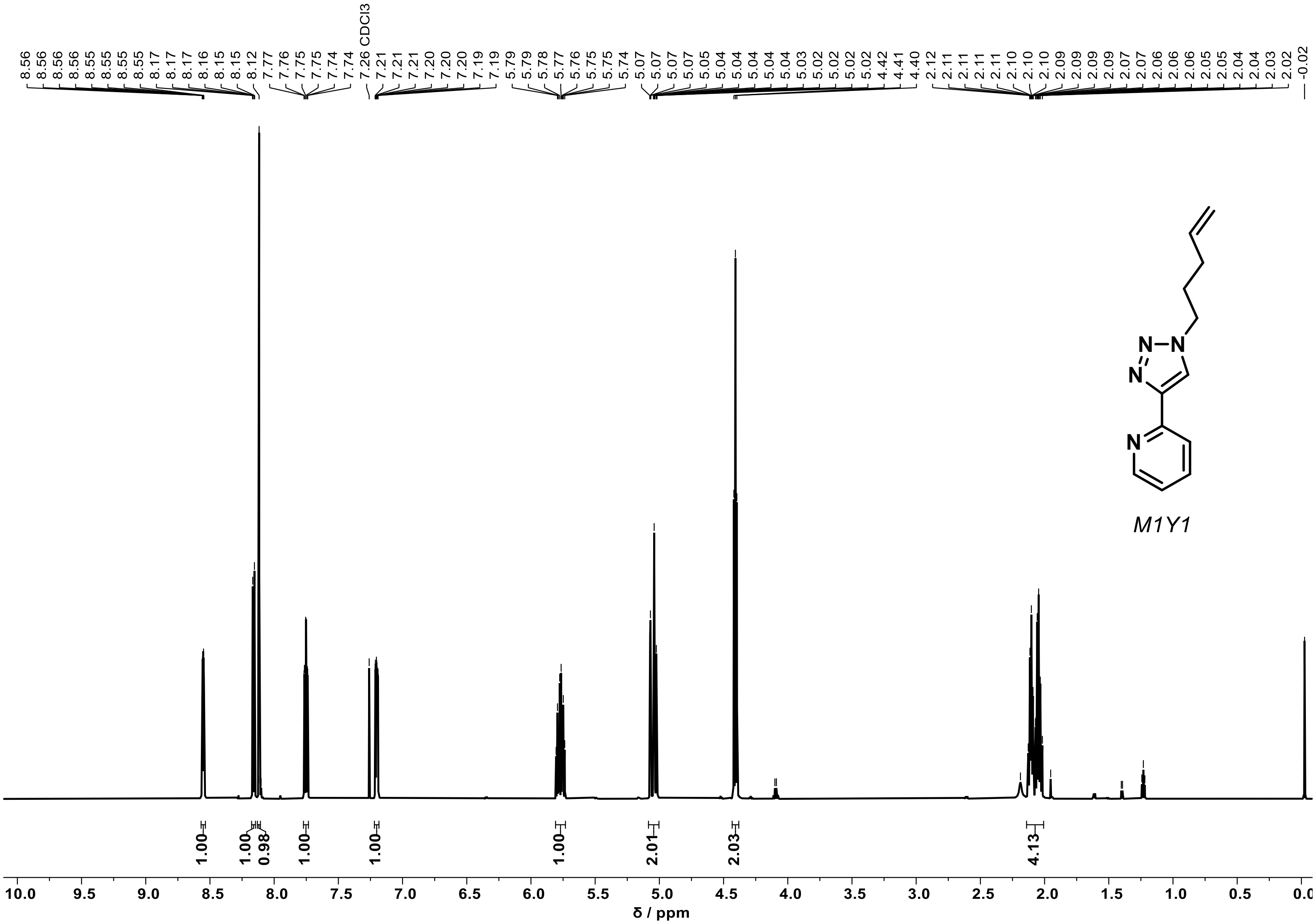

Figure S54: ^{1}H NMR (600 MHz, C*D*Cl$_3$, 32 scans, 10 s relaxation delay) spectrum of M1Y1. Lab book ref. DRH-051-1

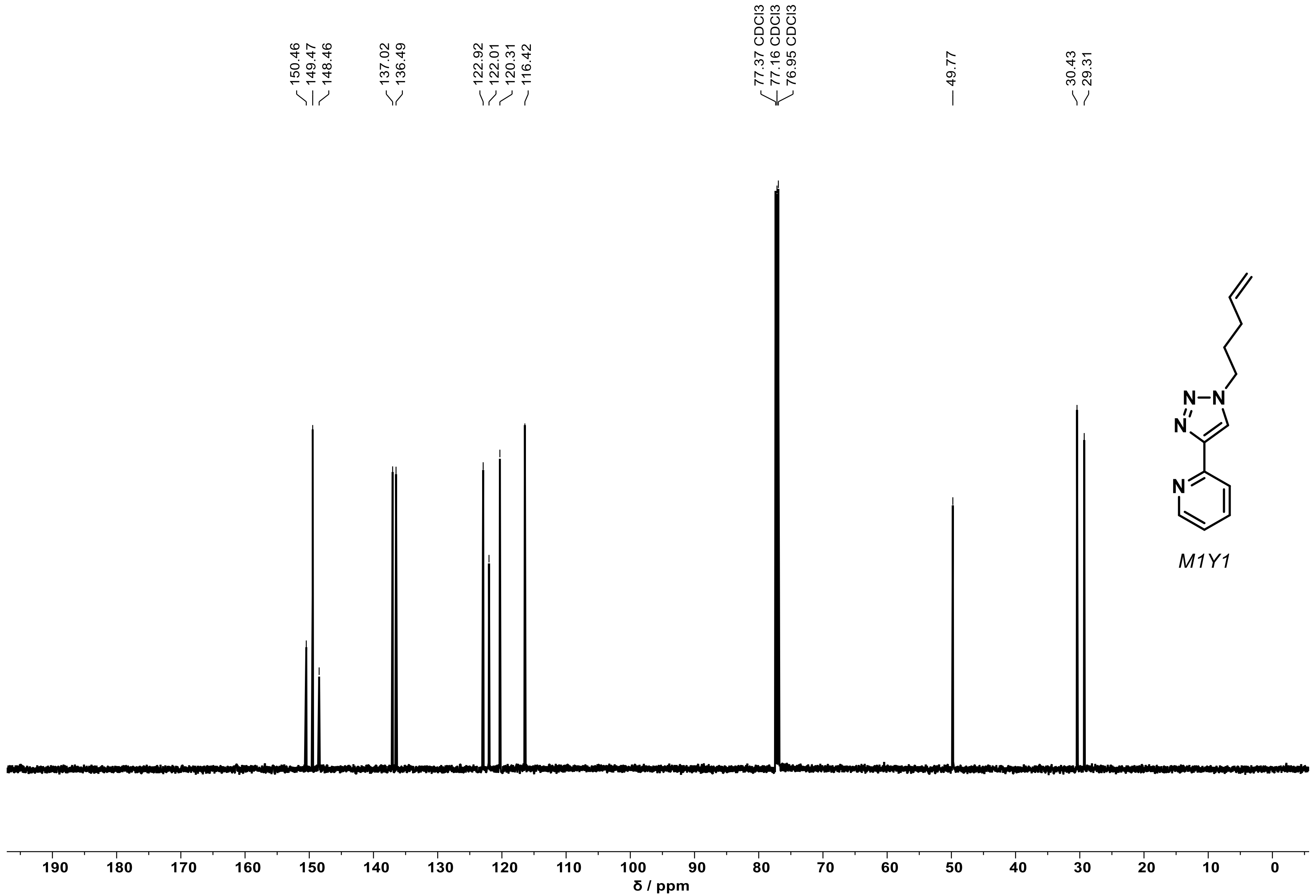

Figure S55: ^{13}C NMR (151 MHz, C*D*Cl_3, 128 scans) spectrum of the M1Y1. Lab book ref. DRH-051-1

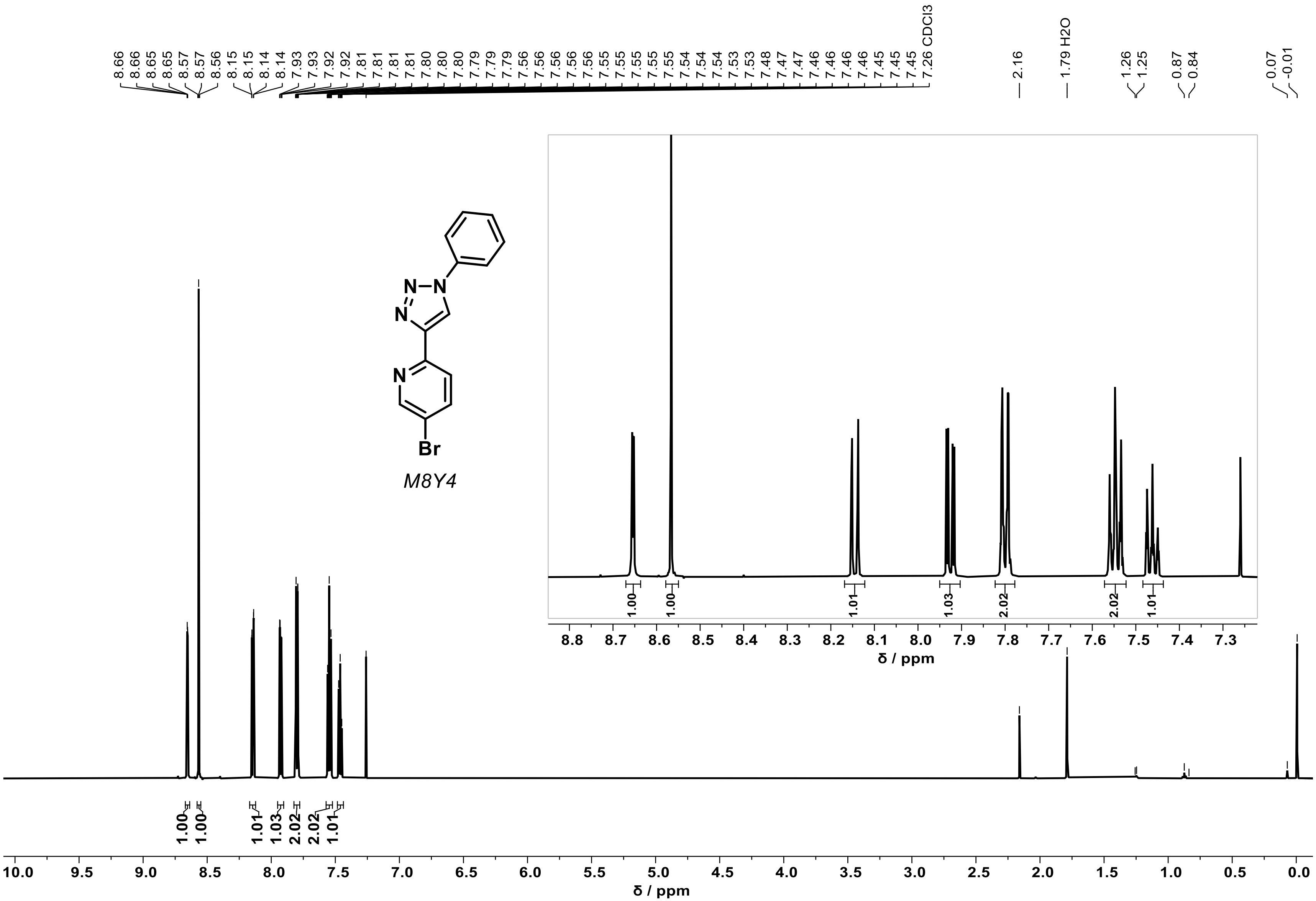

Figure S56: ^{1}H NMR (600 MHz, C*D*Cl$_3$, 32 scans, 10 s relaxation delay) spectrum of M8Y4. Lab book ref. DRH-051-2

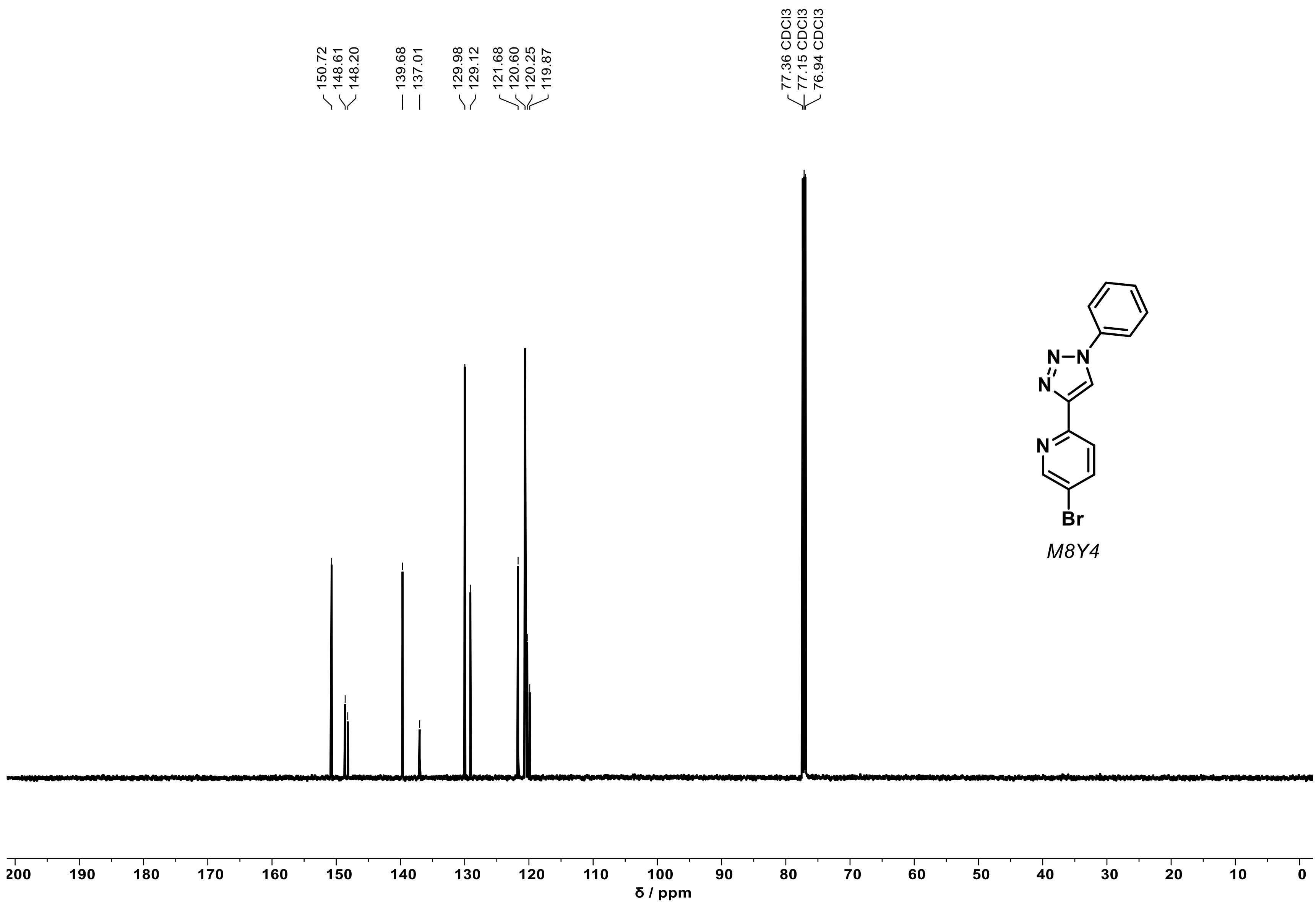

Figure S57: ^{13}C NMR (151 MHz, C*D*Cl$_3$, 256 scans) spectrum of the M8Y4. Lab book ref. DRH-051-2

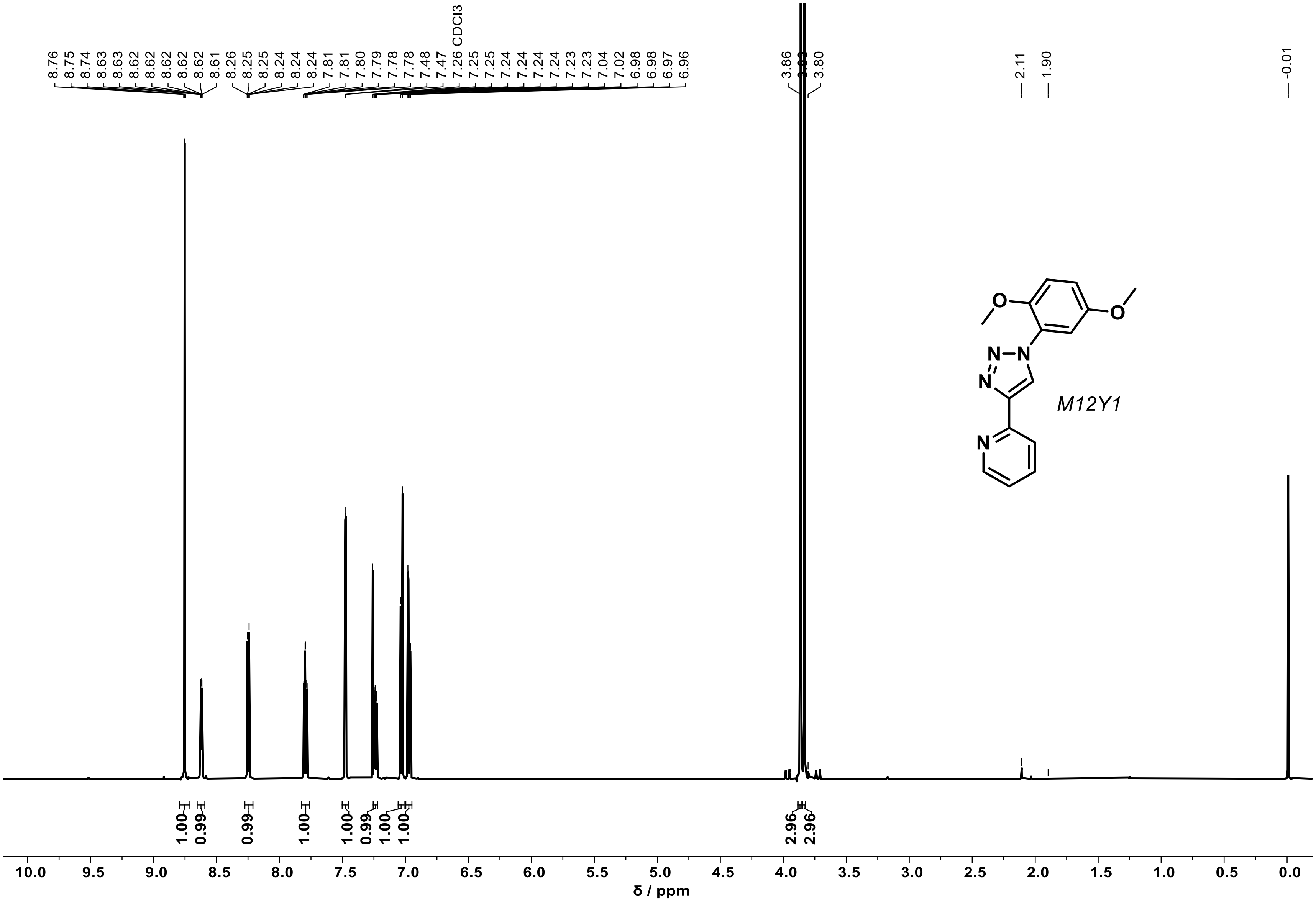

Figure S58: ^{1}H NMR (600 MHz, C*D*Cl$_3$, 16 scans, 10 s relaxation delay) spectrum of the M12Y1. Lab book ref. DRH-051-3

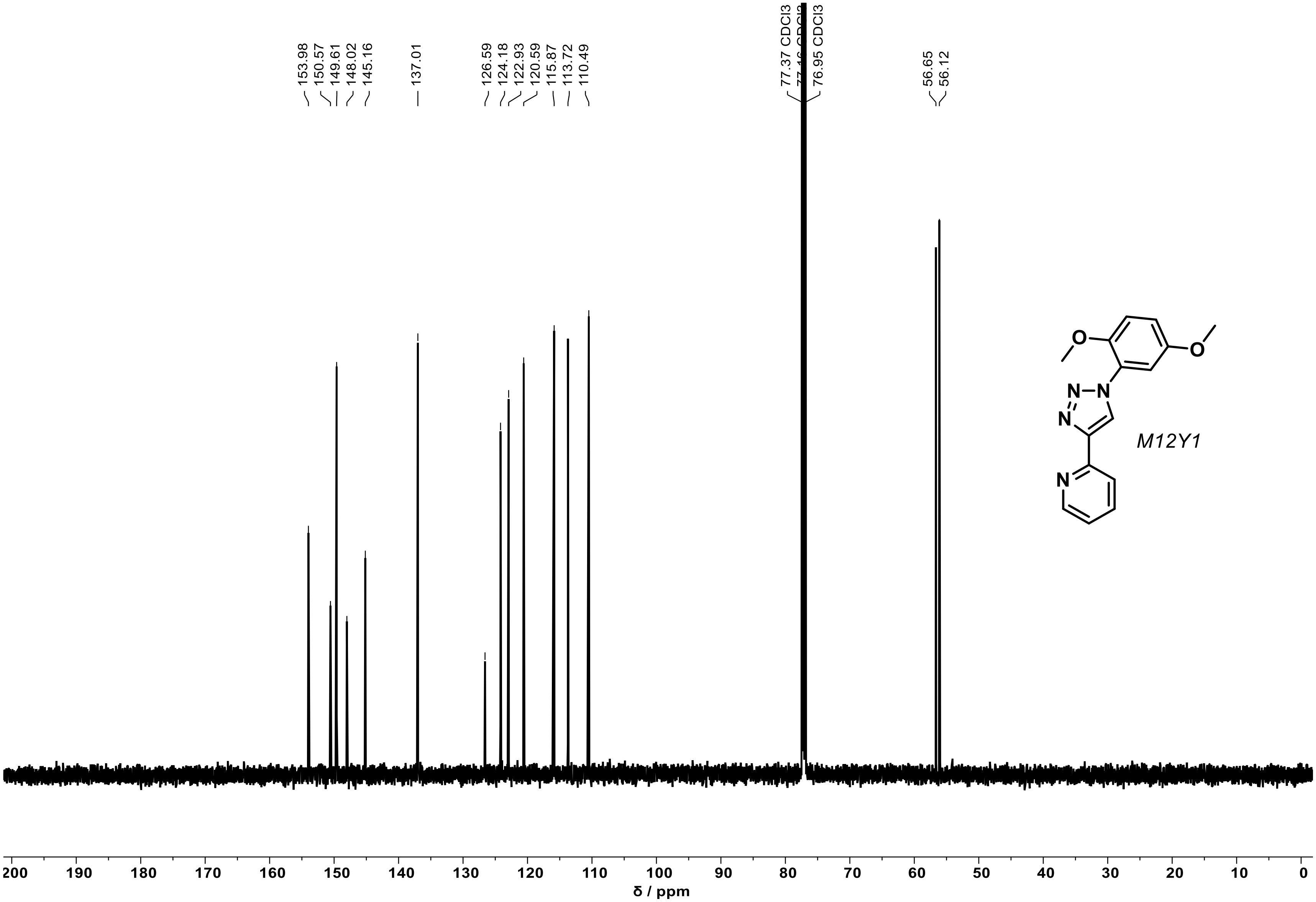

Figure S59: ^{13}C NMR (151 MHz, C*D*Cl$_3$, 128 scans) spectrum of M12Y1. Lab book ref. DRH-051-3

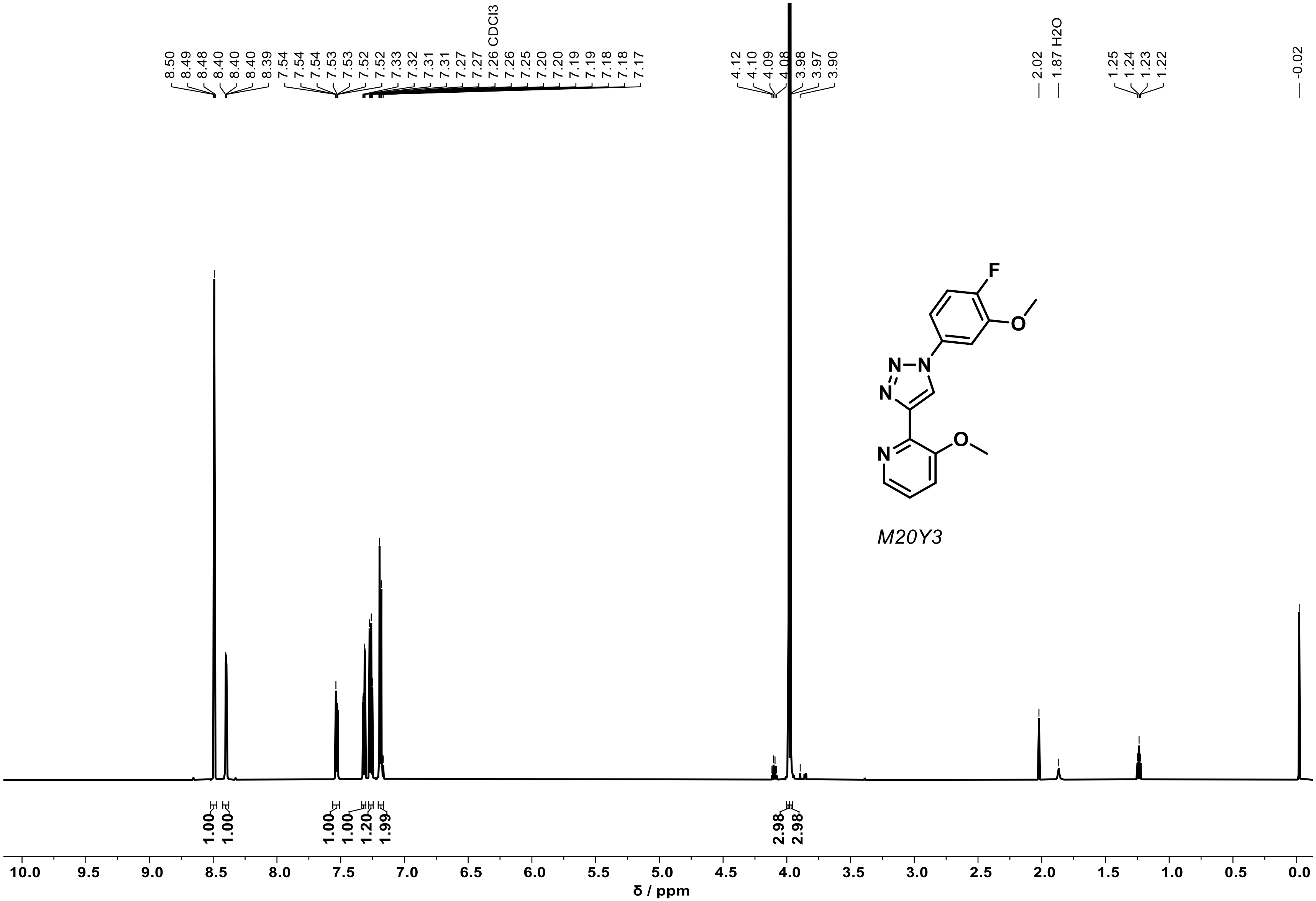

Figure S60: ^{1}H NMR (600 MHz, C*D*Cl$_3$, 16 scans, 10 s relaxation delay) spectrum of M20Y3. Lab book ref. DRH-051-5

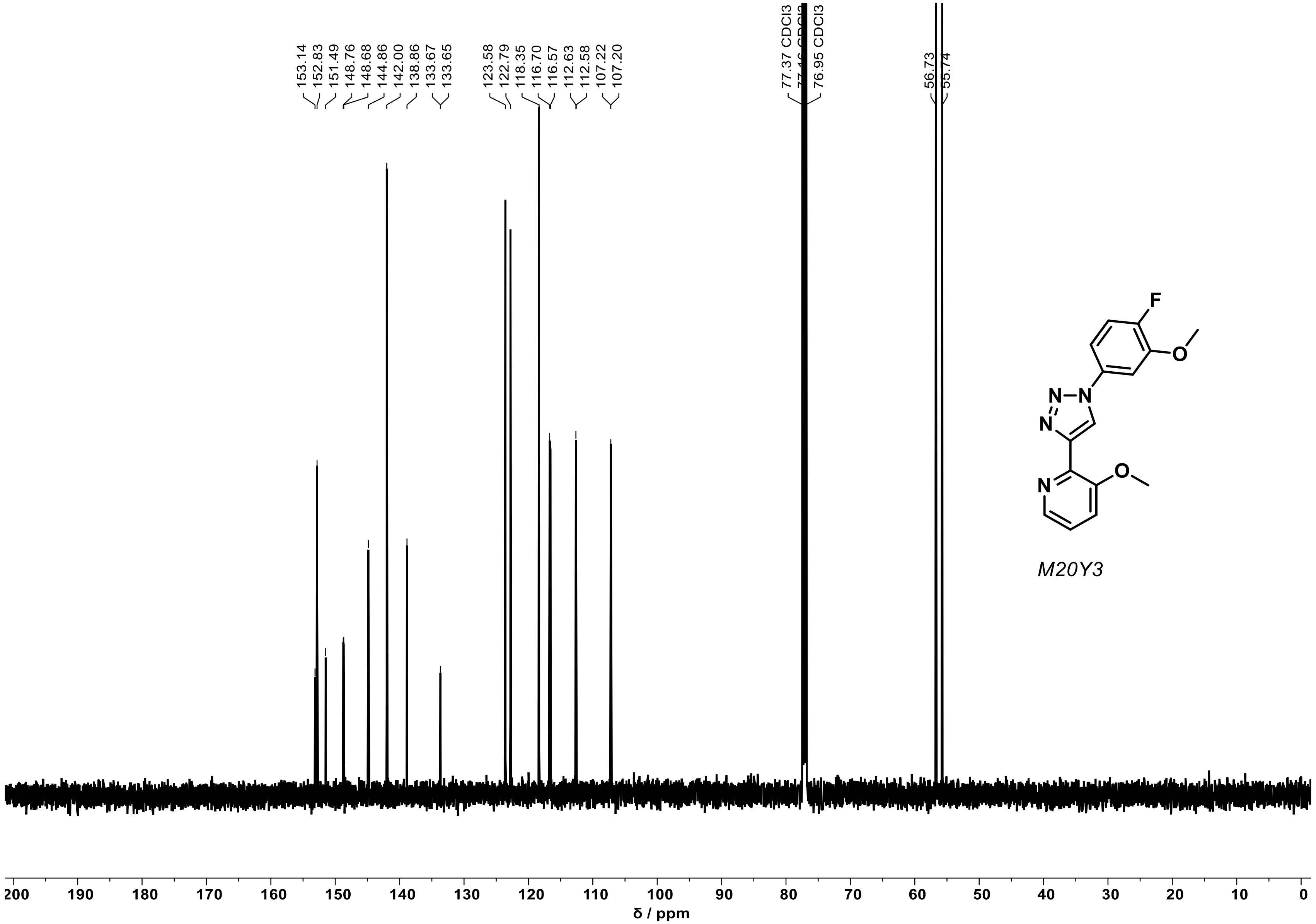

Figure S61: ^{13}C NMR (151 MHz, C*D*Cl$_3$, 128 scans) spectrum of M20Y3. Lab book ref. DRH-051-5

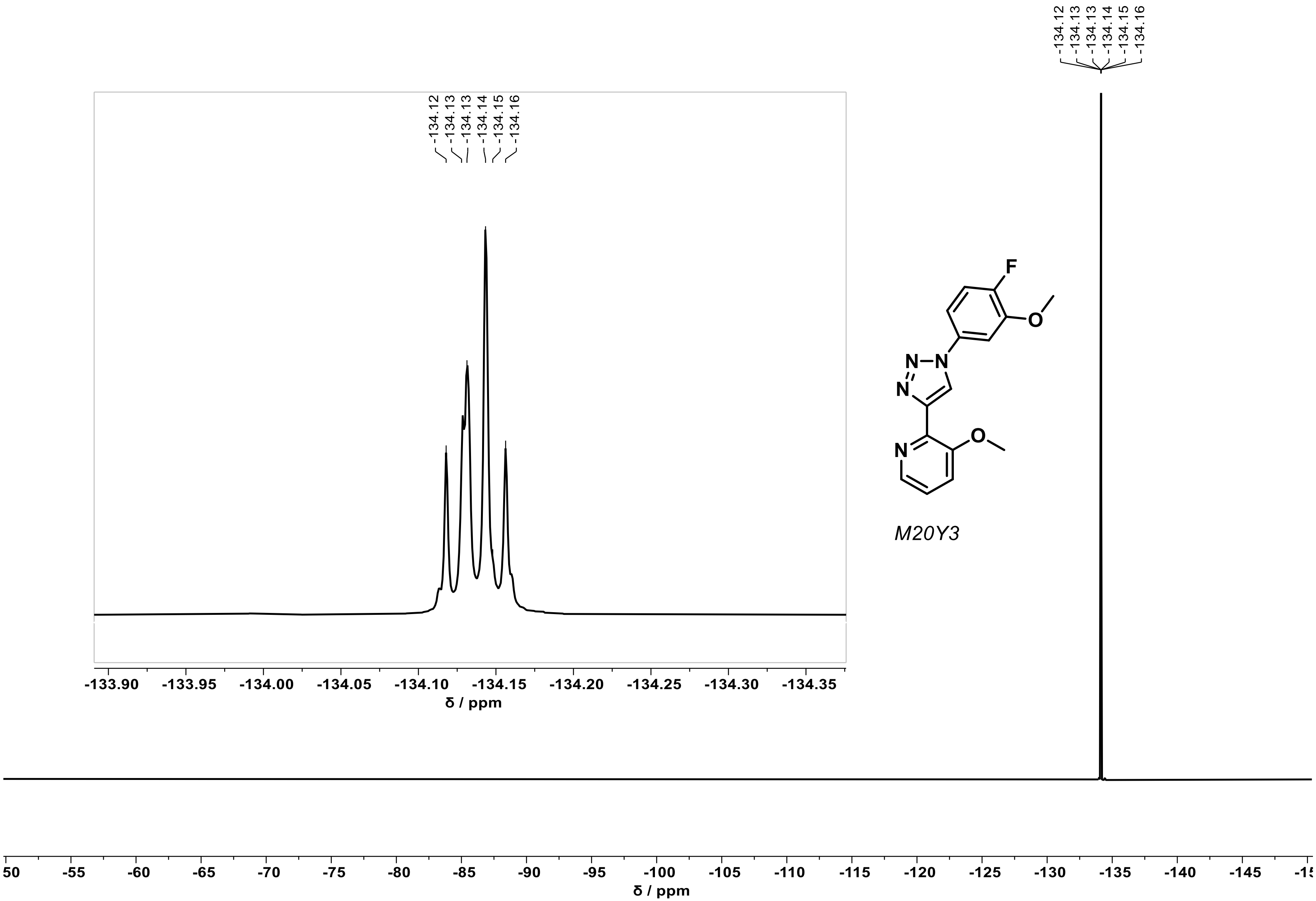

Figure S62: ^{19}F NMR (565 MHz, C*D*Cl$_3$, 32 scans) spectrum of M20Y3. Lab book ref. DRH-051-5

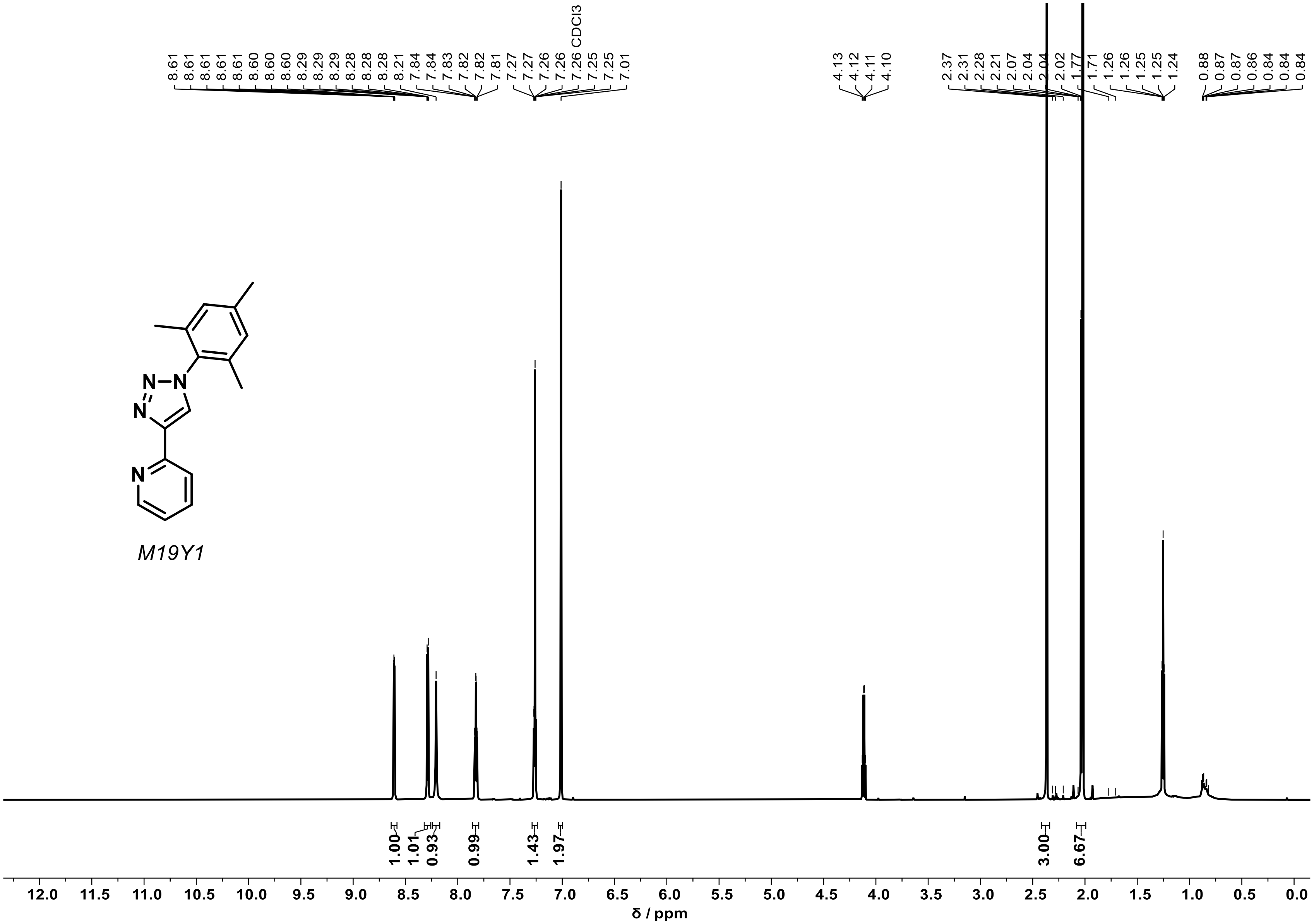

Figure S63: ^{1}H NMR (700 MHz, C*D*Cl$_3$, 16 scans, 12 s relaxation delay) spectrum of M19Y1. Lab book ref. DRH-066-1

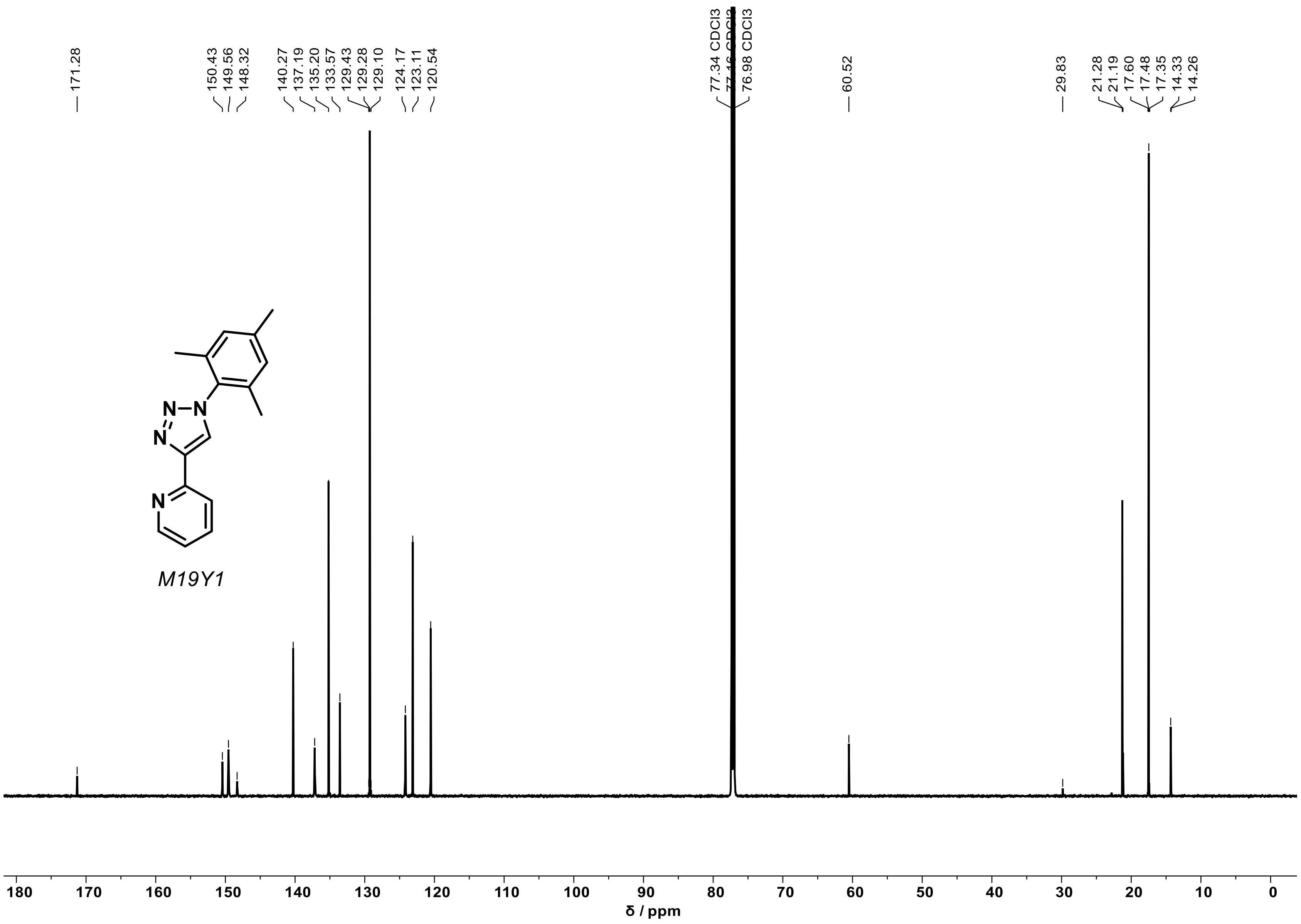

Figure S64: ^{13}C NMR (176 MHz, C*D*Cl$_3$, 2048 scans) spectrum of M19Y1. Lab book ref. DRH-066-1

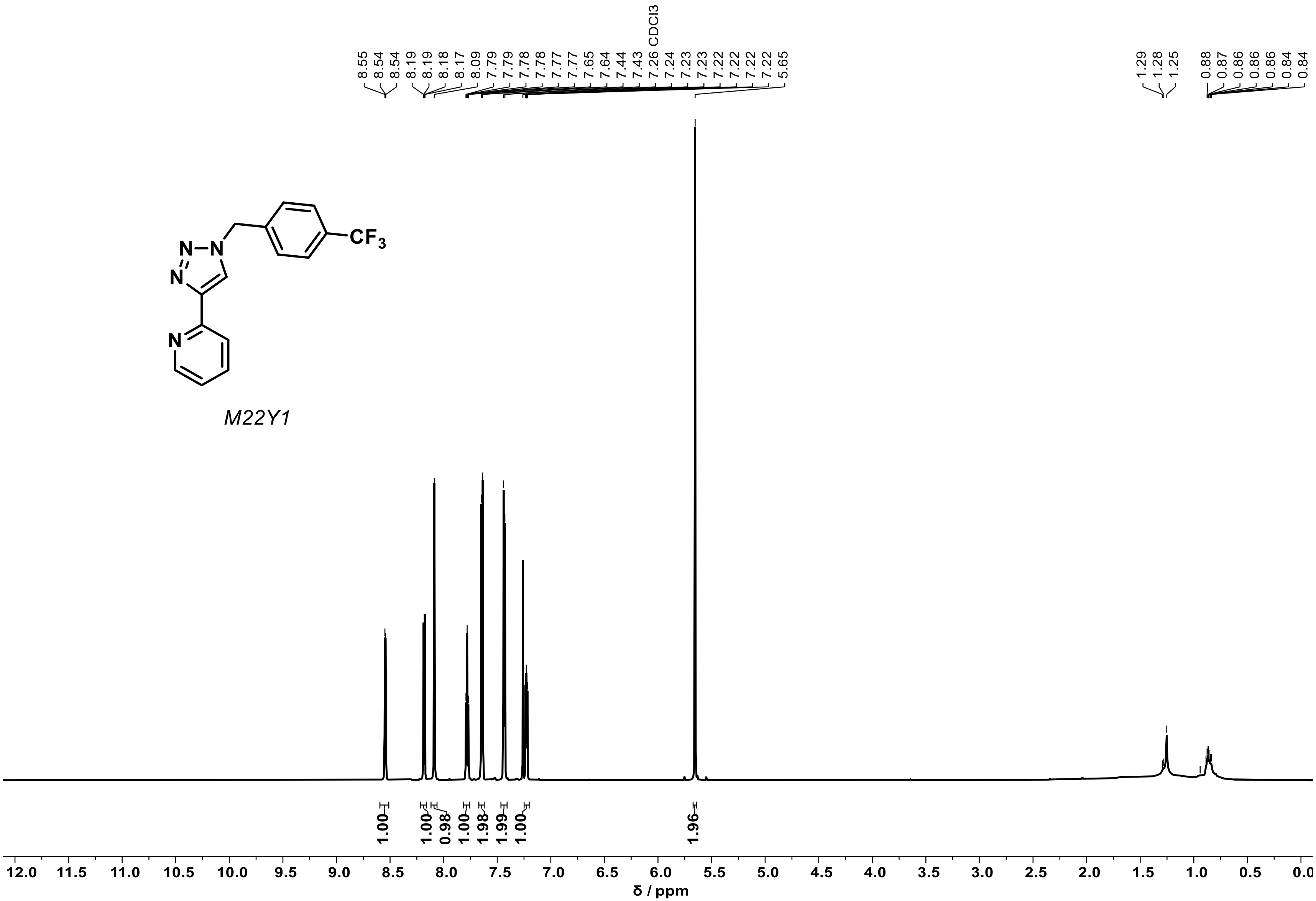

Figure S65: ^{1}H NMR (700 MHz, C*D*Cl$_3$, 16 scans, 12 s relaxation delay) spectrum of M22Y1. Lab book ref. DRH-066-2

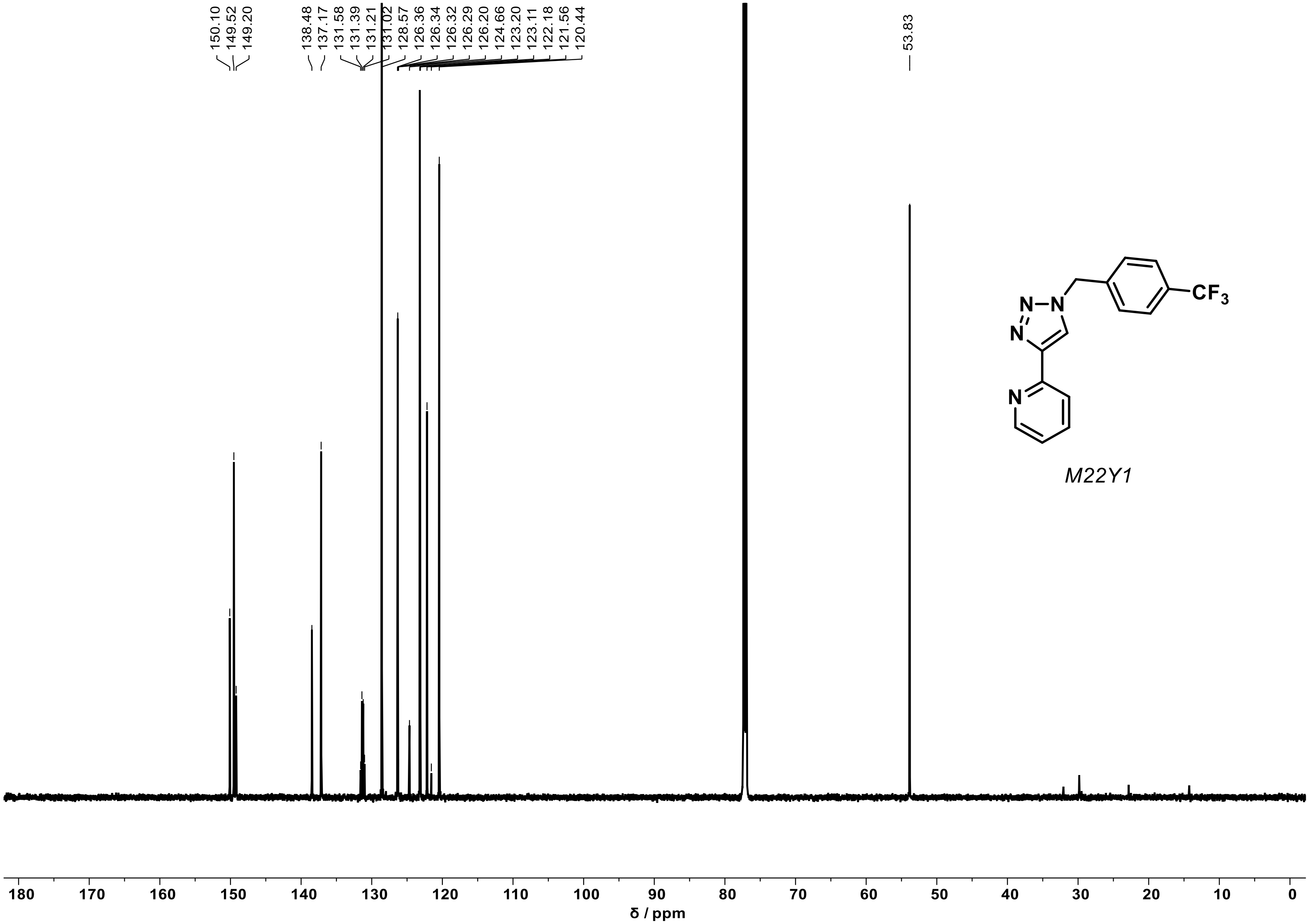

Figure S66: ^{13}C NMR (176 MHz, C*D*Cl₃, 2048 scans) spectrum of M22Y1. Lab book ref. DRH-066-2

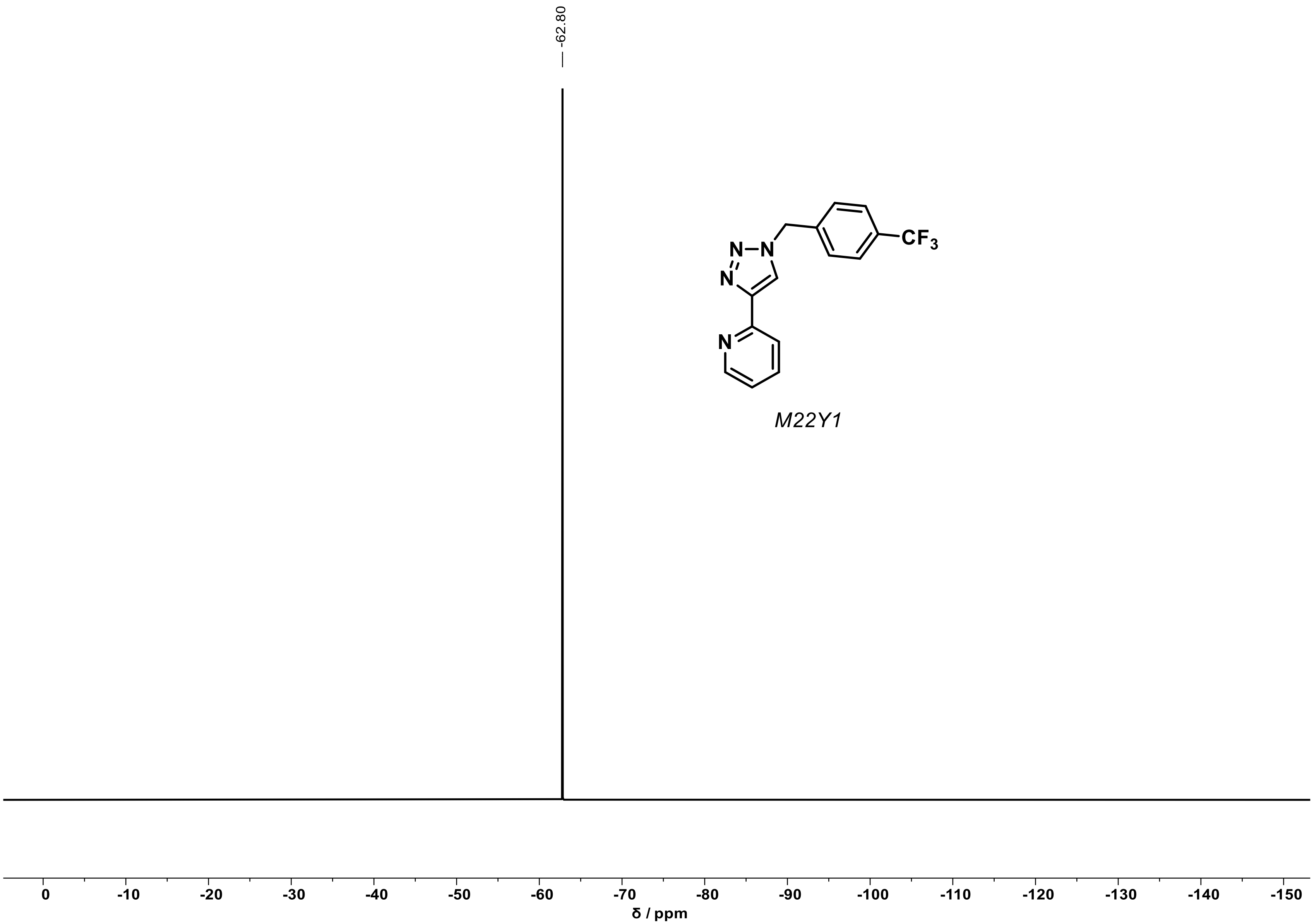

Figure S67: ^{19}F NMR (659 MHz, C*D*Cl$_3$, 64 scans) spectrum of M22Y1. Lab book ref. DRH-066-2

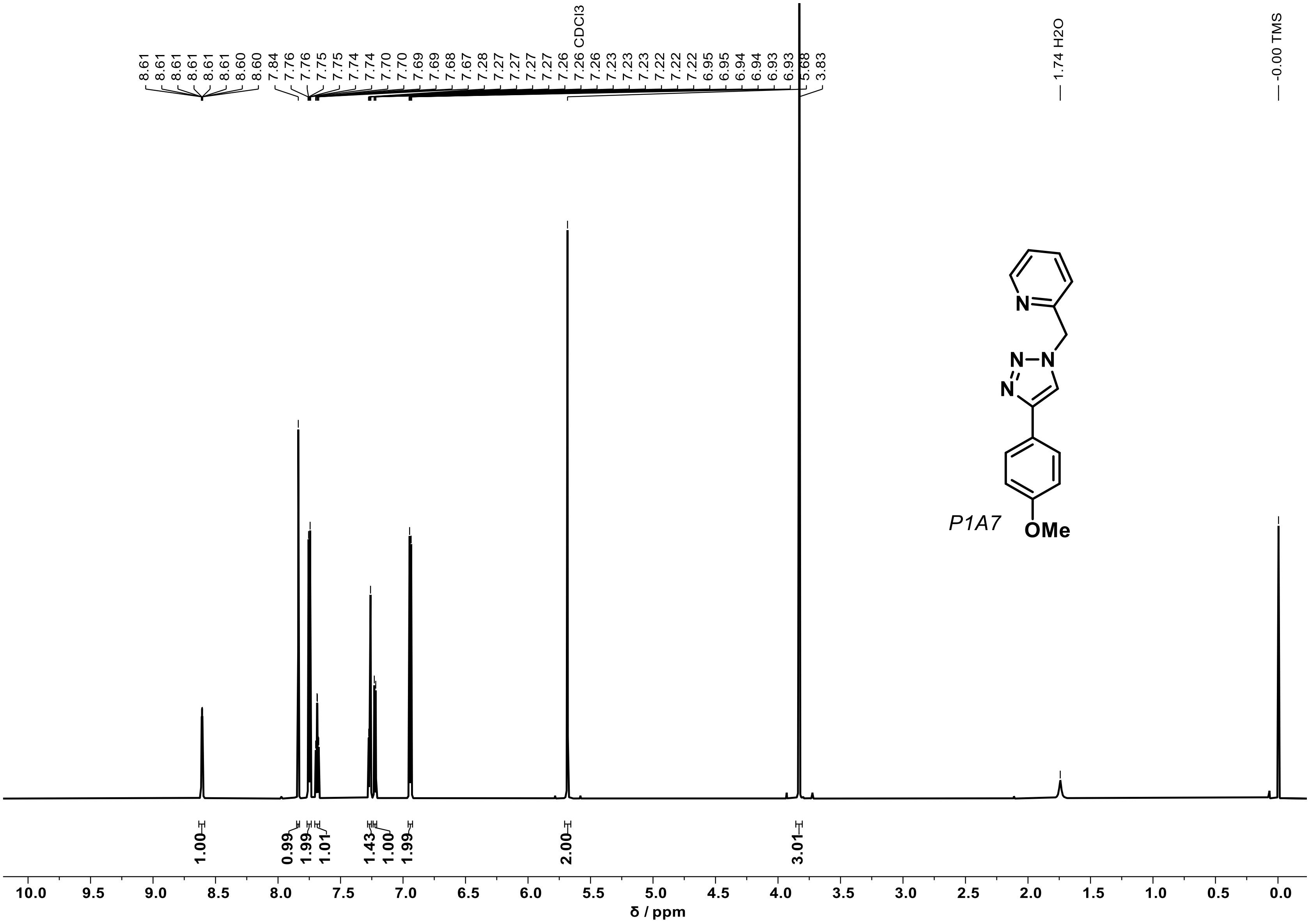

Figure S68: ^{1}H NMR (700 MHz, C*D*Cl_3, 32 scans, 12 s relaxation delay) spectrum of P1A7. Lab book ref. DRH-085-2

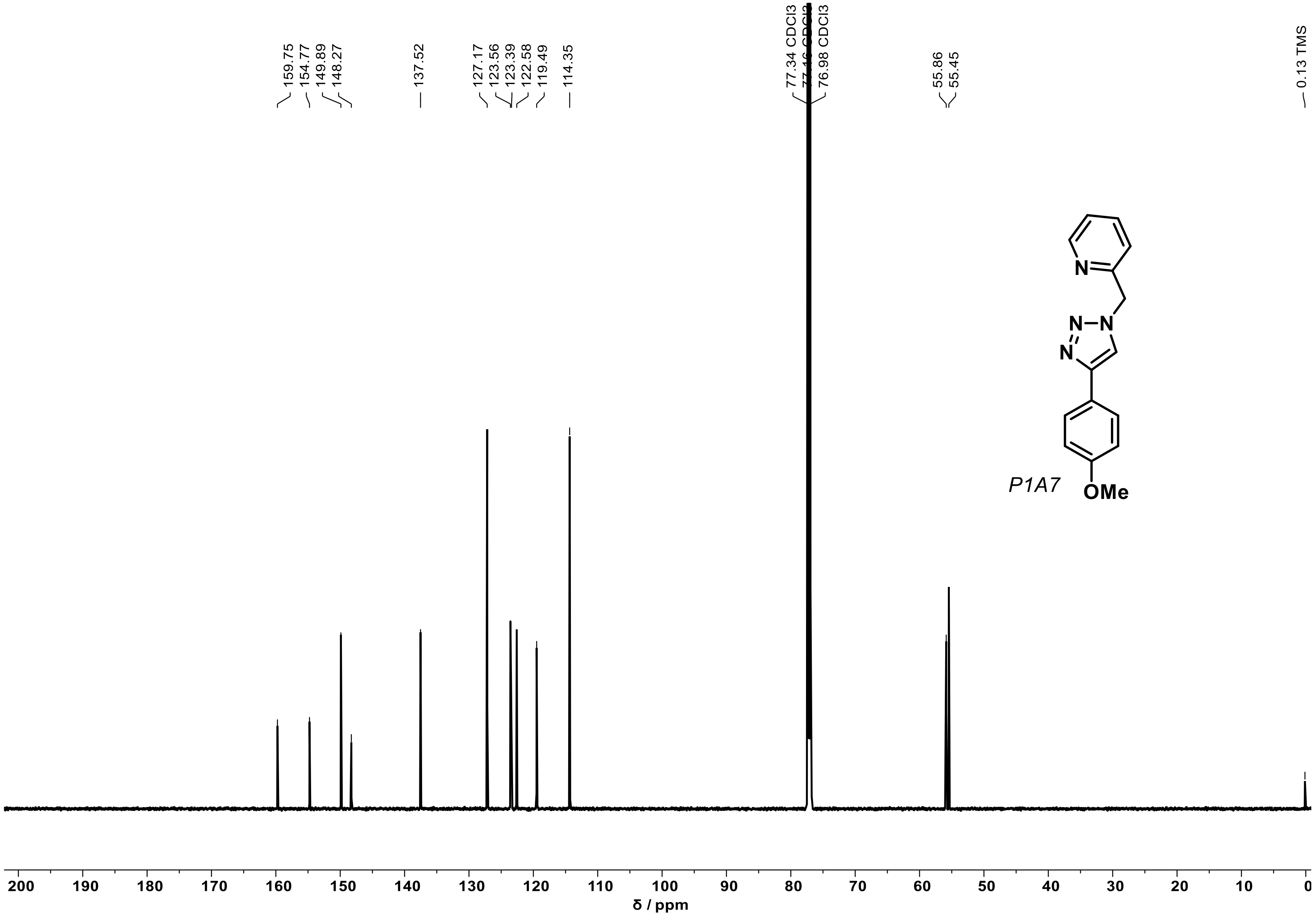

P1A7

Figure S69: ^{13}C NMR (176 MHz, C*D*Cl$_3$, 2048 scans) spectrum of P1A7. Lab book ref. DRH-085-2

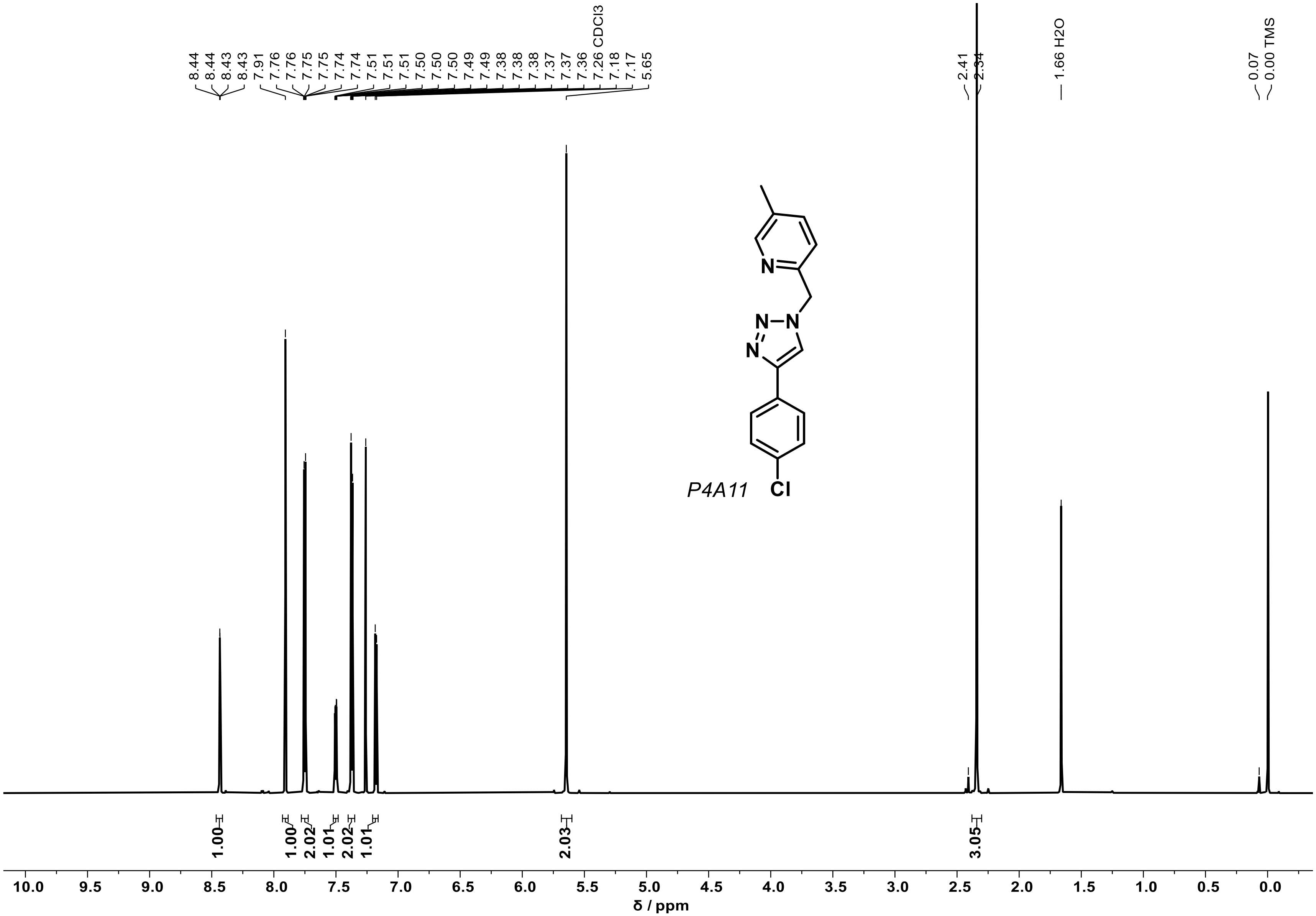

Figure S70: ^{1}H NMR (700 MHz, C*D*Cl$_3$, 32 scans, 12 s relaxation delay) spectrum of P4A11. Lab book ref. DRH-085-3

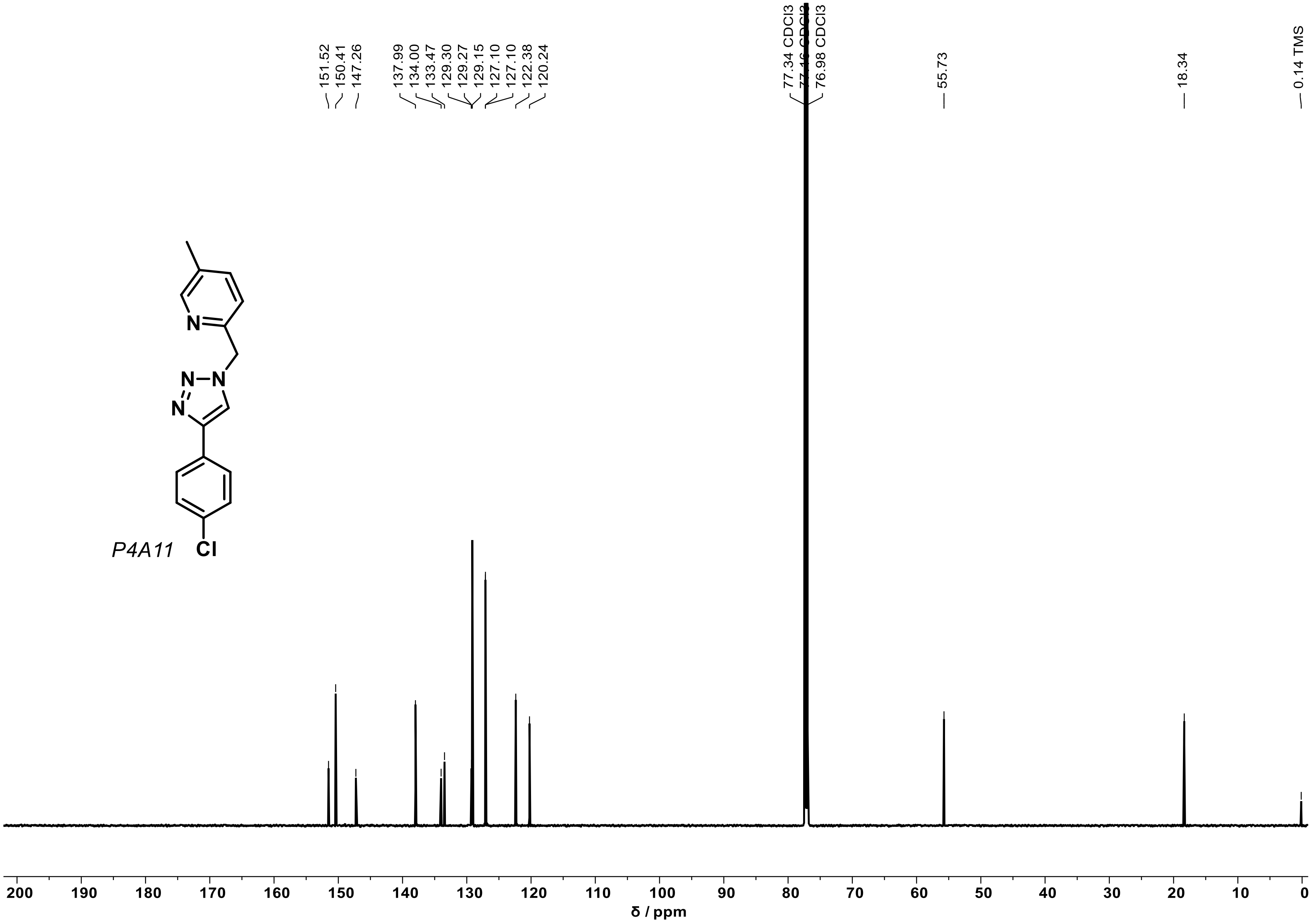

Figure S71: ^{13}C NMR (176 MHz, C*D*Cl$_3$, 2048 scans) spectrum of P4A11. Lab book ref. DRH-085-3

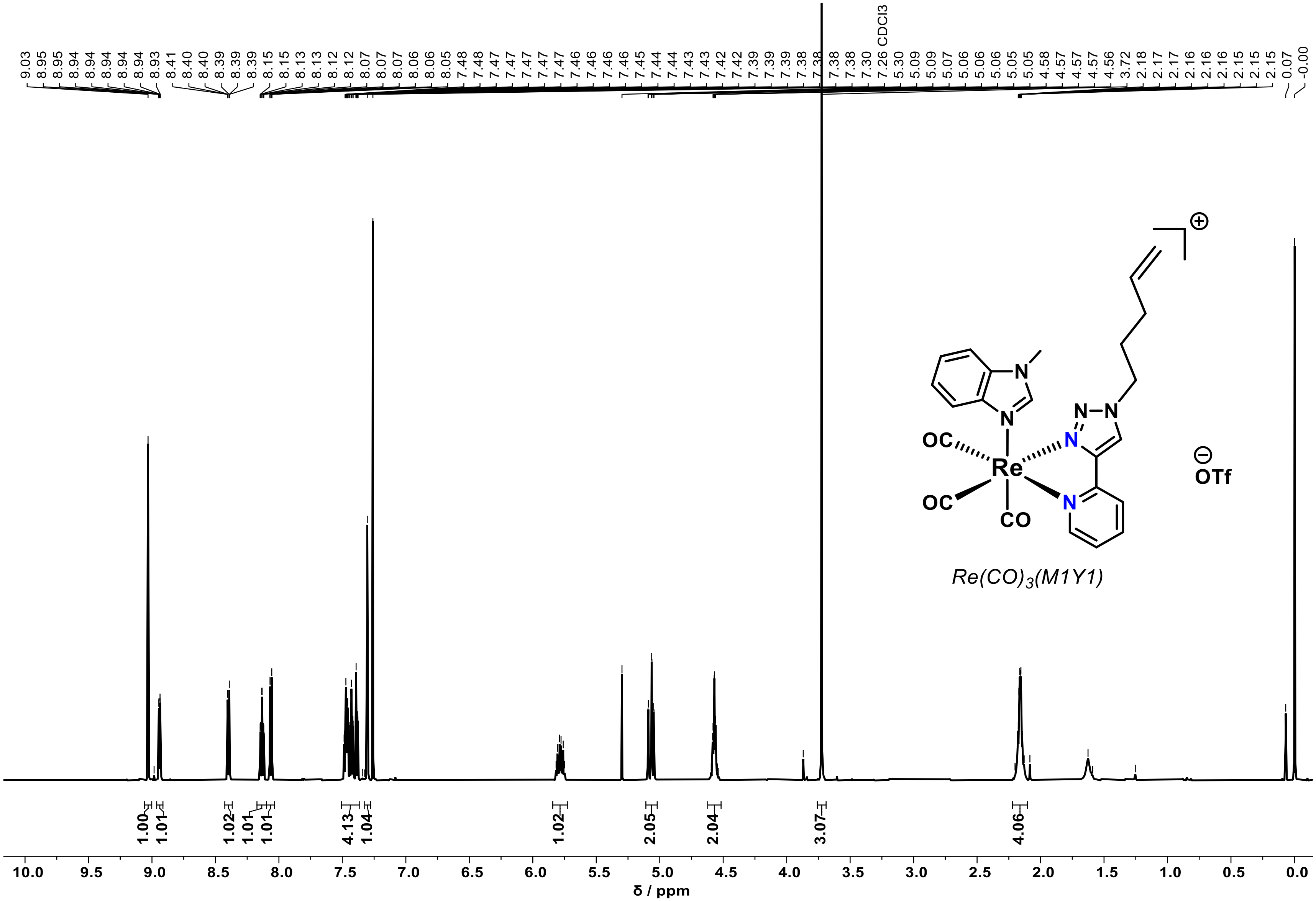

Figure S72: ^{1}H NMR (600 MHz, C*D*Cl$_3$, 16 scans, 10 s relaxation delay) spectrum of Re(CO)$_3$(M1Y1) . Lab book ref. DRH-052

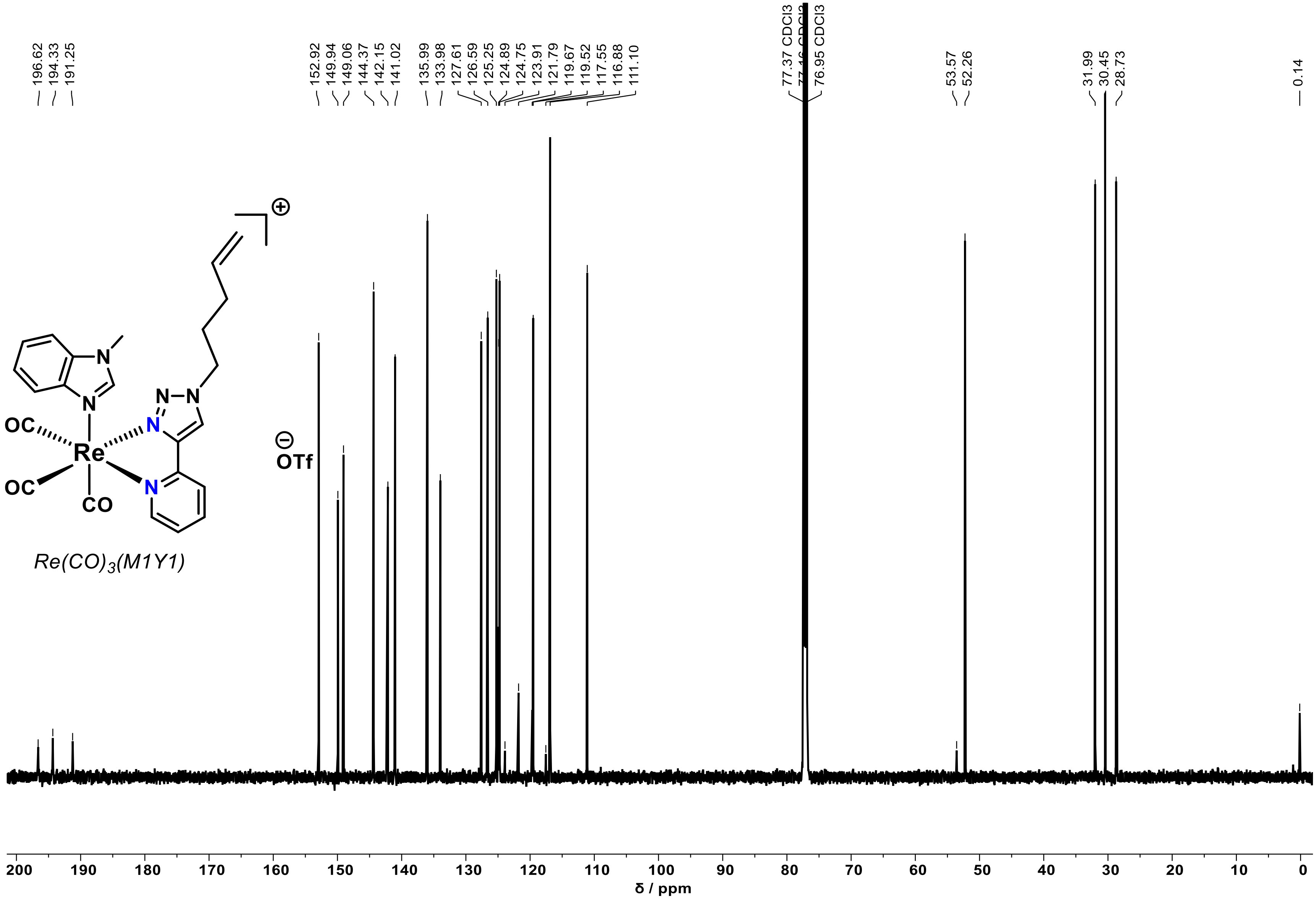

Figure S73: ^{13}C NMR (151 MHz, C*D*Cl$_3$, 16384 scans) spectrum of Re(CO)$_3$(M1Y1). Lab book ref. DRH-052

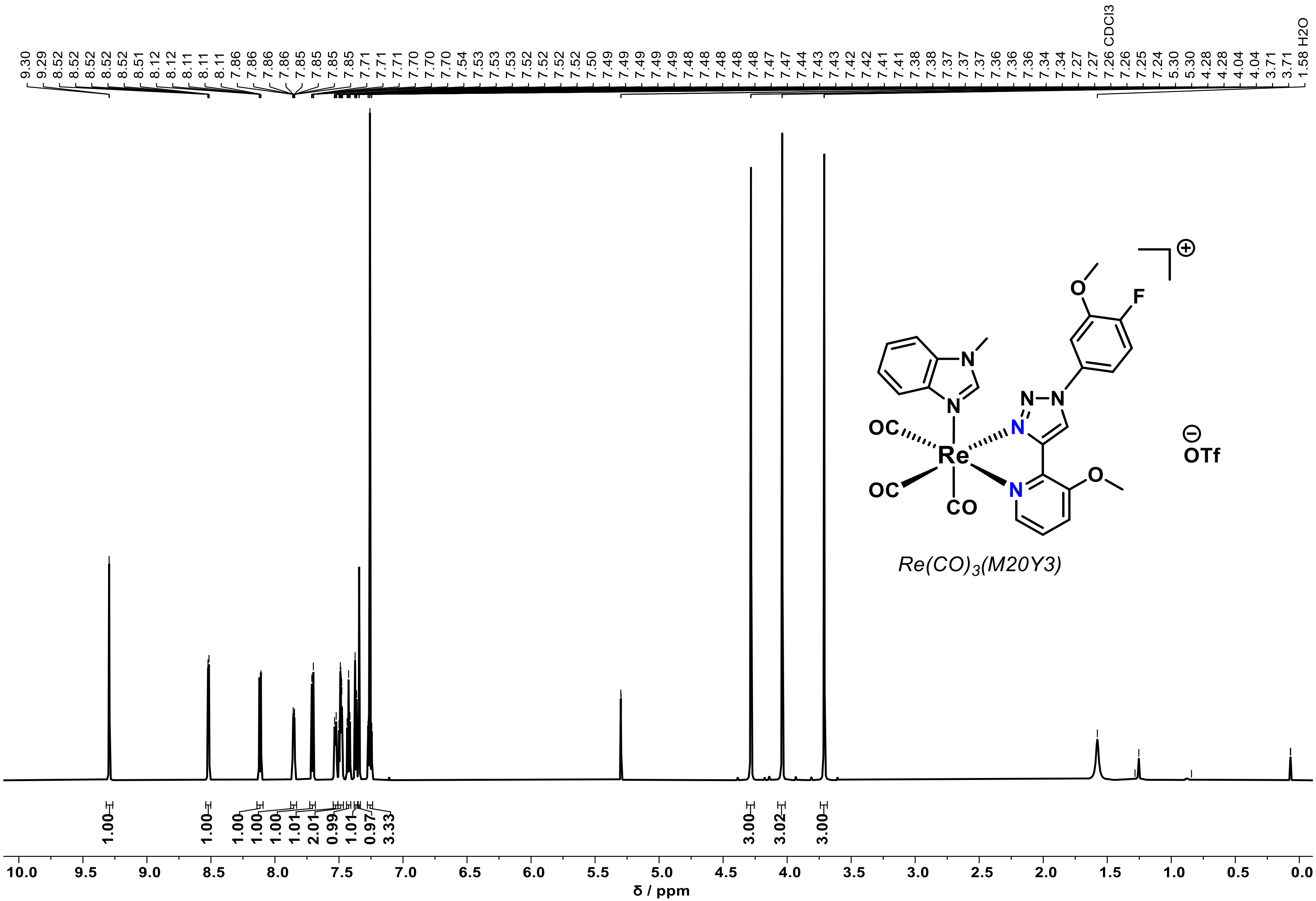

Figure S74: ^{1}H NMR (700 MHz, C*D*Cl$_3$, 32 scans, 12 s relaxation delay) spectrum of Re(CO)$_3$(M20Y3). Lab book ref. DRH-053

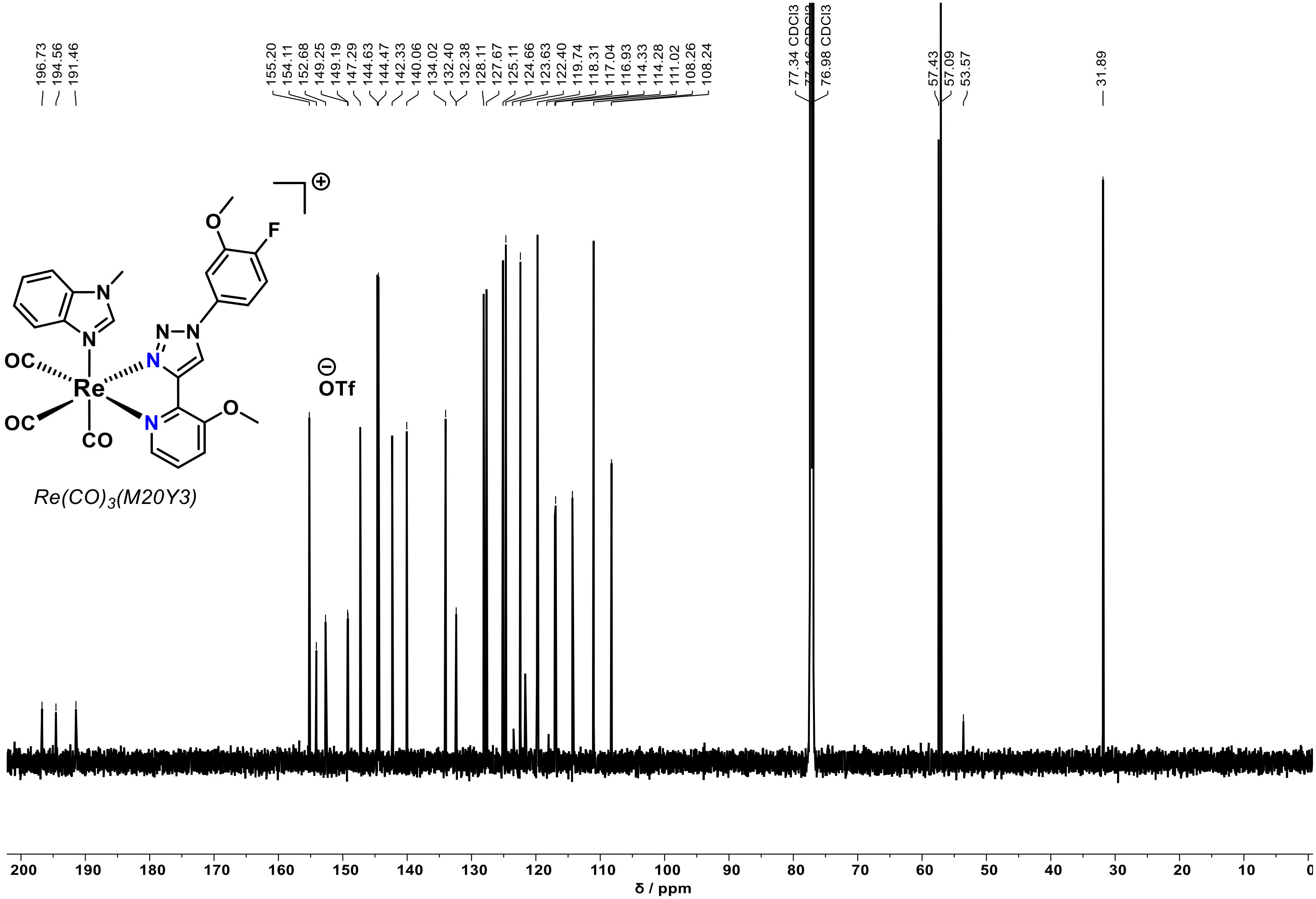

Figure S75: ^{13}C NMR (176 MHz, C*D*Cl$_3$, 3072 scans) spectrum of Re(CO)$_3$(M20Y3). Lab book ref. DRH-053

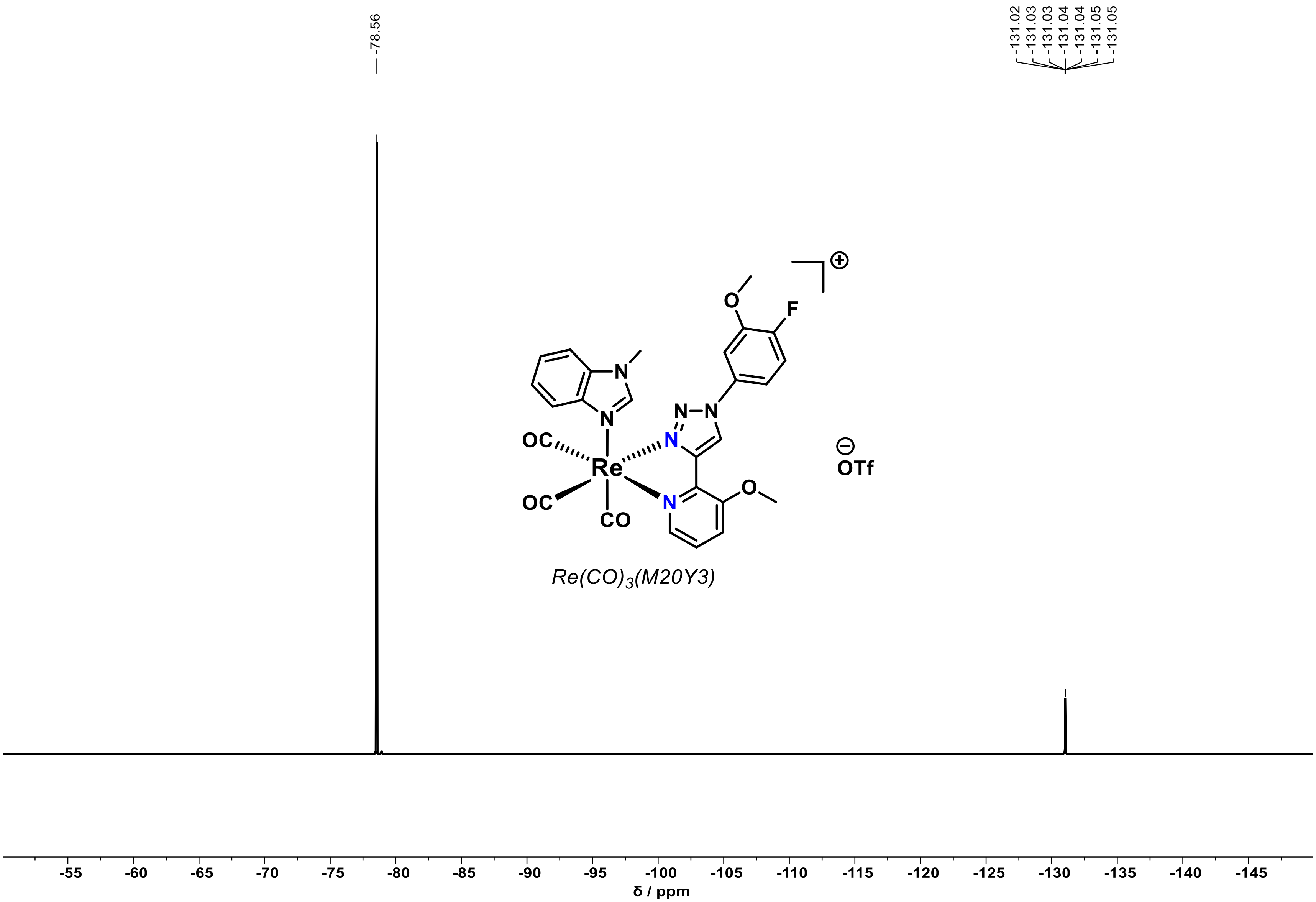

Figure S76: ^{19}F NMR (659 MHz, C*D*Cl_3, 128 scans) spectrum of Re(CO)$_3$(M20Y3). Lab book ref. DRH-053

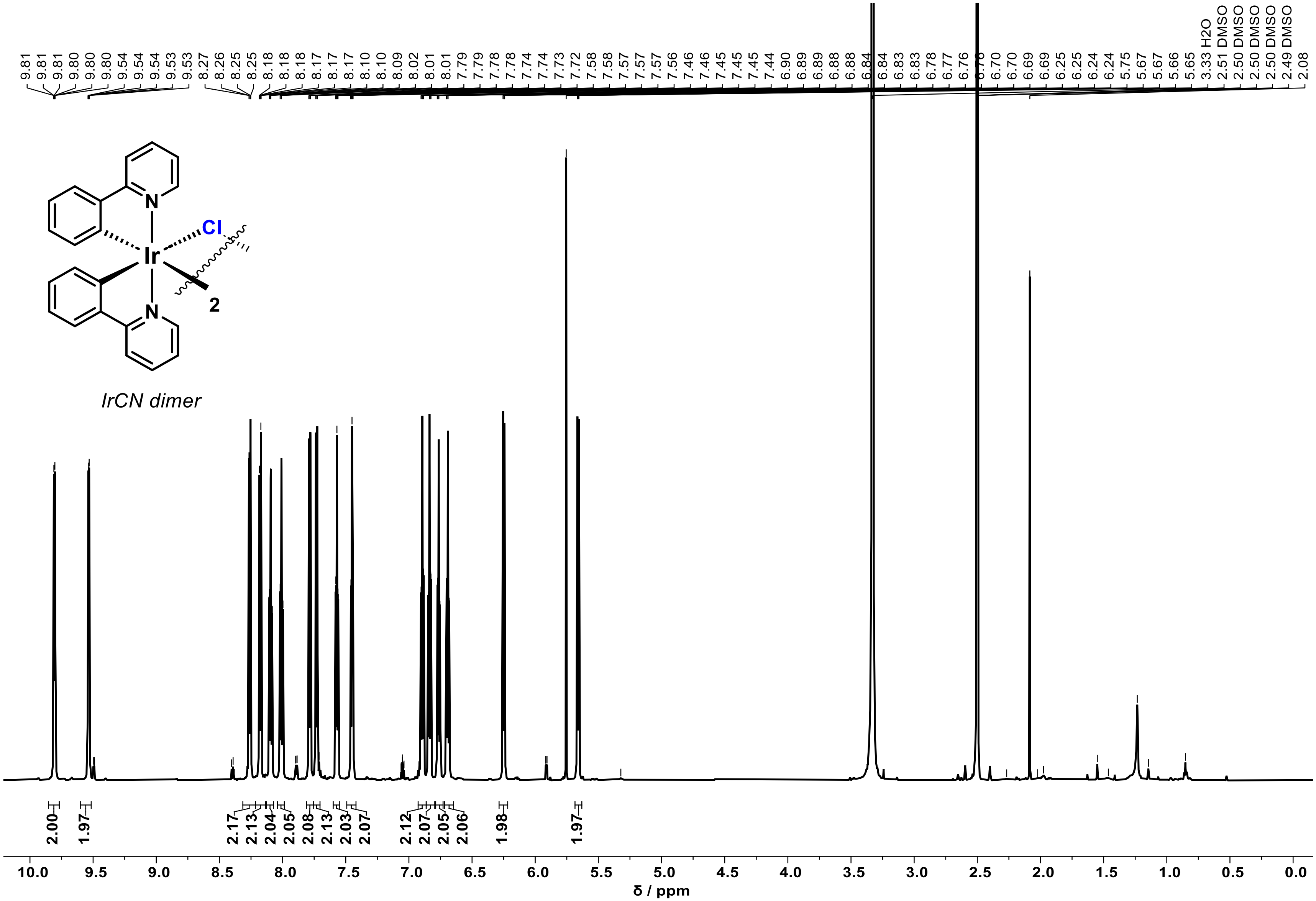

Figure S77: ^{1}H NMR (700 MHz, DMSO-d_6, 16 scans, 1 s relaxation delay) spectrum of IrCN dimer. Lab book ref. DRH-055

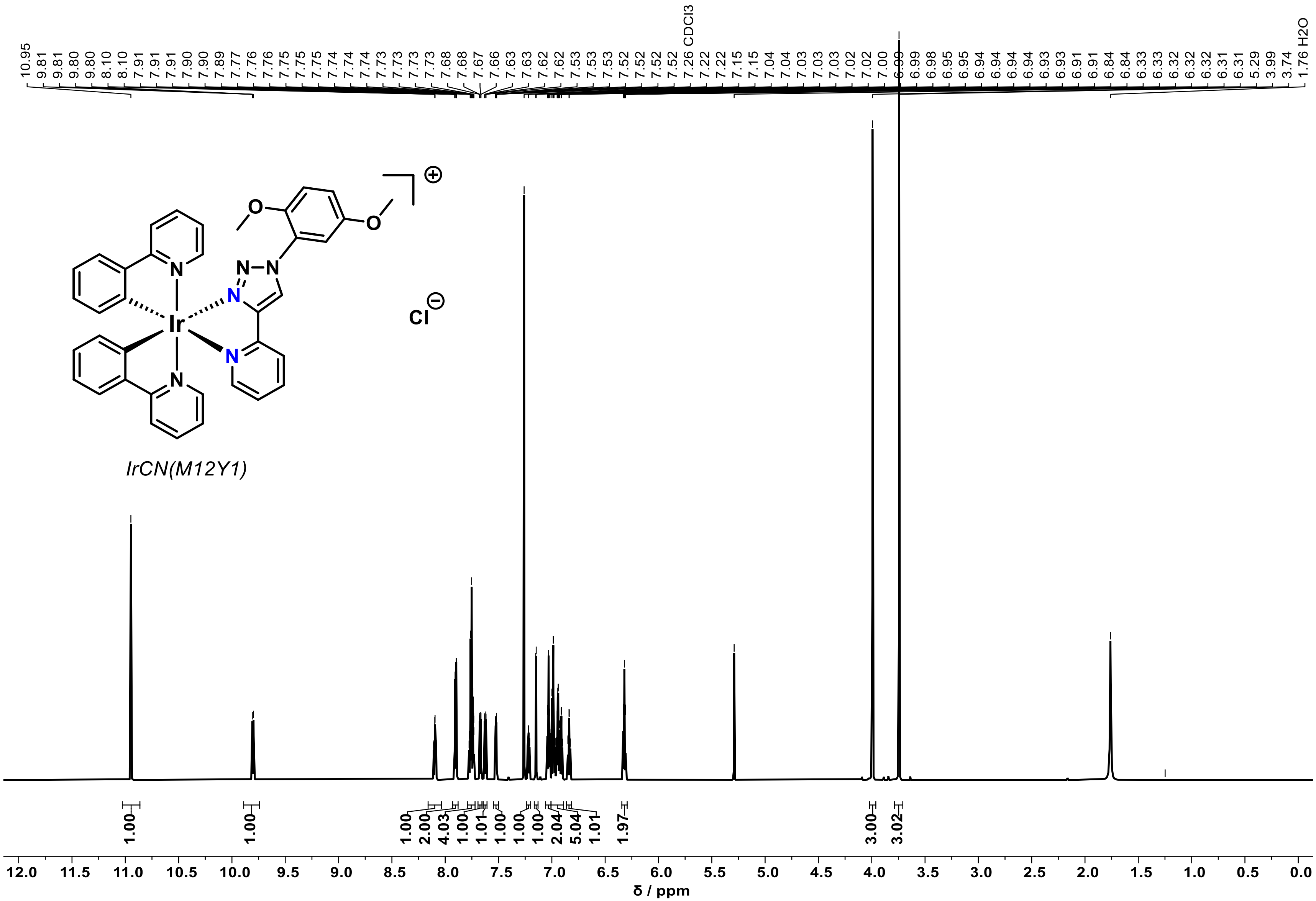

Figure S78: ^{1}H NMR (700 MHz, C*D*Cl$_3$, 32 scans, 12 s relaxation delay) spectrum of IrCN(M12Y1). Lab book ref. DRH-056

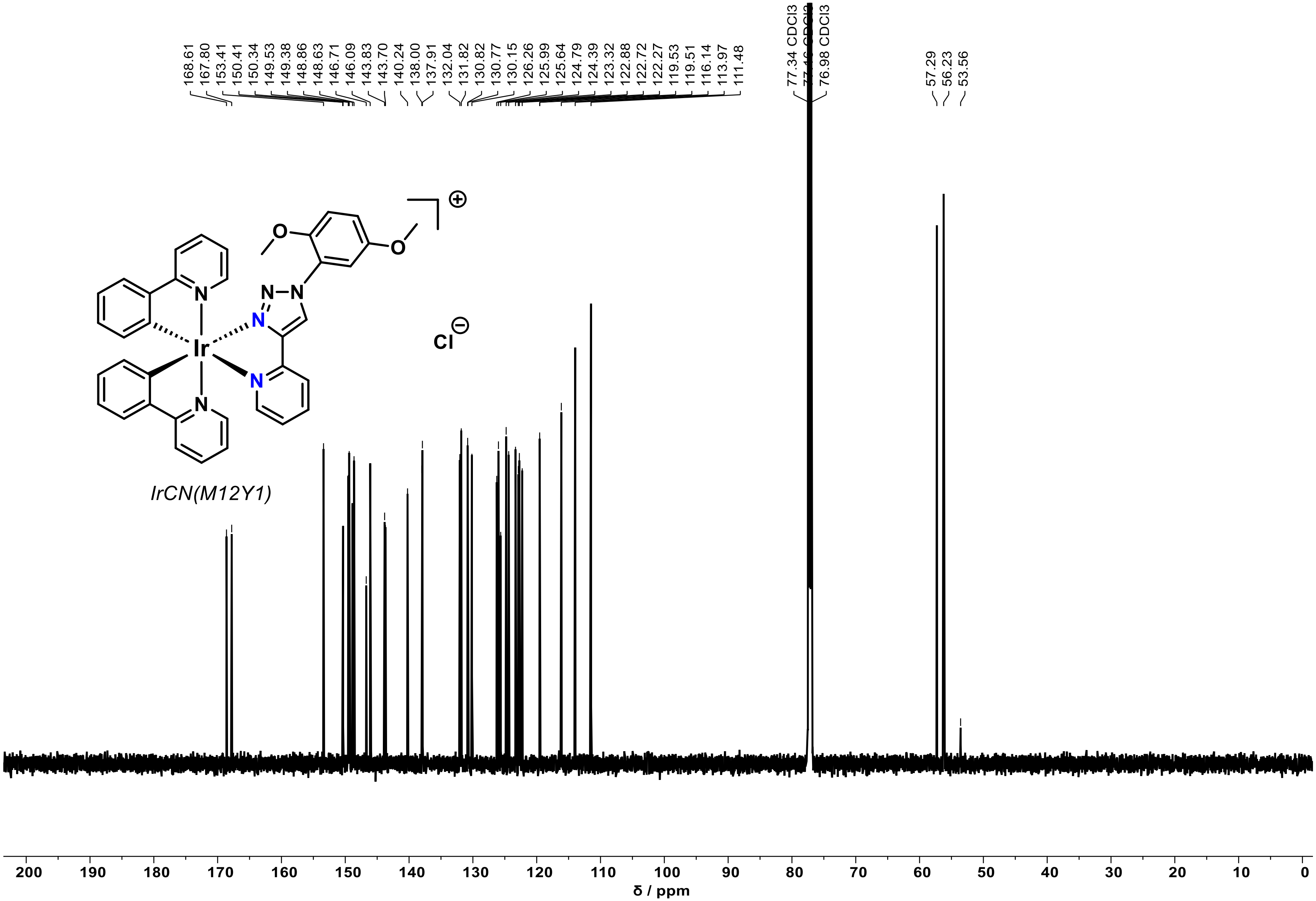

Figure S79: ^{13}C NMR (176 MHz, C*D*Cl$_3$, 2048 scans) spectrum of IrCN(M12Y1). Lab book ref. DRH-056

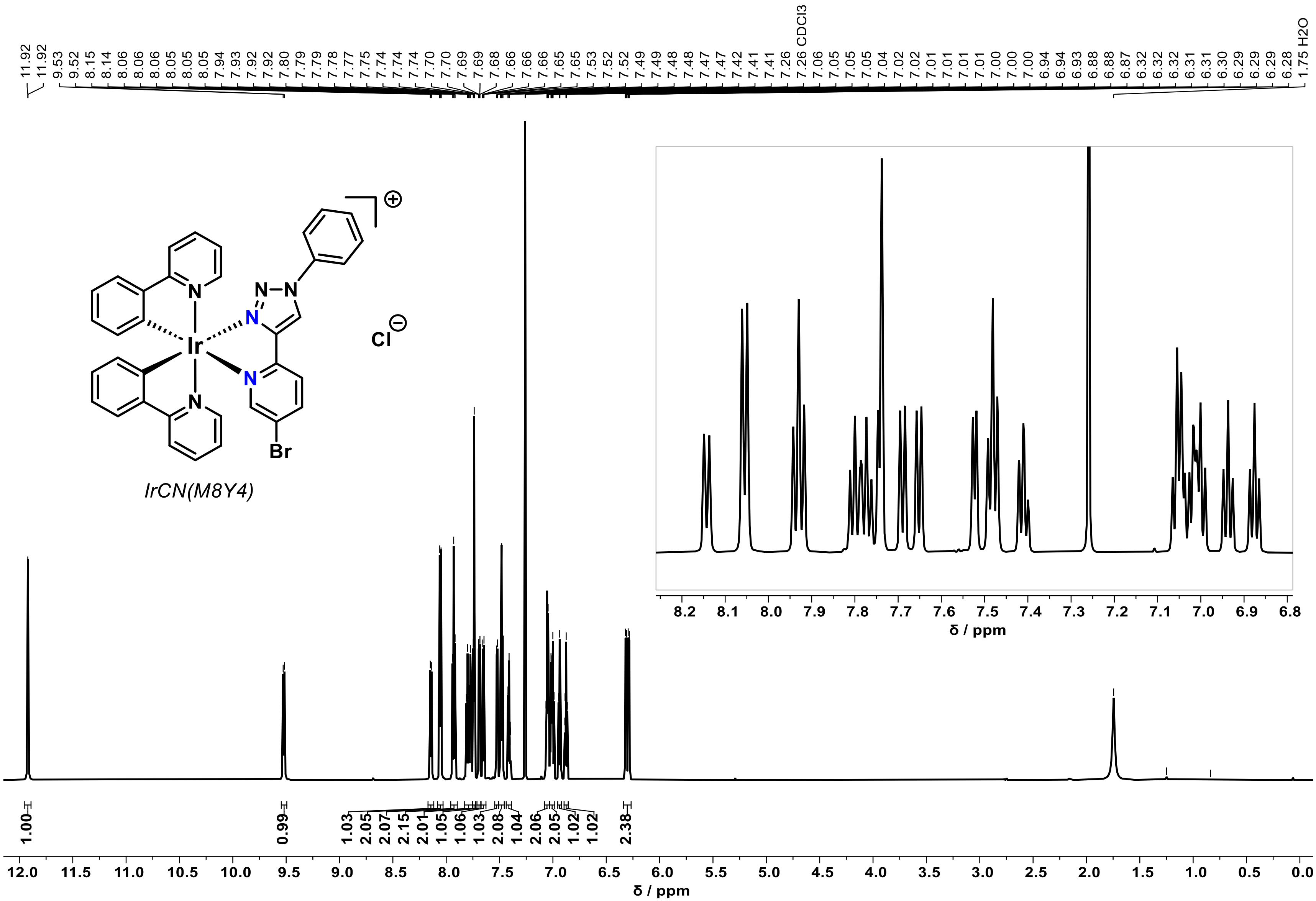

Figure S80: ^{1}H NMR (700 MHz, C*D*Cl$_3$, 32 scans, 12 s relaxation delay) spectrum of IrCN(M8Y4). Lab book ref. DRH-057

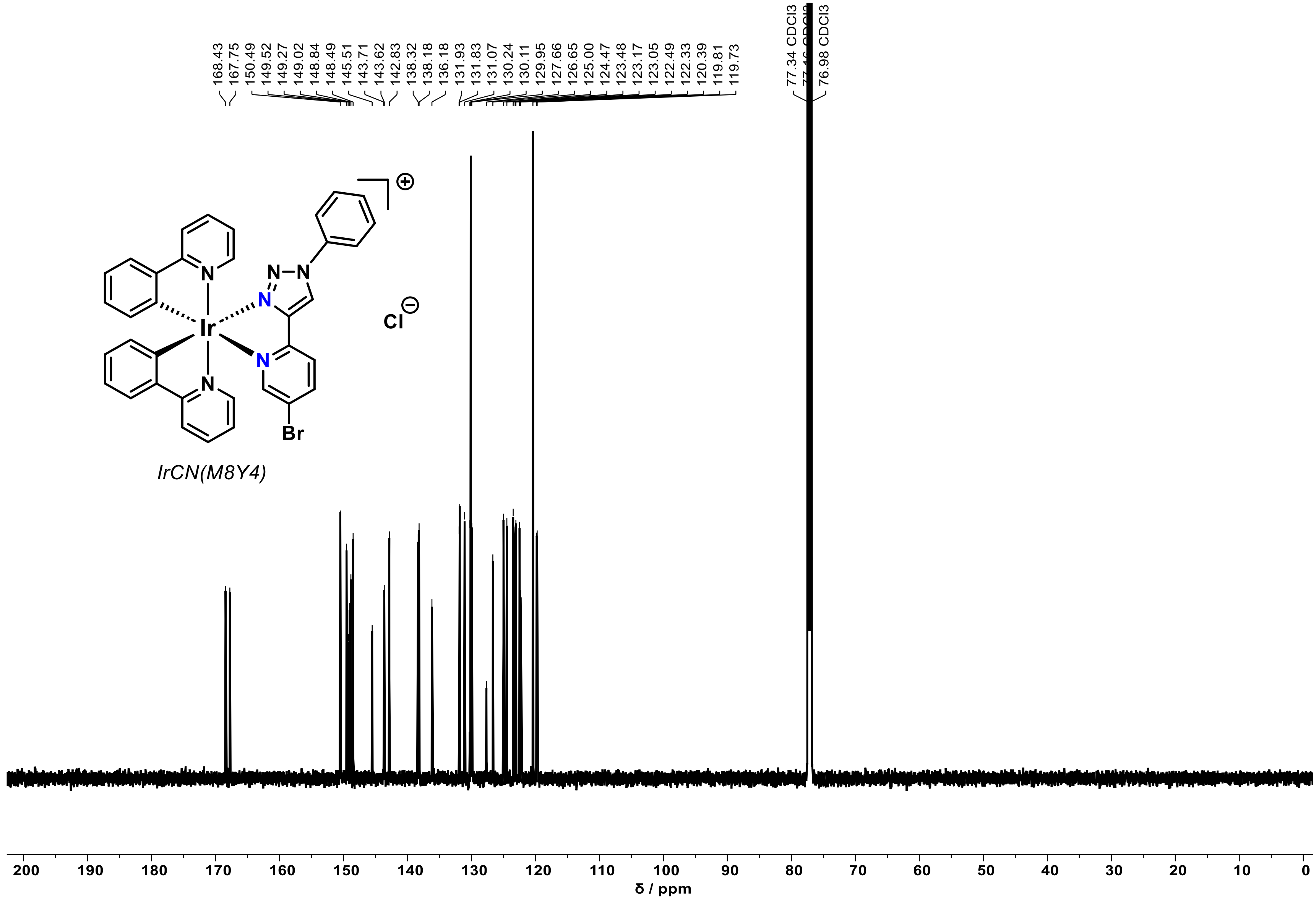

Figure S81: ^{13}**C NMR (176 MHz, C*D*Cl**$_3$**, 2048 scans) spectrum of IrCN(M8Y4). Lab book ref. DRH-057**

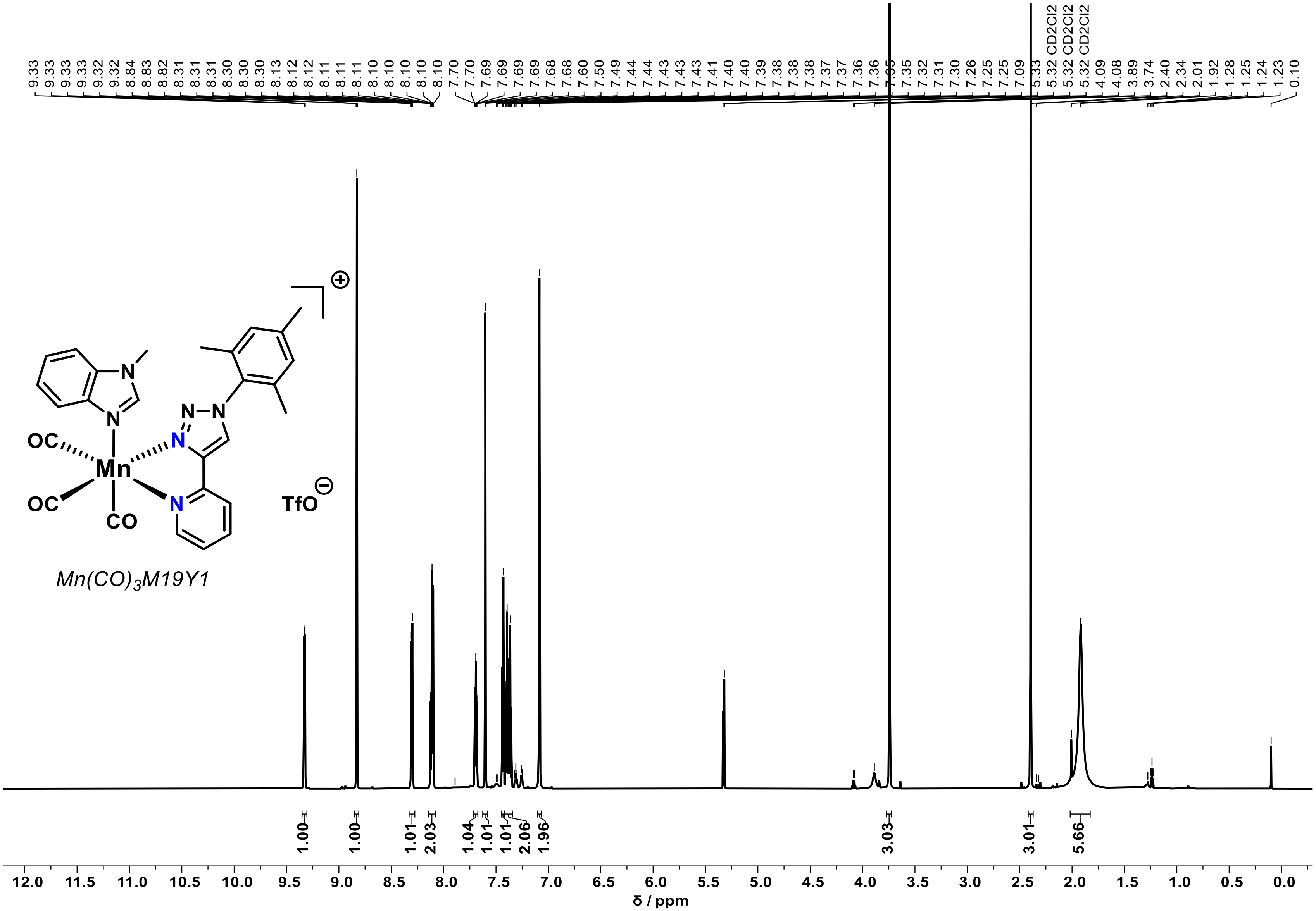

Figure S82: ^{1}H NMR (700 MHz, CD_2Cl_2, 16 scans, 12 s relaxation delay) spectrum of Mn(CO)$_3$M19Y1. Lab book ref. DRH-068-1

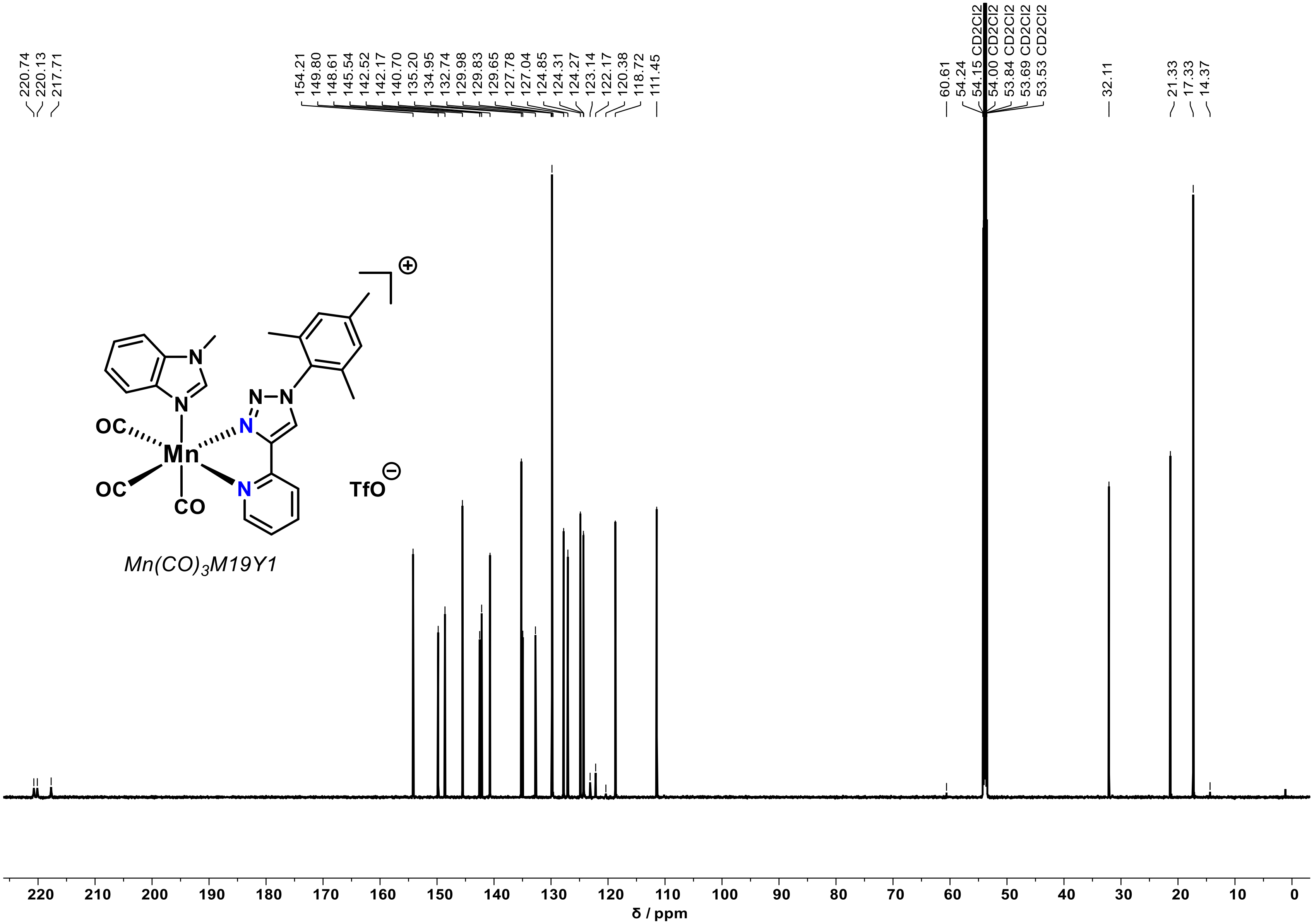

Figure S83: ^{13}C NMR (176 MHz, CD_2Cl_2, 1024 scans) spectrum of $Mn(CO)_3$M19Y1. Lab book ref. DRH-068-1

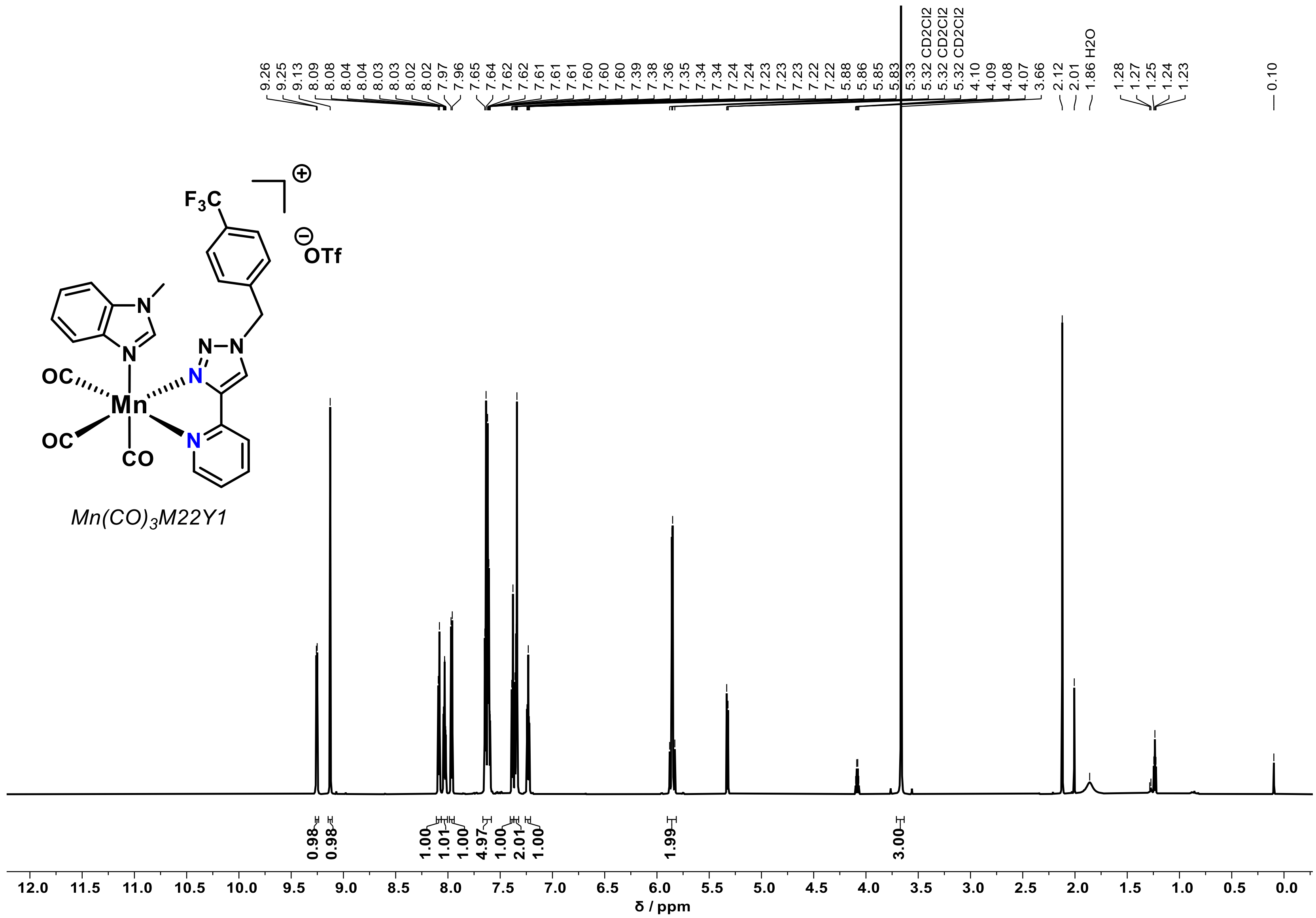

Figure S84: ^{1}H NMR (700 MHz, CD_2Cl$_2$, 16 scans, 12 s relaxation delay) spectrum of Mn(CO)$_3$M22Y1. Lab book ref. DRH-068-2

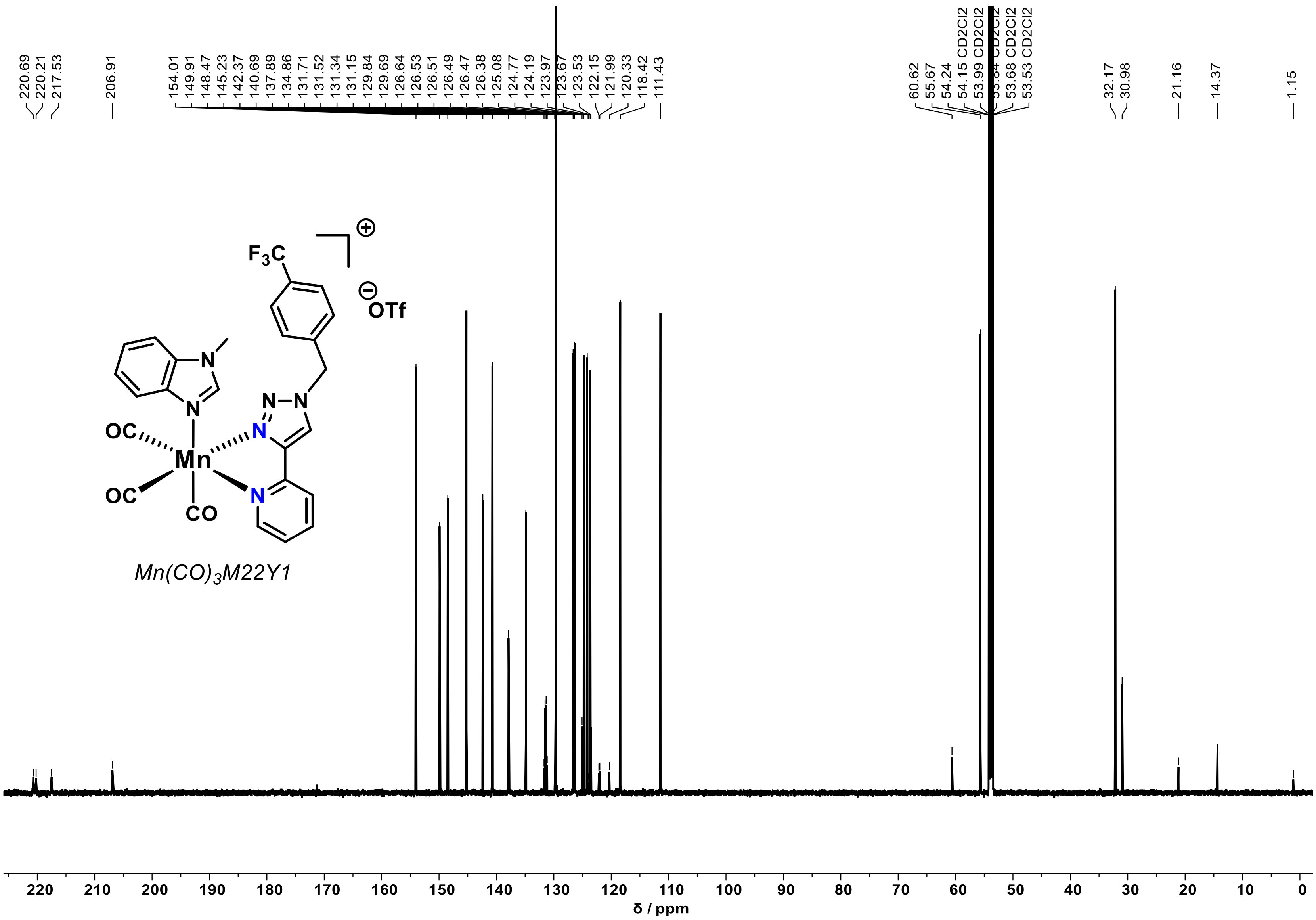

Figure S85: **^{13}C NMR (176 MHz, C*D*$_2$Cl$_2$, 512 scans) spectrum of Mn(CO)$_3$M22Y1. Lab book ref. DRH-068-2**

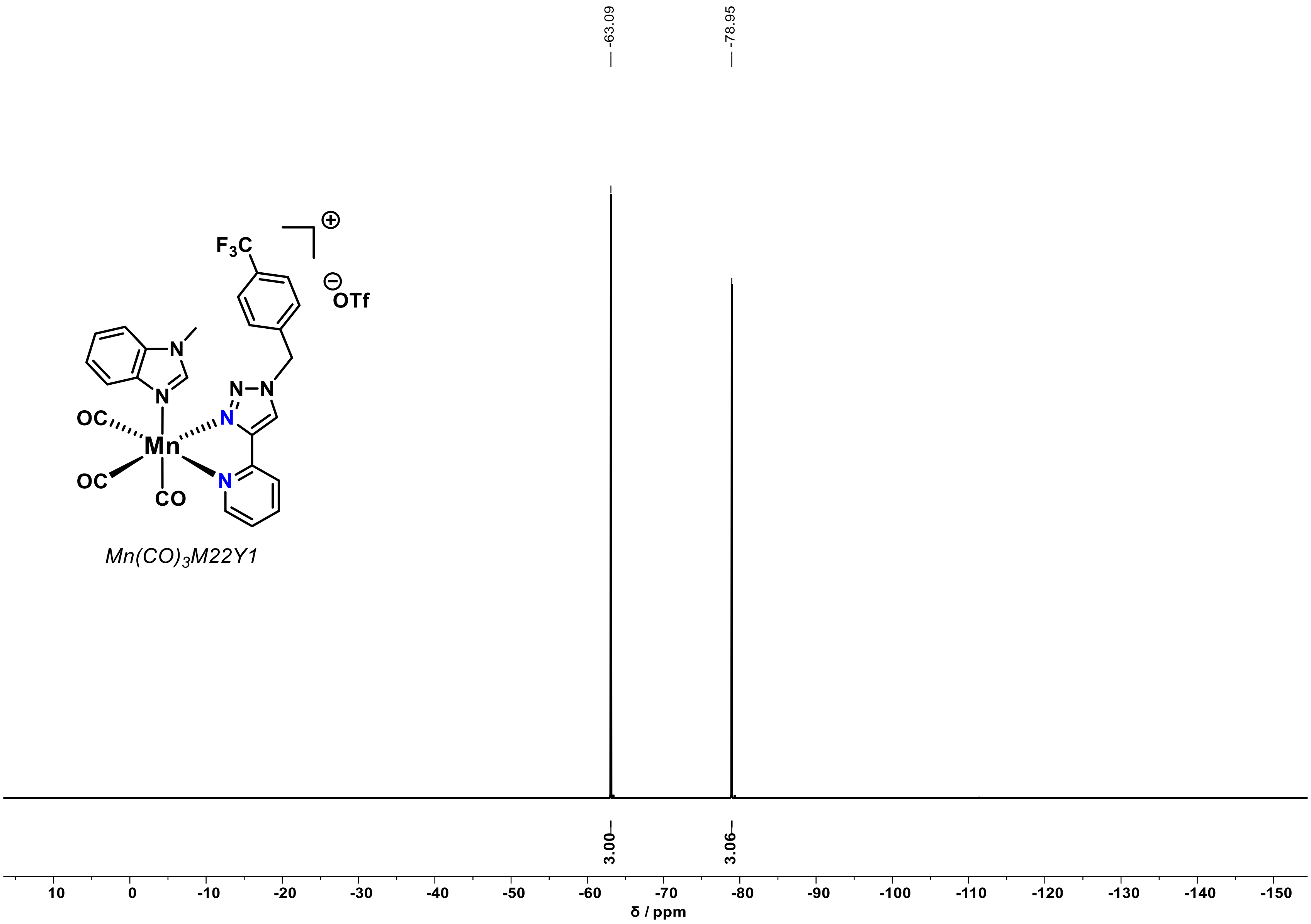

Figure S86: ^{19}F NMR (659 MHz, C*D*$_2$Cl$_2$, 64 scans) spectrum of Mn(CO)$_3$M22Y1. Lab book ref. DRH-068-2

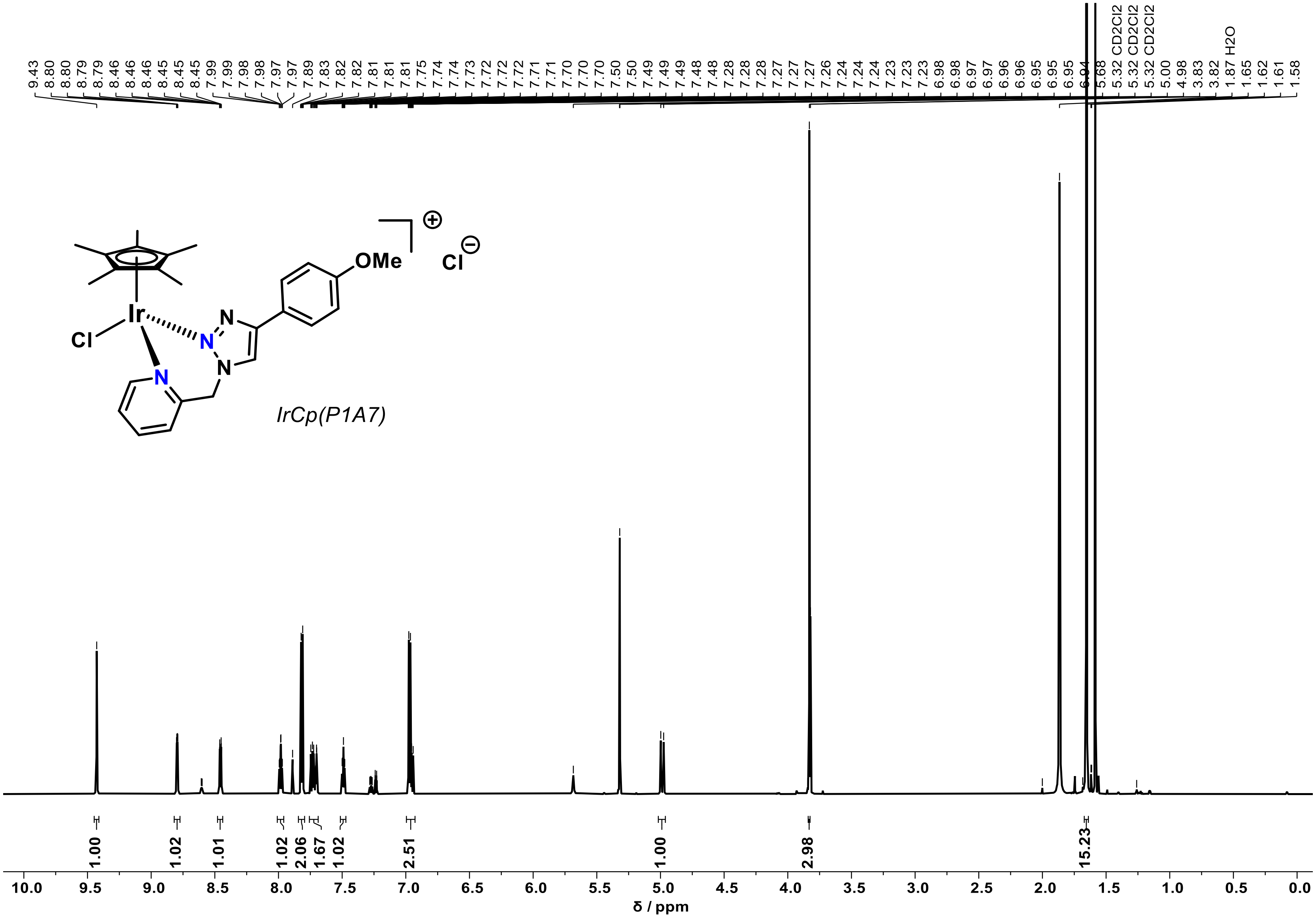

Figure S87: ^{1}H NMR (700 MHz, CD_2Cl_2, 32 scans, 12 s relaxation delay) spectrum of IrCp*(P1A7). Only peaks relevant to the complex are integrated, and in a couple of cases these overlap with free ligand peaks that are present in the sample (17% by ^{1}H integration). Lab book ref. DRH-088-2

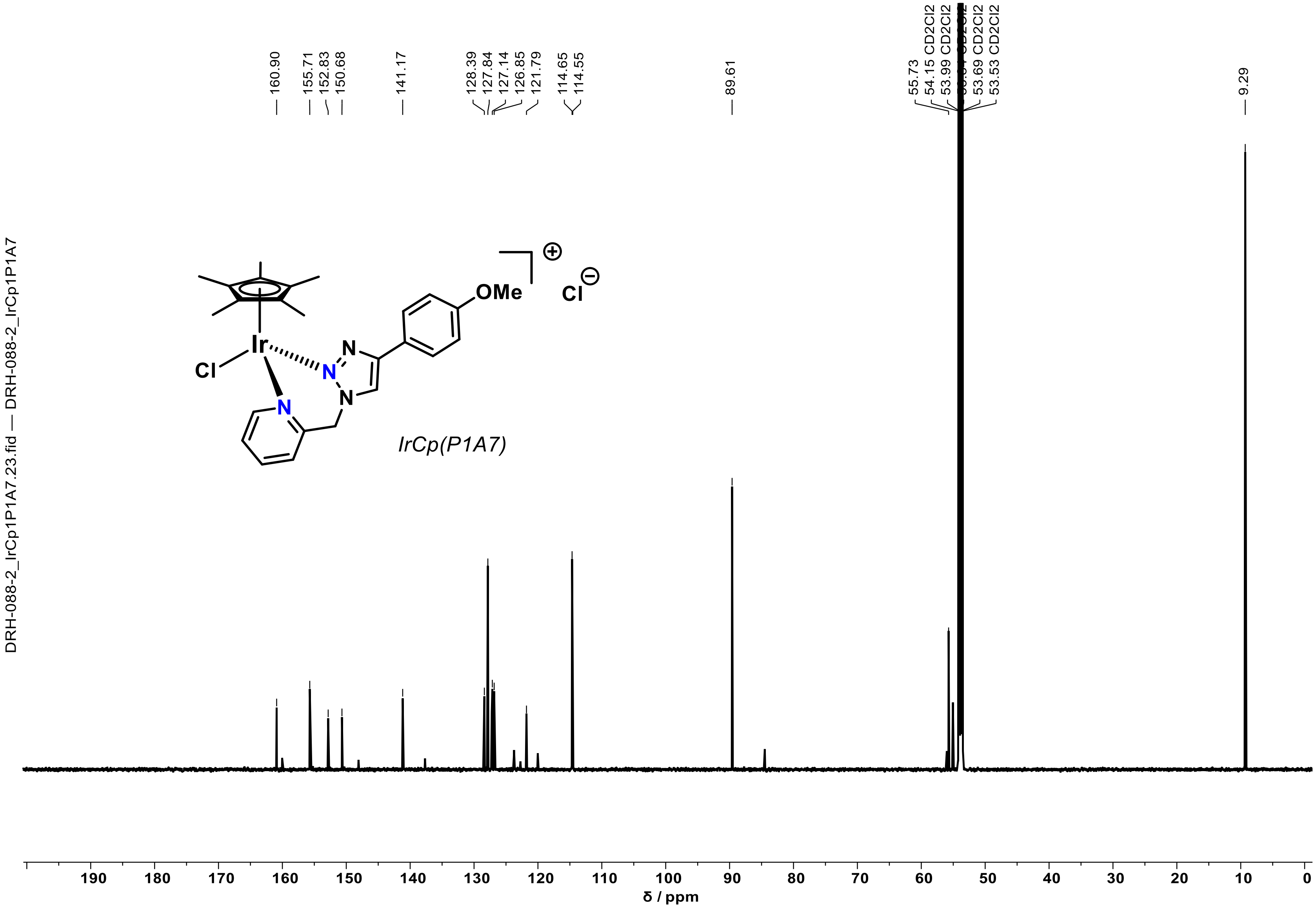

Figure S88: ^{13}C NMR (700 MHz, CD_2Cl$_2$, 2048 scans) spectrum of IrCp*(P1A7). Only peaks relevant to the complex are indicated, with free ligand peaks that are present in the sample (17% by ^{1}H integration). Lab book ref. DRH-088-2

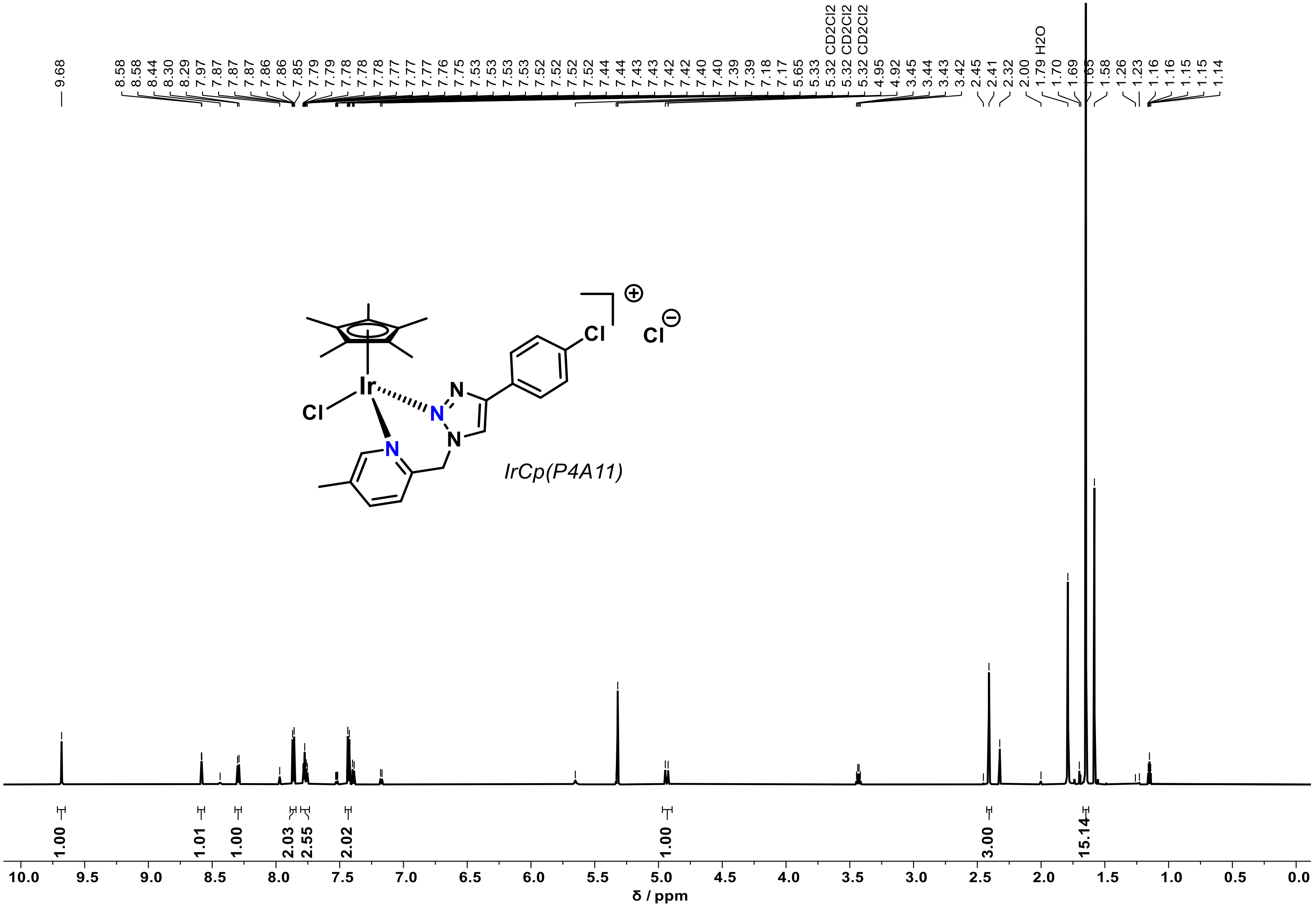

Figure S89: **^{1}H NMR (700 MHz, C*D*$_2$Cl$_2$, 32 scans, 12 s relaxation delay) spectrum of IrCp*(P4A11). Only peaks relevant to the complex are integrated, and in a couple of cases these overlap with free ligand peaks that are present in the sample (19% by ^{1}H integration). Lab book ref. DRH-088-3**

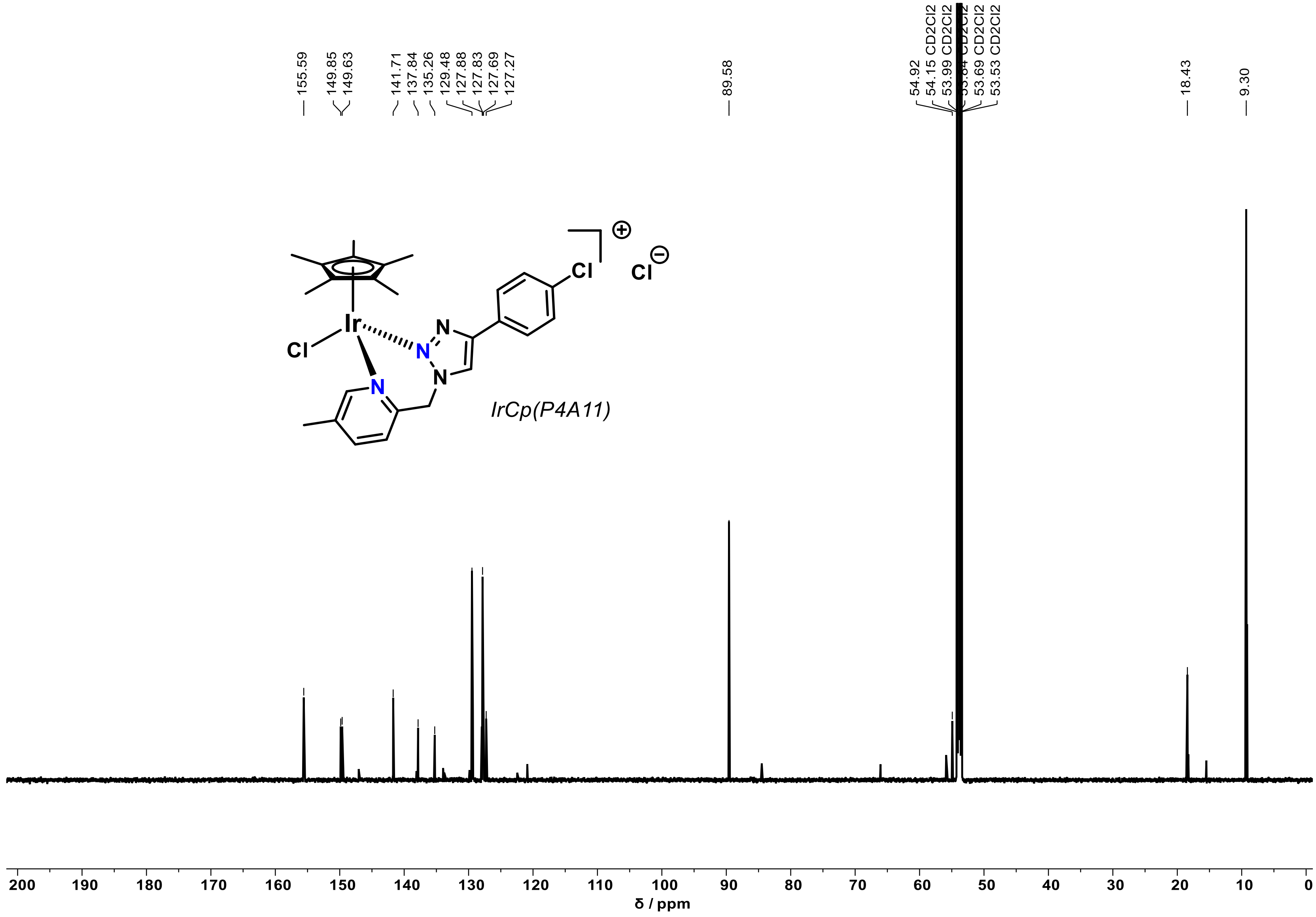

Figure S90: ^{13}C NMR (700 MHz, CD_2Cl$_2$, 2048 scans) spectrum of IrCp*(P4A11). Only peaks relevant to the complex are indicated, with free ligand peaks that are present in the sample (19% by ^{1}H integration). Lab book ref. DRH-088-2

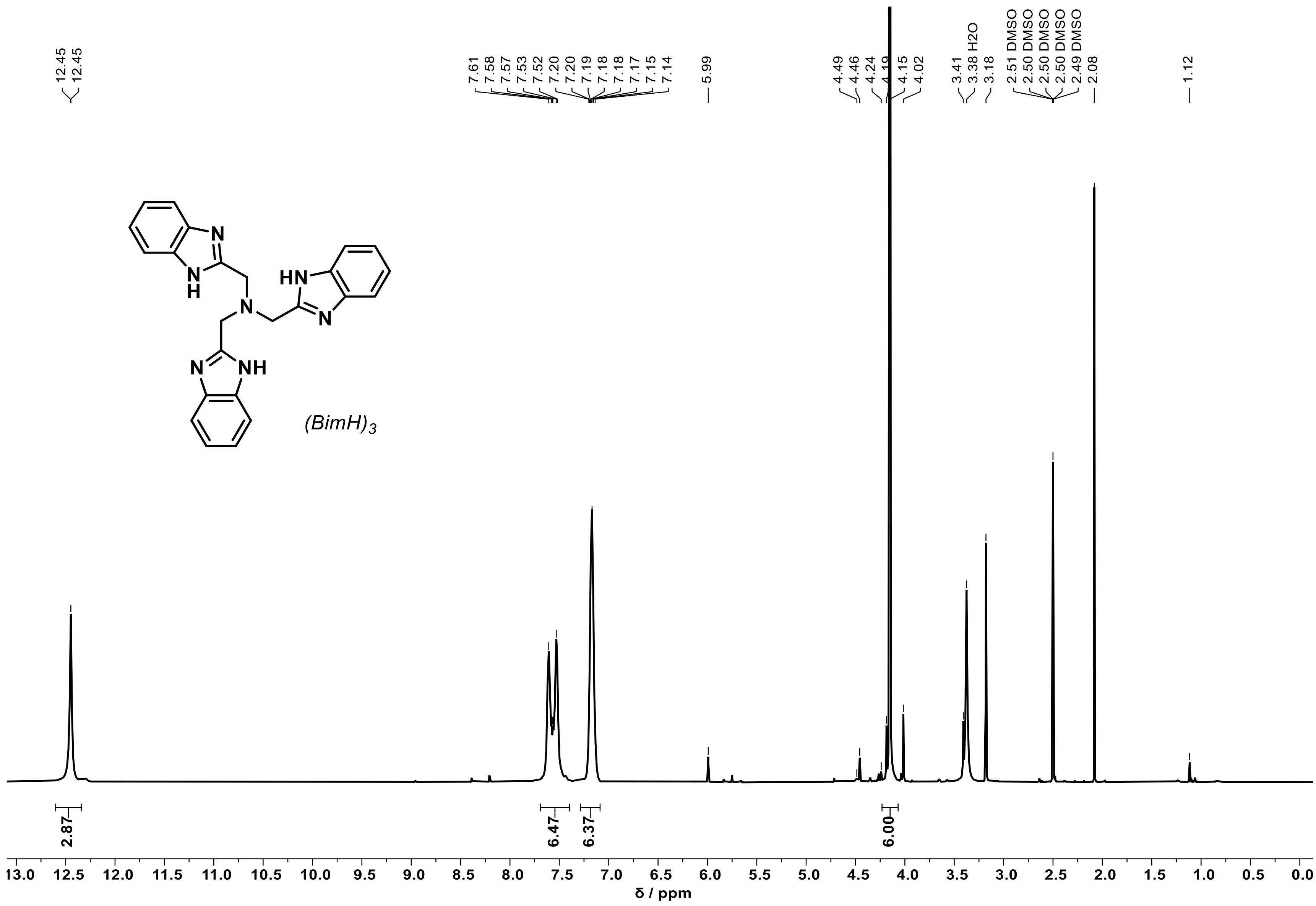

Figure S91: ^{1}H NMR (600 MHz, DMSO-d_6, 16 scans, 10 s relaxation delay) spectrum of $(BimH)_3$. Lab book ref. DRH-008

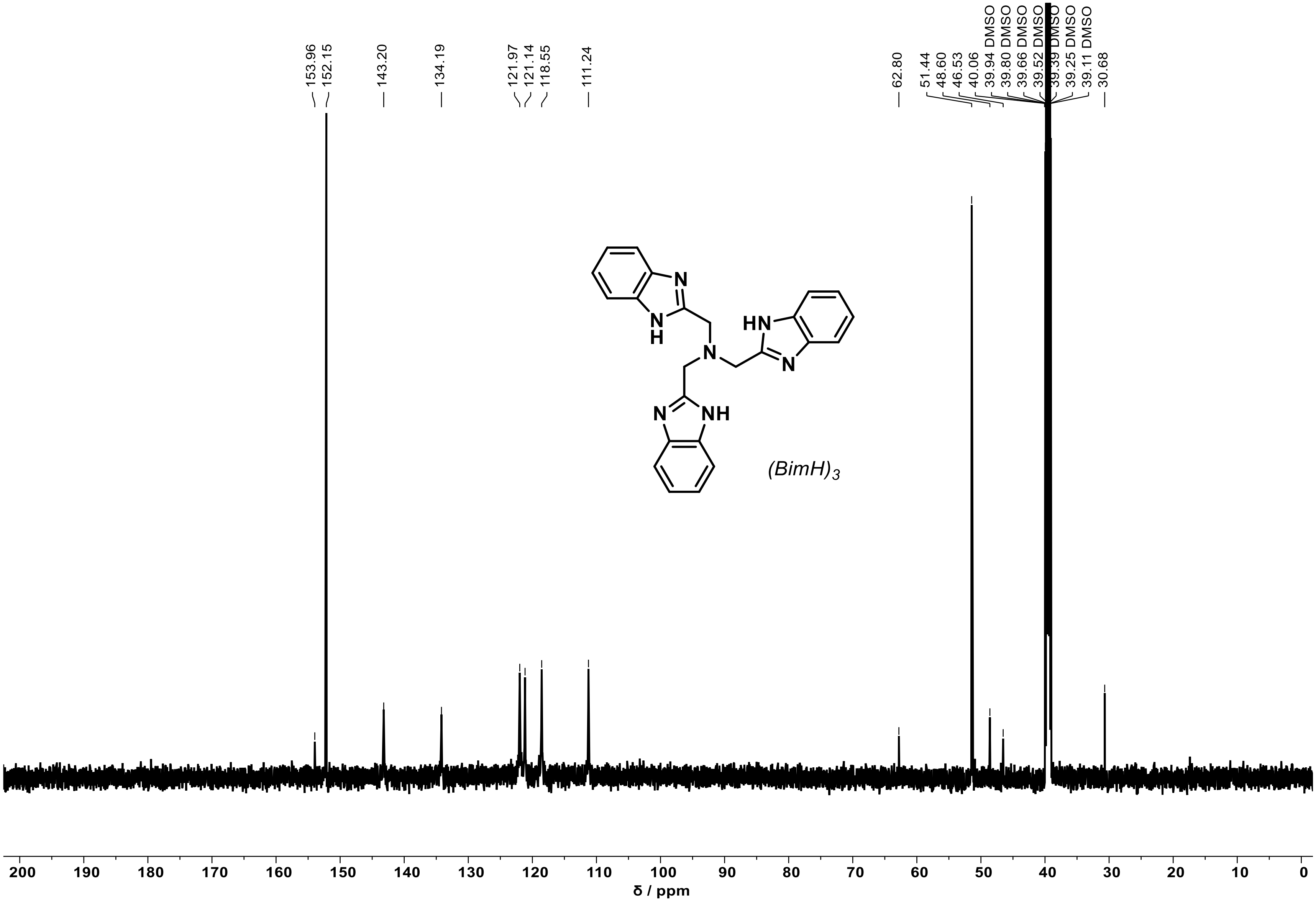

Figure S92: ^{13}**C NMR (151 MHz, DMSO-*d*$_6$, 256 scans) spectrum of (BimH)3. Lab book ref. DRH-008**

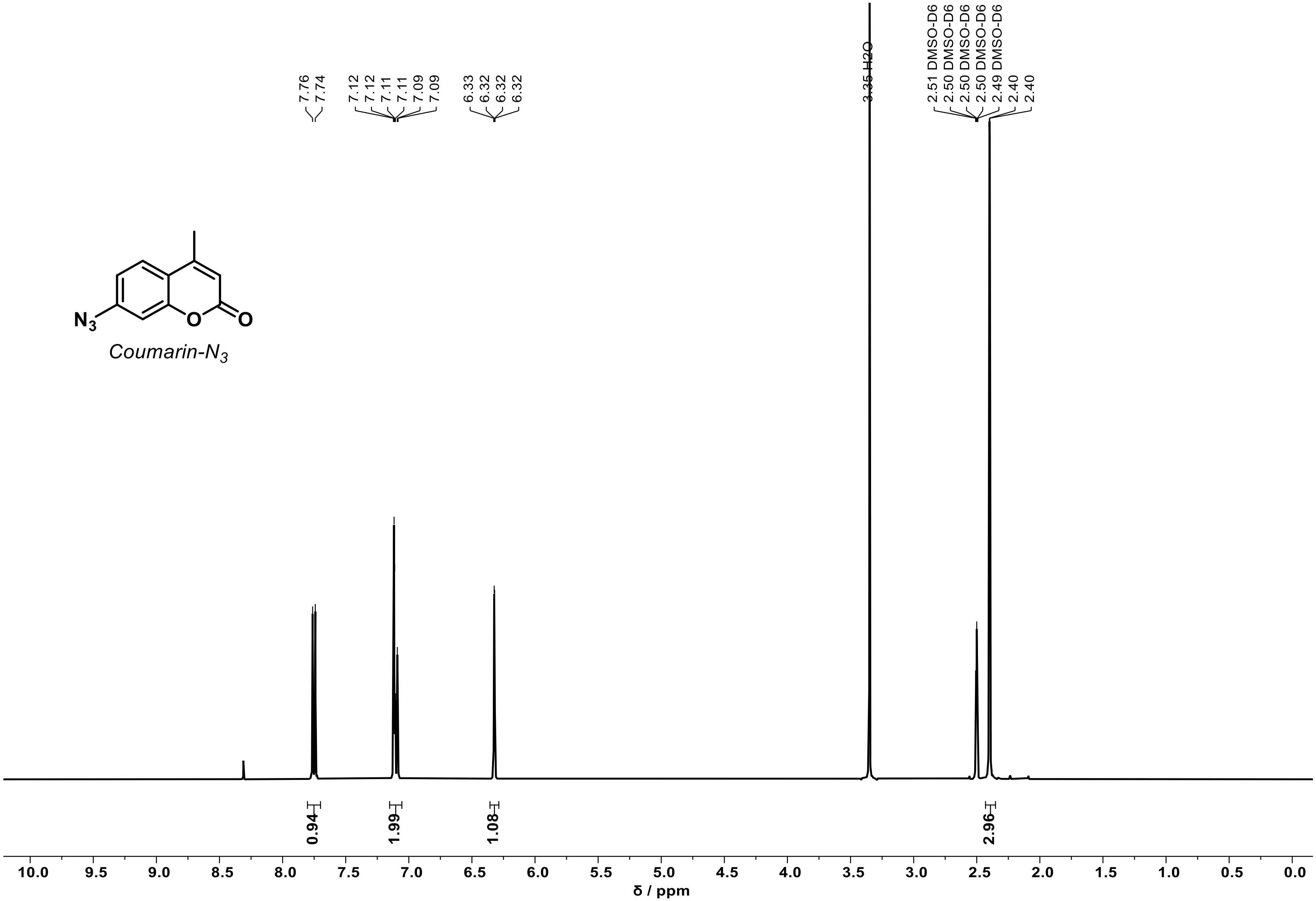

Figure S93: ^{1}H NMR (400 MHz, DMSO-d_6, 32 scans, 10 s relaxation delay) spectrum of Coumarin-N_3. Lab book ref. DRH-030

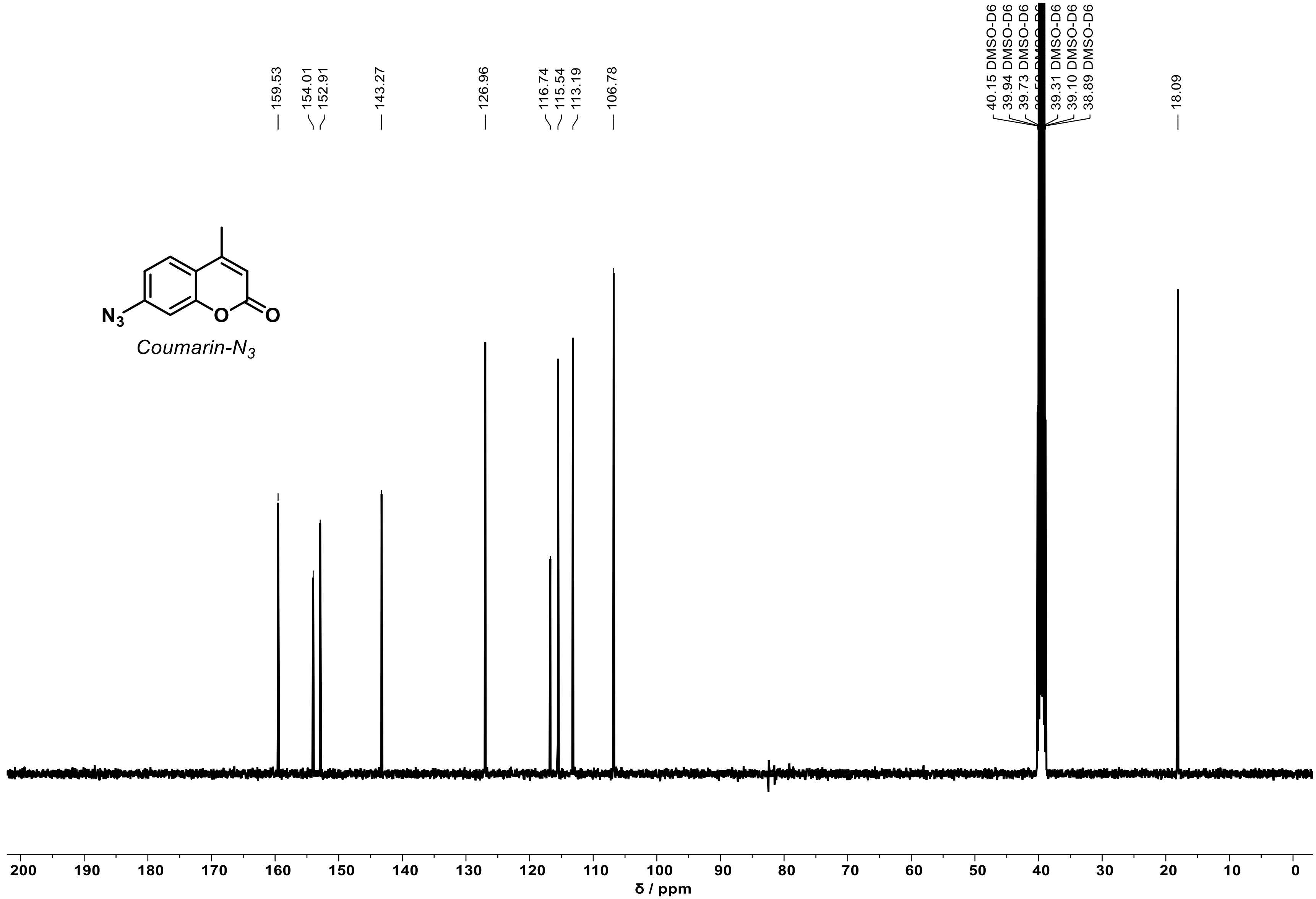

Figure S94: ^{13}C NMR (101 MHz, DMSO-d_6, 2048 scans) spectrum of Coumarin-N_3. Lab book ref. DRH-030

7. HRMS data for Organic and Inorganic Compounds

York - Chemistry - Mass Spectrometry Service Report

DRH-051-1_af117401dh

Analysis Information

Analysis Filename	af117401dh_P1-B-1_01_55634.d	Acquisition Date	21/02/2025 10:25:20
Method	_ESI_low mass 1200series.m	Instrument	compact
Submission Name	af117401dh	ESI	Positive

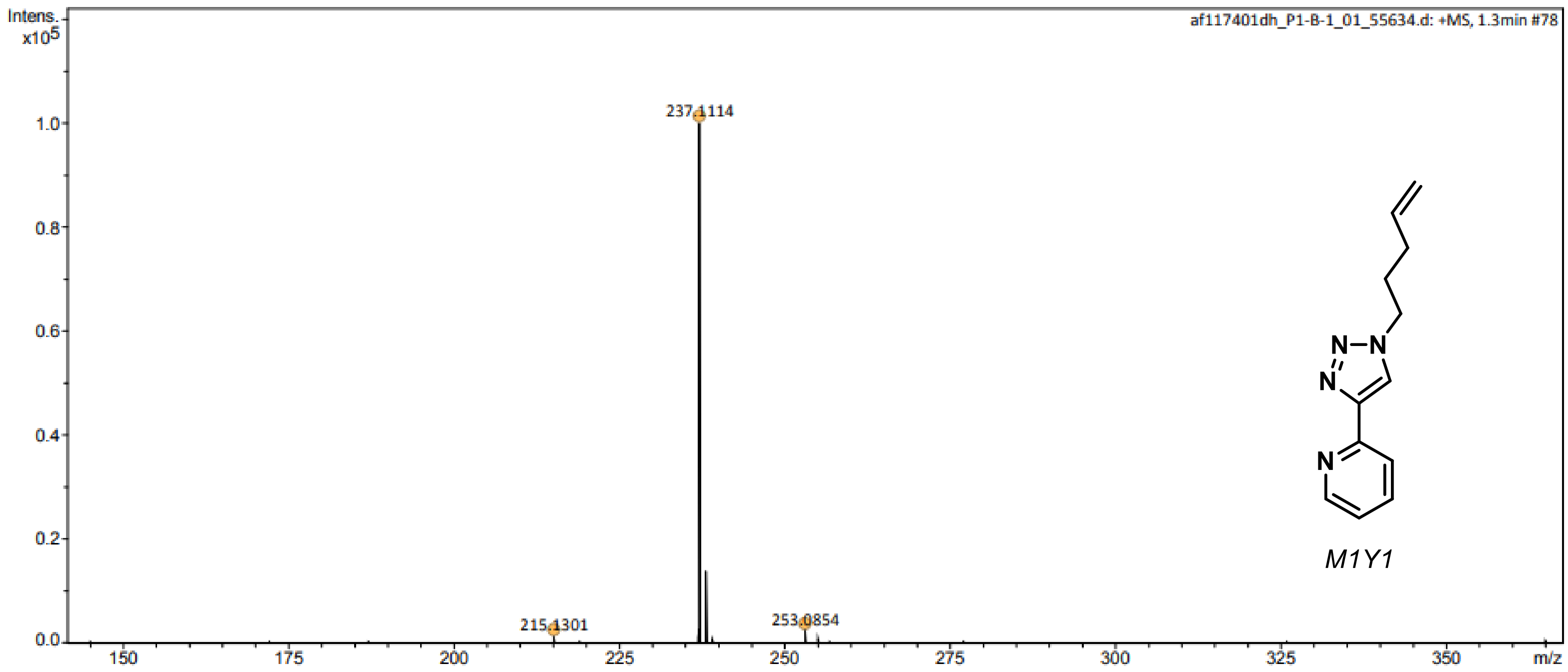

Meas. m/z	#	Ion Formula	m/z	err [ppm]	err [mDa]	mSigma	Mean err [ppm]
215.1301	1	C12H15N4	215.1291	-4.4	-1.0	15.5	-2.4
237.1114	1	C12H14N4Na	237.1111	-1.5	-0.3	3.6	0.8
253.0854	1	C12H14KN4	253.0850	-1.6	-0.4	n.a.	-0.7

Figure S95: HRMS (ESI positive mode) of M1Y1 (lab book ref. DRH-051-1)

York - Chemistry - Mass Spectrometry Service Report

DRH-051-2_af117435dh

Analysis Information

Analysis Filename	af117435dh_P1-D-8_01_55666.d	Acquisition Date	24/02/2025 07:17:16
Method	_ESI_low mass 1200series.m	Instrument	compact
Submission Name	af117435dh	ESI	Positive

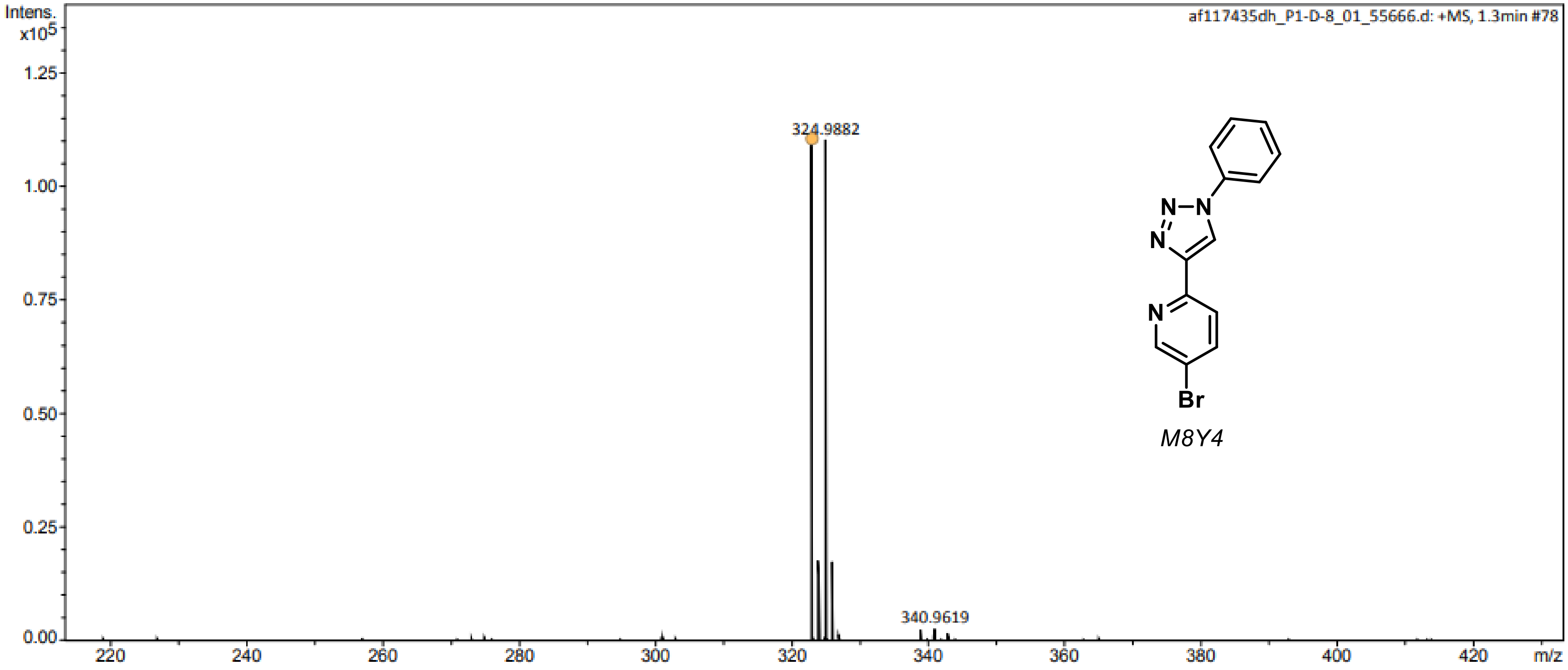

Meas. m/z	#	Ion Formula	m/z	err [ppm]	err [mDa]	mSigma	Mean err [ppm]
322.9900	1	C13H9BrN4Na	322.9903	0.7	0.2	8.9	0.4

Figure S96: HRMS (ESI positive mode) of M8Y4 (lab book ref. DRH-051-2)

York - Chemistry - Mass Spectrometry Service Report

DRH-051-3_af117436dh

Analysis Information

Analysis Filename	af117436dh_P1-D-9_01_55667.d	Acquisition Date	24/02/2025 07:20:16
Method	_ESI_low mass 1200series.m	Instrument	compact
Submission Name	af117436dh	ESI	Positive

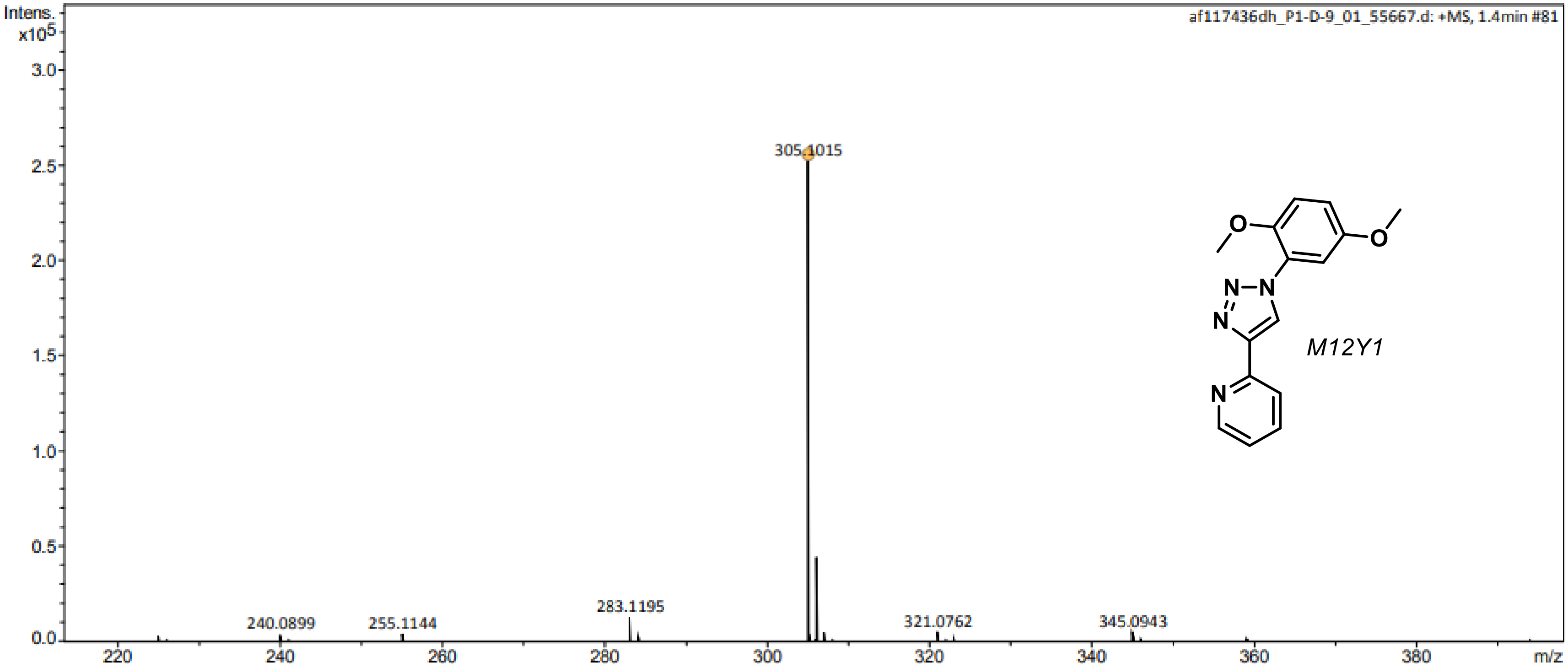

Meas. m/z	#	Ion Formula	m/z	err [ppm]	err [mDa]	mSigma	Mean err [ppm]
305.1015	1	C15H14N4NaO2	305.1009	-2.1	-0.7	1.8	-1.8

Figure S97: HRMS (ESI positive mode) of M12Y1 (lab book ref. DRH-051-3)

York - Chemistry - Mass Spectrometry Service Report

DRH-051-5_af117588dh

Analysis Information

Analysis Filename	af117588dh_P1-B-8_01_55845.d	Acquisition Date	27/02/2025 15:04:35
Method	_ESI_low mass 1200series.m	Instrument	compact
Submission Name	af117588dh	ESI	Positive

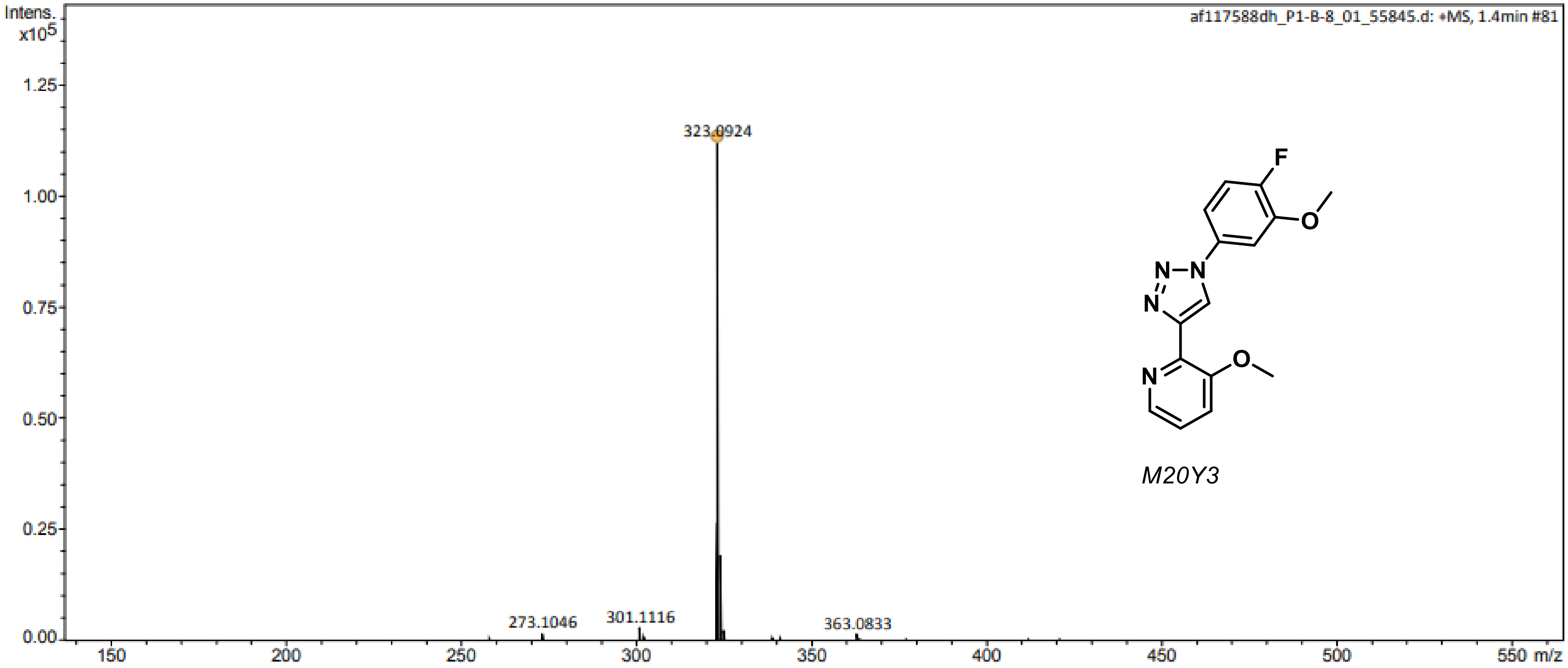

Meas. m/z	#	Ion Formula	m/z	err [ppm]	err [mDa]	mSigma	Mean err [ppm]
323.0924	1	C15H13FN4NaO2	323.0915	-2.9	-1.0	3.8	-3.2

Figure S98: HRMS (ESI positive mode) of M20Y3 (lab book ref. DRH-051-5)

York - Chemistry - Mass Spectrometry Service Report

DRH-066-M19Y1_af119618dh

Analysis Information

Analysis Filename	af119618dh_P1-D-2_01_57981.d	Acquisition Date	14/05/2025 07:07:42
Method	ESI_low mass_2c1s.m	Instrument	compact
Submission Name	af119618dh	ESI	Positive

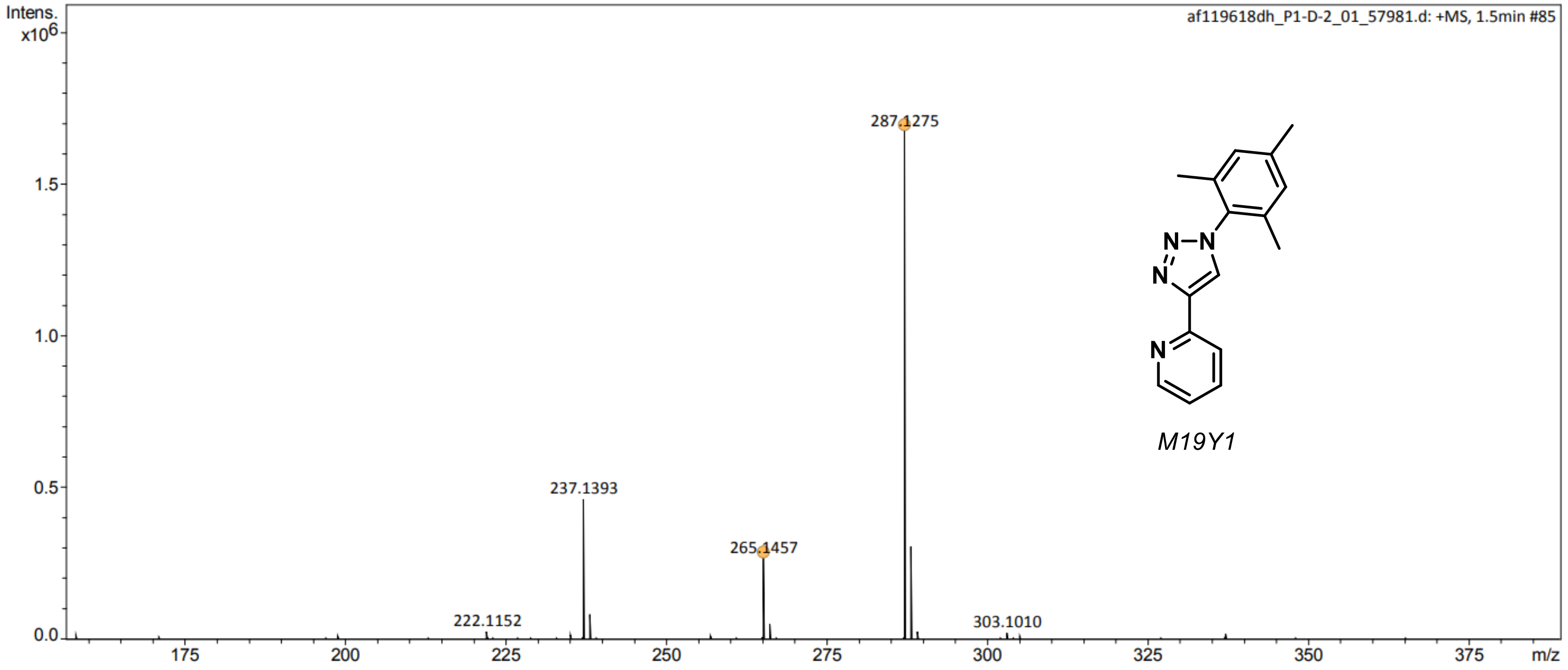

Meas. m/z	#	Ion Formula	m/z	err [ppm]	err [mDa]	mSigma	Mean err [ppm]
265.1457	1	C16H17N4	265.1448	-3.3	-0.9	1.8	-3.0
287.1275	1	C16H16N4Na	287.1267	-2.6	-0.7	4.3	-3.3

Figure S99: HRMS (ESI positive mode) of M19Y1 (lab book ref. DRH-066-1)

York - Chemistry - Mass Spectrometry Service Report

DRH-066-M22Y1_af119619dh

Analysis Information

Analysis Filename	af119619dh_P1-D-3_01_57982.d	Acquisition Date	14/05/2025 07:10:42
Method	ESI_low mass_2c1s.m	Instrument	compact
Submission Name	af119619dh	ESI	Positive

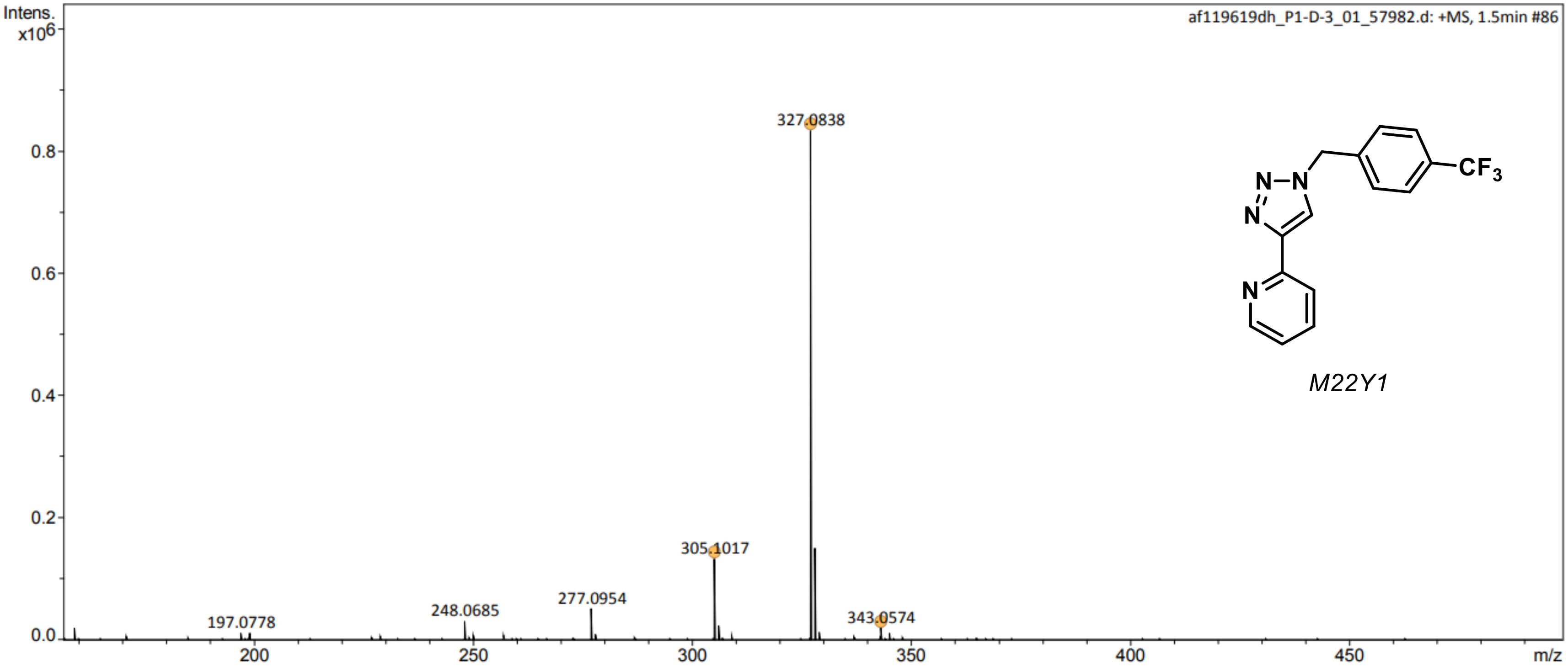

Meas. m/z	#	Ion Formula	m/z	err [ppm]	err [mDa]	mSigma	Mean err [ppm]
305.1017	1	C15H12F3N4	305.1009	-2.9	-0.9	0.9	-4.0
327.0838	1	C15H11F3N4Na	327.0828	-3.0	-1.0	1.8	-4.4
343.0574	1	C15H11F3KN4	343.0567	-1.8	-0.6	49.1	-0.6

Figure S100: HRMS (ESI positive mode) of M22Y1 (lab book ref. DRH-066-2)

York - Chemistry - Mass Spectrometry Service Report

DRH-085-2_af121299dh

Analysis Information

Analysis Filename	af121299dh_P1-F-3_01_59802.d	Acquisition Date	26/08/2025 10:14:44
Method	ESI_low mass_2c1s.m	Instrument	compact
Submission Name	af121299dh	ESI	Positive

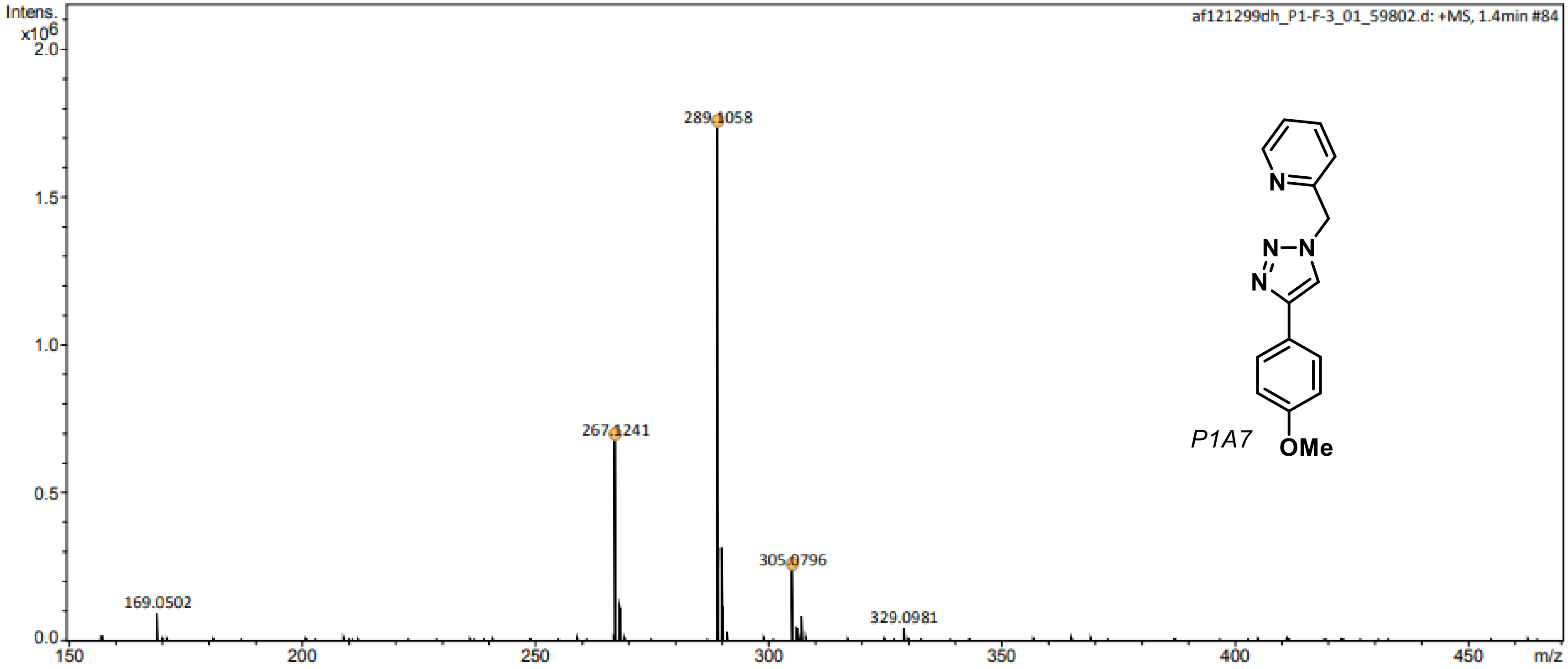

Meas. m/z	#	Ion Formula	m/z	err [ppm]	err [mDa]	mSigma	Mean err [ppm]
267.1241	1	C15H15N4O	267.1240	-0.1	-0.0	4.0	-1.0
289.1058	1	C15H14N4NaO	289.1060	0.5	0.2	1.5	0.1
305.0796	1	C15H14KN4O	305.0799	0.9	0.3	45.4	-0.2

Figure S101: HRMS (ESI positive mode) of P1A7 (lab book ref. DRH-085-2)

York - Chemistry - Mass Spectrometry Service Report

DRH-085-3_af121300dh

Analysis Information

Analysis Filename	af121300dh_P1-F-4_01_59803.d	Acquisition Date	26/08/2025 10:17:43
Method	ESI_low mass_2c1s.m	Instrument	compact
Submission Name	af121300dh	ESI	Positive

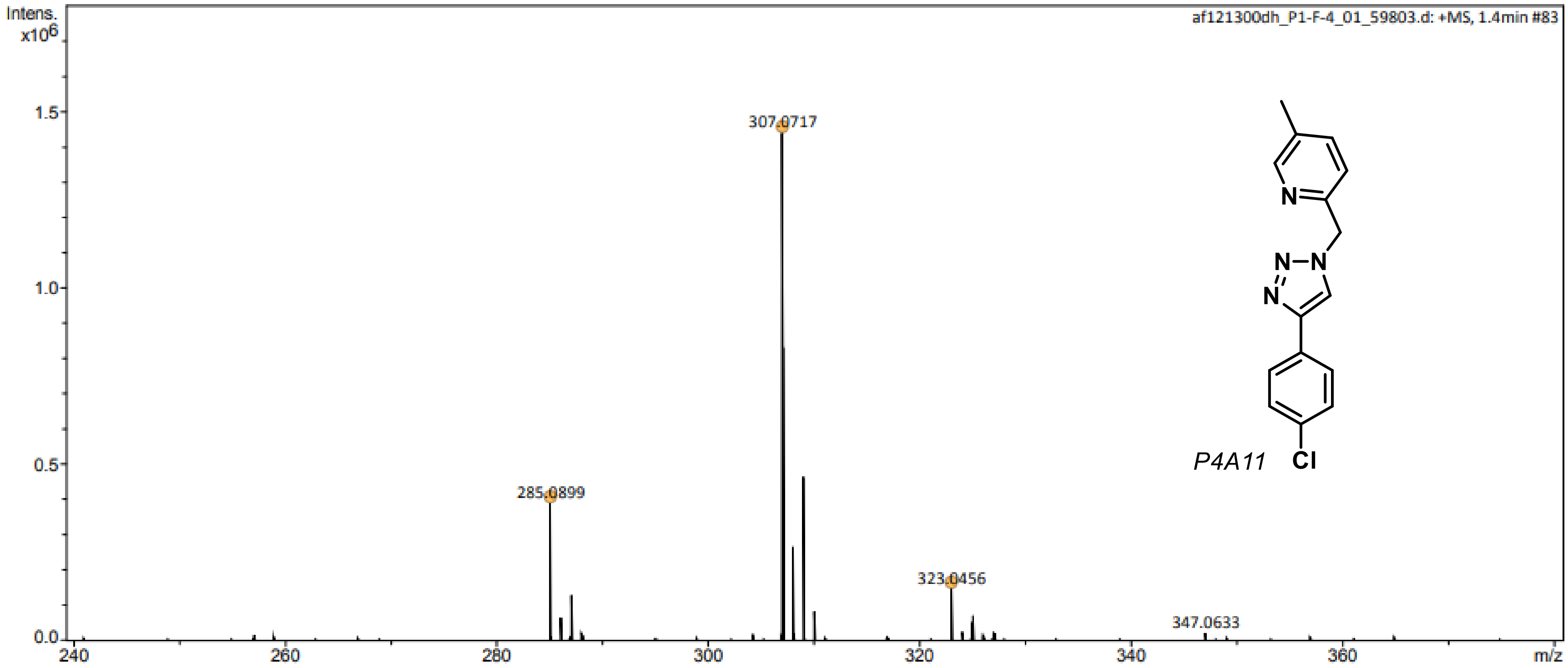

Meas. m/z	#	Ion Formula	m/z	err [ppm]	err [mDa]	mSigma	Mean err [ppm]
285.0899	1	C15H14ClN4	285.0902	1.0	0.3	5.3	1.1
307.0717	1	C15H13ClN4Na	307.0721	1.2	0.4	6.5	1.1
323.0456	1	C15H13ClKN4	323.0460	1.5	0.5	14.4	-0.1

Figure S102: HRMS (ESI positive mode) of P4A11 (lab book ref. DRH-085-3)

York - Chemistry - Mass Spectrometry Service Report

DRH-052_af117557dh

Analysis Information

Analysis Filename	af117557dh_P1-E-5_01_55798.d	Acquisition Date	26/02/2025 14:03:51
Method	_ESI_low mass 1200series.m	Instrument	compact
Submission Name	af117557dh	ESI	Positive

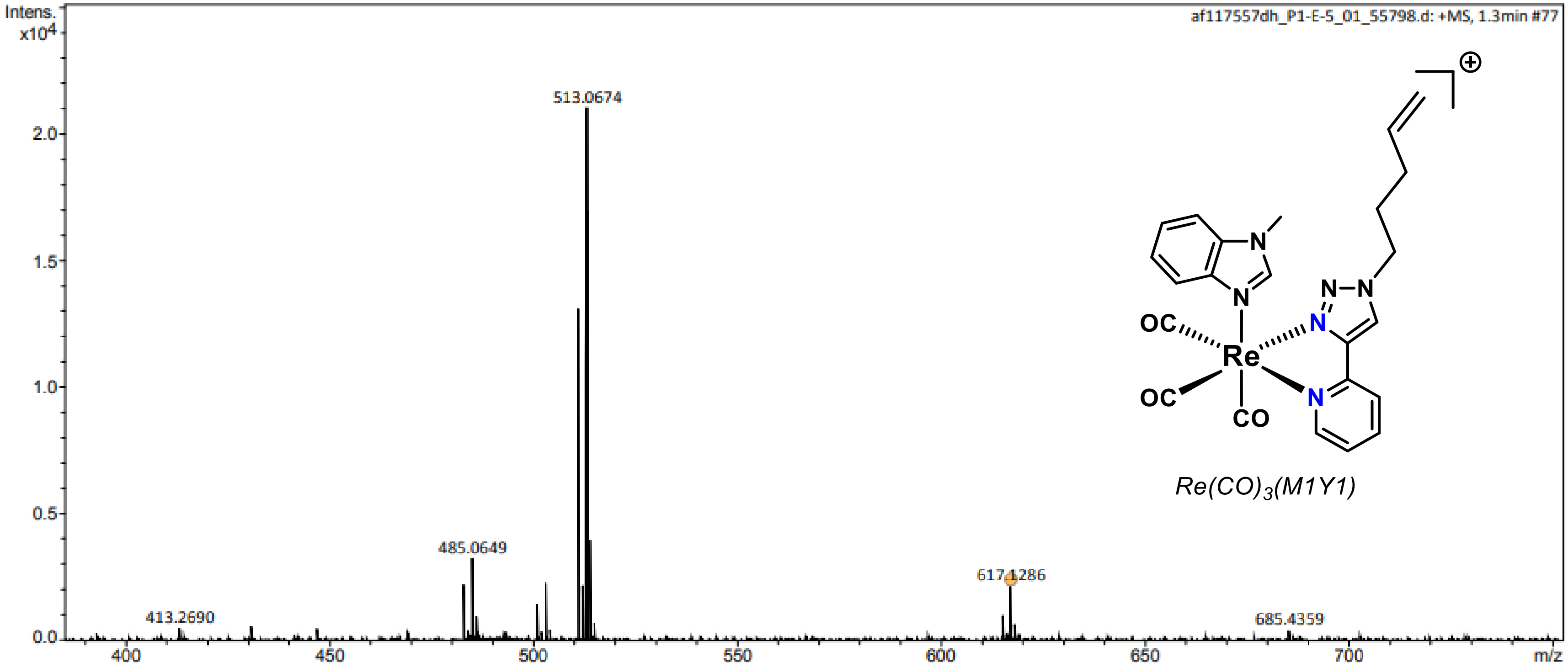

Meas. m/z	#	Ion Formula	m/z	err [ppm]	err [mDa]	mSigma	Mean err [ppm]
617.1286	1	C23H22N6O3Re	617.1305	3.2	2.0	70.5	3.9

Figure S103: HRMS (ESI positive mode) of $Re(CO)_3$(M1Y1) (lab book ref. DRH-052)

York - Chemistry - Mass Spectrometry Service Report

DRH-053_af117608dh

Analysis Information

Analysis Filename	af117608dh_P1-D-1_01_55858.d	Acquisition Date	28/02/2025 10:48:19
Method	_ESI_low mass 1200series.m	Instrument	compact
Submission Name	af117608dh	ESI	Positive

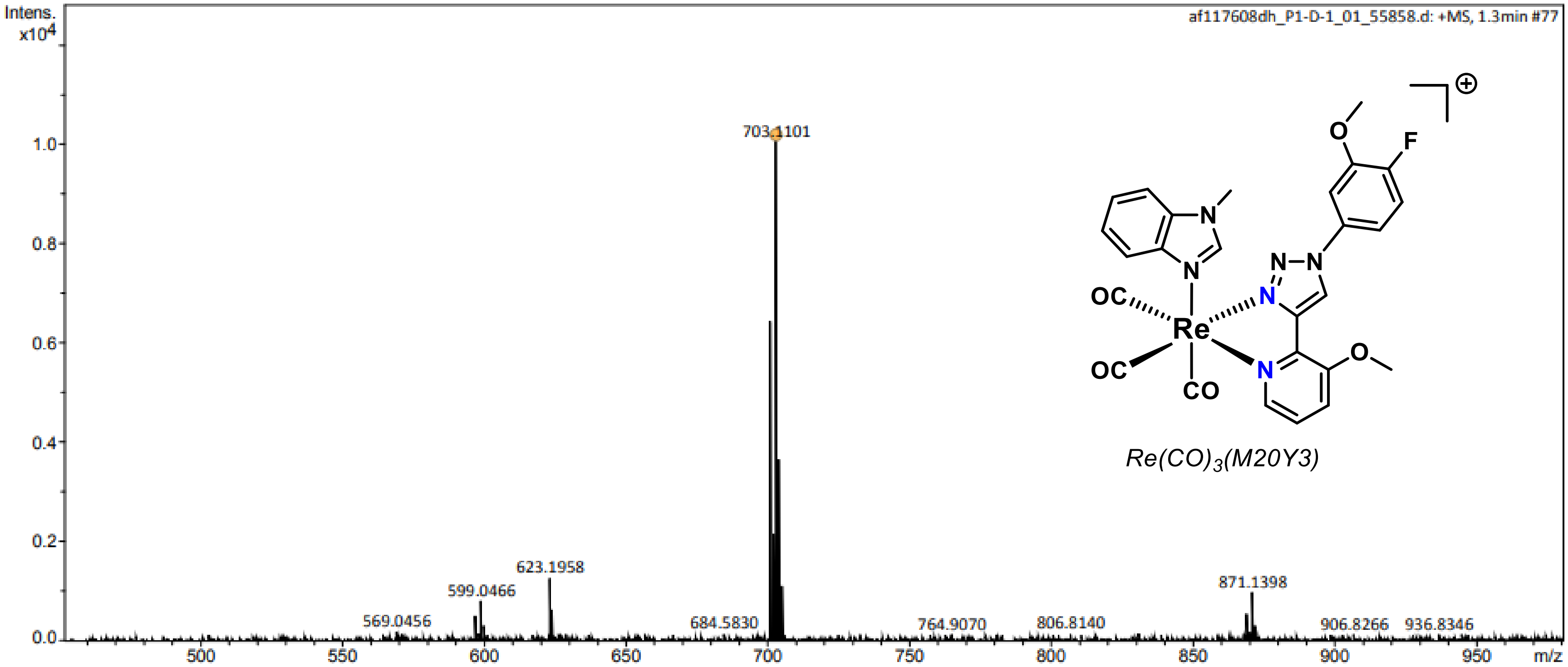

Meas. m/z	#	Ion Formula	m/z	err [ppm]	err [mDa]	mSigma	Mean err [ppm]
703.1101	1	C26H21FN6O5Re	703.1109	1.4	1.0	48.6	2.2

Figure S104: HRMS (ESI positive mode) of Re(CO)$_3$(M20Y3) (lab book ref. DRH-053)

York - Chemistry - Mass Spectrometry Service Report

DRH-056_af117746dh

Analysis Information

Analysis Filename	af117746dh_P1-F-2_01_56006.d	Acquisition Date	05/03/2025 13:37:30
Method	_ESI_low mass 1200series.m	Instrument	compact
Submission Name	af117746dh	ESI	Positive

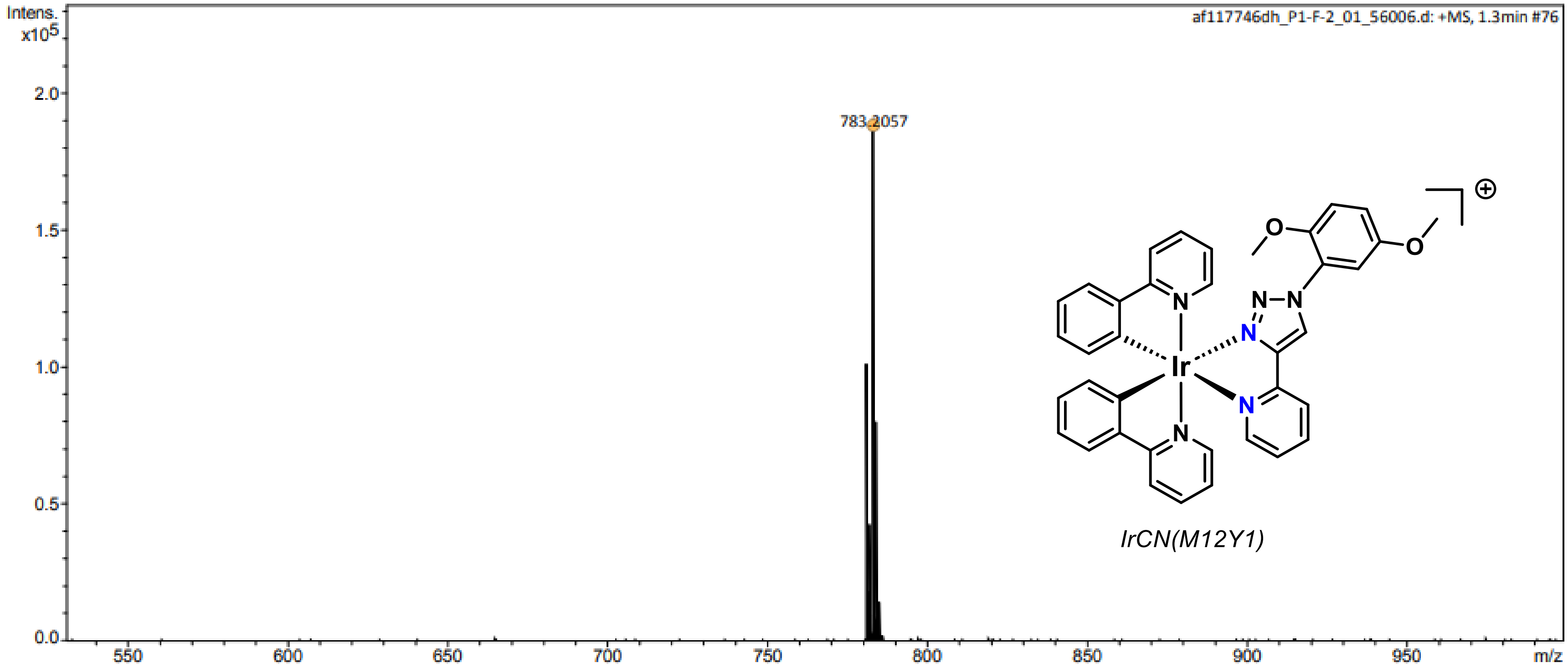

Meas. m/z	#	Ion Formula	m/z	err [ppm]	err [mDa]	mSigma	Mean err [ppm]
783.2057	1	C37H30IrN6O2	783.2054	-0.1	-0.1	12.4	-0.3

Figure S105: HRMS (ESI positive mode) of IrCN(M12Y1) (lab book ref. DRH-056)

York - Chemistry - Mass Spectrometry Service Report

DRH-057_af117796dh

Analysis Information

Analysis Filename	af117796dh_P1-E-7_01_56059.d	Acquisition Date	06/03/2025 12:47:49
Method	_ESI_low mass 1200series.m	Instrument	compact
Submission Name	af117796dh	ESI	Positive

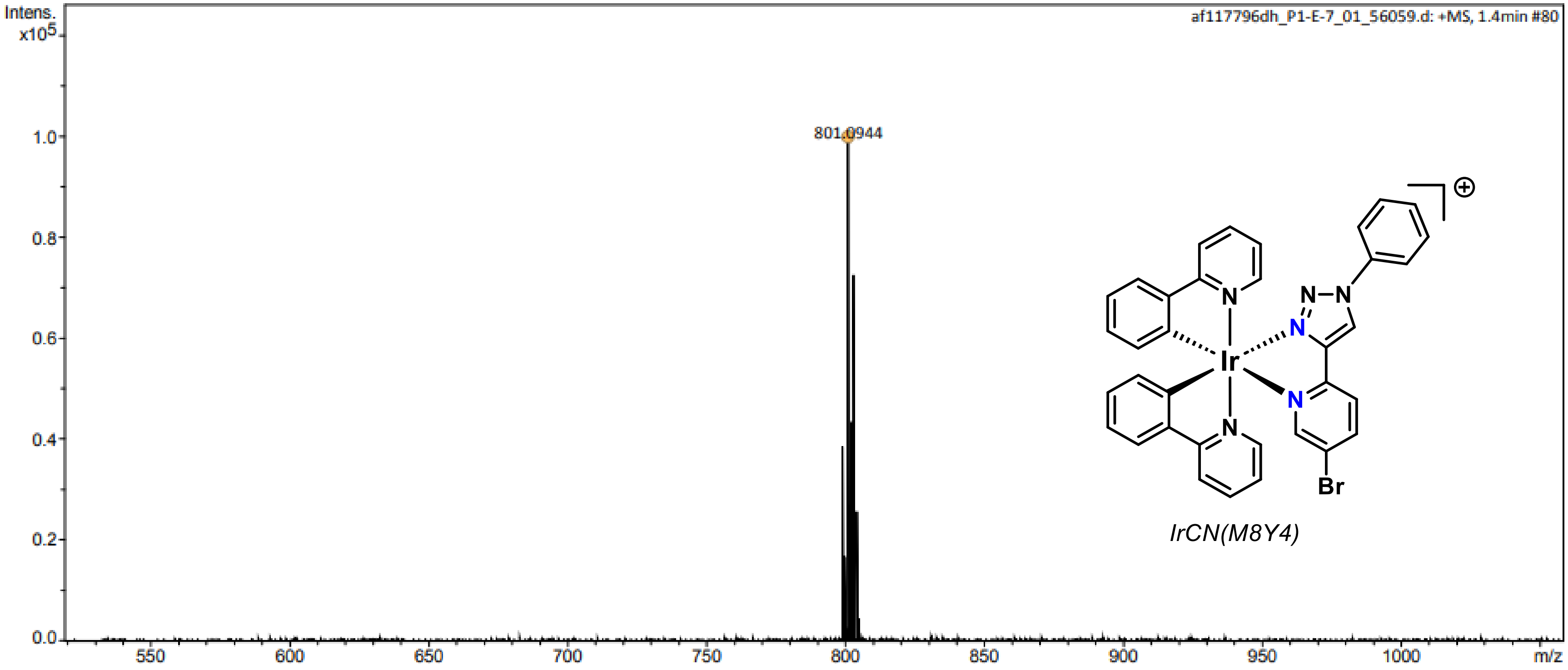

Meas. m/z	#	Ion Formula	m/z	err [ppm]	err [mDa]	mSigma	Mean err [ppm]
801.0944	1	C35H25BrIrN6	801.0948	-1.3	-1.0	31.4	-1.7

Figure S106: HRMS (ESI positive mode) of IrCN(M8Y4) (lab book ref. DRH-057)

York - Chemistry - Mass Spectrometry Service Report

DRH-068-MnM19Y1_af119676dh

Analysis Information

Analysis Filename	af119676dh_P1-C-7_01_58040.d	Acquisition Date	15/05/2025 15:19:54
Method	ESI_low mass_2c1s.m	Instrument	compact
Submission Name	af119676dh	ESI	Positive

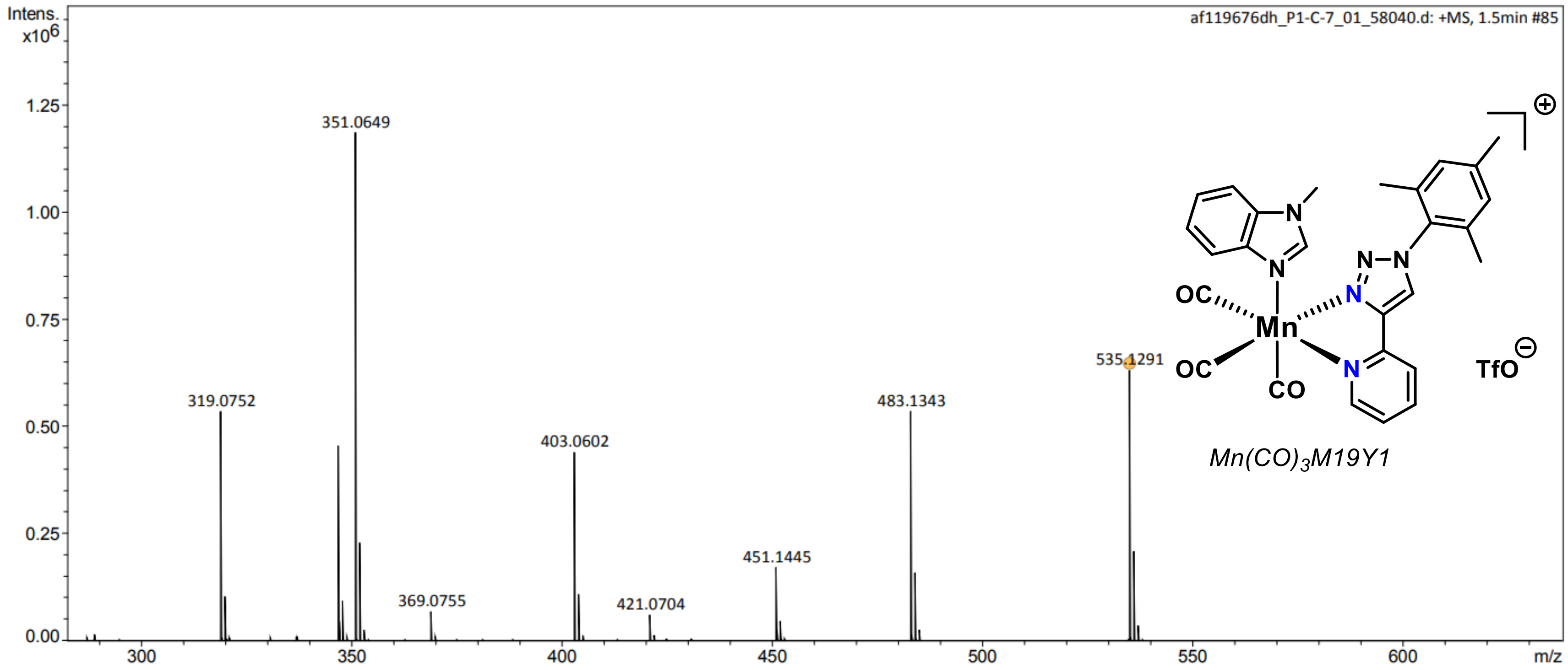

Meas. m/z	#	Ion Formula	m/z	err [ppm]	err [mDa]	mSigma	Mean err [ppm]
535.1291	1	C27H24MnN6O3	535.1285	-1.2	-0.6	6.6	-1.5

Figure S107: HRMS (ESI positive mode) of $Mn(CO)_3$(M19Y1) (lab book ref. DRH-068-1)

York - Chemistry - Mass Spectrometry Service Report

DRH-068-MnM22Y1_af119675dh

Analysis Information

Analysis Filename	af119675dh_P1-C-6_01_58039.d	Acquisition Date	15/05/2025 15:01:32
Method	ESI_low mass_2c1s.m	Instrument	compact
Submission Name	af119675dh	ESI	Positive

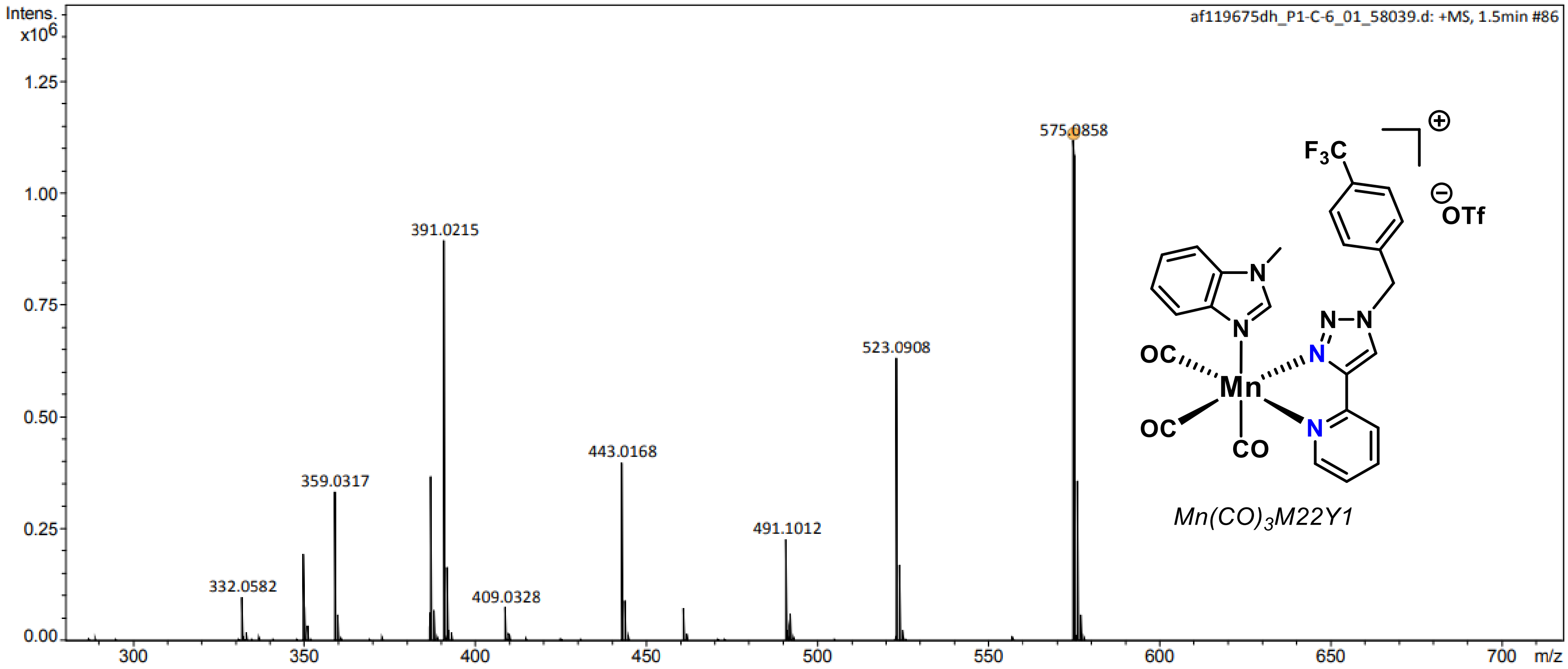

Meas. m/z	#	Ion Formula	m/z	err [ppm]	err [mDa]	mSigma	Mean err [ppm]
575.0858	1	C26H19F3MnN6O3	575.0846	-2.1	-1.2	8.1	-2.3

Figure S108: HRMS (ESI positive mode) of $Mn(CO)_3$(M22Y1) (lab book ref. DRH-068-2)

York - Chemistry - Mass Spectrometry Service Report

DRH-088-2_af121303dh

Analysis Information

Analysis Filename	af121303dh_P1-F-7_01_59806.d	Acquisition Date	26/08/2025 10:26:41
Method	ESI_low mass_2c1s.m	Instrument	compact
Submission Name	af121303dh	ESI	Positive

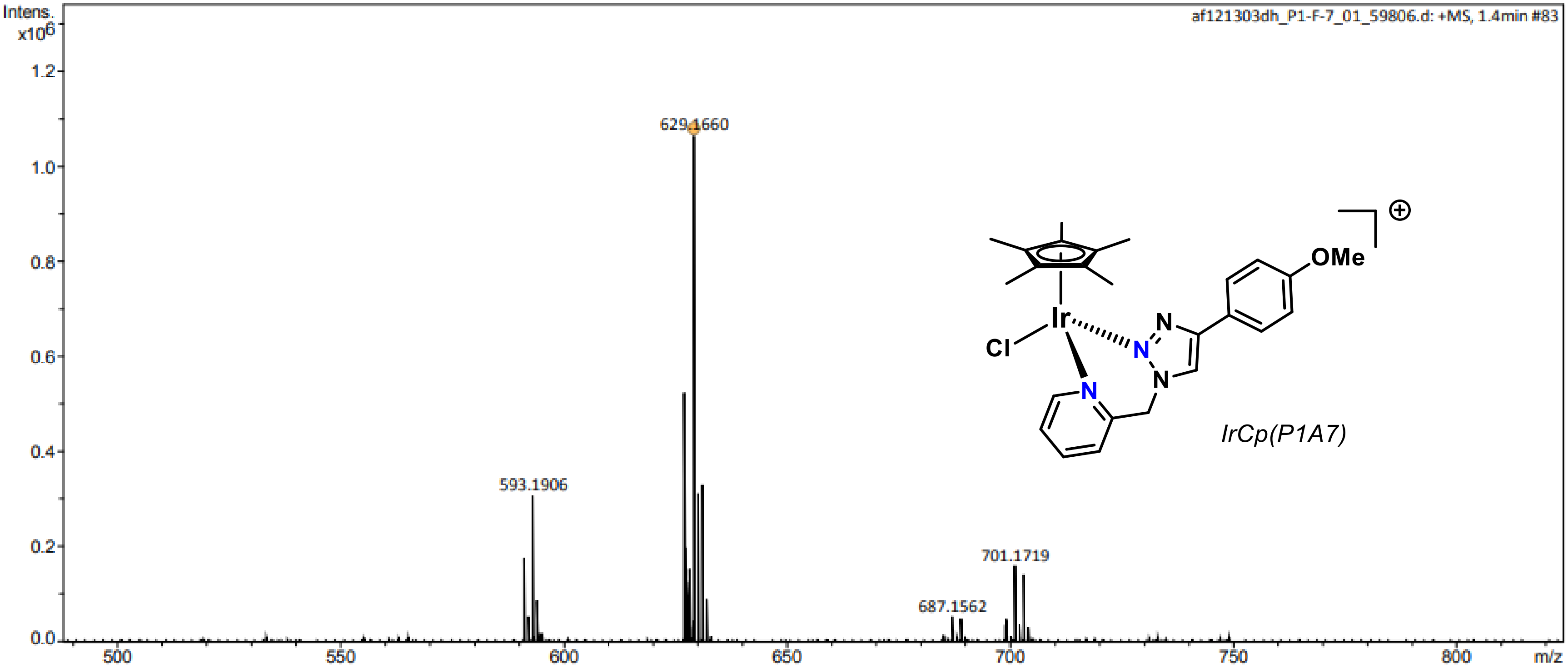

Meas. m/z	#	Ion Formula	m/z	err [ppm]	err [mDa]	mSigma	Mean err [ppm]
629.1660	1	C25H29ClIrN4O	629.1654	-2.2	-1.4	4.6	-2.6

Figure S109: HRMS (ESI positive mode) of IrCp*(P1A7) (lab book ref. DRH-088-2)

York - Chemistry - Mass Spectrometry Service Report

DRH-088-4_af121304dh

Analysis Information

Analysis Filename	af121304dh_P1-F-8_01_59807.d	Acquisition Date	26/08/2025 10:29:39
Method	ESI_low mass_2c1s.m	Instrument	compact
Submission Name	af121304dh	ESI	Positive

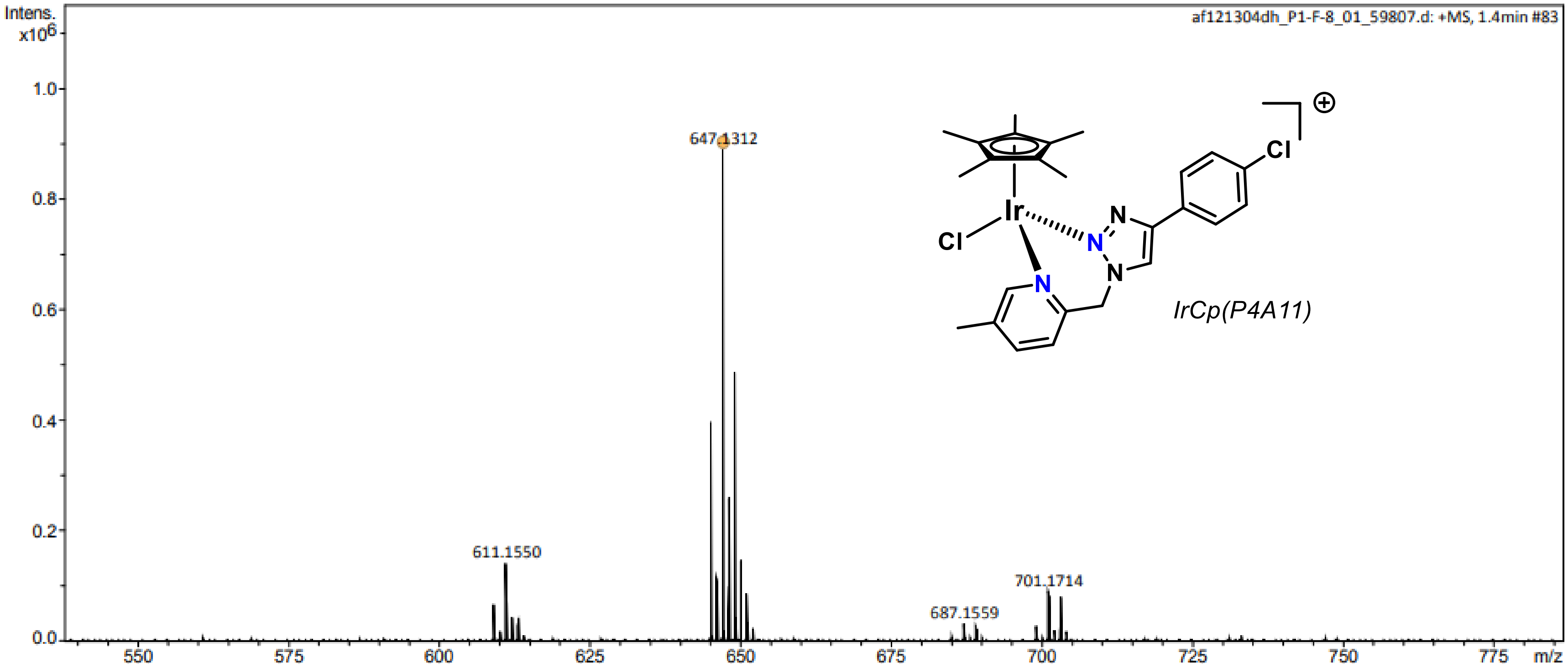

Meas. m/z	#	Ion Formula	m/z	err [ppm]	err [mDa]	mSigma	Mean err [ppm]
647.1312	1	C25H28Cl2IrN4	647.1315	-1.7	-1.1	11.4	-2.1

Figure S110: HRMS (ESI positive mode) of IrCp*(P4A11) (lab book ref. DRH-088-3. This sample was erroneously labelled as DRH-088-4)

York - Chemistry - Mass Spectrometry Service Report

DRH-008_af112223dh

Analysis Information

Analysis Filename	af112223dh_P1-E-7_01_50465.d	Acquisition Date	14/06/2024 10:16:44
Method	ESI_low mass_2c1s.m	Instrument	compact
Submission Name	af112223dh	ESI	Positive

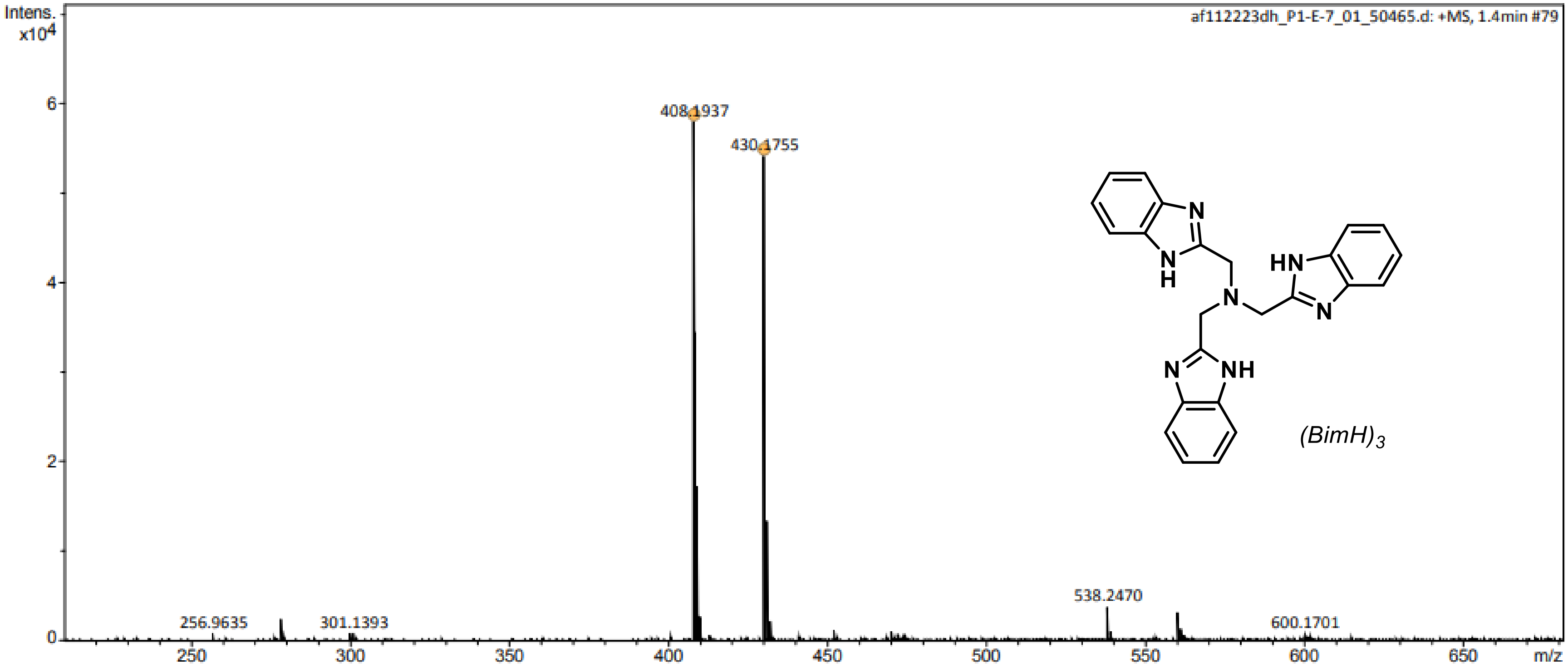

Meas. m/z	#	Ion Formula	m/z	err [ppm]	err [mDa]	mSigma	Mean err [ppm]
408.1937	1	C24H22N7	408.1931	-1.4	-0.6	7.3	-1.3
430.1755	1	C24H21N7Na	430.1751	-1.0	-0.4	23.3	-0.4

Figure S111: HRMS (ESI positive mode) of $(BimH)_3$ (lab book ref. DRH-008)

York - Chemistry - Mass Spectrometry Service Report

DRH-030_af114818dh

Analysis Information

Analysis Filename	af114818dh_P1-F-1_01_53048.d	Acquisition Date	24/10/2024 09:49:57
Method	_ESI_low mass 1200series.m	Instrument	compact
Submission Name	af114818dh	ESI	Positive

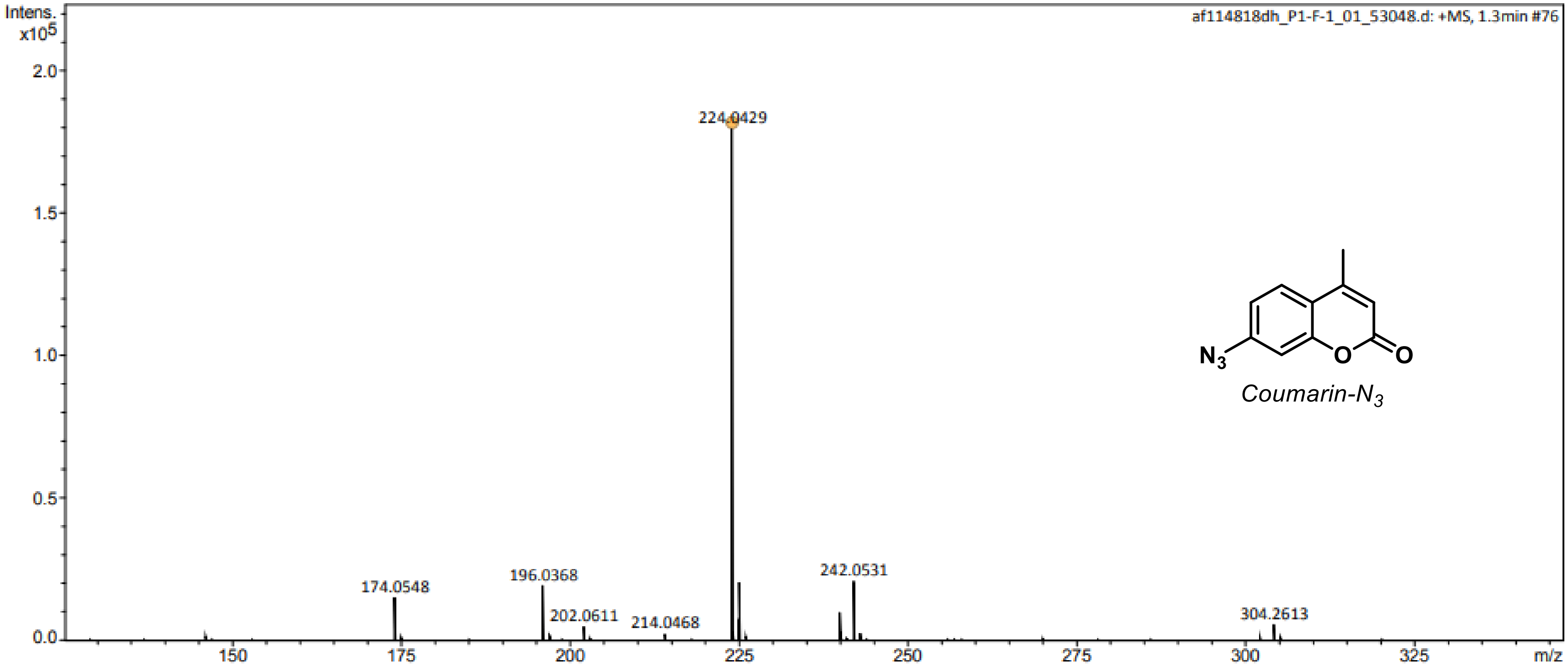

Meas. m/z	#	Ion Formula	m/z	err [ppm]	err [mDa]	mSigma	Mean err [ppm]
224.0429	1	C10H7N3NaO2	224.0430	0.6	0.1	4.1	0.4

Figure S112: HRMS (ESI positive mode) of Coumarin-N_3 (lab book ref. DRH-030)

8. LCMS traces for Organic and Inorganic Compounds

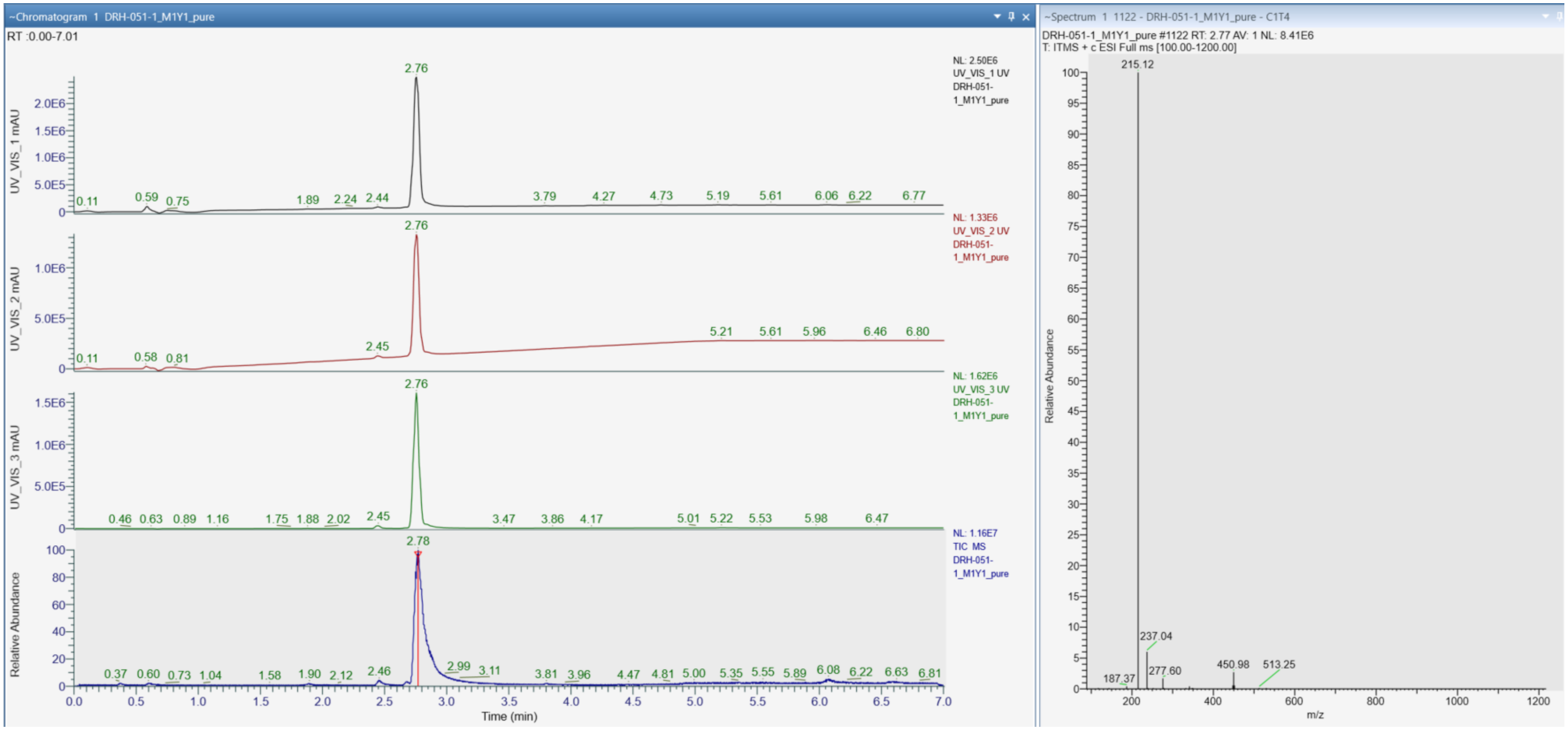

Figure S113: LCMS trace (7 min method) of M1Y1 (lab book ref. DRH-051-1)

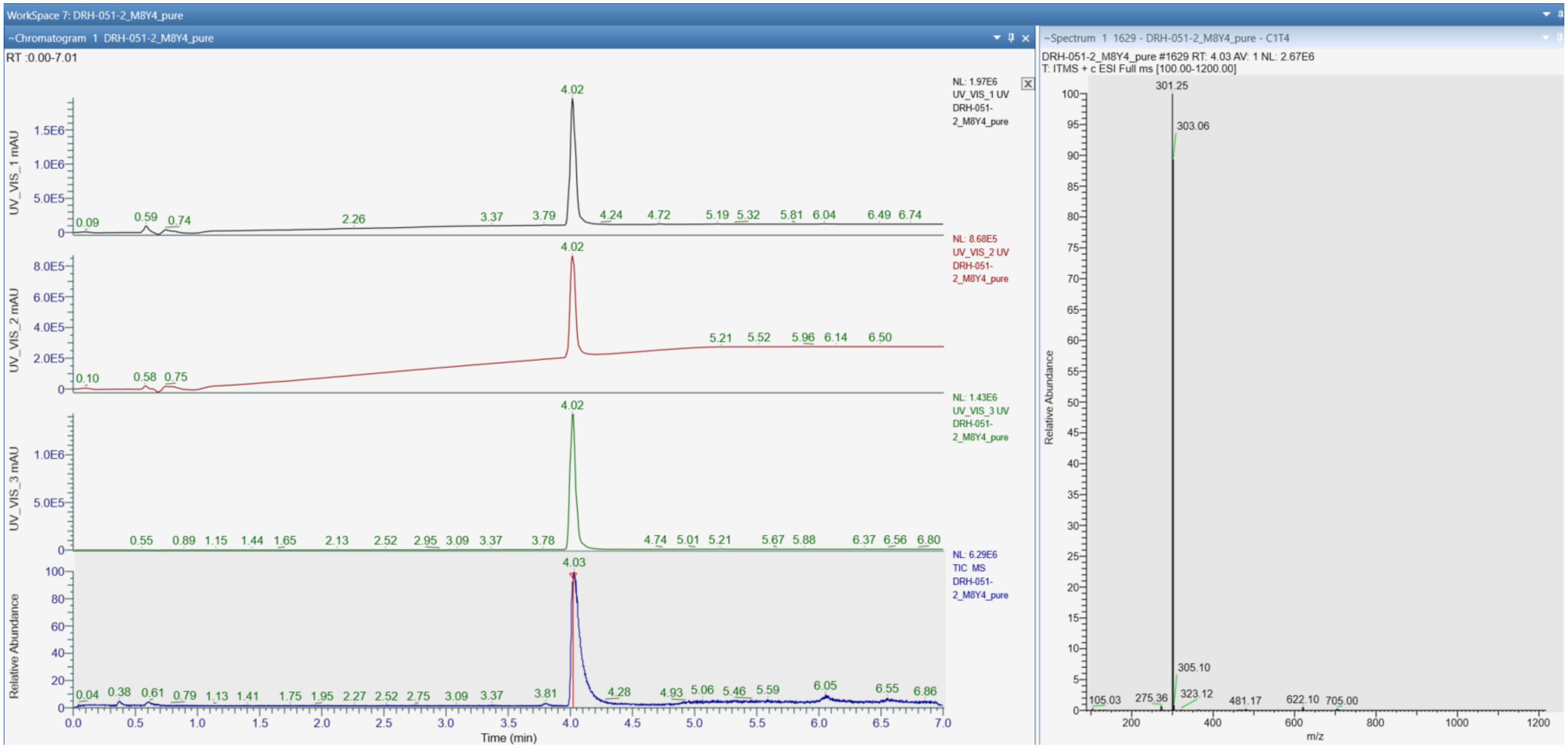

Figure S114: LCMS trace (7 min method) of M8Y4 (lab book ref. DRH-051-2)

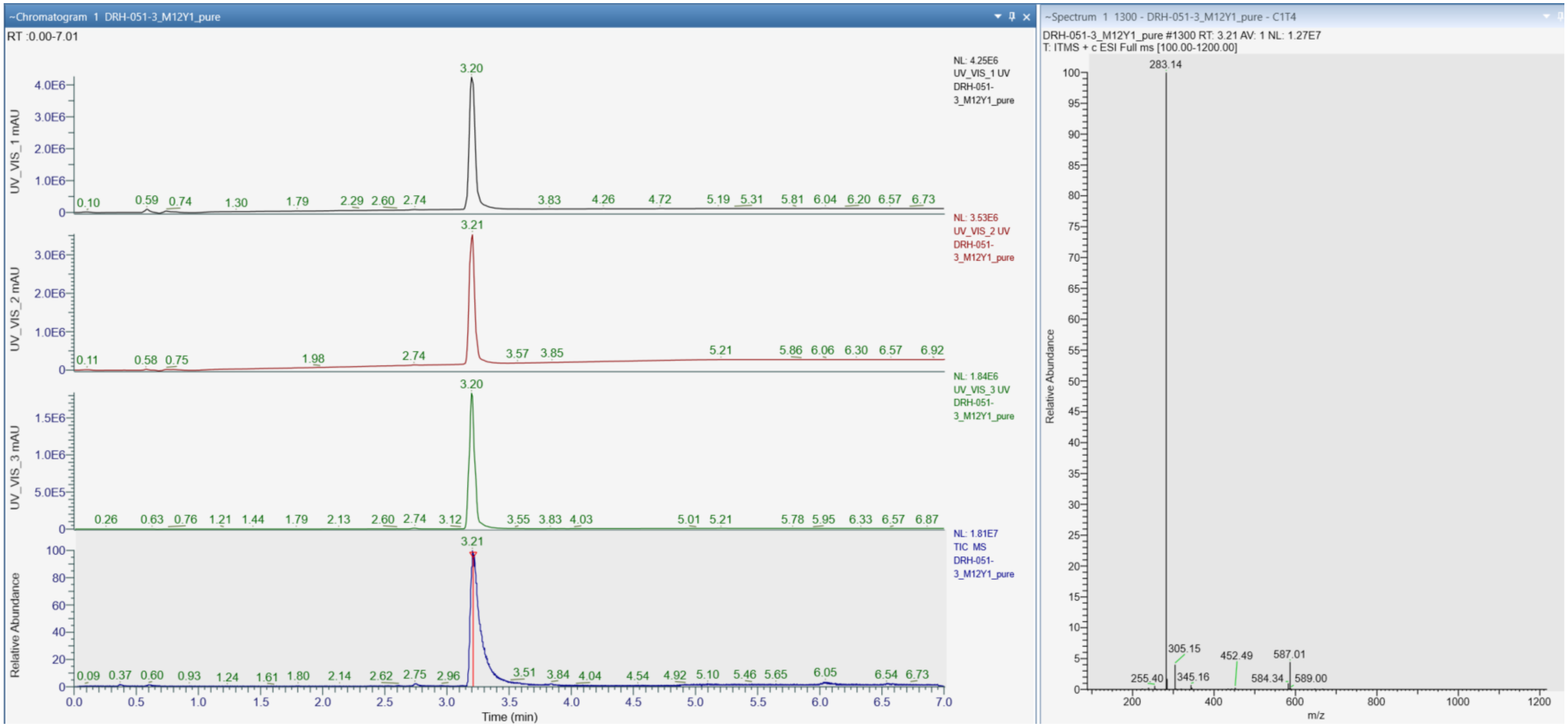

Figure S115: LCMS trace (7 min method) of M12Y1 (lab book ref. DRH-051-3)

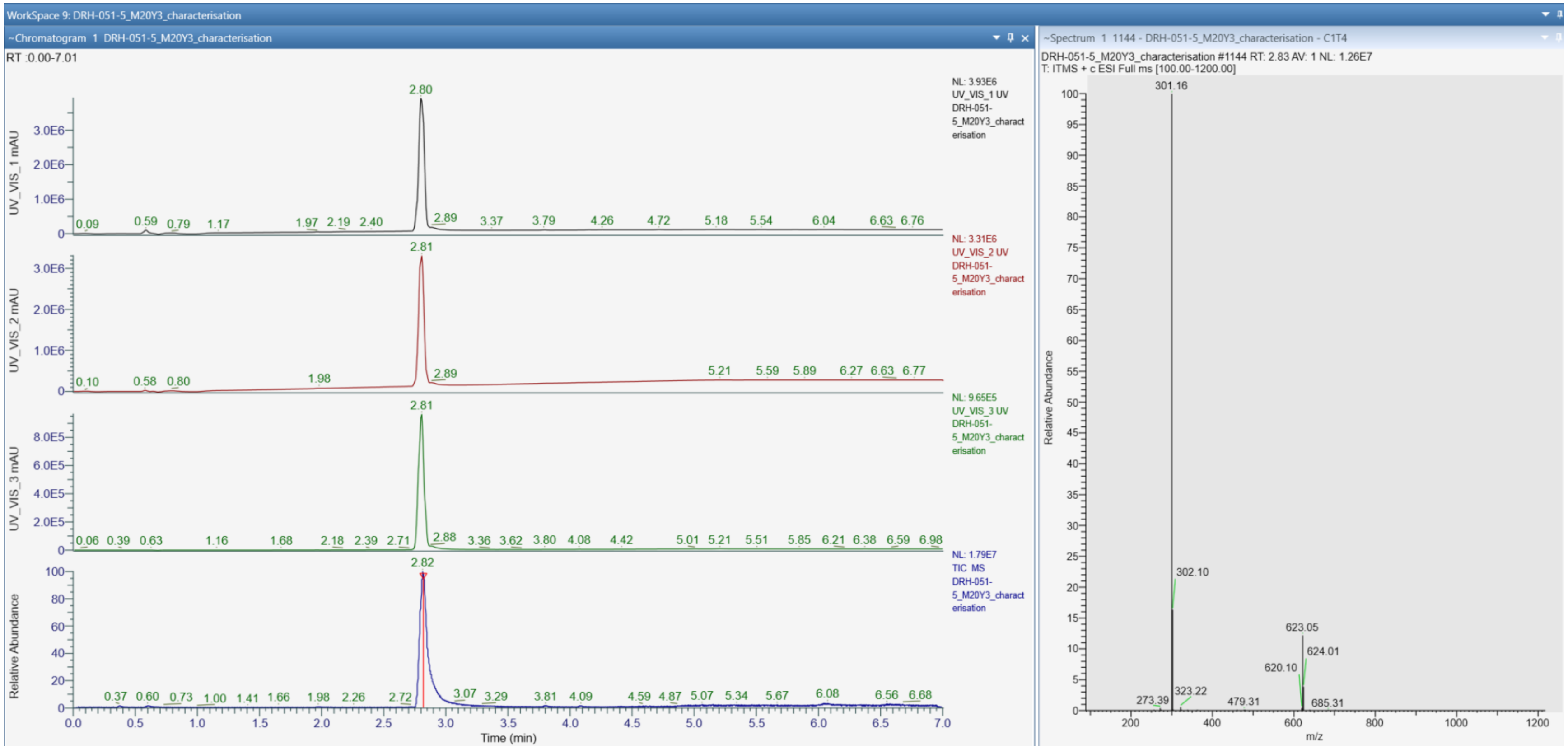

Figure S116: LCMS trace (7 min method) of M20Y3 (lab book ref. DRH-051-5)

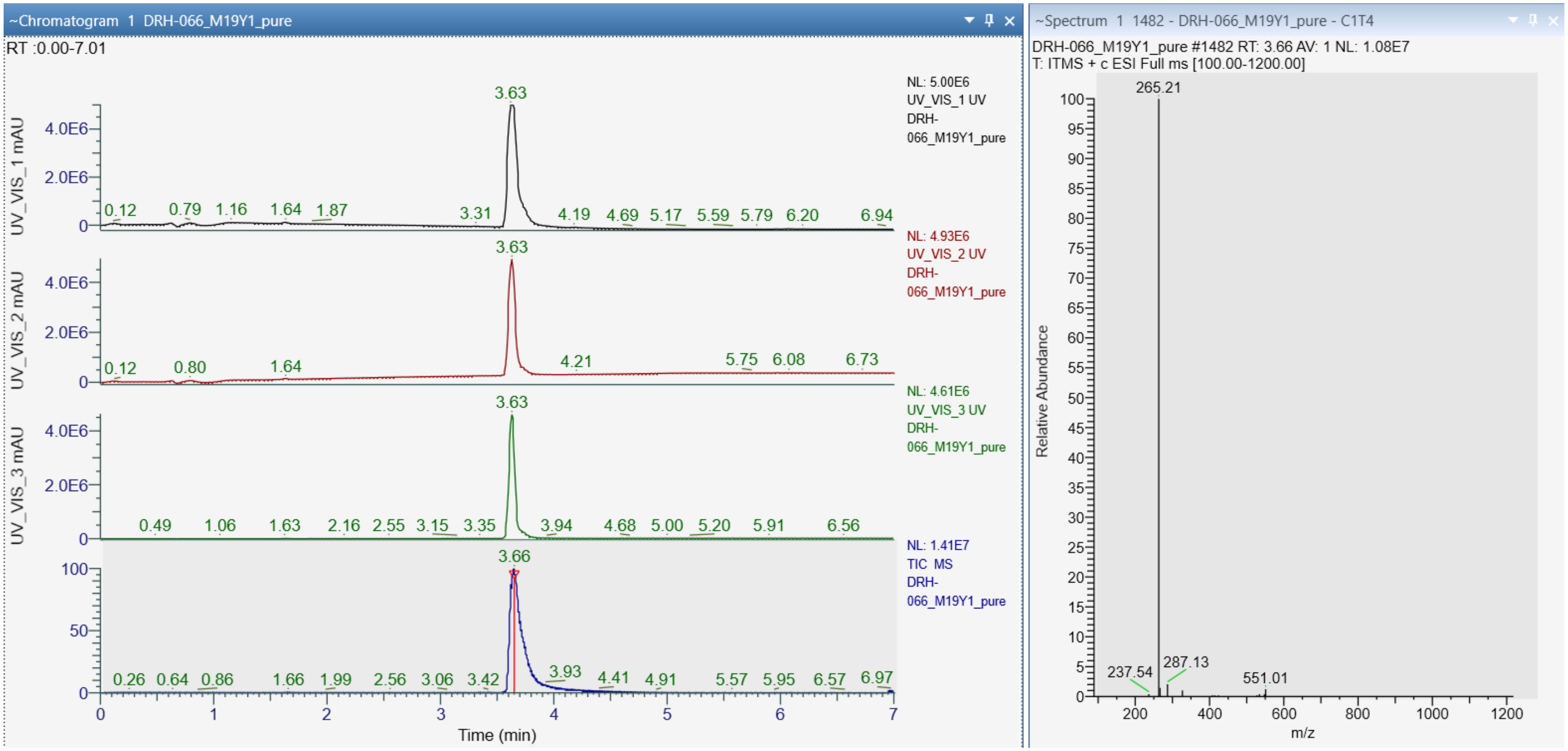

Figure S117: LCMS trace (7 min method) of M19Y1 (lab book ref. DRH-066-1)

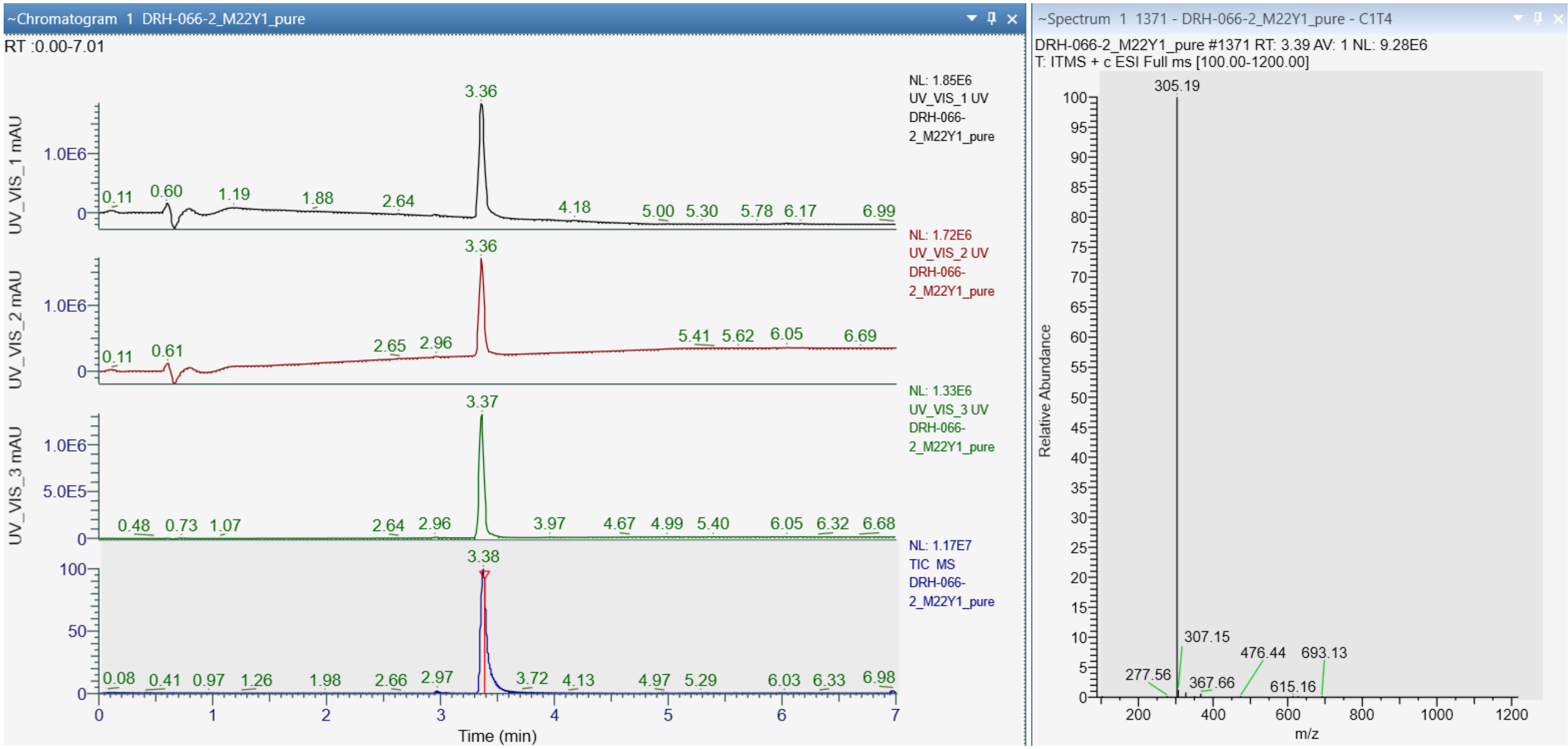

Figure S118: LCMS trace (7 min method) of M22Y1 (lab book ref. DRH-066-2)

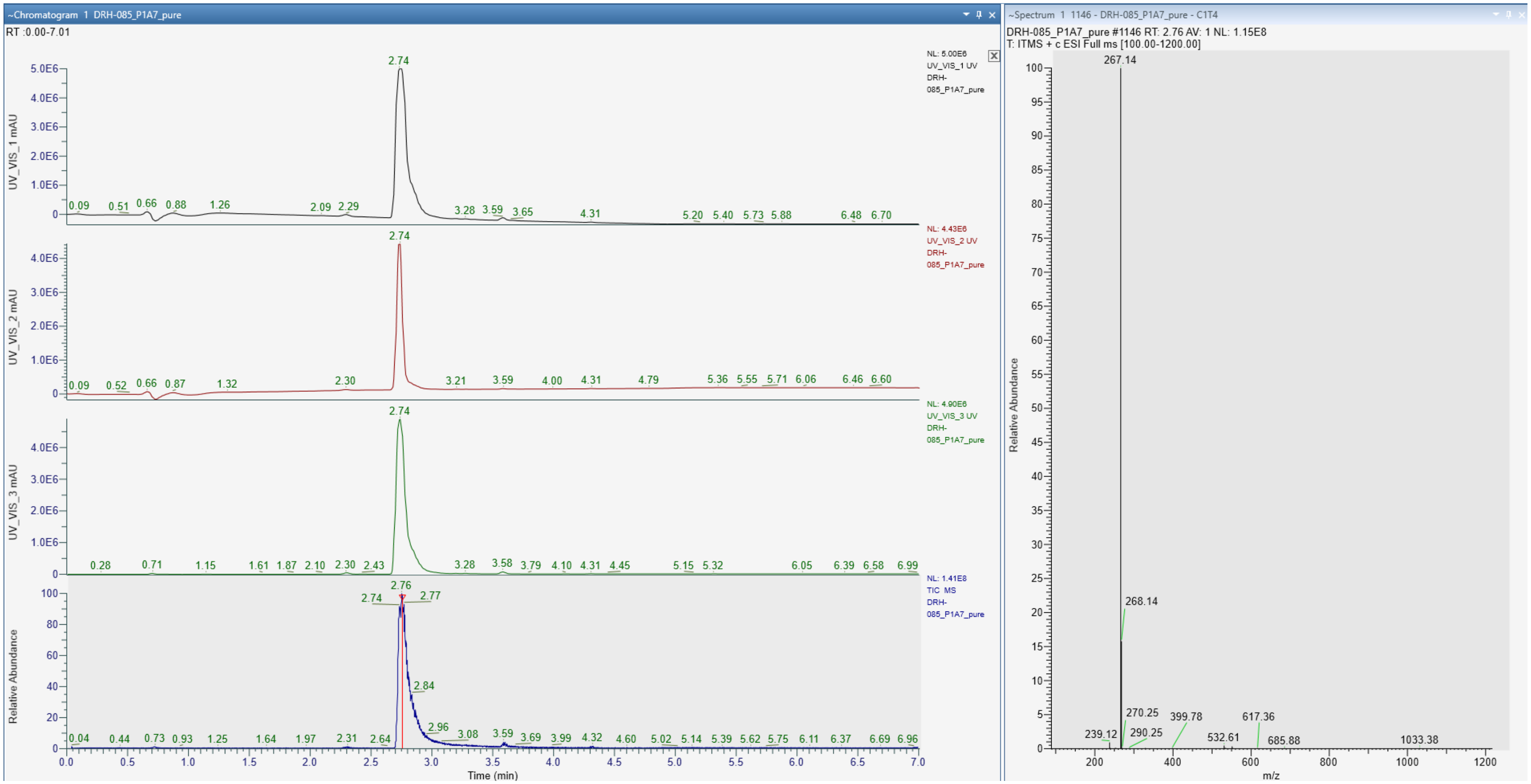

Figure S119: LCMS trace (7 min method) of P1A7 (lab book ref. DRH-085-2)

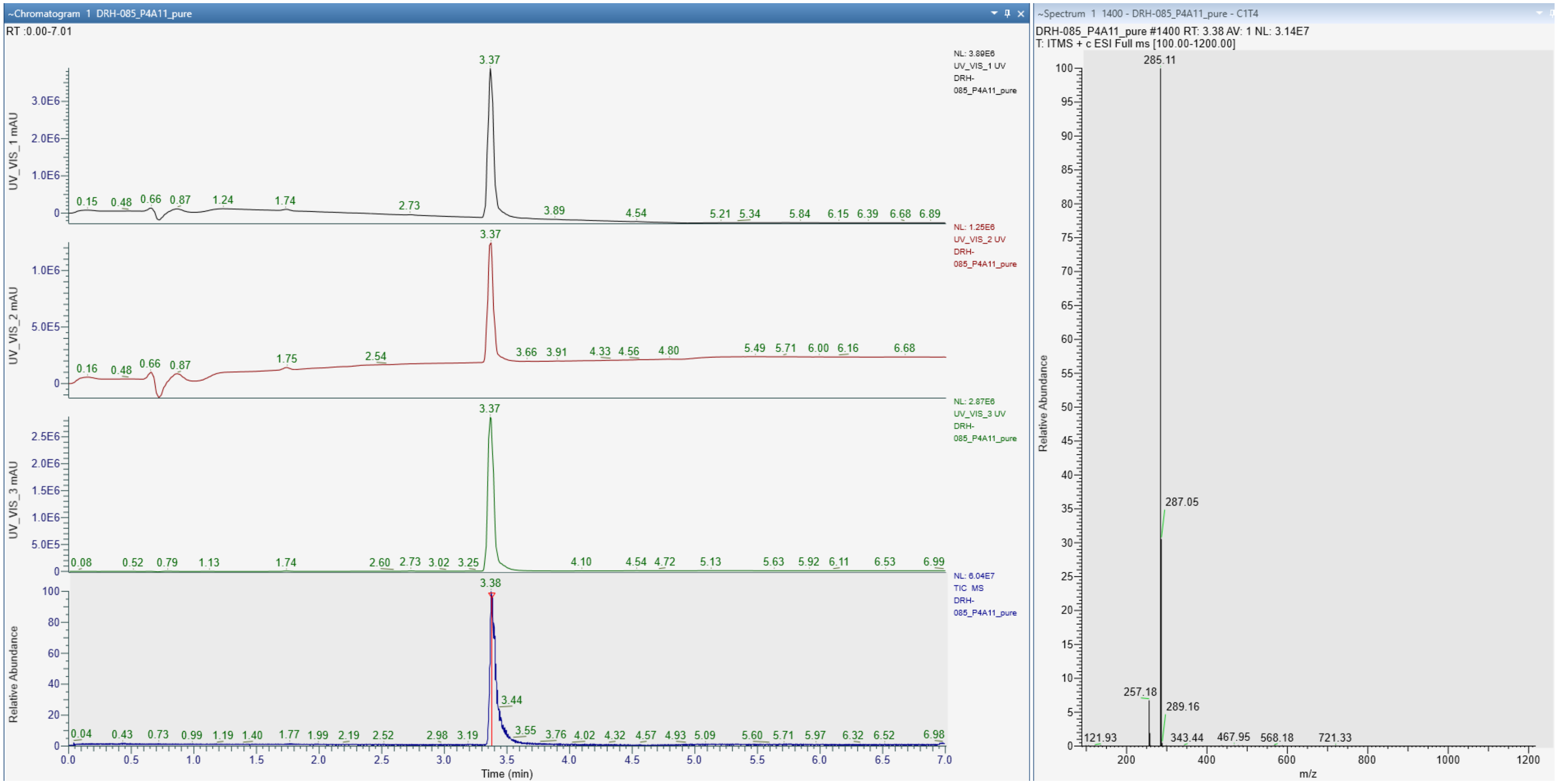

Figure S120: LCMS trace (7 min method) of P4A11 (lab book ref. DRH-085-3)

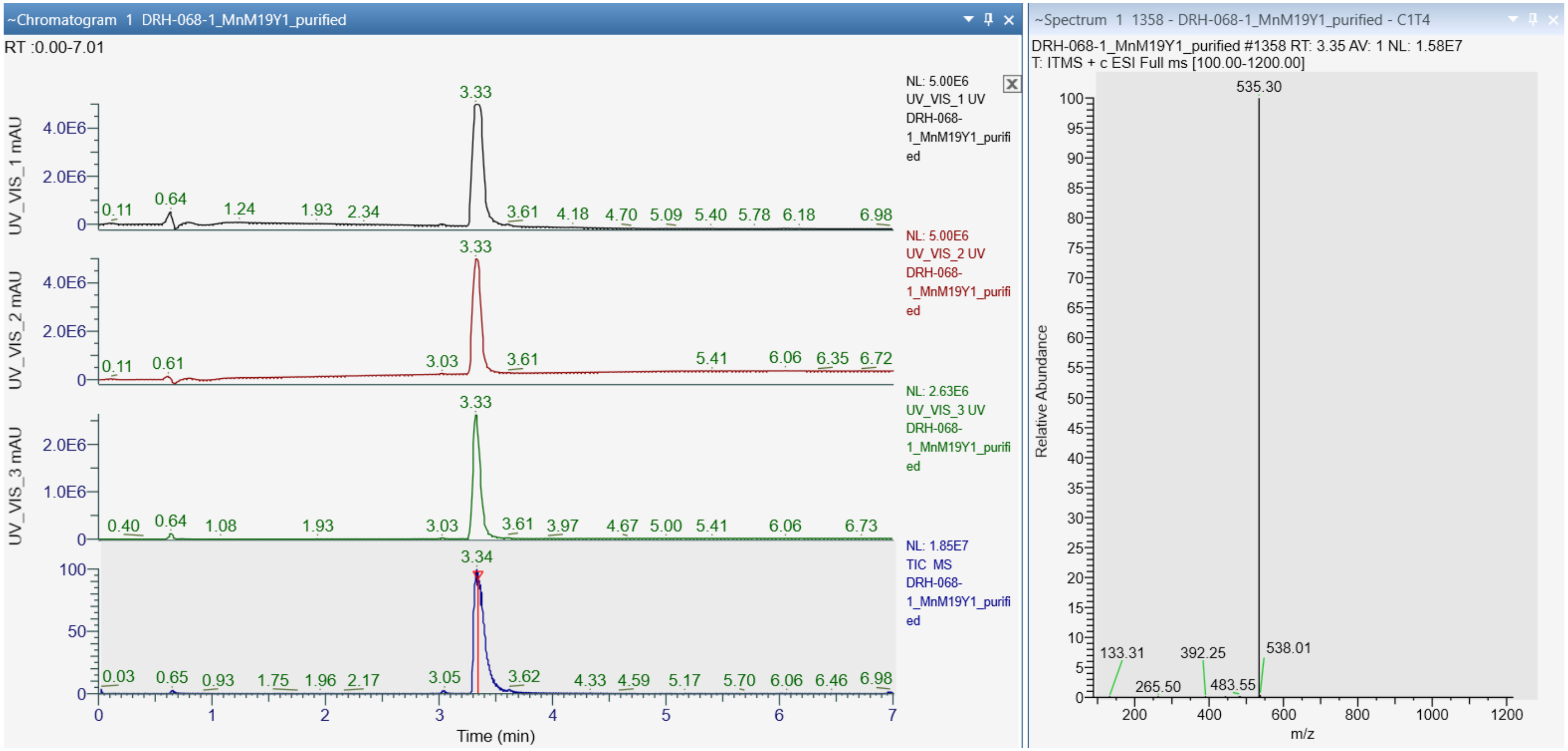

Figure S121: LCMS trace (7 min method) of Mn(CO)$_3$M19Y1 (lab book ref. DRH-068-1)

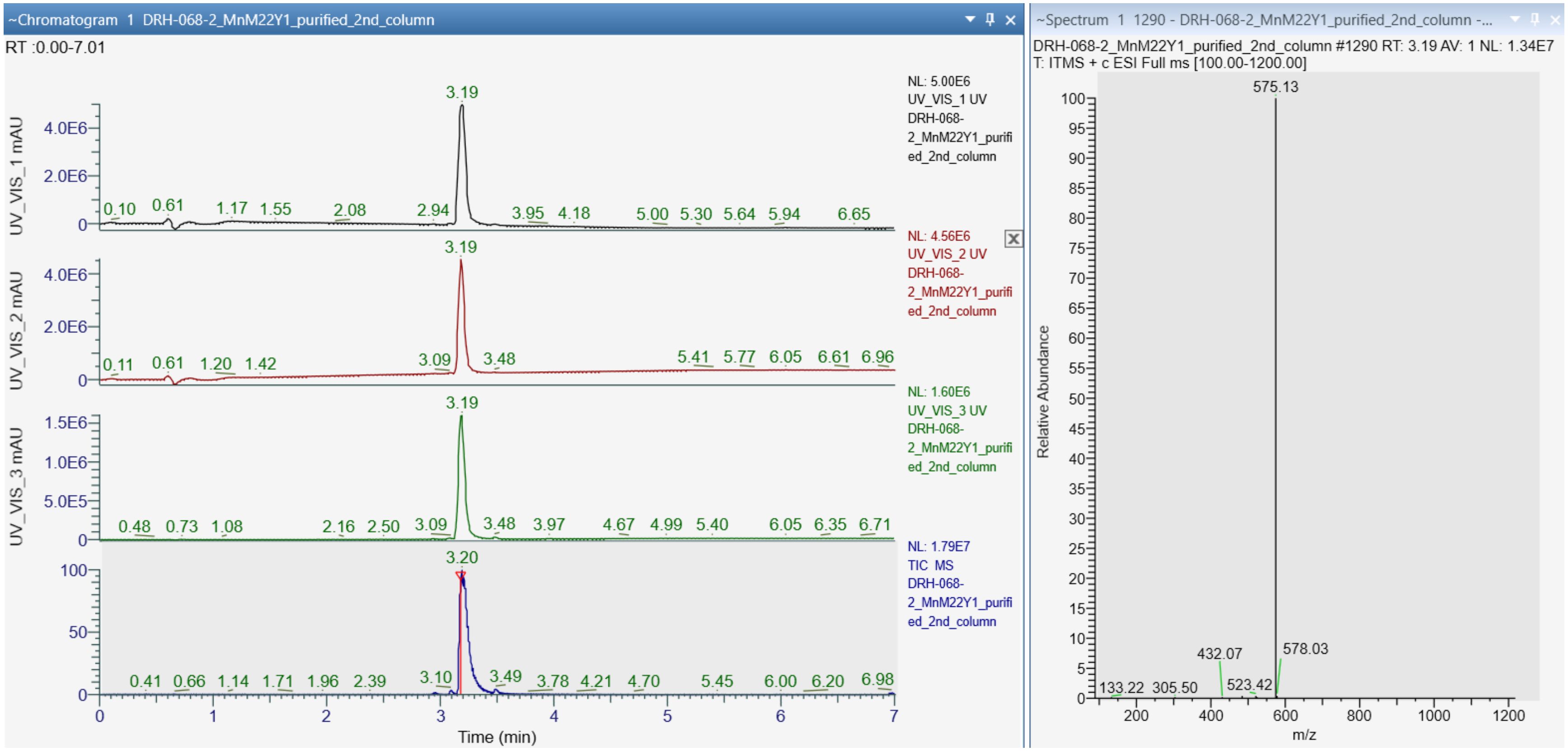

Figure S122: LCMS trace (7 min method) of $Mn(CO)_3$M22Y1 (lab book ref. DRH-068-2)

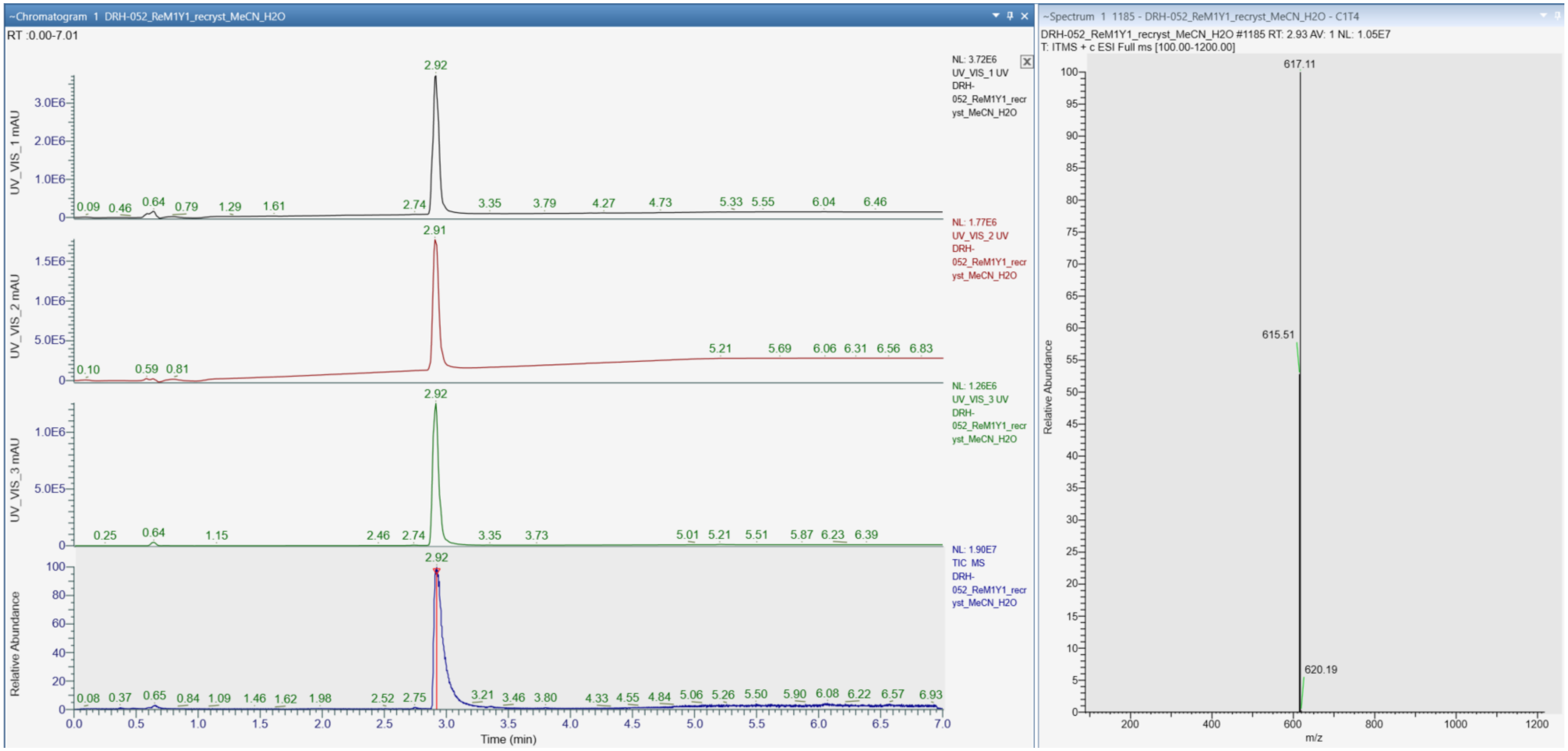

Figure S123: LCMS trace (7 min method) of Re(CO)$_3$(M1Y1) (lab book ref. DRH-052)

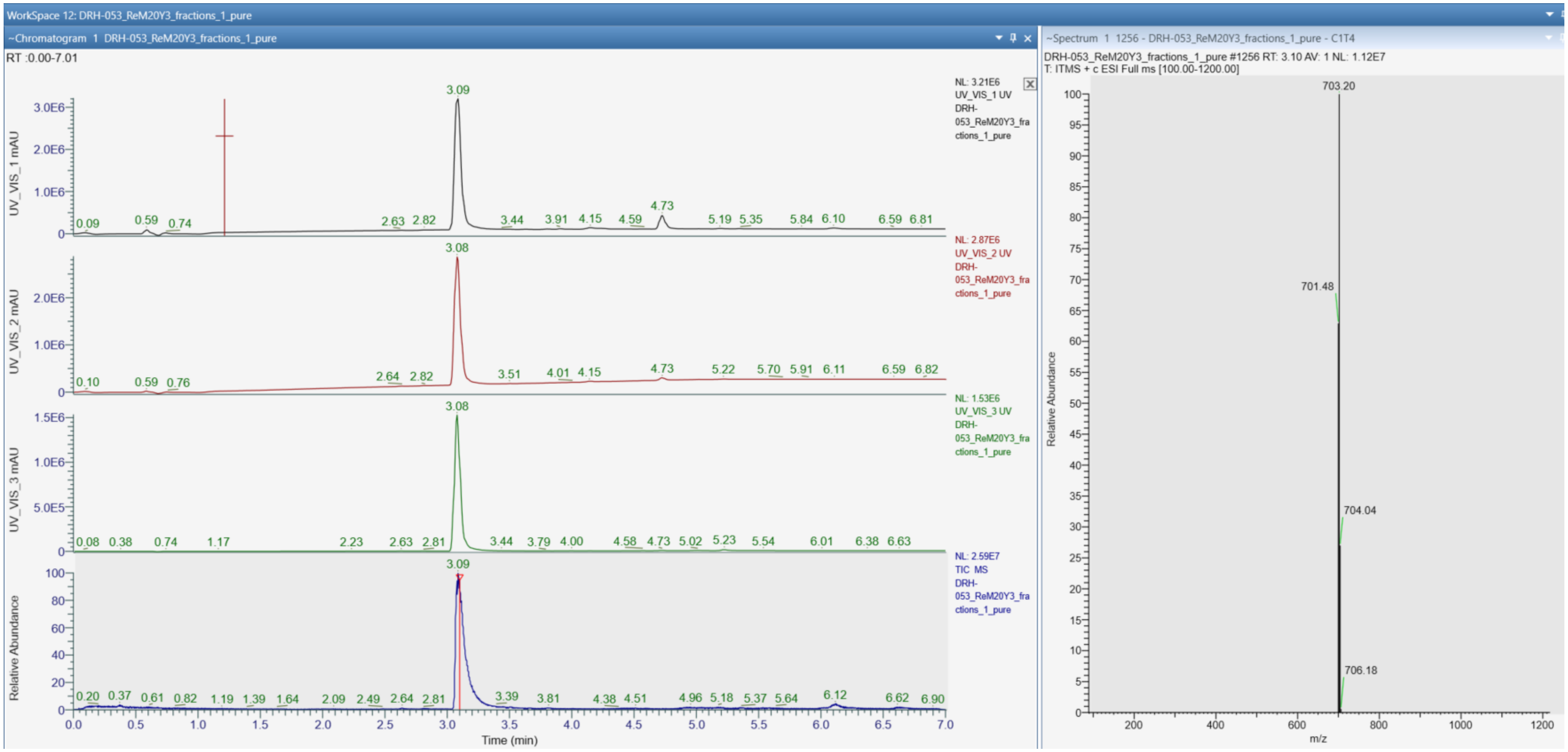

Figure S124: LCMS trace (7 min method) of Re(CO)$_3$(M20Y3) (lab book ref. DRH-053)

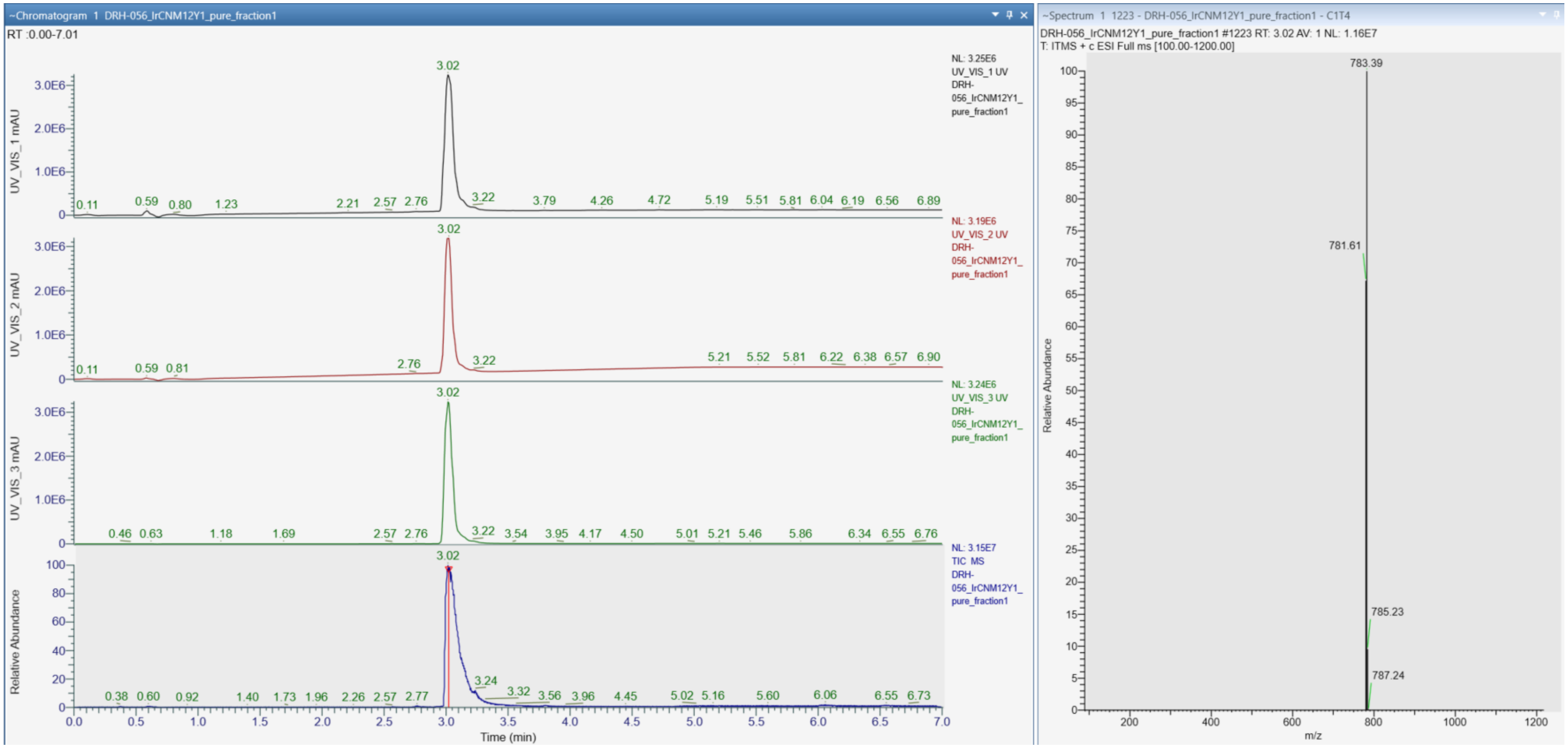

Figure S125: LCMS trace (7 min method) of IrCN(M12Y1) (lab book ref. DRH-056)

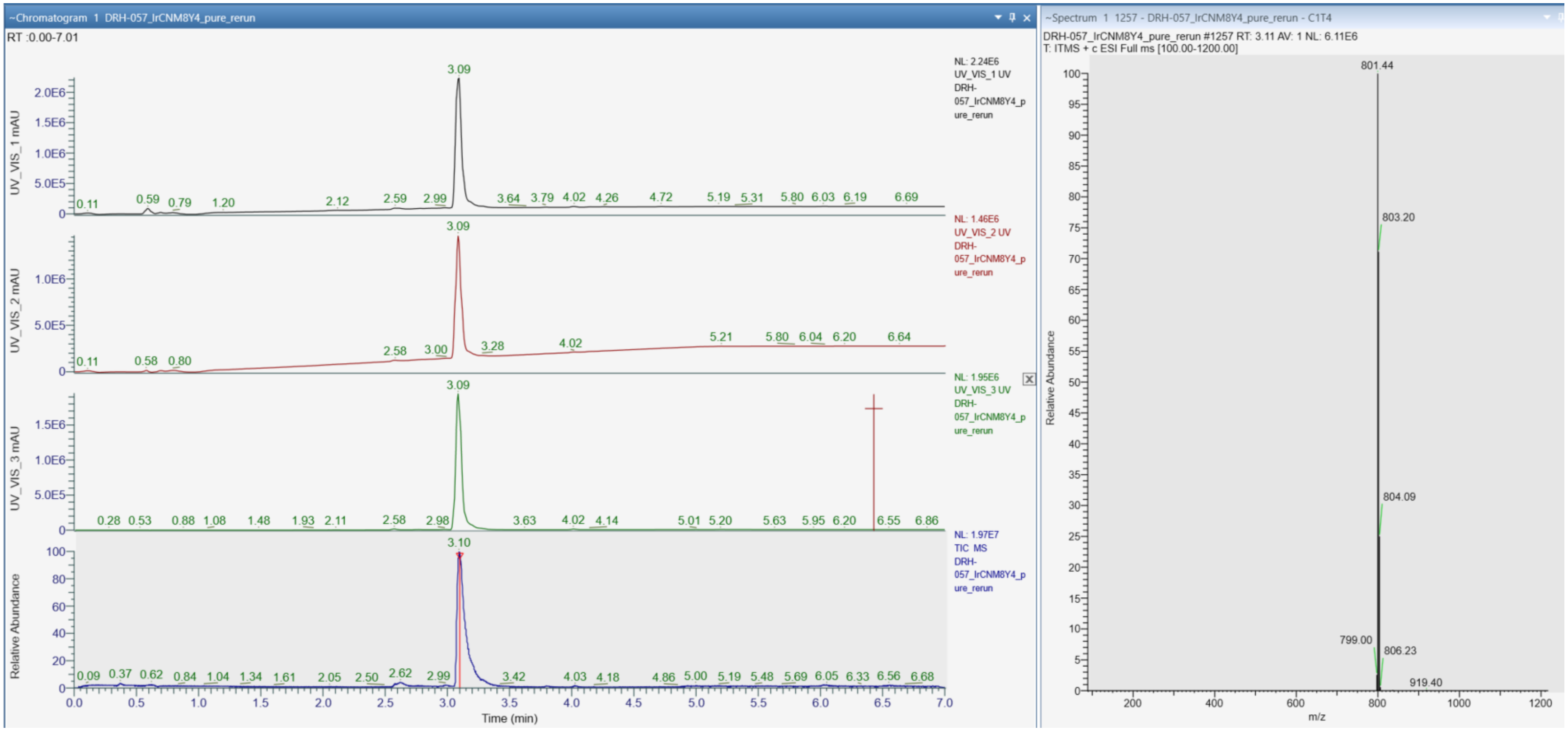

Figure S126: LCMS trace (7 min method) of IrCN(M8Y4) (lab book ref. DRH-057)

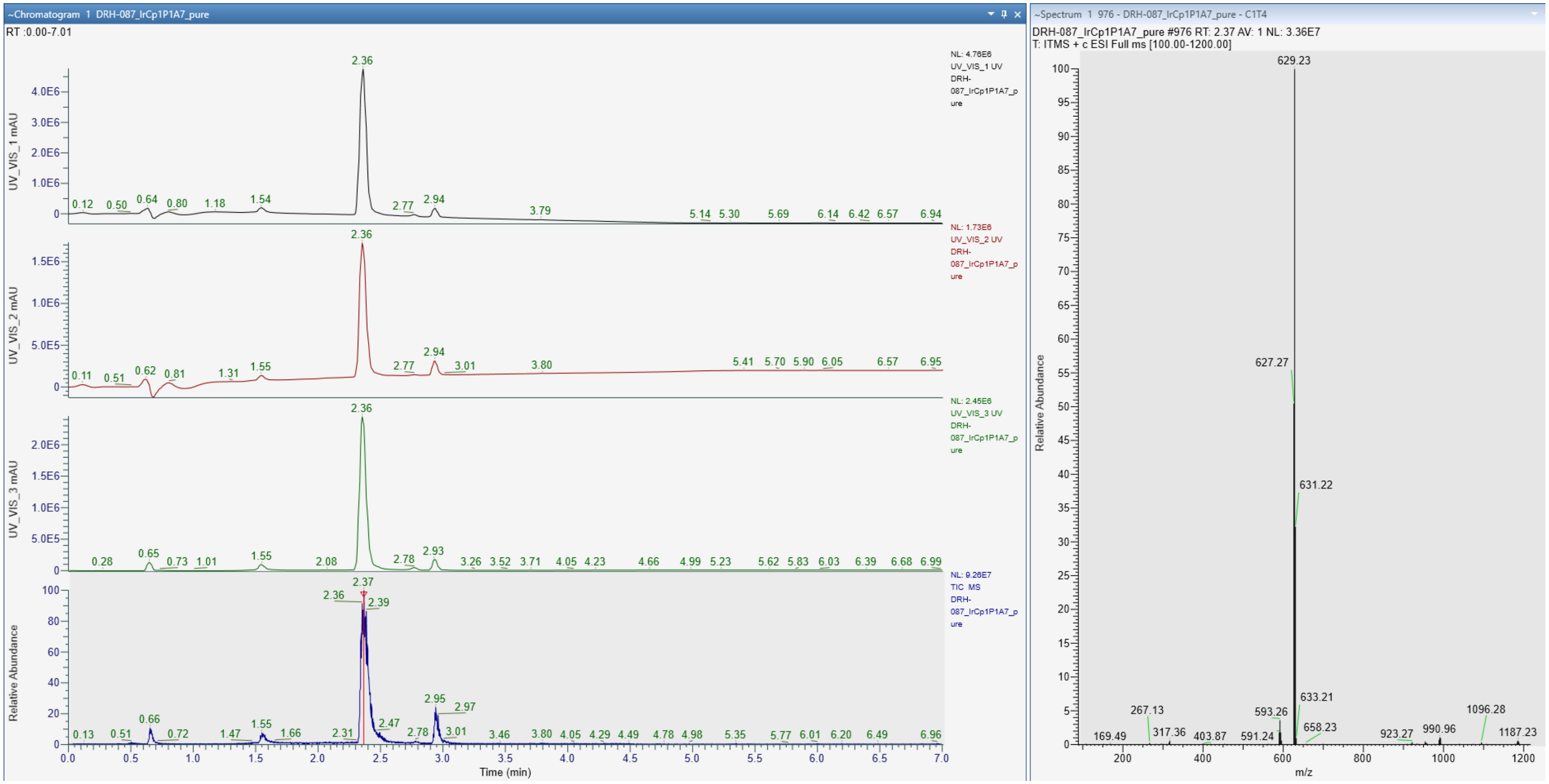

Figure S127: LCMS trace (7 min method) of IrCp*(P1A7) (lab book ref. DRH-088-2)

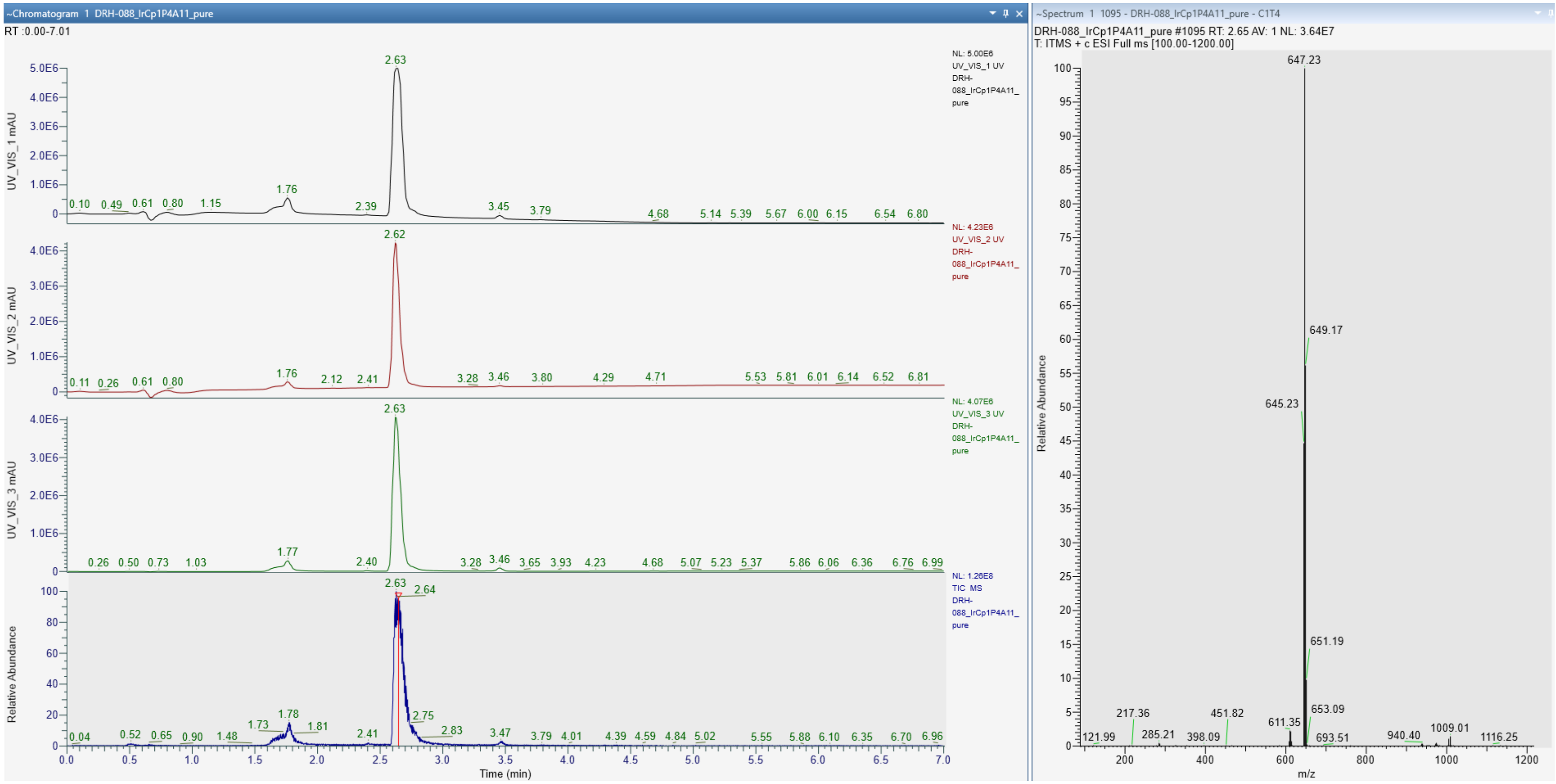

Figure 128: LCMS trace (7 min method) of IrCp*(P4A11) (lab book ref. DRH-088-3)

9. FTIR data for Organic and Inorganic Compounds

PerkinElmer Spectrum Version 10.5.4
06 March 2025 14:03

Analyst Administrator
Date 06 March 2025 14:03

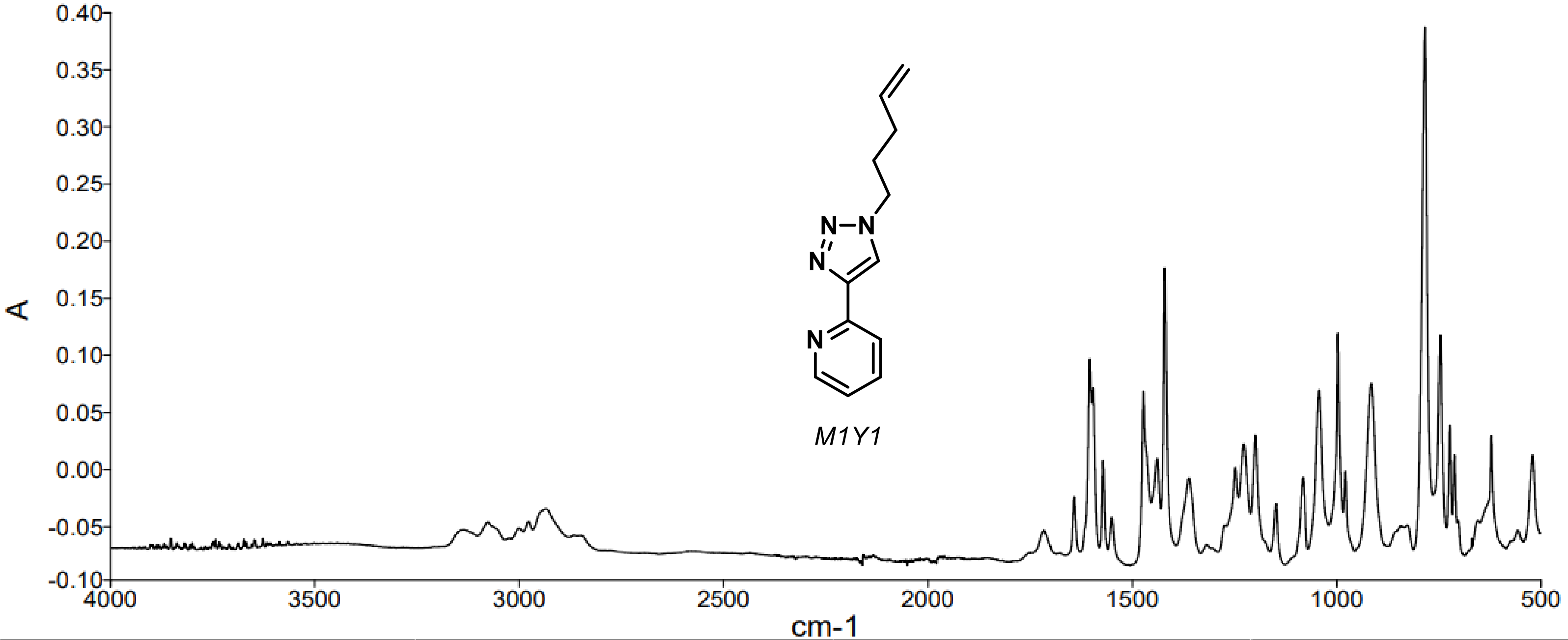

Sample Name	Description	Quality Checks	Number of peaks
DRH-051-1_M1Y1	Sample 158 By Administrator Date Thursday, March 06 2025	The Quality Checks give rise to a Baseline High warning for the sample.	32

Figure S129: FTIR (ATIR) spectrum of M1Y1 (lab book ref. DRH-051-1)

PerkinElmer Spectrum Version 10.5.4
06 March 2025 14:11

Analyst Administrator
Date 06 March 2025 14:11

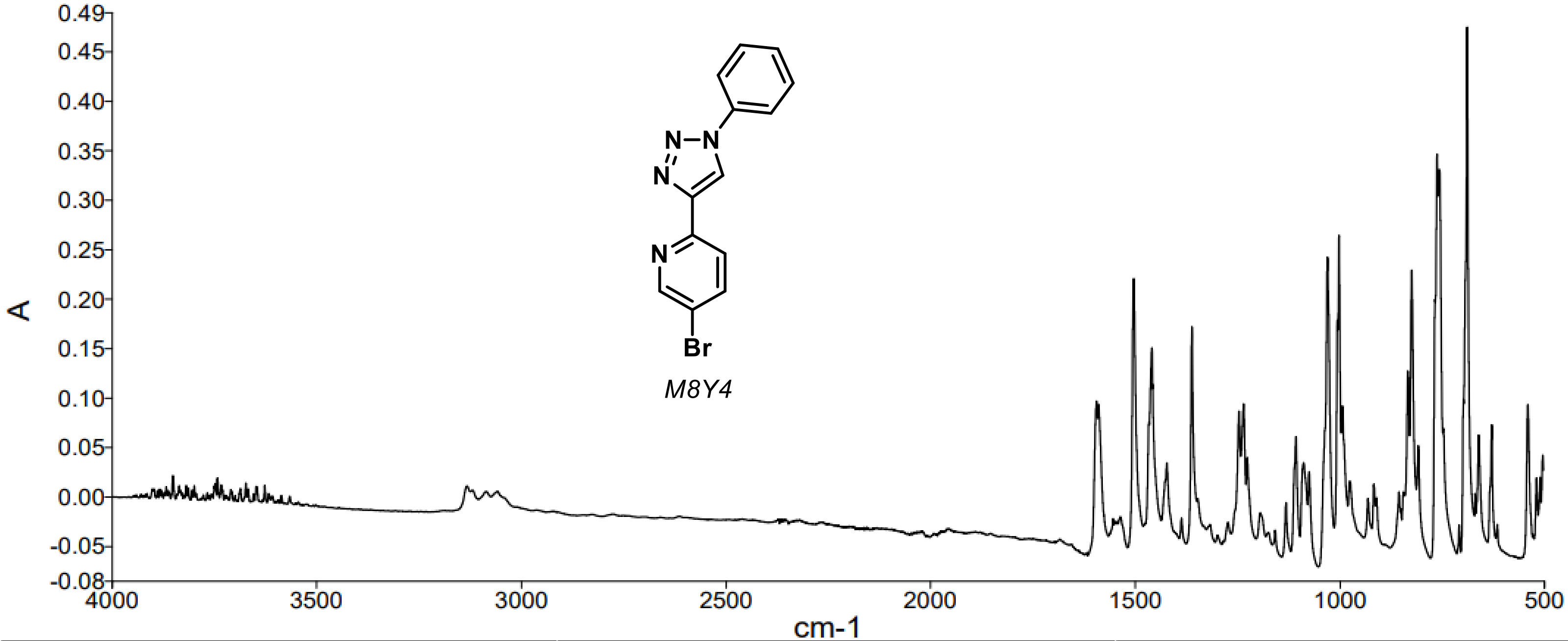

Sample Name	Description	Quality Checks
DRH-051-2_M8Y4	Sample 160 By Administrator Date Thursday, March 06 2025	The Quality Checks give rise to a Baseline High warning for the sample.

Figure S130: FTIR (ATIR) spectrum of M8Y4 (lab book ref. DRH-051-2)

PerkinElmer Spectrum Version 10.5.4
06 March 2025 14:20

Analyst Administrator
Date 06 March 2025 14:20

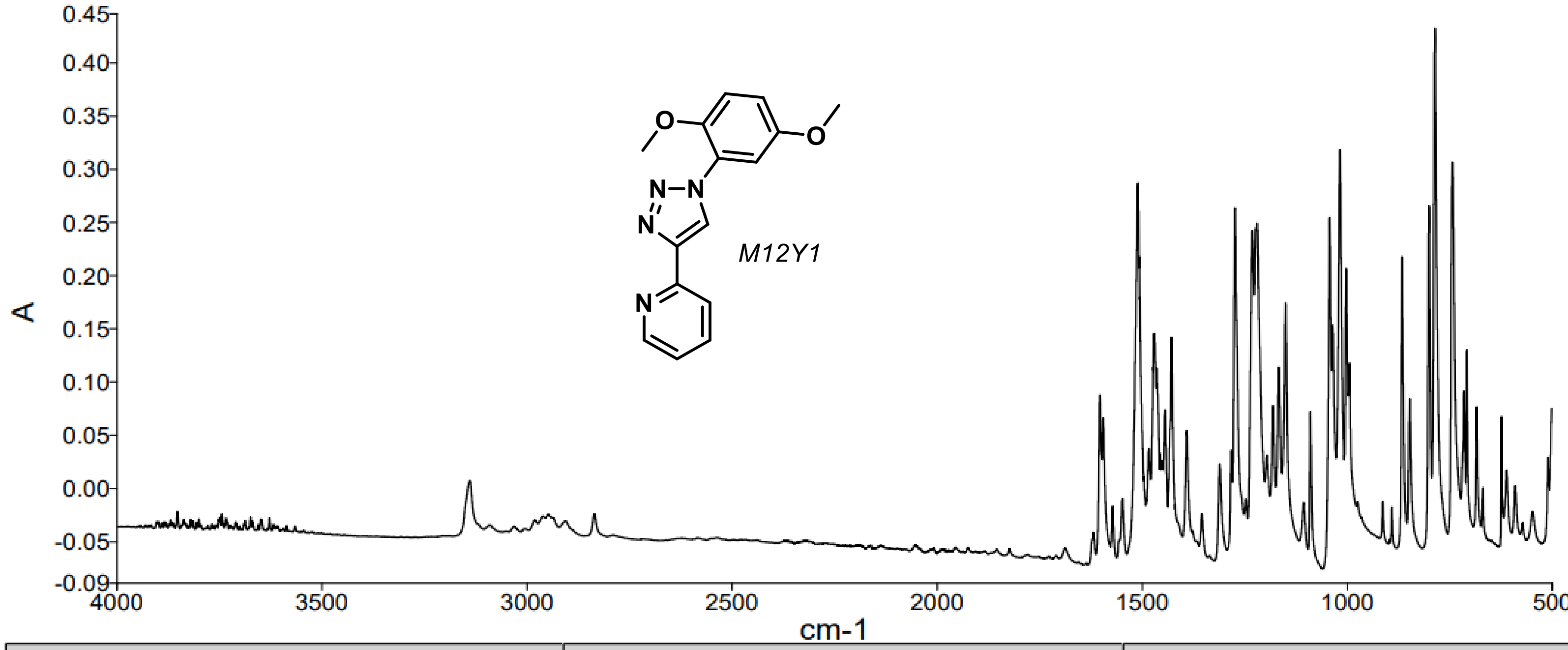

Sample Name	Description	Quality Checks
DRH-051-3_M12Y1	Sample 161 By Administrator Date Thursday, March 06 2025	The Quality Checks give rise to a Baseline High warning for the sample.

Figure S131: FTIR (ATIR) spectrum of M12Y1 (lab book ref. DRH-051-3)

PerkinElmer Spectrum Version 10.5.4
06 March 2025 14:36

Analyst Administrator
Date 06 March 2025 14:36

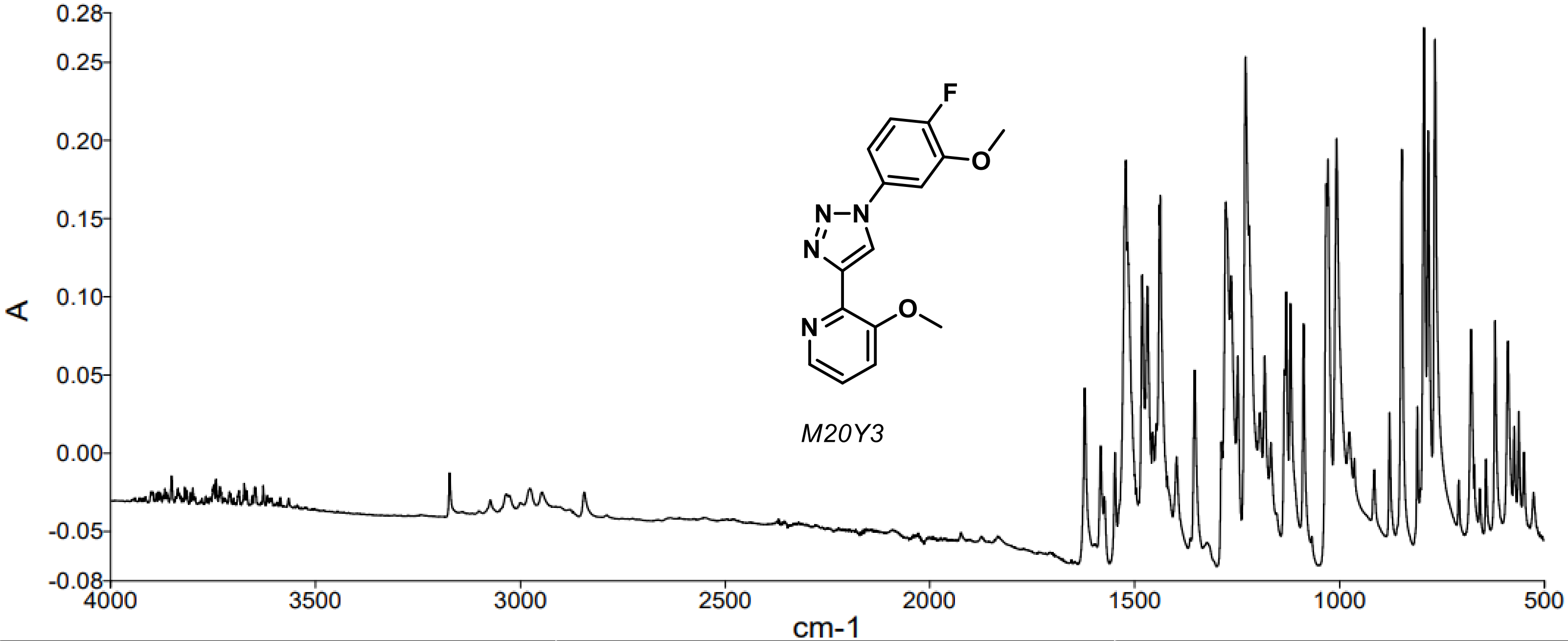

Sample Name	Description	Quality Checks
DRH-051-5_M20Y3	Sample 164 By Administrator Date Thursday, March 06 2025	The Quality Checks give rise to a Baseline High warning for the sample.

Figure S132: FTIR (ATIR) spectrum of M20Y3 (lab book ref. DRH-051-5)

PerkinElmer Spectrum Version 10.5.4
15 May 2025 15:32

Analyst	Administrator
Date	15 May 2025 15:32

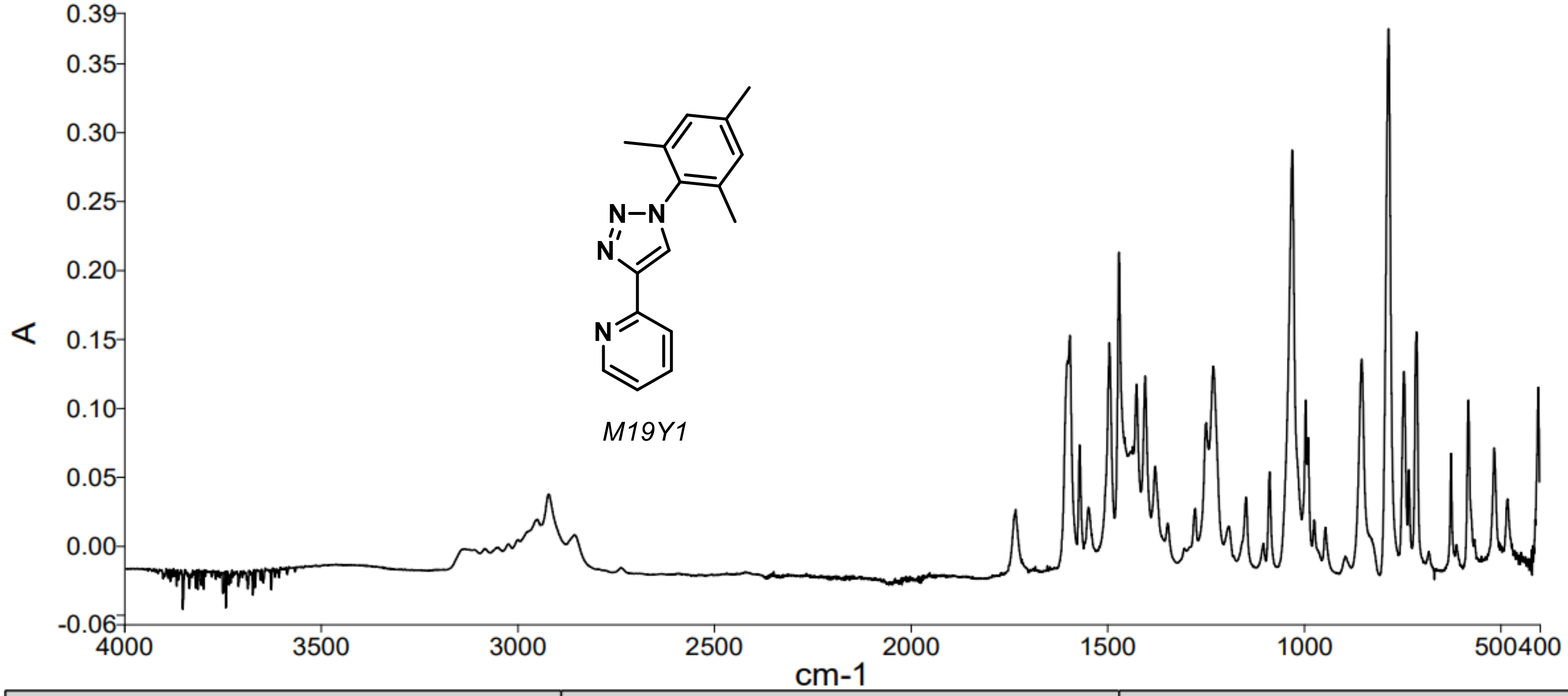

Sample Name	Description	Quality Checks
DRH-066 M19Y1	DRH-066 M19Y1	The Quality Checks give rise to a Baseline High warning for the sample.

Figure S133: FTIR (ATIR) spectrum of M19Y1 (lab book ref. DRH-066-1)

PerkinElmer Spectrum Version 10.5.4
15 May 2025 15:27

Analyst	Administrator
Date	15 May 2025 15:27

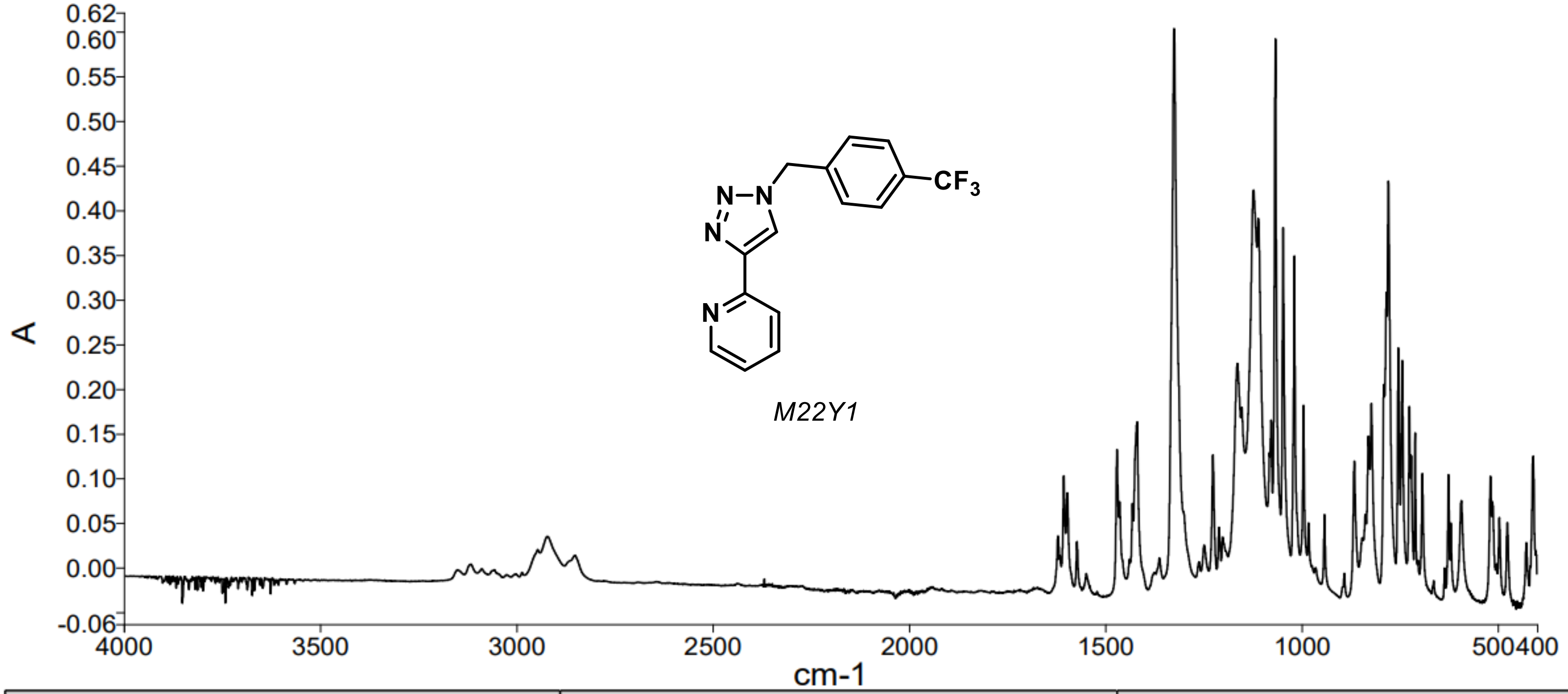

Sample Name	Description	Quality Checks
DRH-066 M22Y1	DRH-066 M22Y1	The Quality Checks give rise to a Baseline High warning for the sample.

Figure S134: FTIR (ATIR) spectrum of M22Y1 (lab book ref. DRH-066-2)

PerkinElmer Spectrum Version 10.5.4
05 September 2025 10:13

Analyst Administrator
Date 05 September 2025 10:13

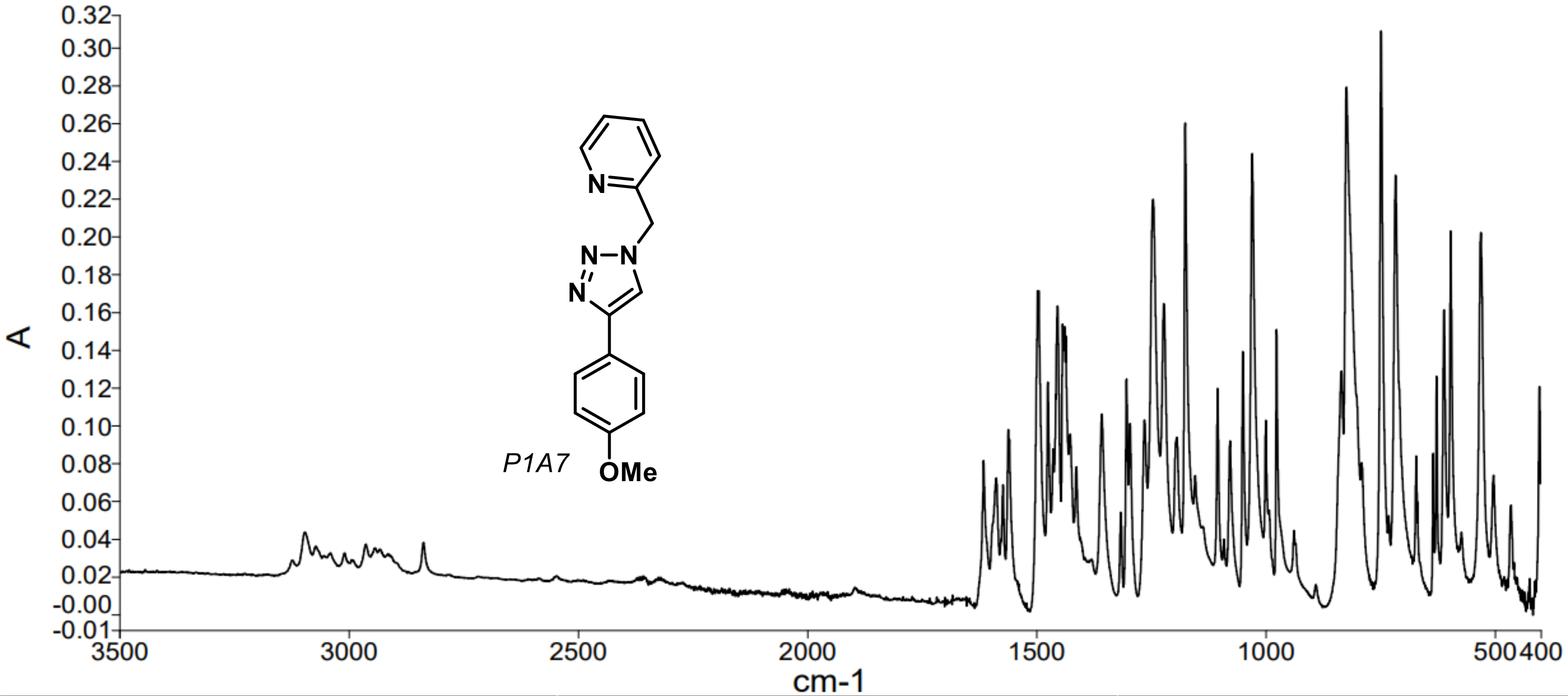

Sample Name	Description	Quality Checks
Administrator 267	DRH-085-2 P1A7	The Quality Checks do not report any warnings for the sample.

Figure S135: FTIR (ATIR) spectrum of P1A7 (lab book ref. DRH-085-2)

PerkinElmer Spectrum Version 10.5.4
08 September 2025 10:18

Analyst	Administrator
Date	08 September 2025 10:18

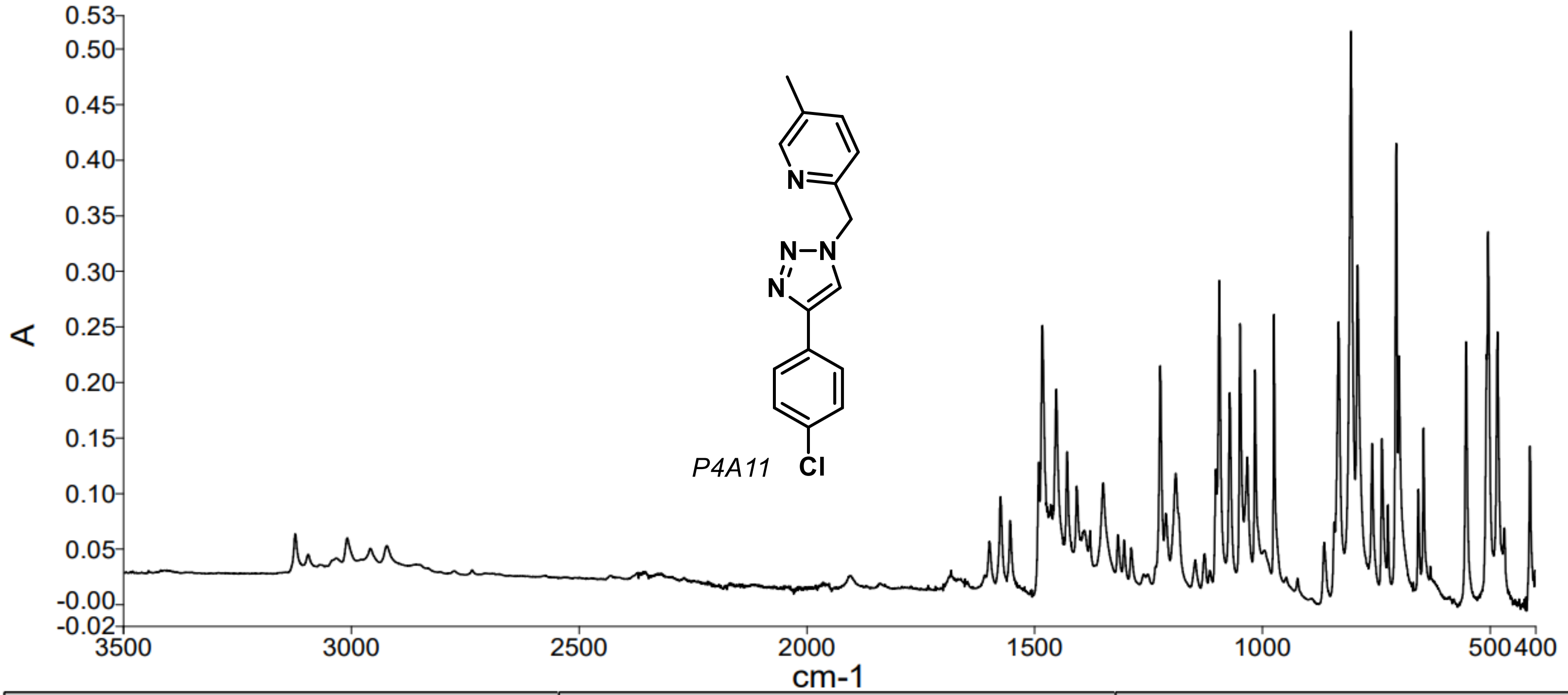

Sample Name	Description	Quality Checks
Administrator 268_1	DRH-085-3 P4A11	The Quality Checks do not report any warnings for the sample.

Figure S136: FTIR (ATIR) spectrum of P4A11 (lab book ref. DRH-085-3)

PerkinElmer Spectrum Version 10.5.4
06 March 2025 14:47

Analyst	Administrator
Date	06 March 2025 14:47

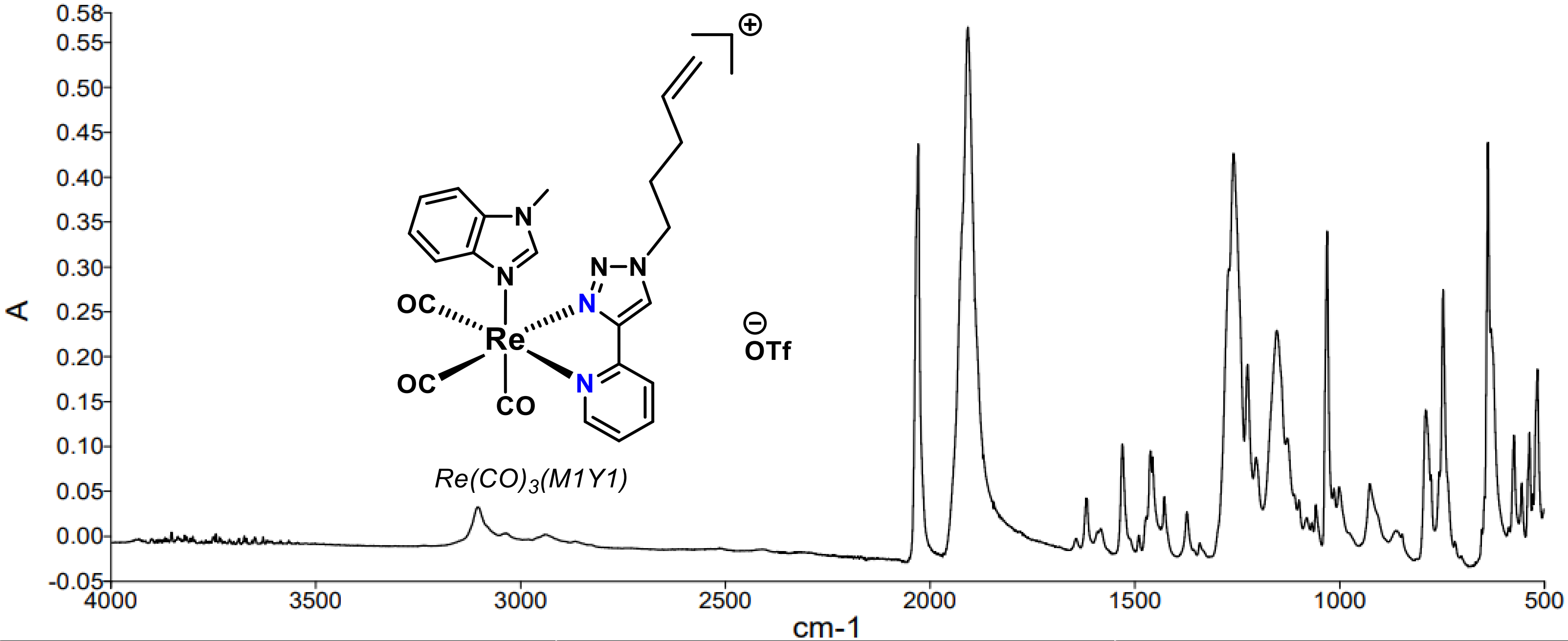

Sample Name	Description	Quality Checks
DRH-052_Re(CO)3M1Y1BeMeIm	Sample 165 By Administrator Date Thursday, March 06 2025	The Quality Checks give rise to a Baseline High warning for the sample.

Figure S137: FTIR (ATIR) spectrum of $Re(CO)_3$(M1Y1) (lab book ref. DRH-052)

PerkinElmer Spectrum Version 10.5.4
06 March 2025 14:54

Analyst	Administrator
Date	06 March 2025 14:54

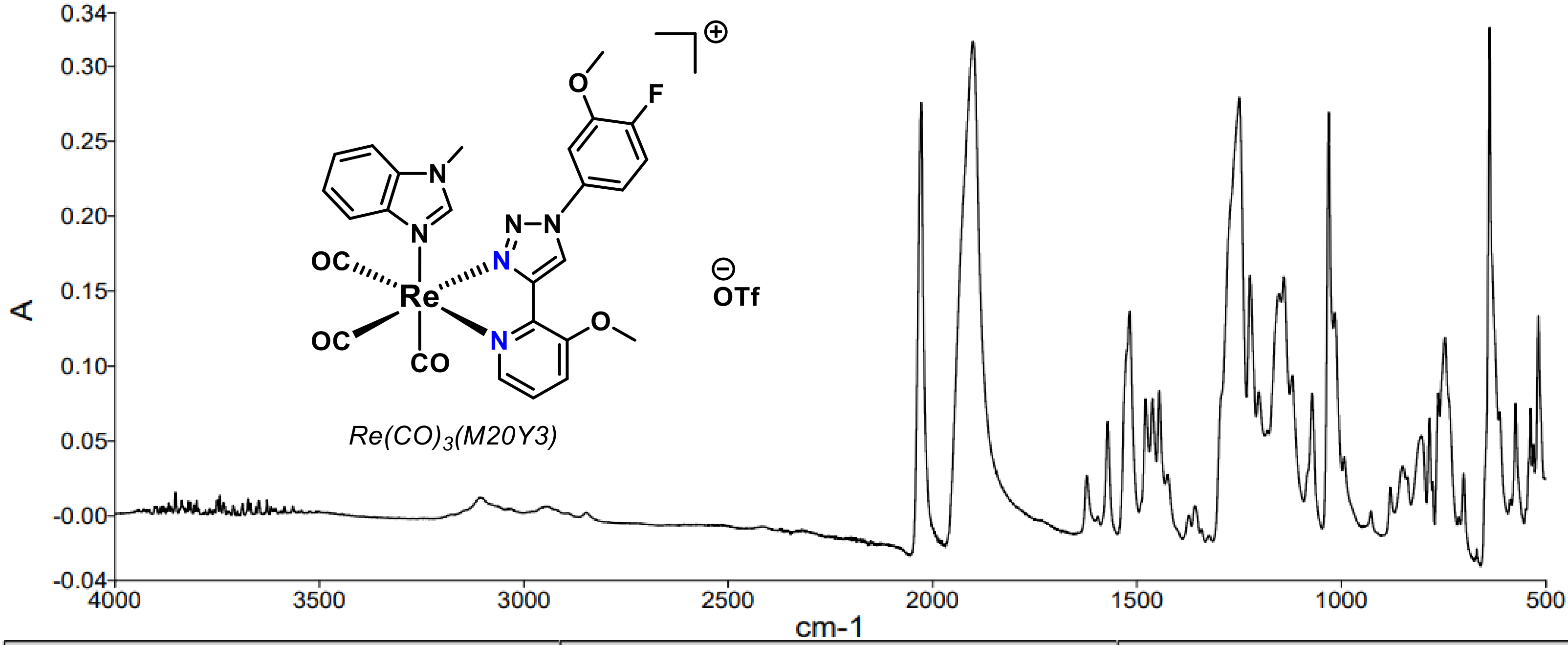

Sample Name	Description	Quality Checks
DRH-053_Re(CO)3M20Y3BeMeIm	Sample 166 By Administrator Date Thursday, March 06 2025	The Quality Checks give rise to a Baseline High warning for the sample.

Figure S138: FTIR (ATIR) spectrum of $Re(CO)_3$(M20Y3) (lab book ref. DRH-053)

PerkinElmer Spectrum Version 10.5.4
06 March 2025 15:05

Analyst Administrator
Date 06 March 2025 15:05

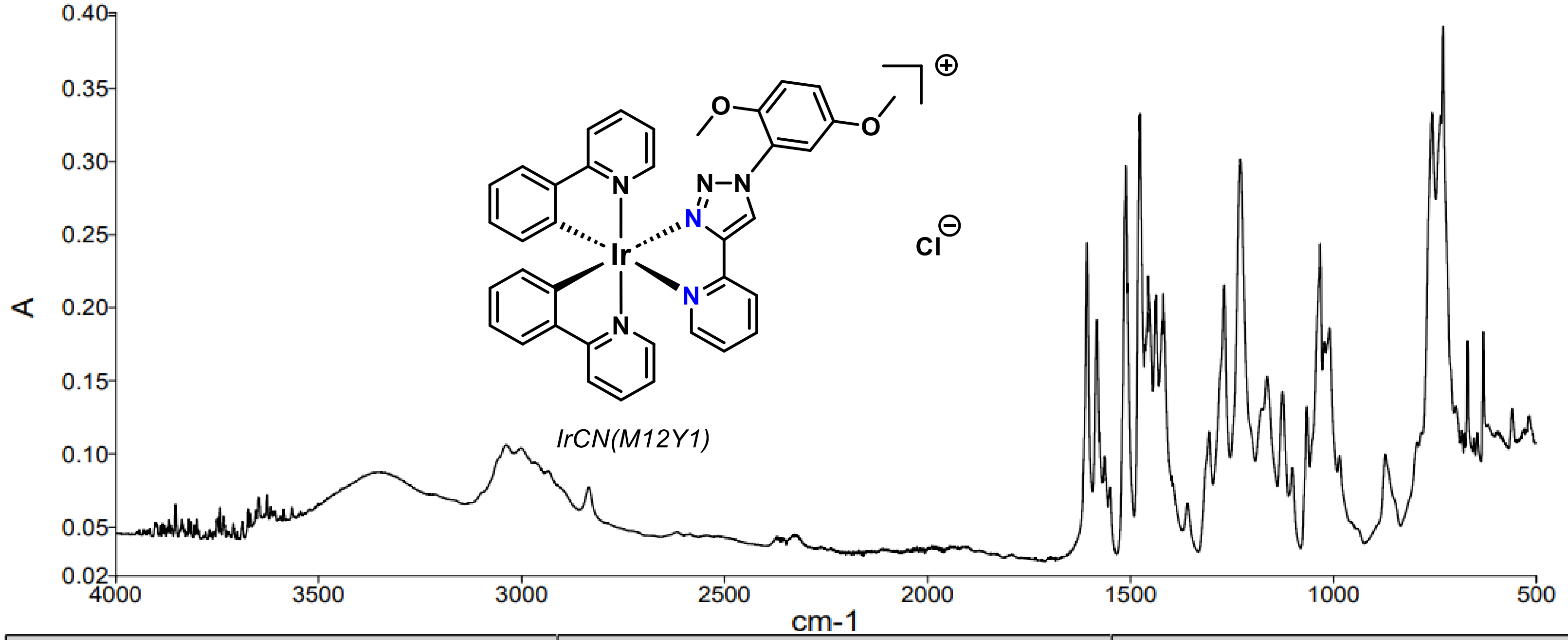

Sample Name	Description	Quality Checks
DRH-056_IrCNM12Y1	Sample 168 By Administrator Date Thursday, March 06 2025	The Quality Checks do not report any warnings for the sample.

Figure S139: FTIR (ATIR) spectrum of IrCN(M12Y1) (lab book ref. DRH-056)

PerkinElmer Spectrum Version 10.5.4
06 March 2025 15:12

Analyst Administrator
Date 06 March 2025 15:12

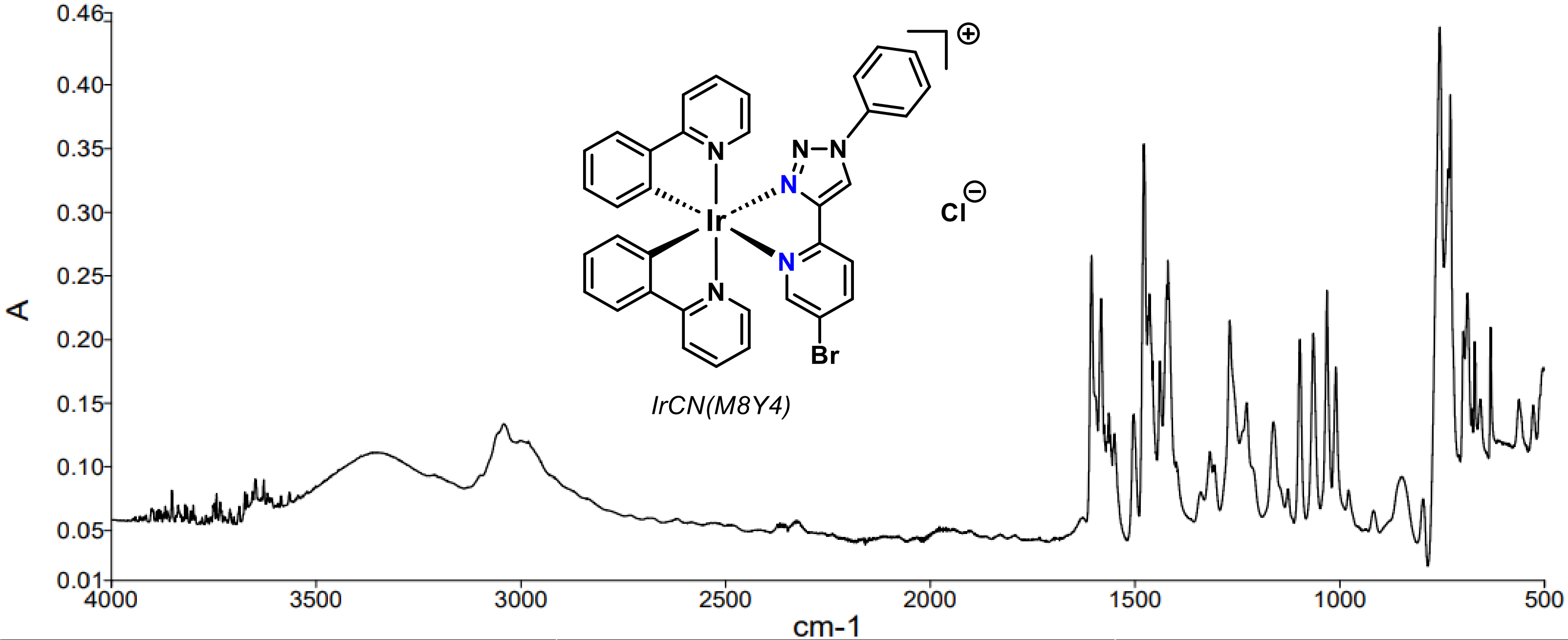

Sample Name	Description	Quality Checks
DRH-057_IrCNM8Y4	Sample 169 By Administrator Date Thursday, March 06 2025	The Quality Checks do not report any warnings for the sample.

Figure S140: FTIR (ATIR) spectrum of IrCN(M8Y4) (lab book ref. DRH-057)

PerkinElmer Spectrum Version 10.5.4
15 May 2025 15:20

Analyst Administrator
Date 15 May 2025 15:20

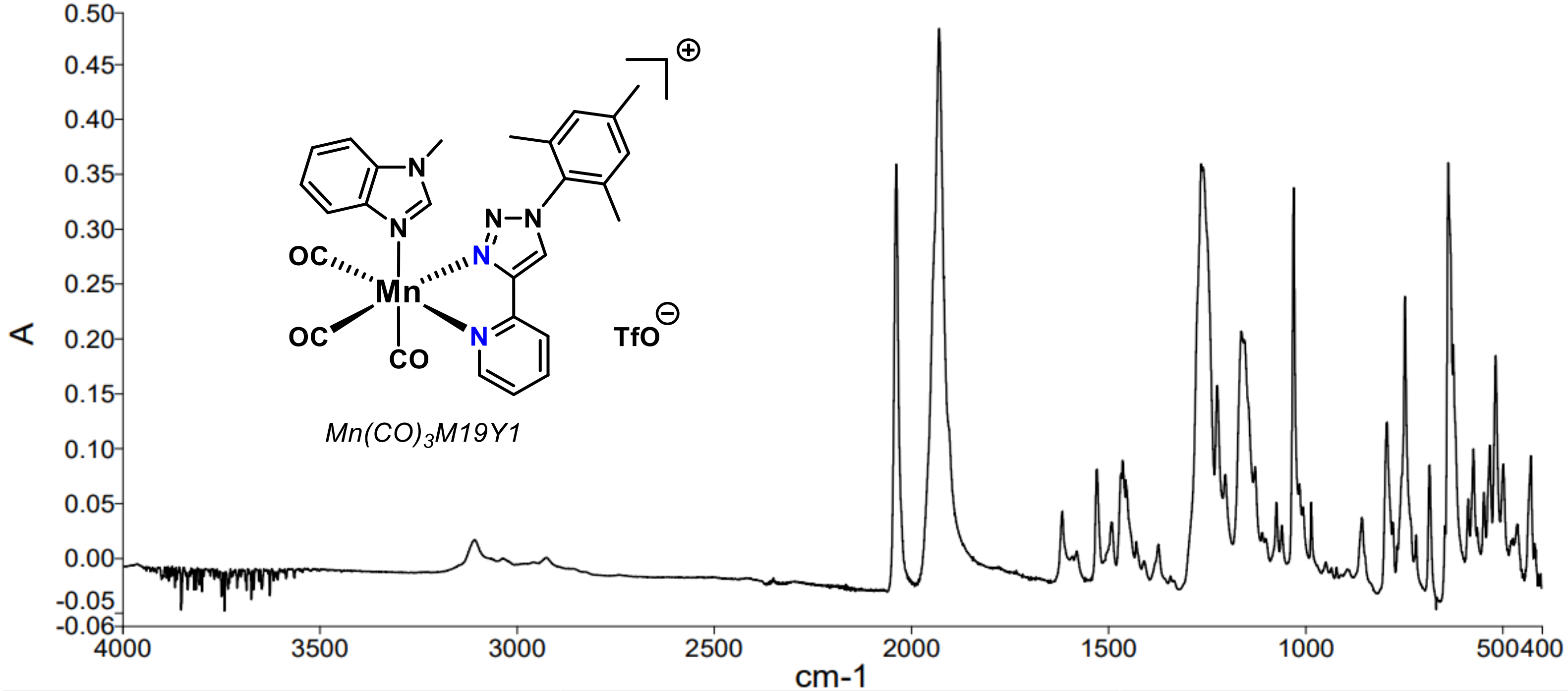

Sample Name	Description	Quality Checks
DRH-068 MnM19Y1	DRH-068 Mn(CO)3M19Y1	The Quality Checks give rise to a Baseline High warning for the sample.

Figure S141: FTIR (ATIR) spectrum of Mn(CO)$_3$(M19Y1) (lab book ref. DRH-068-1)

PerkinElmer Spectrum Version 10.5.4
15 May 2025 15:06

Analyst Administrator
Date 15 May 2025 15:06

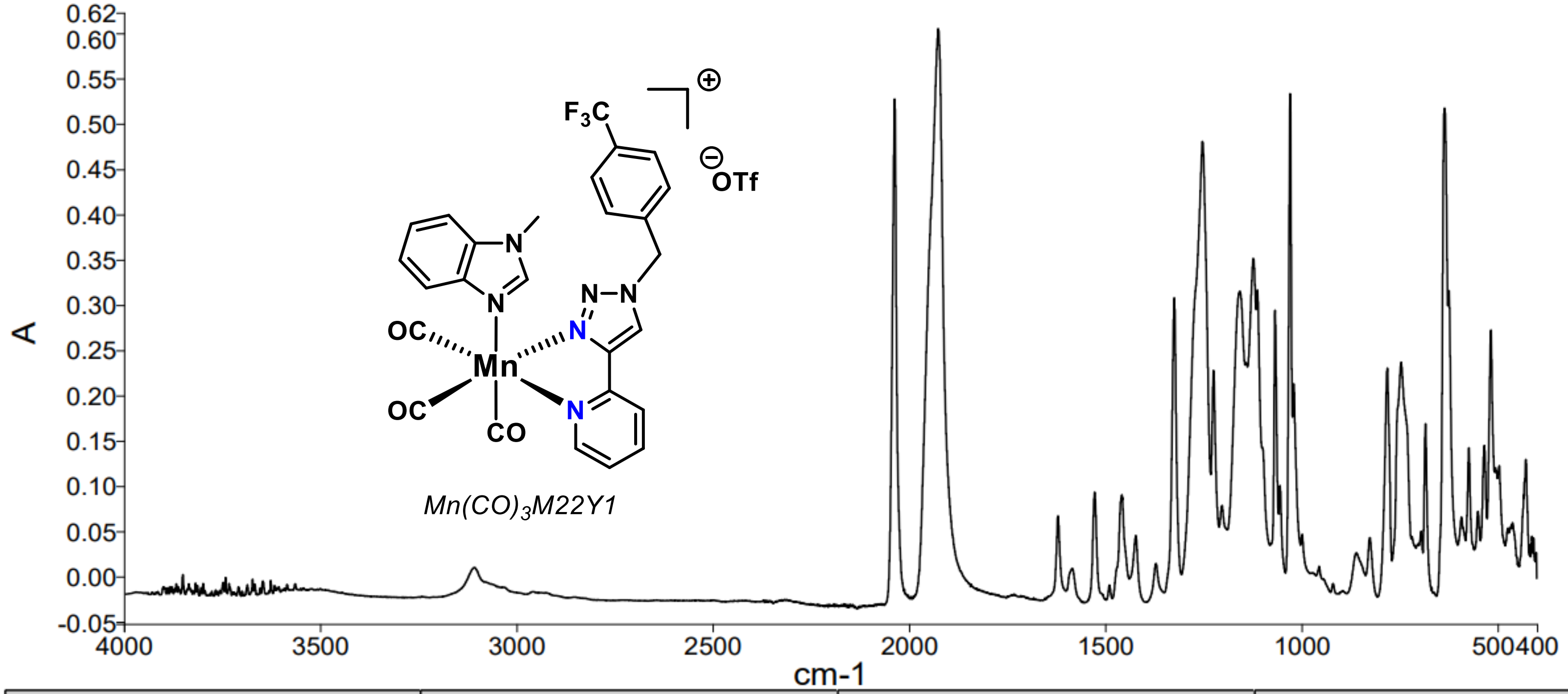

Sample Name	Description	Quality Checks	Number of peaks
DRH-068 MnM22Y1	DRH-068 Mn(CO)3M22Y1	The Quality Checks give rise to a Baseline High warning for the sample.	57

Figure S142: FTIR (ATIR) spectrum of $Mn(CO)_3$(M22Y1) (lab book ref. DRH-068-1)

PerkinElmer Spectrum Version 10.5.4
08 September 2025 10:19

Analyst	Administrator
Date	08 September 2025 10:19

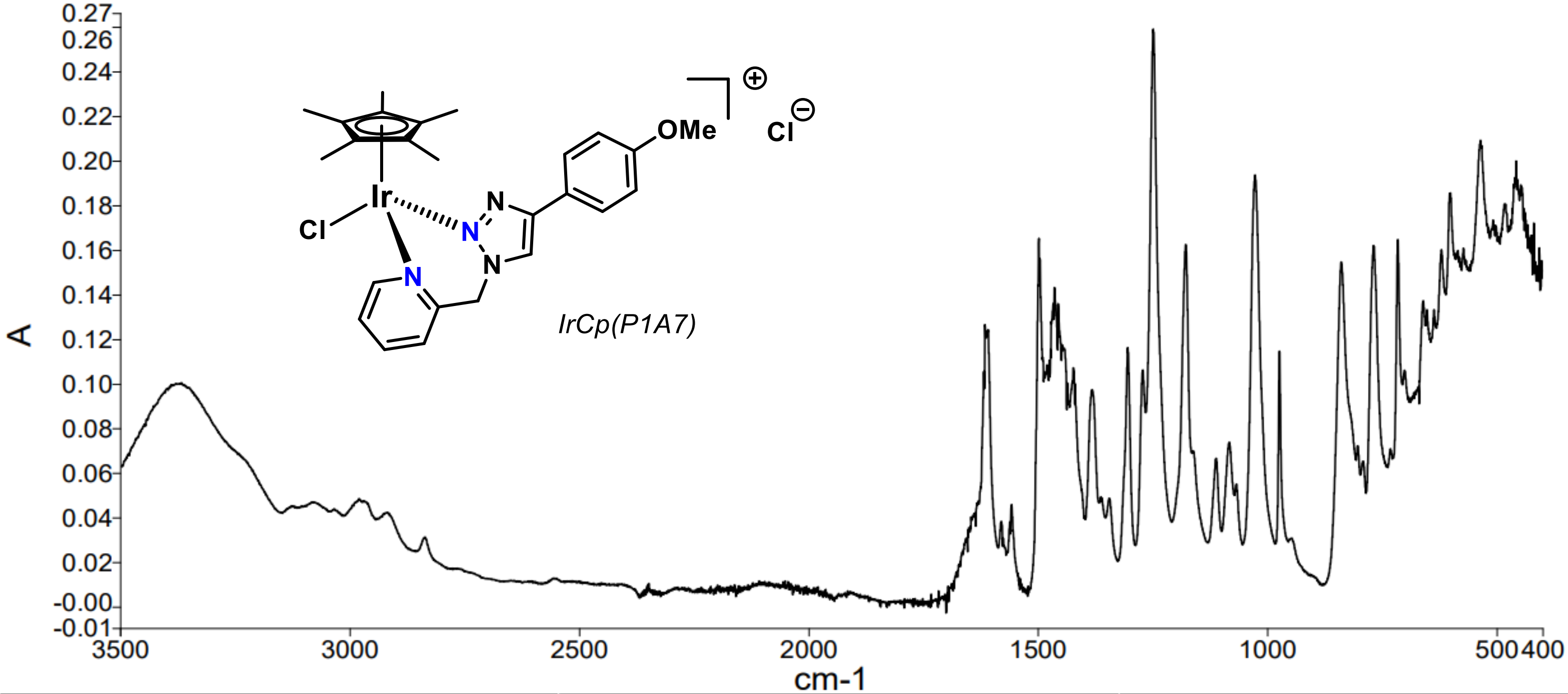

Sample Name	Description	Quality Checks
Administrator 269	DRH-088-2 IrCp1P1A7	The Quality Checks give rise to a Baseline Slope warning for the sample.

Figure S143: FTIR (ATIR) spectrum of IrCp*(P1A7) (lab book ref. DRH-088-2)

PerkinElmer Spectrum Version 10.5.4
05 September 2025 10:30

Analyst	Administrator
Date	05 September 2025 10:30

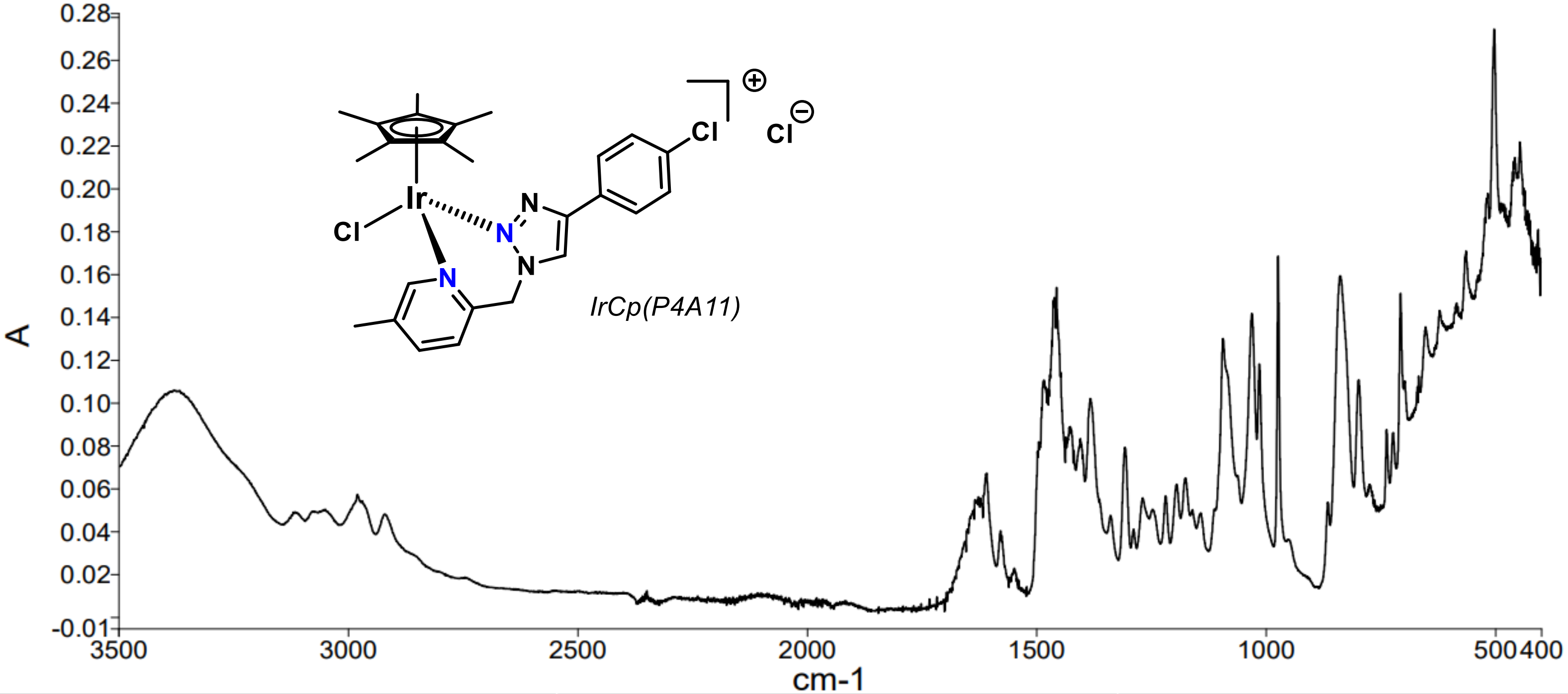

Sample Name	Description	Quality Checks
Administrator 270	DRH-088-3 IrCp1P4A11	The Quality Checks give rise to multiple warnings for the sample.

Figure S144: FTIR (ATIR) spectrum of IrCp*(P4A11) (lab book ref. DRH-088-3)

PerkinElmer Spectrum Version 10.5.4
16 March 2025 11:49

Analyst	Administrator
Date	16 March 2025 11:49

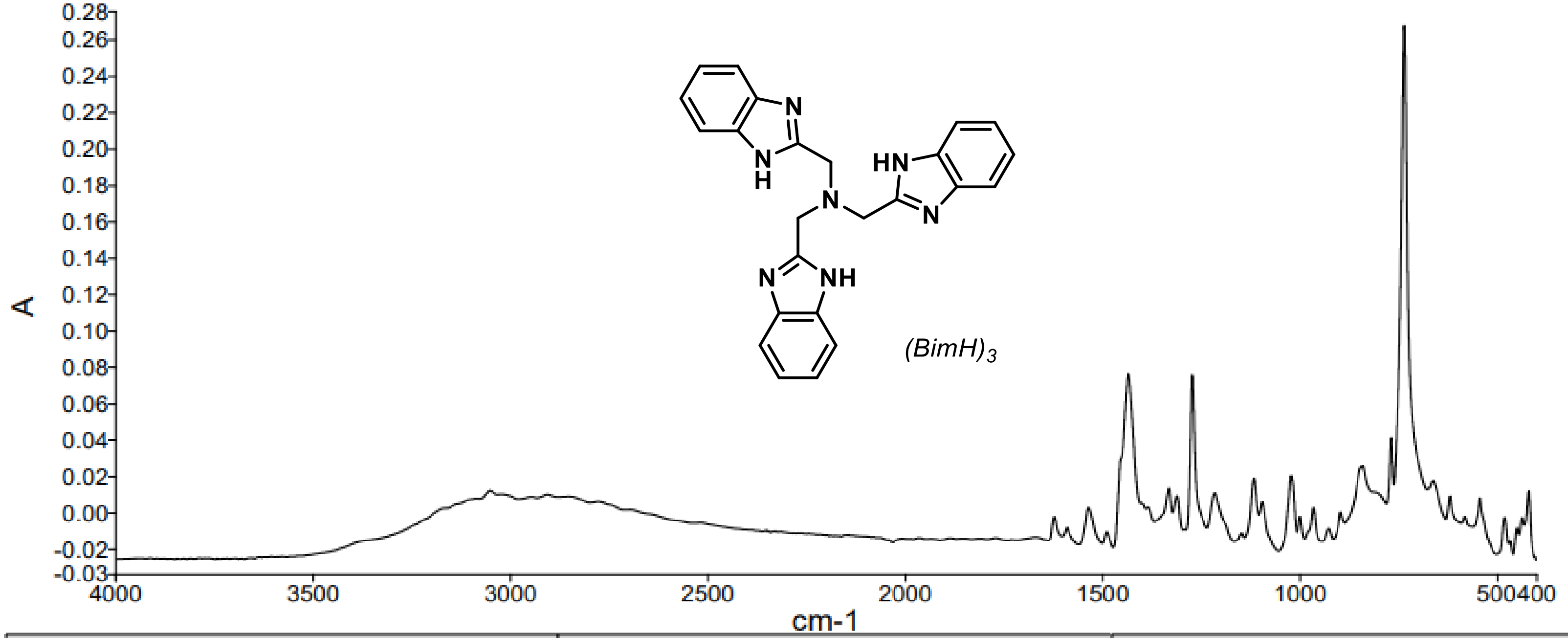

Sample Name	Description	Quality Checks
DRH-008 (BimH)3 characterisation_1_abs	Sample 116 By Administrator Date Friday, June 14 2024	The Quality Checks do not report any warnings for the sample.

Figure S145: FTIR (ATIR) spectrum of $(BimH)_3$ (lab book ref. DRH-008)

PerkinElmer Spectrum Version 10.5.4
16 March 2025 11:43

Analyst Administrator
Date 16 March 2025 11:43

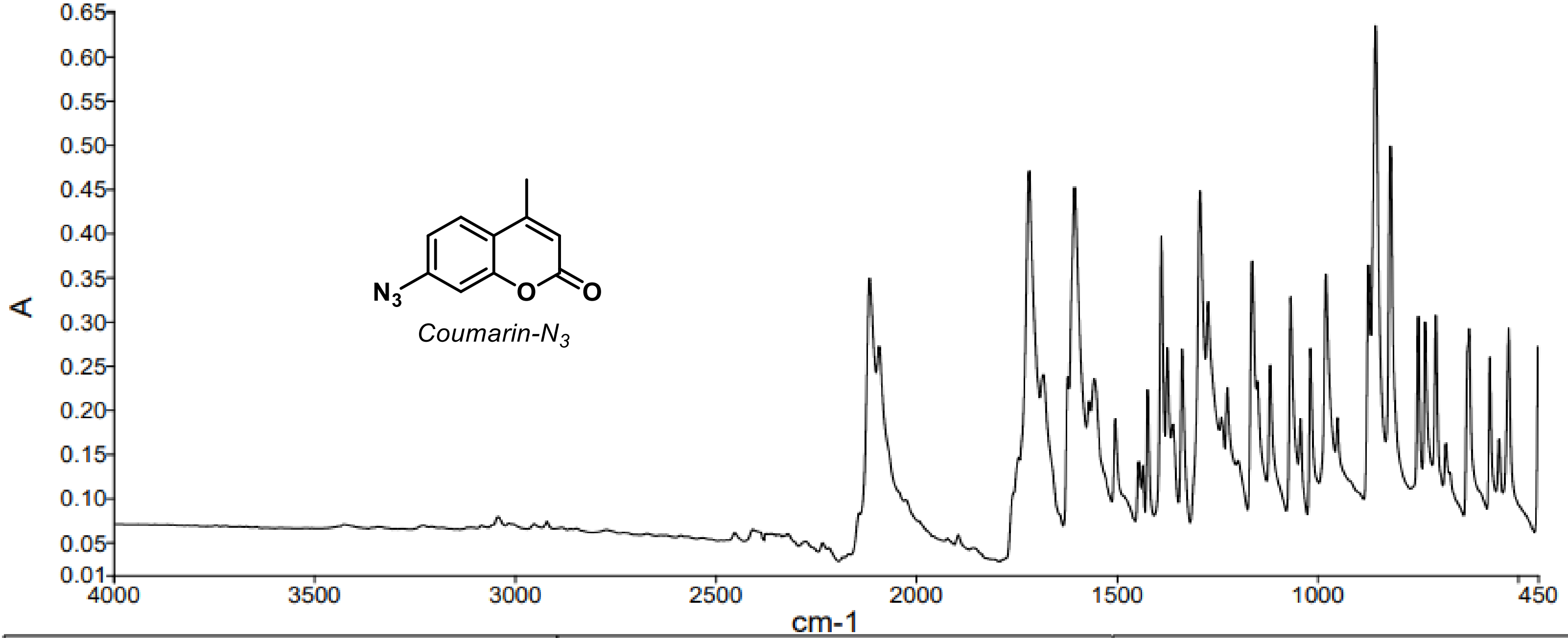

Sample Name	Description	Quality Checks
Administrator 91	DRH-030 coumarin-N3	The Quality Checks do not report any warnings for the sample.

Figure S146: FTIR (ATIR) spectrum of Coumarin-N_3 (lab book ref. DRH-030)

10. References

1. Meng, G. *et al.* Modular click chemistry libraries for functional screens using a diazotizing reagent. *Nature* **574**, 86–89 (2019).
2. Weng, C., Shen, L. & Ang, W. H. Harnessing Endogenous Formate for Antibacterial Prodrug Activation by in cellulo Ruthenium-Mediated Transfer Hydrogenation Reaction. *Angew. Chem. Int. Ed.* **59**, 9314–9318 (2020).
3. Kench, T. *et al.* A Semi-Automated, High-Throughput Approach for the Synthesis and Identification of Highly Photo-Cytotoxic Iridium Complexes. *Angew. Chem. Int. Ed.* **63**, e202401808 (2024).
4. Miller, A. H. *et al.* Catch-and-Release: The Assembly, Immobilization, and Recycling of Redox-Reversible Artificial Metalloenzymes. *ACS Catal.* **14**, 3218–3227 (2024).
5. Entradas, T., Waldron, S. & Volk, M. The detection sensitivity of commonly used singlet oxygen probes in aqueous environments. *J. Photochem. Photobiol. B* **204**, 111787 (2020).
6. Frei, A. *et al.* Synthesis, Characterization, and Biological Evaluation of New Ru(II) Polypyridyl Photosensitizers for Photodynamic Therapy. *J. Med. Chem.* **57**, 7280–7292 (2014).
7. Hulstaert, N. *et al.* ThermoRawFileParser: Modular, Scalable, and Cross-Platform RAW File Conversion. *J. Proteome. Res.* **19**, 537–542 (2020).
8. Mosmann, T. Rapid colorimetric assay for cellular growth and survival: Application to proliferation and cytotoxicity assays. *J. Immunol. Methods* **65**, 55–63 (1983).
9. Stach, M. *et al.* Membrane disrupting antimicrobial peptide dendrimers with multiple amino termini. *Med. Chem. Commun.* **3**, 86–89 (2012).
10. Orsi, M. & Frei, A. ELECTRUM: an electron configuration-based universal metal fingerprint for transition metal compounds. *Digit. Discov.* Advance Article (2025). doi:10.1039/D5DD00145E
11. CrysAlisPro. CrysAlisPro, Oxford Diffraction Ltd. Version 1.171.34.41. Oxford Diffraction Ltd. Version 1.171.34.41.
12. Empirical absorption correction using spherical harmonics implemented in SCALE3 ABSPACK scaling algorithm within CrysAlisPro software. Scale3. Oxford Diffraction Ltd. Version 1.171.34.40.

13. Dolomanov, O. V., Bourhis, L. J., Gildea, R. J., Howard, J. A. K. & Puschmann, H. OLEX2: A complete structure solution, refinement and analysis program. *J. Appl. Crystallogr.* **42**, 339–341 (2009).

14. Sheldrick, G. M. SHELXT - Integrated space-group and crystal-structure determination. *Acta Cryst. A* **71**, 3–8 (2015).
