# Supplementary material for: High-throughput triazole-based combinatorial click chemistry for the synthesis and identification of functional metal complexes
Source: Nat Commun. 2025 Dec 23;16:11195. doi: 10.1038/s41467-025-67341-z (PMC12728216; doi:10.1038/s41467-025-67341-z)
Supplement: Supplementary file 2 — Description of Additional Supplementary Files [file 41467_2025_67341_MOESM2_ESM.pdf]

Supplementary Data 1

Collated LC-MS (Liquid Chromotography – Mass Spectrometry) spectra of the libraries described in the associated publication. The lower trace in each spectrum is the UV absorbance at 254 nm is shown, and the upper trace is the extracted ion chromatogram (ESI, positive mode) for the target mass of the complex. The retention time and peak area percentage of the target peak is displayed. Spectra for the following libraries are shown; Tz-4-P (mass + H), IrCN, IrCp(Tz-4-P), IrCp(Tz-4-P) (2+ adduct), ReCO3, ReCO3 solvent, MnCO3, MnCO3 solvent, RuCy(Tz-4-P), Tz-1-MP (mass + H), IrCp(Tz-1-MP), IrCp(Tz-1-MP) (2+ adduct), RuCy(Tz-1-MP). The IrCp 2+ adducts have a mass corresponding to $[M\text{- Cl, + MeCN}]^{2+}$.
