# Supplementary material for: High-throughput triazole-based combinatorial click chemistry for the synthesis and identification of functional metal complexes
Source: Nat Commun. 2025 Dec 23;16:11195. doi: 10.1038/s41467-025-67341-z (PMC12728216; doi:10.1038/s41467-025-67341-z)
Supplement: Supplementary file 4 — Reporting Summary [file 41467_2025_67341_MOESM4_ESM.pdf]

nature portfolio

Corresponding author(s): Angelo Frei

Last updated by author(s): Nov 14, 2025

Reporting Summary

Nature Portfolio wishes to improve the reproducibility of the work that we publish. This form provides structure for consistency and transparency in reporting. For further information on Nature Portfolio policies, see our Editorial Policies and the Editorial Policy Checklist.

Statistics

For all statistical analyses, confirm that the following items are present in the figure legend, table legend, main text, or Methods section.

n/a	Confirmed	
☐	☒	The exact sample size (*n*) for each experimental group/condition, given as a discrete number and unit of measurement
☐	☒	A statement on whether measurements were taken from distinct samples or whether the same sample was measured repeatedly
☒	☐	The statistical test(s) used AND whether they are one- or two-sided<br>*Only common tests should be described solely by name; describe more complex techniques in the Methods section.*
☒	☐	A description of all covariates tested
☒	☐	A description of any assumptions or corrections, such as tests of normality and adjustment for multiple comparisons
☐	☒	A full description of the statistical parameters including central tendency (e.g. means) or other basic estimates (e.g. regression coefficient) AND variation (e.g. standard deviation) or associated estimates of uncertainty (e.g. confidence intervals)
☒	☐	For null hypothesis testing, the test statistic (e.g. *F*, *t*, *r*) with confidence intervals, effect sizes, degrees of freedom and *P* value noted<br>*Give P values as exact values whenever suitable.*
☒	☐	For Bayesian analysis, information on the choice of priors and Markov chain Monte Carlo settings
☒	☐	For hierarchical and complex designs, identification of the appropriate level for tests and full reporting of outcomes
☒	☐	Estimates of effect sizes (e.g. Cohen's *d*, Pearson's *r*), indicating how they were calculated

Our web collection on statistics for biologists contains articles on many of the points above.

Software and code

Policy information about availability of computer code

Data collection	NMR data was collected using Bruker Topspin 3.5pl6 (600 MHz), TopSpin 4.1pl3 (700 MHz). LCMS data was collected using Thermo Scientific Xcalibur 4.4. High-resolution mass spectrometry data was collected using Bruker Compass HyStar 4.1. FT-IR data collected using PerkinElmer Spectrum v10.5.4. UV-Vis data, fluorescence and biological data was collected using Techan i-control 2.0. XRD data was collected using Rigaku CrysAlisPro v44.128a.
Data analysis	Data analysis was performed for High-resolution mass spectrometry using Bruker Compass DataAnalysis. NMR data was processed using MestReNova 15.1.0-37919. XRD data was processed in Olex2-1.5-ac6-020. Biological data (CC50, Haemolysis) was analysed and processed using GraphPad Prism v8.0.2 (GraphPad Software, California, USA). Code writing was done on Visual Studio Code 1.105.1, using Python 3.11.9. LC-MS Spectra were processed using R-and Python scripts as well as the ThermoFisherParser code from: https://github.com/compomics/ThermoRawFileParser/releases . All our code has been uploaded to our GitHub: https://github.com/TheFreiLab/FreiLab-LCMSProcessing and to https://github.com/TheFreiLab/ClickCombChem-ML

For manuscripts utilizing custom algorithms or software that are central to the research but not yet described in published literature, software must be made available to editors and reviewers. We strongly encourage code deposition in a community repository (e.g. GitHub). See the Nature Portfolio guidelines for submitting code & software for further information.

Data

Policy information about availability of data

All manuscripts must include a data availability statement. This statement should provide the following information, where applicable:

- Accession codes, unique identifiers, or web links for publicly available datasets
- A description of any restrictions on data availability
- For clinical datasets or third party data, please ensure that the statement adheres to our policy

The authors declare that all data supporting the current findings of this study are available in the main manuscript or in the associated Supporting Information. All data are available from the corresponding author upon request. Source data are provided with this paper. This constitutes all numerical LC-MS (retention times and conversion%) and biological (Minimum inhibitory concentrations, HEK293T Growth inhibition)) data. The collated LC-MS spectra of all combinatorial libraries are supplied as Supplementary Data 1. All LC-MS, NMR, XRD and kinetic raw data can be found at https://doi.org/10.15124/728916c9-e516-4790-b75d-41cd0d91a362 and the X-ray crystallographic coordinates for the structures Re(CO)3(M1Y1), Re(CO)3(M20Y3) and Mn(CO)3(M19Y1) reported in this study has been deposited at the Cambridge Crystallographic Data Centre (CCDC), under deposition numbers CDCC 2455425, 2504788, 2504789. These data can be obtained free of charge from The Cambridge Crystallographic Data Centre via www.ccdc.cam.ac.uk/data_request/cif.

Research involving human participants, their data, or biological material

Policy information about studies with human participants or human data. See also policy information about sex, gender (identity/presentation), and sexual orientation and race, ethnicity and racism.

Reporting on sex and gender	not applicable, information not gathered from blood donors
Reporting on race, ethnicity, or other socially relevant groupings	not applicable, information not gathered from blood donors
Population characteristics	not applicable, information not gathered from blood donors
Recruitment	Volunteers were recruited for blood donation. Informed written consent will be obtained by all participants as stated in the blood donation protocol. Volunteers will be recruited through advertising at various locations on the University of York, at York Hospital NHS; and through the local media where deemed necessary. Contact details for the project team will be listed on all material that is used to publicise the research project.
Ethics oversight	Biology Ethics Committee University of York

Note that full information on the approval of the study protocol must also be provided in the manuscript.

Field-specific reporting

Please select the one below that is the best fit for your research. If you are not sure, read the appropriate sections before making your selection.

☒ Life sciences ☐ Behavioural & social sciences ☐ Ecological, evolutionary & environmental sciences

For a reference copy of the document with all sections, see nature.com/documents/nr-reporting-summary-flat.pdf

Life sciences study design

All studies must disclose on these points even when the disclosure is negative.

Sample size	All biological experiments were performed with 2-3 biological and 2-3 technical replicates each as is custom for these assays.
Data exclusions	No data has been excluded from this study
Replication	All biological experiments (microbiological, cell cytotox, haemolysis) were repeated with 2-3 biological and 3 technical replicates each. Results were reproducible
Randomization	Randomization not applicable to the type of testing conducted
Blinding	No direct blinding was done. Generally compounds were tested in biological essays by other collaborators then the one who synthesized them removing potential biases in the interpretation of assays.

Reporting for specific materials, systems and methods

We require information from authors about some types of materials, experimental systems and methods used in many studies. Here, indicate whether each material, system or method listed is relevant to your study. If you are not sure if a list item applies to your research, read the appropriate section before selecting a response.

Materials & experimental systems

n/a	Involved in the study
x	Antibodies
	x Eukaryotic cell lines
x	Palaeontology and archaeology
x	Animals and other organisms
x	Clinical data
x	Dual use research of concern
	Plants

Methods

n/a	Involved in the study
x	ChIP-seq
x	Flow cytometry
x	MRI-based neuroimaging

Eukaryotic cell lines

Policy information about cell lines and Sex and Gender in Research

Cell line source(s)	The human embryonic kidney (HEK293T) cells used were donated by the Willems Research Group at the University of York. They had purchased the cells from ATCC (CRL-3216).
Authentication	None
Mycoplasma contamination	Cells tested negative (by PCR combined with gel electrophoresis)
Commonly misidentified lines (See ICLAC register)	None

Plants

Seed stocks	n/a
Novel plant genotypes	n/a
Authentication	n/a
