# Supplementary material for: High-throughput triazole-based combinatorial click chemistry for the synthesis and identification of functional metal complexes
Source: Nat Commun. 2025 Dec 23;16:11195. doi: 10.1038/s41467-025-67341-z (PMC12728216; doi:10.1038/s41467-025-67341-z)

Tz-4-P library

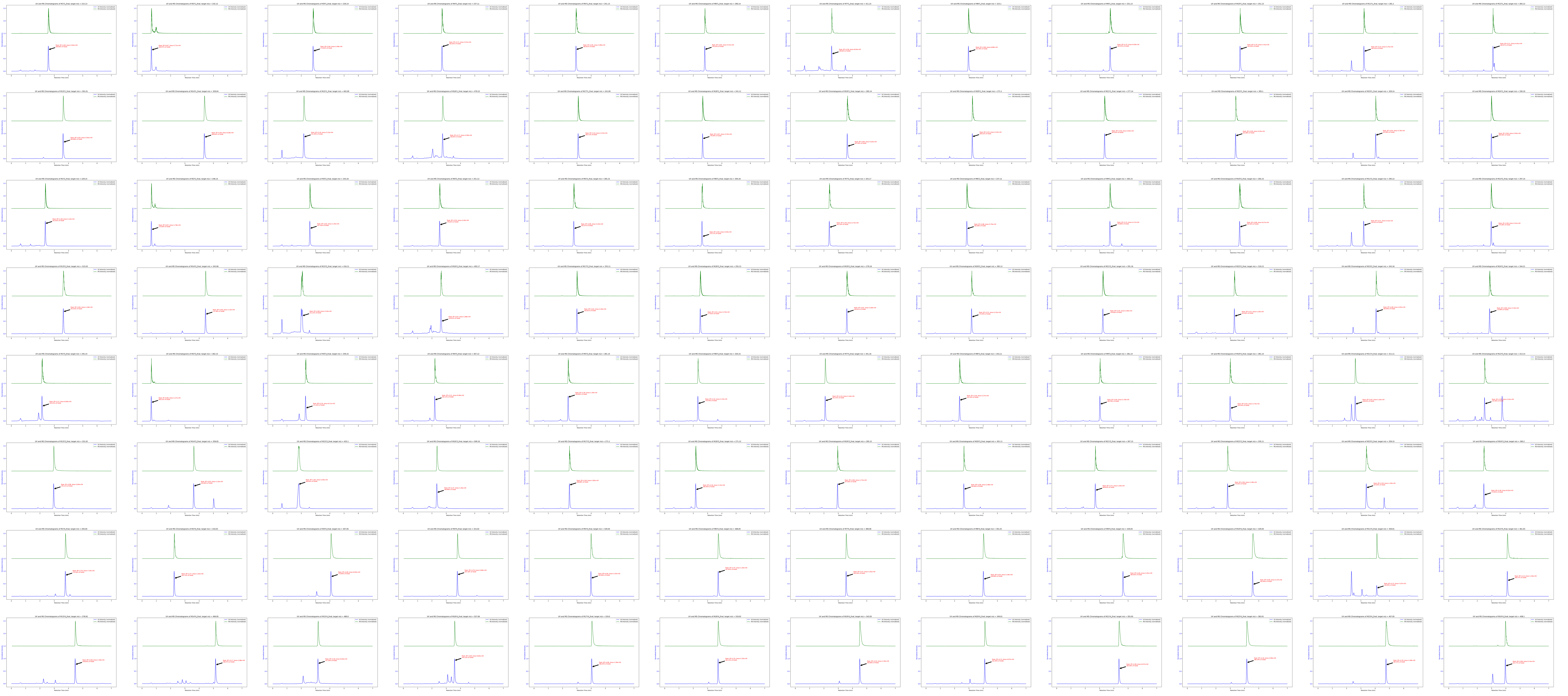

IrCN library

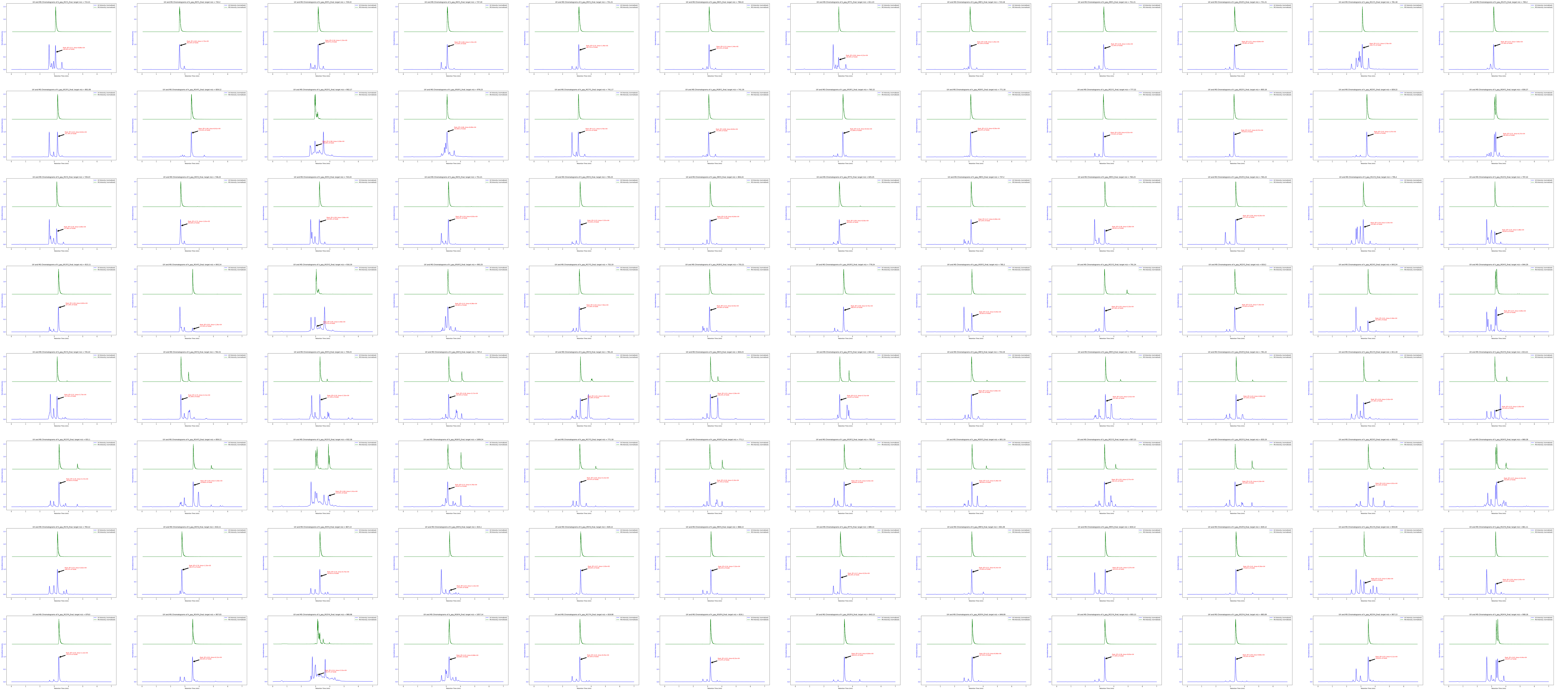

IrCp(Tz-4-P) library

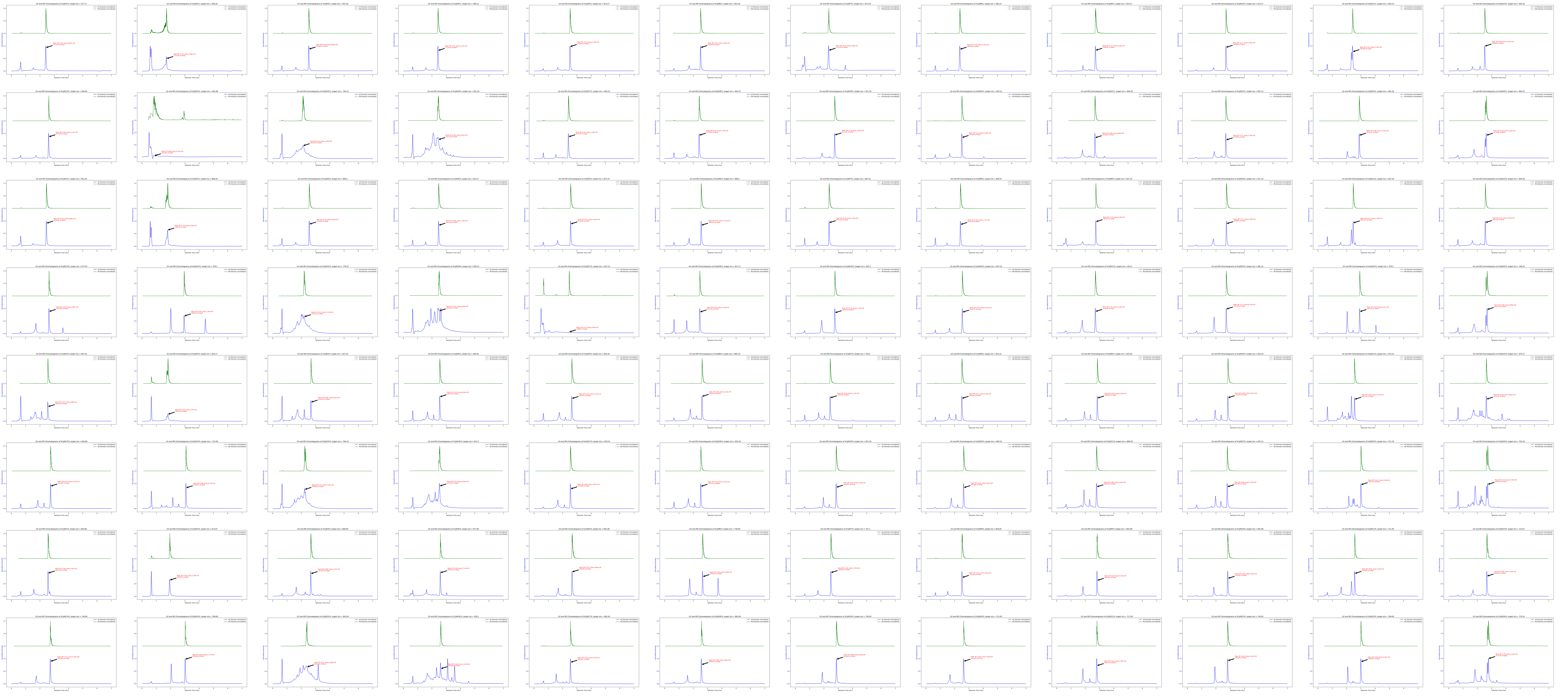

IrCp(Tz-4-P) (2+ adduct) library

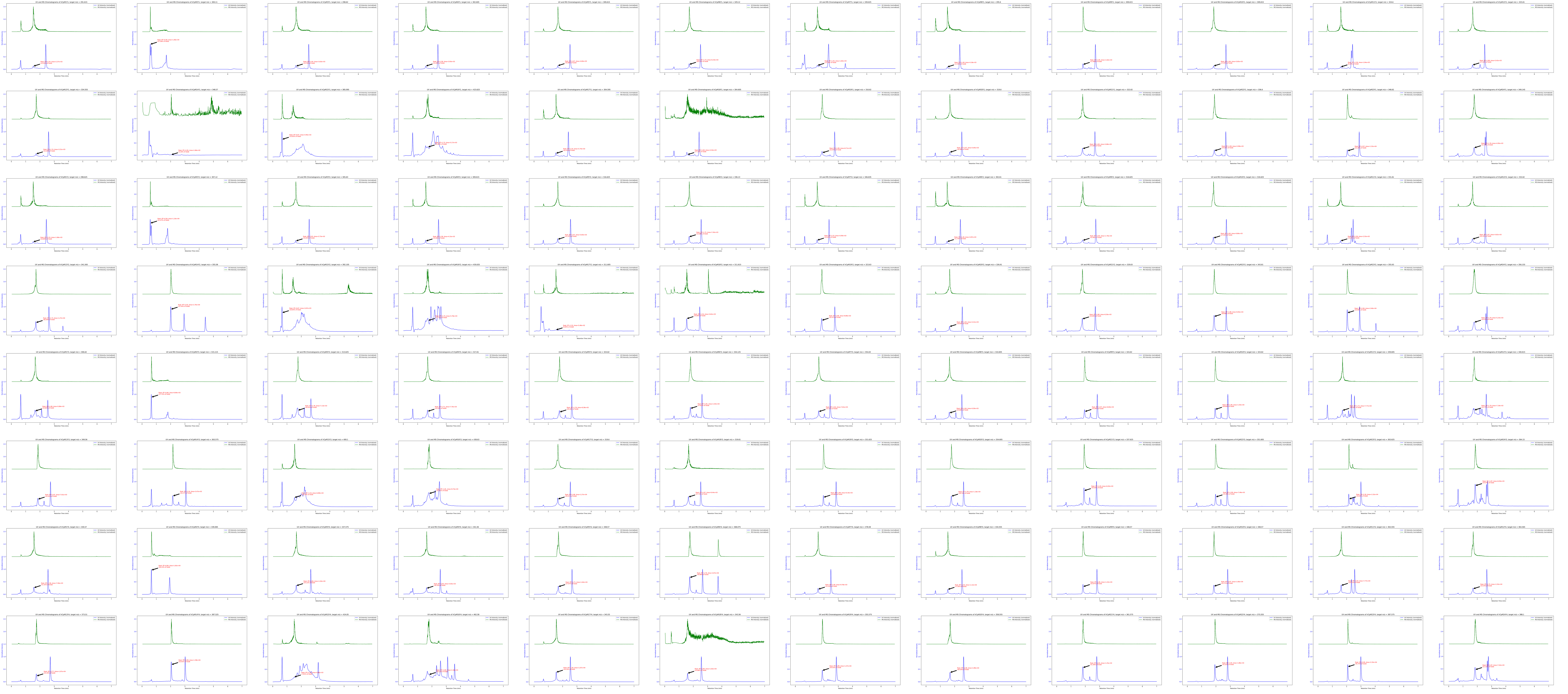

# Re(CO)3 library

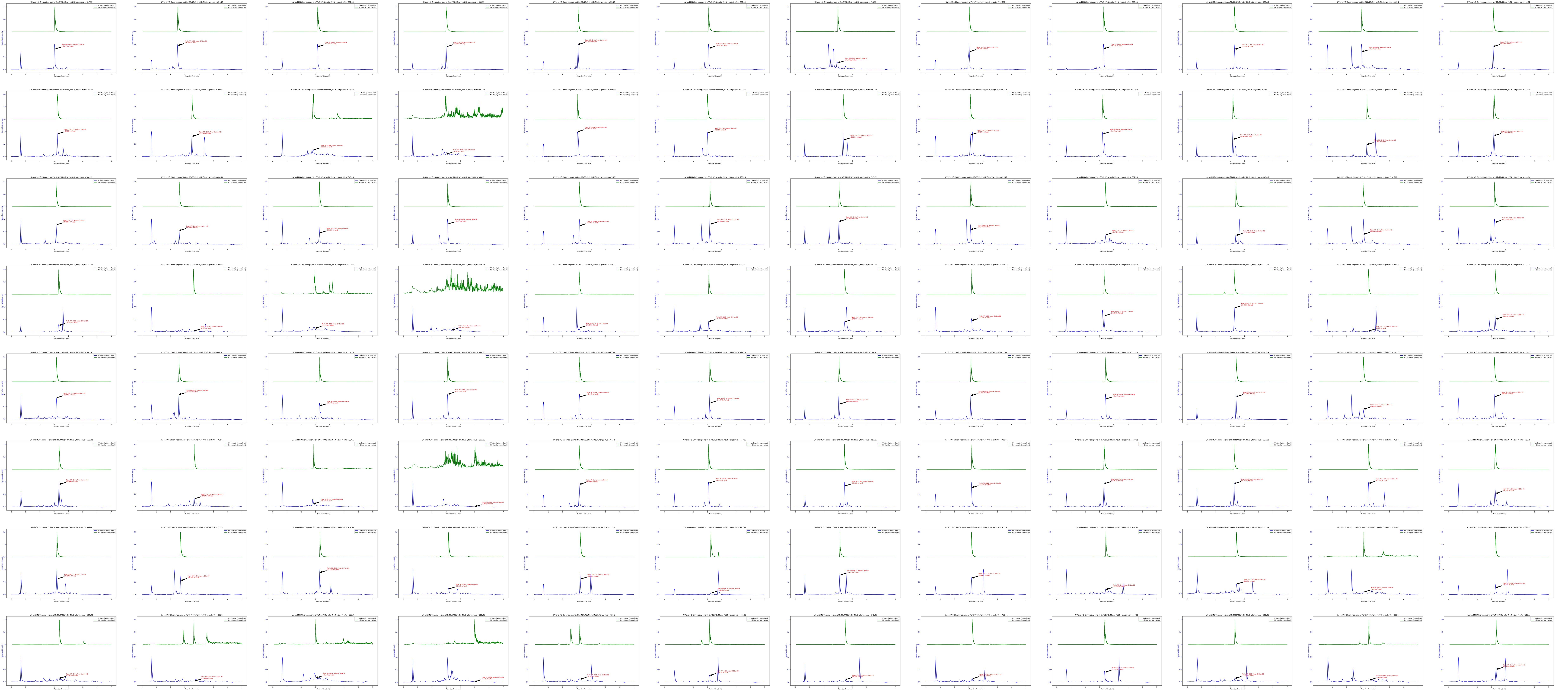

Re(CO)3 Solvent library

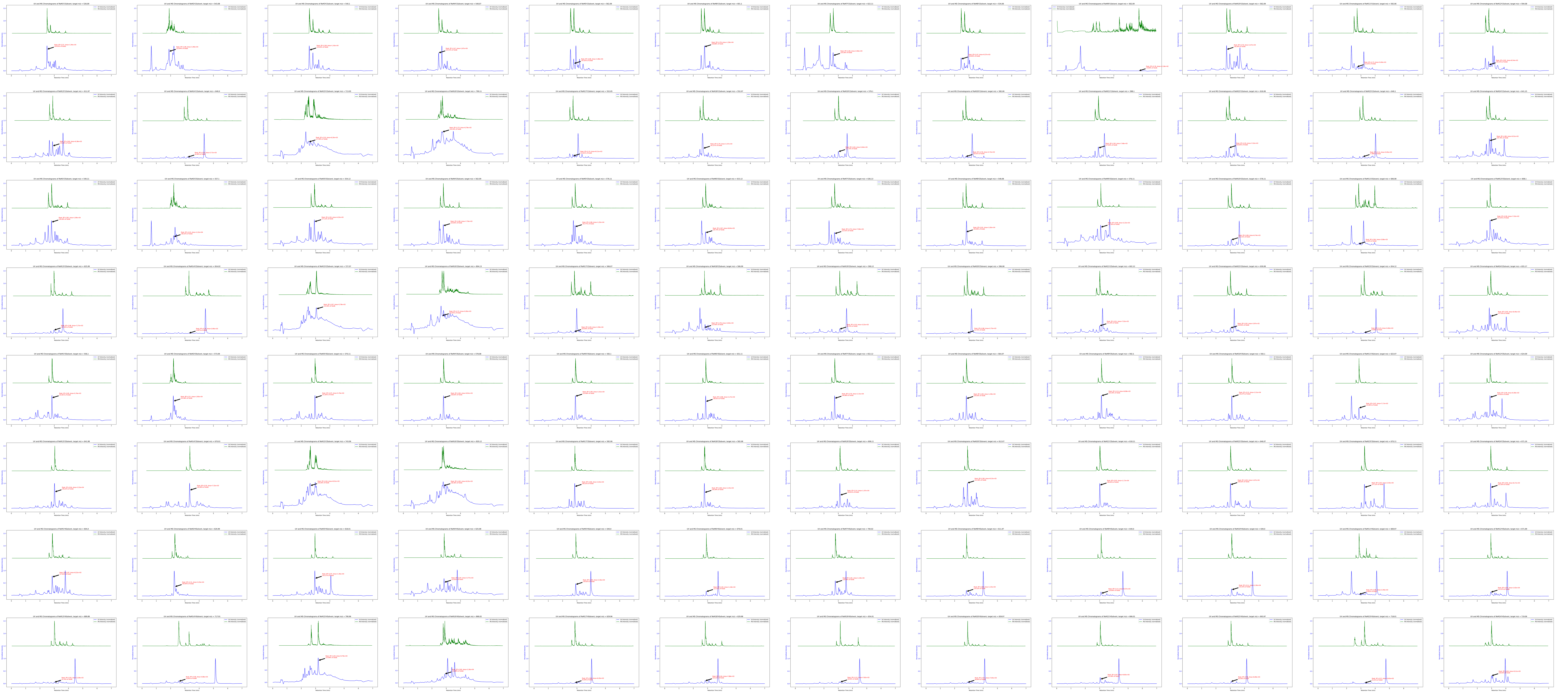

Mn(CO)3 library

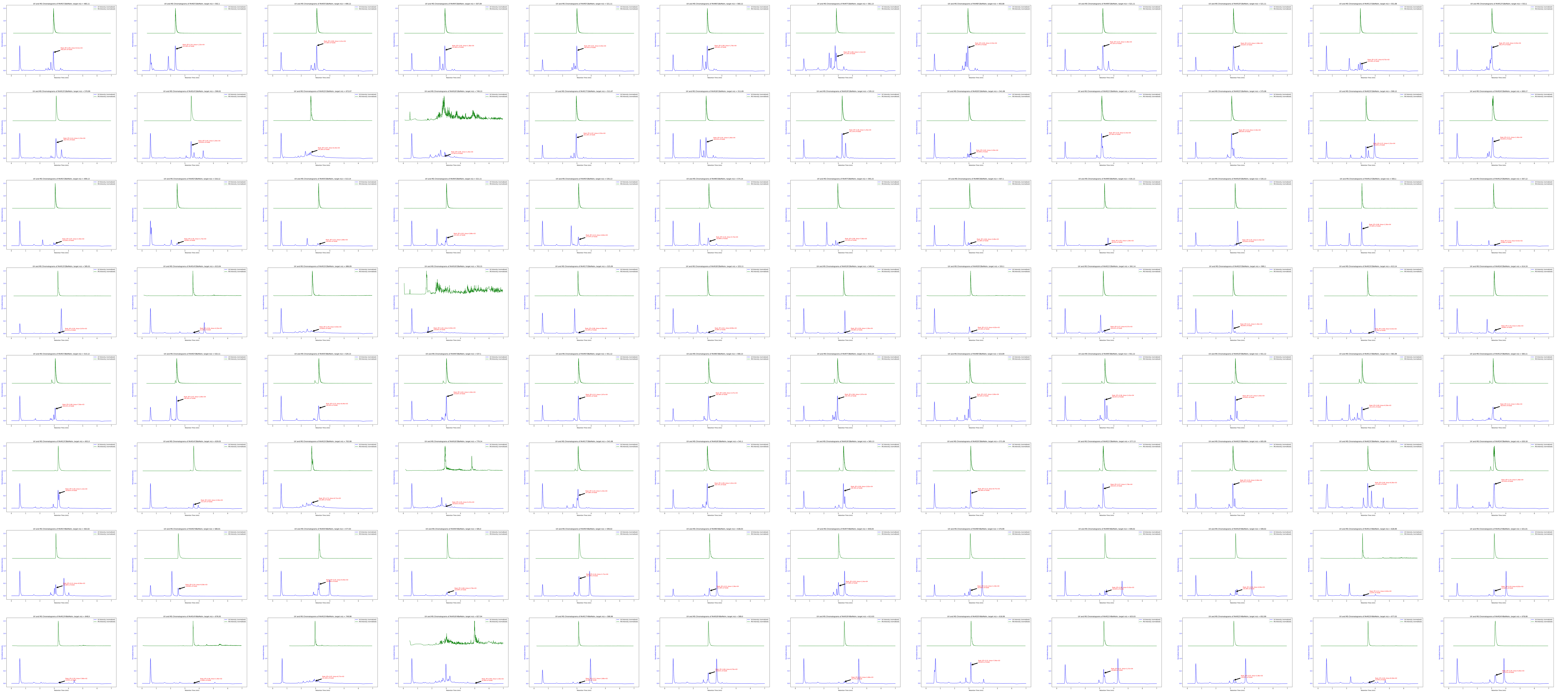

Mn(CO)3 Solvent library

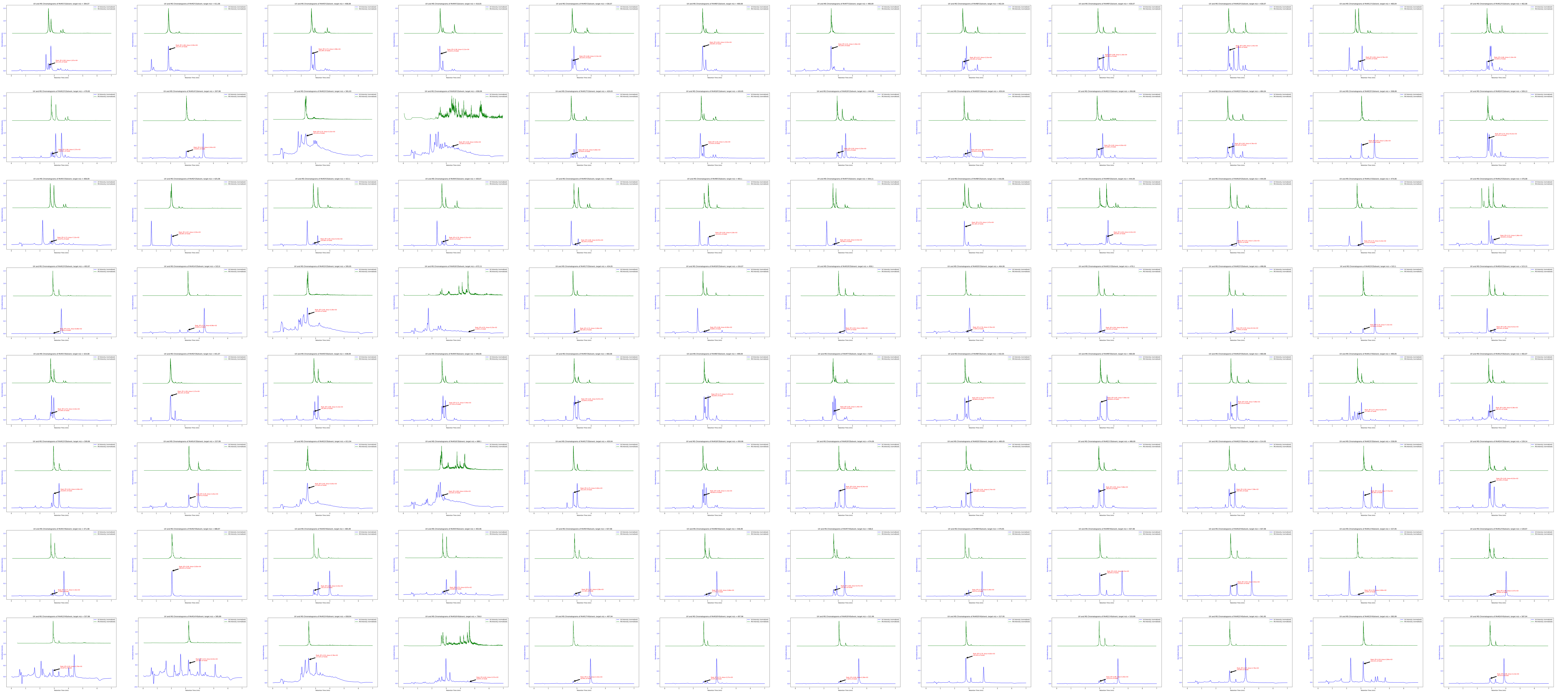

RuCy(Tz-4-P) library

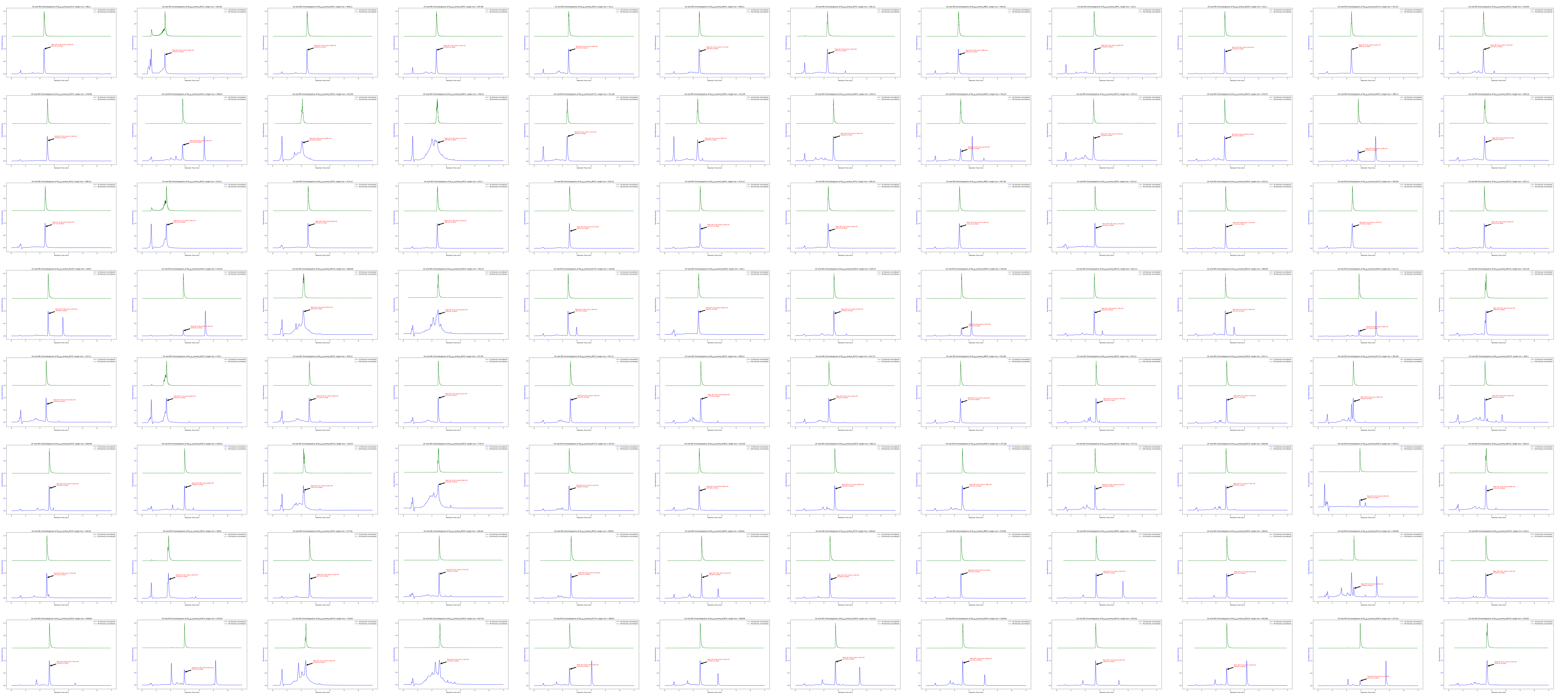

Tz-1-MP library

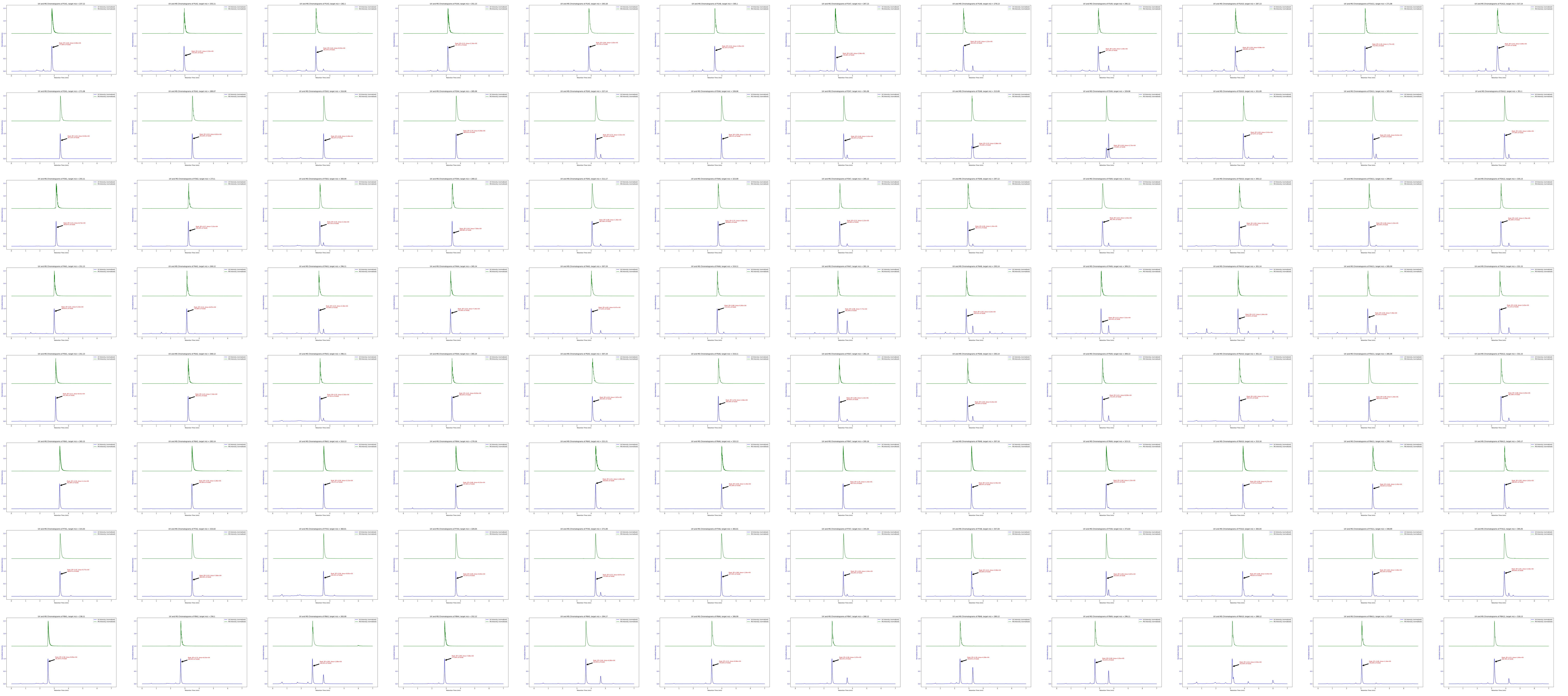

IrCp(Tz-1-MP) library

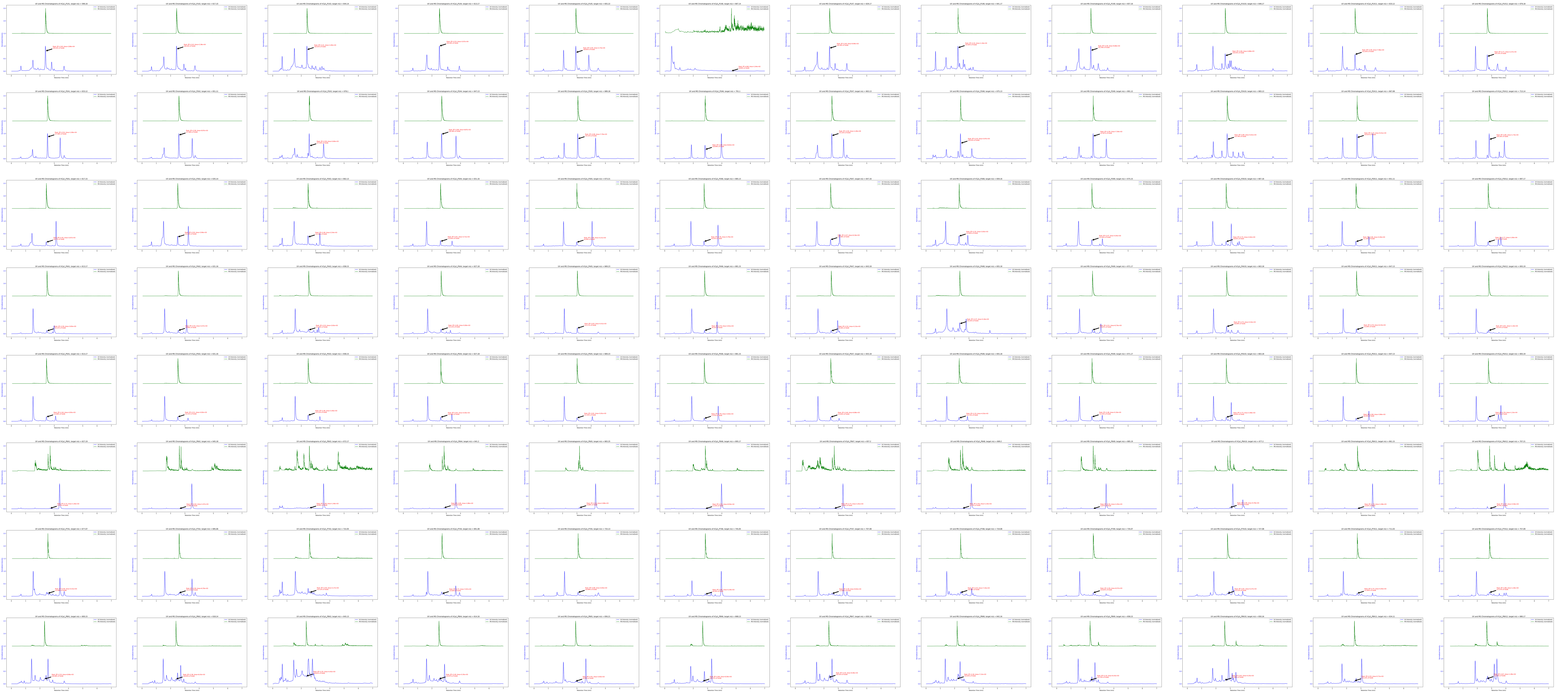

IrCp(Tz-1-MP) (2+ adduct) library

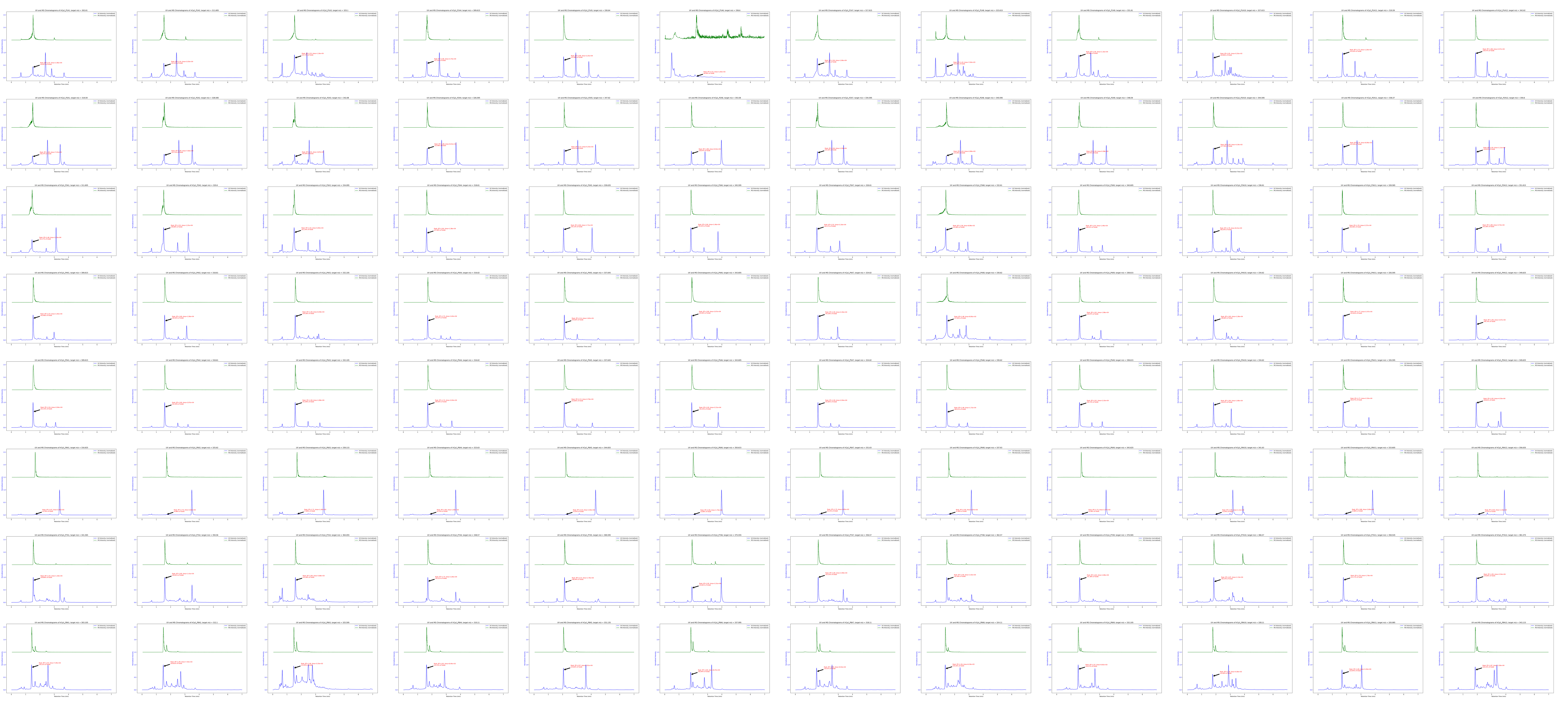

RuCy(Tz-1-MP) library

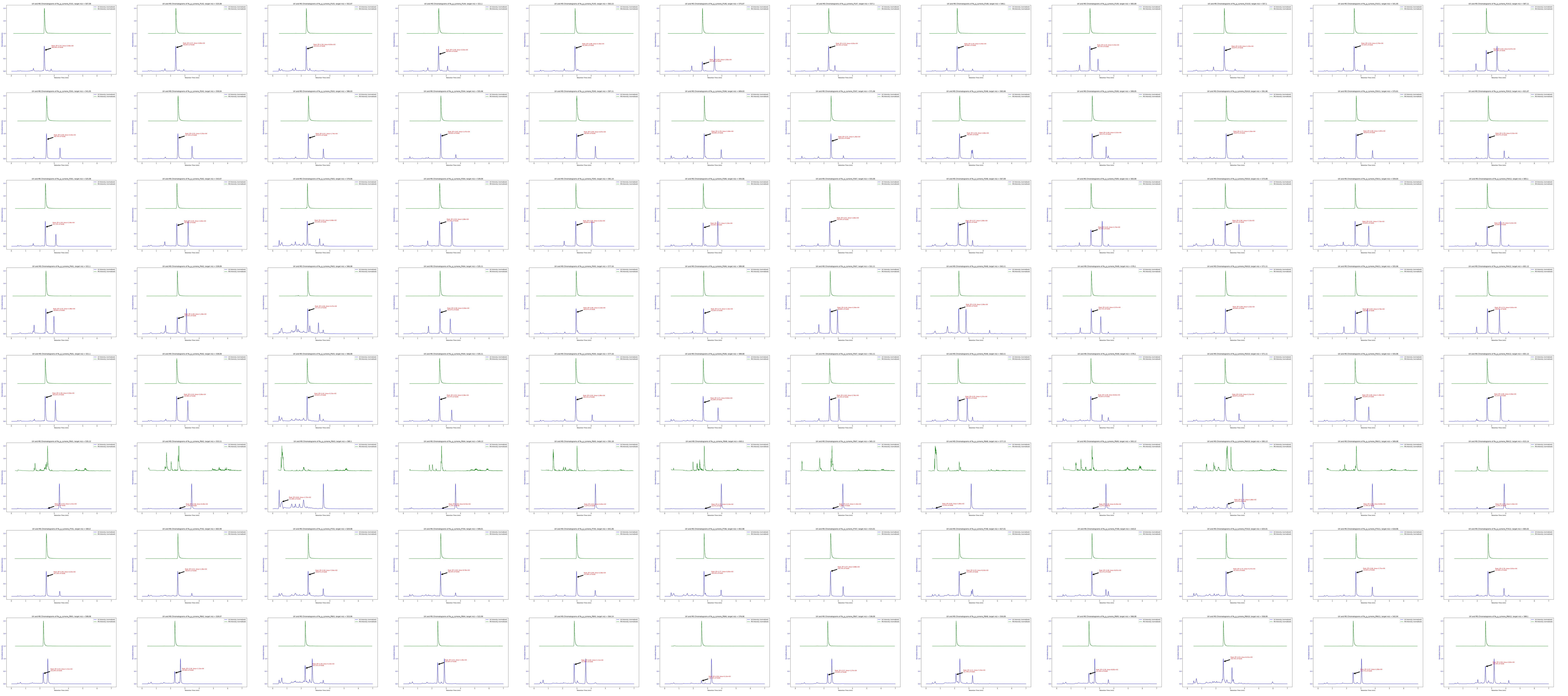

Supplement: Supplementary file 3 — Supplementary Data 1 [file 41467_2025_67341_MOESM3_ESM.pdf]
